# Traditional scientific data vs. uncoordinated citizen science effort: A review of the current status and comparison of data on avifauna in Southern Brazil

**DOI:** 10.1371/journal.pone.0188819

**Published:** 2017-12-11

**Authors:** Louri Klemann-Junior, Marcelo Alejandro Villegas Vallejos, Pedro Scherer-Neto, Jean Ricardo Simões Vitule

**Affiliations:** 1 Centro de Estudos Superiores de Itacoatiara, Universidade do Estado do Amazonas, Itacoatiara, Amazonas, Brazil; 2 Programa de Pós-Graduação em Ecologia e Conservação, Setor de Ciências Biológicas, Universidade Federal do Paraná, Curitiba, Paraná, Brazil; 3 Laboratório de Ecologia e Conservação, Departamento de Engenharia Ambiental, Setor de Tecnologia, Universidade Federal do Paraná, Curitiba, Paraná, Brazil; 4 Autonomous Environmental Analyst, Curitiba, Paraná, Brazil; 5 Museu de História Natural “Capão da Imbuia”, Rua Benedito Conceição 407, Curitiba, Paraná, Brazil; University of Colorado, UNITED STATES

## Abstract

Data generated by citizen science is particularly valuable in ecological research. If used discerningly with data from traditional scientific references, citizen science can directly contribute to biogeography knowledge and conservation policies by increasing the number of species records in large geographic areas. Considering the current level of knowledge on south Brazilian avifauna, the large volume of data produced by uncoordinated citizen science effort (CS), and the growing need for information on changes in abundance and species composition, we have compiled an updated, general list of bird species occurrence within the state of Paraná. We have listed extinct, invasive and recently-colonizing species as well as indicator species of the state’s vegetation types. We further assess the degree of knowledge of different regions within the state based on data from traditional scientific references, and the effect of including CS data in the same analysis. We have compiled data on 766 bird species, based on 70,346 individual records from traditional scientific references, and 79,468 from CS. Extinct and invasive species were identified by comparing their occurrence and abundance over a series of three time periods. Indicator species analysis pointed to the existence of three areas with bird communities typically found within the state: the Semideciduous Tropical Forest, the Tropical Rainforest and the junction of Grassland and Araucaria Moist Forest. We used rarefaction to measure sampling sufficiency, and found that rarefaction curves reached stabilization for all vegetation types except in Savanna. We observed differences in the level of knowledge of bird biodiversity among the microregions of the state, but including CS data, these differences were mitigated. The same effect was observed in other exploratory analyzes conducted here, emphasizing the fundamental importance of including CS data in macroecological studies. Production of easily accessible data and its unrestricted availability makes CS a very important tool, especially in highly diverse regions as the Neotropics, as it can offer a more accurate picture of bird composition in comparison to the exclusive use of traditional scientific references.

## Introduction

Information on bird species composition in the southern Atlantic Forest is available from several published sources, the avifauna of the state of Paraná being one of the best known in Brazil [1], [2], [3], [4], [5]. Naturalists’ field notes thoroughly describing the landscape of the state [6], [7], bird specimens housed at different research centers (see S1 Table), and studies focused on different aspects of Paraná’s avifauna [8], [9], [10], [11], are among the traditional scientific references. Part of this information has been systematically compiled and organized over the last decades. Detailed and extensive studies about Paraná’s ornithological history [4], [12], [13], [14], [15], [16], as well as local, regional and state species list compilations are available in several technical publications [12], [17].

More recently, the acquisition of ornithological records via citizen science has proven to be a valuable tool that directly contributes, for example, to biogeography studies and conservation actions in large areas and over long periods of time [18], [19], [20], [21]. This approach, as in other parts of the world [18], [22], [23], has played an important role in increasing the amount of available information and documentation of bird species occurrence in Paraná [24], [25], [26], [27], both via citizen science programs and uncoordinated citizen science effort (i.e. casual records gathered without a specific academic research goal, for instance records made by birdwatchers and nature photographers).

Despite the large amount of available information on avifauna of Paraná, there is still need for studies that simultaneously use citizen science data and evaluate the effects of its inclusion on regional avifauna knowledge. For instance, approaches that explicitly evaluate records in a spatio-temporal context, and that show differences in the avian composition in different regions within the state, are necessary to improve characterization of Paraná birds. In addition, for such characterization, and since vegetation is a known determinant factor for the establishment of bird species composition in the Neotropics [28], it is also necessary to identify species occurring in distinct vegetation types and species which are expanding their distributions and/or were introduced. Such evaluations, that report changes both in abundance and in the composition of species over space and time, are fundamentally important [29]. Once these changes are reported they can be related to factors such as human land use intensity or climate change, that make it possible to understand the potential causes for the alterations in structure, composition and spatio-temporal distribution of communities, as well as to use this knowledge in increasingly necessary conservation actions [29].

Considering the above, this work aims to congregate bird records in Paraná, accessible through published data (i.e., from traditional scientific references and gathered via uncoordinated citizen science effort—CS), and consolidate a unified and updated list indicating the vegetation types of each species’ occurrence. Also, we classify and group species according to qualitative, quantitative and temporal data within the following categories: a) locally and regionally extinct, b) recently-colonizing species (i.e., species that occur in Brazil, in the initial stages of their invasion or colonization of Paraná), c) non-native invasive species (i.e., allochthonous species, with established and expanding populations), d) native invasive species (i.e., species that occur in Paraná, with both established and expanding populations), and e) indicator species for each vegetation type. Furthermore, we compare the level of avifaunal knowledge, through the number of records, species, sources of information and localities, within geopolitical regions of the state, and finally we evaluate the effect of including CS data on all the above mentioned variables.

## Methods

### Database and species list

Data were acquired by means of an intensive search and compilation of Paraná records available from museum biological collections (M) (S1 Table) and in the technical literature (B) (S2 Table); these two sources were defined as traditional scientific references (BM). Museum specimens were accessed through online databases, and we have finished our consultation at 2015 August 31 (see S1 Table). Thus, new records might have been incorporated into these databases, in addition to some specimens that may lack online information for several reasons. However, given that we have included all available records at the time of collection consultation, we believe that our dataset includes the vast majority of known museum records from the state. Records available on Wiki Aves [27] (WA) were considered as CS data, and were retrieved in November 2015. We only used this database because all the records can be validated through photographs and sound recordings, which is not possible in the same manner (i.e. it allows independent verification of bird identifications) using other CS sources (e.g., eBird [30], Táxeus [31]). Furthermore, Wiki Aves stores more than two million documented records of Brazilian birds (data from July 2017), currently the largest repository of ornithological records in this region. We thus believe that records from this data source alone sufficiently represent the current effort of uncoordinated citizen science effort, as defined by the authors. In addition, the following records were not included in our dataset: i) records with taxonomic identities that could not be attributed to single species (i.e., citations of genera alone); ii) records with geographic coordinates that could not be retrieved from any source (e.g., maps, topographic charts, gazetteers); iii) unreliable records, geographically displaced in relation to the habitat and the known distribution of the species in Paraná (for details see comments on each record in S5 Table).

Whenever information of date and location was available in each source, we used their original information. For long-term studies, when there was no specified date for each record, we used the starting date of the sampling period. Each record was assigned geographic coordinates, either provided by the source or by consulting maps and topographic charts. We complemented geographic coordinates by consulting specific ornithological gazetteers from Brazil [32], [33]. For records in which the only geographical information available was the municipality, we used the coordinates of the municipal headquarters.

All species were classified according to type of record from which the species occurrence could be inferred, following the established criteria of the Brazilian Ornithological Records Committee [34] and Scherer-Neto et al. [17], and assigned to one of the following lists: **Primary List**–species with at least one record supported by proper documentation, in the form of full or partial specimen (i.e. feathers or body parts that allow taxa identification by independent researchers), photographs, audio or video recordings that allow independent and guaranteed assessment of the taxon’s identity; **Secondary List**–species with records not supported by proper documentation, but whose occurrence in the state is corroborated by distribution and/or habitat preferences; **Tertiary List**–species with undocumented records and with improbable occurrence in Paraná considering the known geographic range and habitat of the species. Allochthonous species without established populations in the state were assigned to the Tertiary List. Records listed in S5 Table, which need further documentation in the vegetation types listed, were excluded from our database due to being displaced from their known geographic distribution within the state. These records were not included in Tertiary List, since these species have documented records in other regions of the state.

Based on these procedures we built two bird species lists with cited occurrences in Paraná: one containing species that belong to the Primary or Secondary lists (S3 Table) and a second list with all species from the Tertiary list (S4 Table). Nomenclature and taxonomic ordering in these lists follow those proposed by the Brazilian Committee for Ornithological Records (CBRO) [35]. Corrections of the names published in the original source followed technical literature [35], [36], [37], [38]. Species from the Primary and Secondary lists are accompanied by the sources of information, the number of records that exist in each vegetation type, and status alterations in relation to the latest state list available [17] (i.e., new records for the state, and species that were transferred from the Secondary or Tertiary lists to the Primary list; S3 Table).

**Fig 1:**
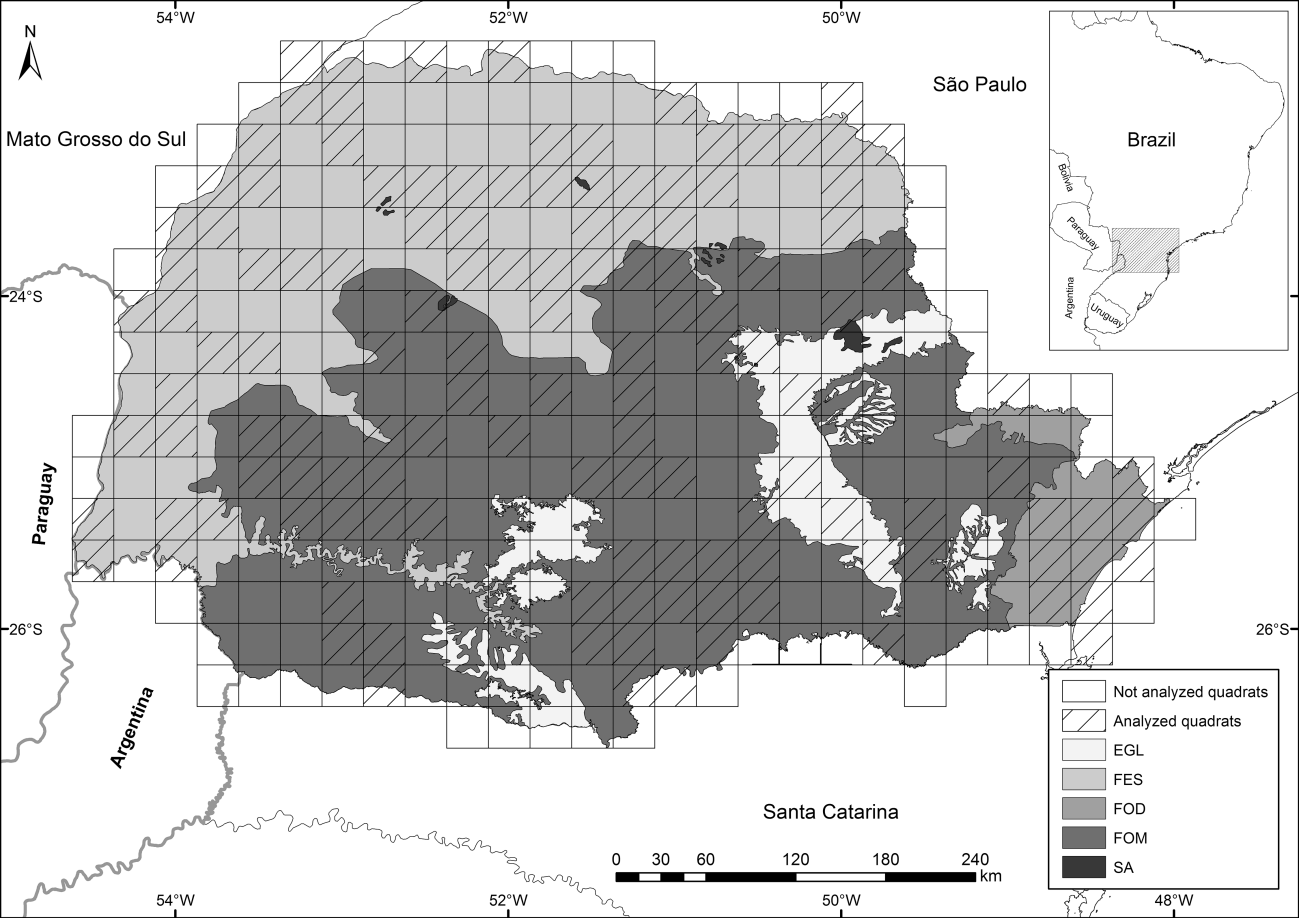
Phytogeographic map of Paraná state and quadrat divisions used for analyses. Vegetation types of the state of Paraná depicting quadrats analyzed, in a different color. For the analyses, only records obtained in the state of Paraná were used. Vegetation types: EGL–Grassland; FES–Semideciduous Tropical Forest; FOD–Tropical Rainforest; FOM–Araucaria Moist Forest; SA–Savanna.

To determine vegetation type for each record we overlaid individual records on a phytogeographic map of Paraná state [39] (Fig 1) using QGIS software [40]. Vegetation types included in our analysis were: i) Grassland (EGL), ii) Semideciduous Tropical Forest (FES), iii) Tropical Rainforest (FOD), iv) Araucaria Moist Forest (FOM), and v) Savanna (SA). Waterbirds and shorebirds, with records outside the coastal zone, were assigned according to the vegetation type designated above. Seabirds (Sphenisciformes, Procellariiformes, some of the Suliformes, and some of the Charadriiformes; S3 Table) were not attributed to any of these vegetation types and were, therefore, excluded from quadrat and indicator species analyses, described below.

To assess if bird species composition is related to vegetation type, we divided the study area in 15’x15’ lat-long quadrats. In order to avoid using under-sampled quadrats (bias due to low richness), or that harbor a composition of species representative of more than one vegetation type (bias due to the mixture of distinct fauna compositions due to spatial scale), we only analyzed quadrats containing at least 50 bird species recorded, regardless of the number of individual bird records, and having a single vegetation type covering over 80% of its total area, measured with QGIS software (Fig 1). Incomplete quadrats (i.e., with part of their area in the ocean or in other states) that meet the two inclusion criteria (i.e., at least 50 bird species recorded and 80% of the land area in Paraná state covered by a single vegetation type) were used in the analysis. Thus, Savanna (SA) was not included in quadrat analyses due to its small coverage area in the state. We used Permutational Multivariate Analysis of Variance (PERMANOVA) [41] to assess differences amongst bird species composition using vegetation type as the predictor variable. A dissimilarity matrix was built by only considering binary data of species’ presence in each quadrat, using the Raup-Crick index [42]. This dissimilarity index should be able to handle unknown and variable sample sizes, using the available species pool and each quadrat’s richness to perform random permutations and determine how often a comparable level of similarity occurs [43]. We evaluated species composition differences among quadrats by plotting the first two axes of a Principal Coordinates Analysis (PCoA) [44]. This analysis was performed twice, excluding and including CS data from our dataset, in order to assess the influence of CS when interpreting bird community composition differences.

We determined indicator species (i.e., species restricted to one or a few habitat types [45]) for each vegetation type by calculating the species’ Indicator Value [46], considering each year separately, between 1820 and 2015, thus excluding years for which no bird records were present (see S6 Table). The two components of the indicator value (i.e., specificity and fidelity) were examined [45], [46], and the specificity value, when equal to 1.0, was used to assign species that are exclusive to each vegetation type. We performed these analyses using the *indval* (= multipatt) function from *indicspecies* package in software R [45], [47], [48], [49]. This function finds the indicator species for each vegetation type (i.e., EGL, FES, FOD, FOM, SA) and for every possible combination of vegetation types (e.g., EGL+FOM, FOM+FOD+FES) [48], [46]. This procedure allows the identification of vegetation types or combinations of vegetation types that have characteristic bird communities (i.e., high number of indicator species and exclusive species). The analysis of all possible combinations is informative given that even spatially discontinuous vegetation types may have common indicator and exclusive species due to factors other than proximity, such as ecological or historical factors (e.g., dispersal history of vegetation types in southern Brazil).

To detect local or regional extinctions, recently-colonizing and invasive species, both native and non-native, all records from each vegetation type were grouped into three time periods: a) 1820–1959; b) 1960–1989; and c) 1990–2015. These time periods were chosen based on critical moments (and the elapsed time of those moments) in the history of land use development in Paraná, namely according to the percentage of forest areas that were replaced by agriculture and pastures (see [50]): a) less than 65% of the forest habitat in the state had been suppressed [51], [52]; b) from 65% to 85% of the forest had been suppressed [51], [52]; and c) more than 85% of the forest had been suppressed [53]. Thus, the time period from 1960 to 1989 is characterized by maintaining forest cover at nearly the extinction threshold (i.e., around 30% of natural vegetation cover [54]), and we expect local and regional extinctions to occur from this point forward. In this manner, these time periods allow us to evaluate the effects of anthropic impact on bird community composition, especially with regards to species turnover.

Species for which there were no records in the second and/or third time periods were considered extinct in a specific vegetation type or in the whole state, as appropriate. Species that inhabit open vegetation formations and/or anthropogenic habitats, with known populations occurring in neighboring areas around the Paraná (i.e., Argentina, Paraguay and the states of Santa Catarina, São Paulo and Mato Grosso do Sul in Brazil), autochthonous and with first records documented after 1989, were classified as recently-colonizing. Only species that inhabit open habitats were included in this category, since those species were likely the most capable to reach and colonize anthropogenic habitats that prevail in Paraná (see [55] and [56]). Allochthonous species, with over 20 records and/or recorded at five or more sites, were classified as non-native invasive species with established populations in Paraná.

To determine native invasive species, we evaluated the increase in the number of records within each vegetation type during the three time periods. Since the number of records is closely linked to the number of individuals in a species (i.e., its relative abundance), to sampling effort, and to the species’ detectability, we expect that an increase in the number of records for any given species will represent an increase in the number of individuals and/or in sampling effort. Thus, considering that the number of records can be used as a proxy of the sampling effort, the variation in sampling effort between pairs of time periods was calculated, for each vegetation type, by dividing the total number of records from the more recent time period by the number of records from the older time period. We considered that increases in bird records represented increases in population numbers in each vegetation type and time periods in the following cases: i) when the increase in the number of records, among the three time periods, was greater than the sampling effort variation in only one vegetation type, we considered that the species local population increased; ii) when the increase occurred in two or more vegetation types, among the three time periods, we considered that the species regional population increased, and it expanded its range (i.e., a native invasive species, see [57], [58]). We did not conduct this analysis on SA due to the lack of records prior to 1960 and to their scarcity between 1960 and 1989.

We used Wilcoxon signed-rank tests to compare the effect of including CS data on the number of species, the number of locally or regionally extinct species, the number of native invasive species, and the number of indicator species in each vegetation type.

### Avifauna knowledge level

To assess the adequacy of the sampling effort applied in Paraná for the characterization of its avifauna, rarefaction-by-sample curves [59] were constructed using the species listed in the primary and secondary lists and the information on their occurrence dates. The curves were constructed using ten-year time interval as sample units, starting at the first compiled record found. We built curves considering data from the entire state and, separately, for each vegetation type, in the *Past* software (Paleontological Statistics, ver. 1.34; [60]). Two sets of curves were created, both including and excluding CS data.

Differences in the level of knowledge of avifauna in the different regions of the state were evaluated through representation, using QGIS software [40], of the number of records in each microregion of Paraná (*sensu* [61]). Microregions were used, instead of other administrative divisions or of vegetation types, because they allow evaluation of bird diversity at a smaller scale in the state. In each microregion the following variables were represented: i) number of species; ii) number of records; iii) number of sources of information (i.e., literature references, museums with deposited specimens and record owners on Wiki Aves); and iv) number of sites (localities or municipalities) with at least one bird record. For every one of the four variables evaluated, a numeric class was attributed, representing the level of knowledge: 1) Very Low, 2) Low, 3) Average, 4) High, 5) Very High. In order to reduce variance within the classes and augment variance between the classes, we used the Jenks natural breaks method [62] to establish limits for the five classes above, which resulted in the following thresholds: i) *Number of species*—Very Low: ≤43, Low: 44–117, Average: 118–204, High: 205–338, Very High: ≥339; ii) *Number of records*—Very Low: ≤537, Low: 538–1443, Average: 1444–2963, High: 2964–7660, Very High: ≥7661; iii) *Number of sources of information*—Very Low: ≤6, Low: 7–14, Average: 15–27, High: 28–54, Very High: ≥55; iv) *Number of sites with at least one bird record*—Very Low: ≤10, Low: 11–21, Average: 22–36, High: 37–71, Very High: ≥72. The microregions are composed of mosaics of different vegetation and land use types, and none of the microregions are covered exclusively by a single species-poor vegetation type (i.e., EGL or SA). Thus, the difference in richness between vegetation types should not affect richness differences among microregions in this analysis.

To determine the overall level of knowledge on the avifauna in each microregion, we used the arithmetic mean of the numeric classes of the four variables evaluated. To determine the level of knowledge in the whole state, we used the arithmetic mean of the level of knowledge of every microregion. We used Wilcoxon signed-rank tests to compare the effect of including CS data on the number of species, number of records, number of sources of information and number of sites.

## Results

### Species list

We found 766 bird species with known occurrences in the state, included in the primary (n = 726) and secondary (n = 41) lists (S3 Table), and other 50 species included in the tertiary list (S4 Table). The bird database with assigned coordinates included 70,346 records ranging from 1820 to 2015, corresponding to 747 species, which derived from traditional scientific references (BM), and 79,468 records ranging from 1986 to 2015 (95% of the which ranged from 2010 to 2015), belonging to 675 species, which derived from CS data. We excluded from this database 371 records found in the literature due to: i) the impossibility of attributing geographic coordinates; ii) records being unreliable, geographically displaced with respect to the habitat and the known distribution of the species in Paraná (for details see comments on each record in S5 Table); and iii) species being only included in the tertiary list of birds of the state (S4 Table).

**Fig 2:**
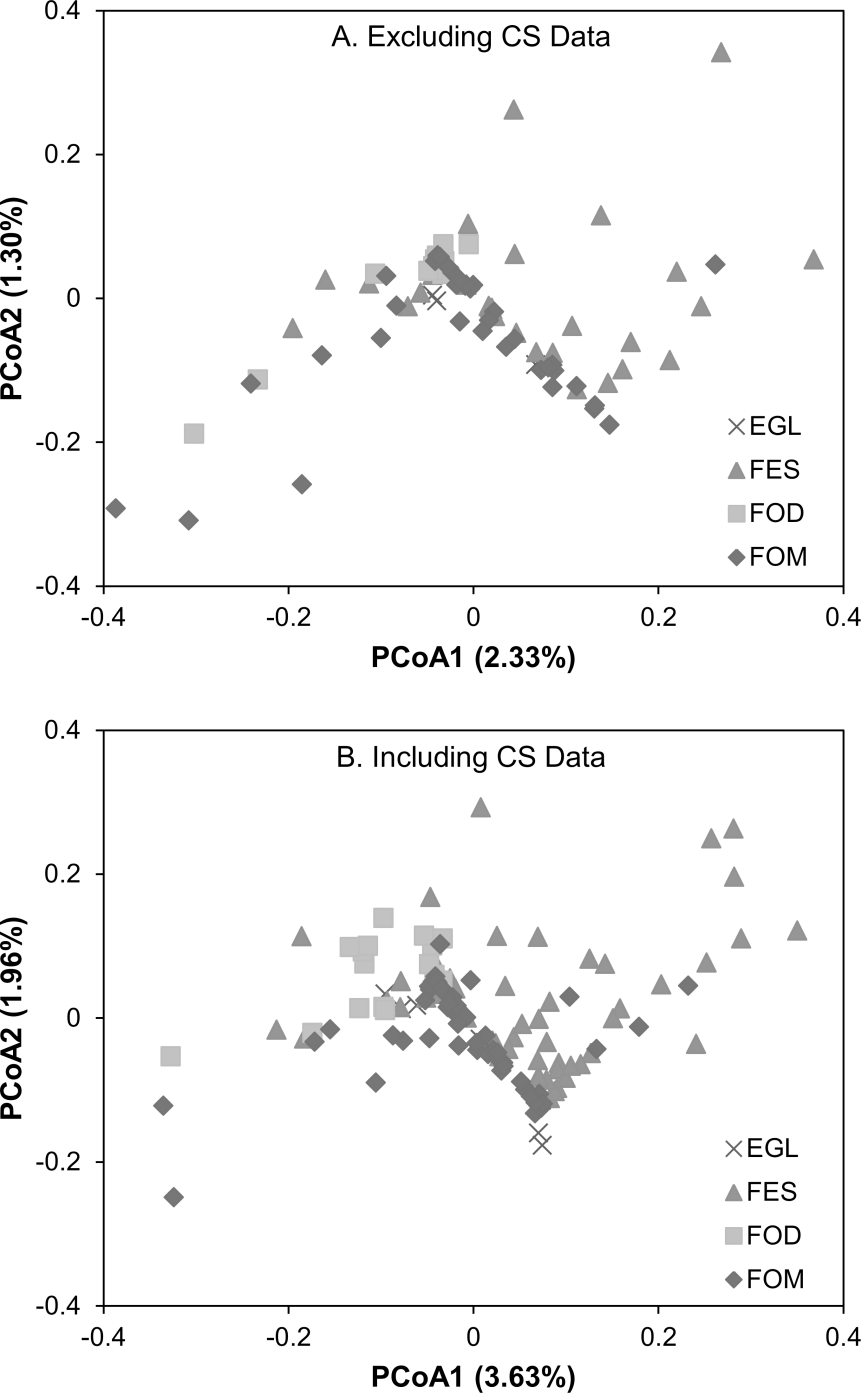
Analysis of avifaunal composition among vegetation types. Scores from the first two axes of a PCoA applied on a Raup-Crick dissimilarity matrix, showing differences in bird communities in Paraná among vegetation types, both excluding (A) and including (B) CS data. Vegetation types: EGL—Grassland, FES—Semideciduous Tropical Forest, FOD—Tropical Rainforest, FOM—Araucaria Moist Forest.

The number of species with records in each vegetation type was 475 (excluding CS data—BM) and 506 (including CS data—BM+CS) in EGL; 535 (BM) and 569 (BM+CS) in FES; 497 (BM) and 523 (BM+CS) in FOD; 501 (BM) and 528 (BM+CS) in FOM; and 244 (BM) and 277 (BM+CS) in SA. Other 48 (BM) and 53 seabirds (BM+CS) are also present in the species list. PERMANOVA analysis indicated that bird composition differs significantly among the vegetation type, both excluding (F = 7.37, p = 0.001; Fig 2A) and including CS data (F = 9.59, p = 0.001; Fig 2B). The number of indicator species in each vegetation type, and in different combinations of types, is presented in Table 1, and the corresponding list of species in S7 Table.

**Table 1:** Indicator species of each vegetation type. Number of species, indicator species and exclusive species for each vegetation type individually and in different combinations of types, both excluding (BM) and including CS data (BM+CS). Vegetation types: EGL–Grassland; FES–Semideciduous Tropical Forest; FOD–Tropical Rainforest; FOM–Araucaria Moist Forest; SA–Savanna. Data presented in descending order of number of indicator species (BM+CS).

Grouping	BM			BM+CS		
**One typology**	**Total N° SP.**	**N° Indicators SP.**	**N° Exclusive SP.**	**Total N° SP.**	**N° Indicators SP.**	**N° Exclusive SP.**
FOD	497	81	38	523	103	44
FES	535	45	27	569	80	37
SA	244	25	3	277	14	2
FOM	501	3	0	528	4	0
EGL	475	4	0	506	3	1
**Two typologies**						
EGL+FOM	536	48	5	563	41	5
FES+FOD	650	10	2	676	23	3
FES+FOM	617	2	0	640	12	2
EGL+SA	484	5	1	515	8	1
FES+SA	553	12	1	584	6	1
EGL+FOD	593	0	0	614	2	0
FOD+FOM	595	5	1	616	2	1
FOD+SA	545	3	0	567	2	0
EGL+FES	617	0	0	641	0	0
FOM+SA	511	1	0	540	0	0
**Three typologies**						
EGL+FES+FOM	637	3	1	656	27	3
EGL+FOD+FOM	619	15	0	641	24	3
EGL+FOM+SA	540	46	0	568	13	1
FES+FOD+FOM	680	7	1	700	12	1
FES+FOM+SA	623	3	0	645	3	0
EGL+FES+SA	620	0	0	644	1	0
EGL+FES+FOD	688	2	0	704	0	0
EGL+FOD+SA	601	0	0	621	0	0
FES+FOD+SA	663	4	0	685	0	0
FOD+FOM+SA	604	0	0	626	0	0
**Four typologies**						
EGL+FES+FOD+FOM	696	4	2	711	44	11
EGL+FES+FOM+SA	640	31	5	659	38	6
EGL+FOD+FOM+SA	623	10	0	645	4	0
FES+FOD+FOM+SA	686	0	0	704	1	0
**Total**	**699**	**369**	**87**	**713**	**467**	**122**

When only considering records from traditional scientific references (BM), we identified 12 extinct species in the whole state and 48 considering each vegetation type separately. These numbers decreased when CS data was included (n = 10 and n = 25 respectively; Table 2). The same species can be considered extinct in more than one vegetation type at the same time period, and thus the total number of species extinct in a given time period is not the direct sum of species from all vegetation types (a complete list of extinct species is presented in S8 Table). No extinct species were identified in SA, both excluding and including CS data. The number of extinct species in all vegetation types, comparing BM and BM+CS, are similar in 1960–1989 and lower in 1990–2015. Also, considering only CS data, the number of extinctions in each vegetation type was higher in 1960–1989 and lower in 1990–2015. Species with marginal occurrences in each vegetation type, migrants, partial migrants and species that perform seasonal dispersal, nomadic species or other with poorly known mobility [63] were excluded from the list (63 in total). The complete list of extinct species, indicating excluded species, is presented in S8 Table.

**Table 2:** Extinct bird species in Paraná. Number of extinct bird species (per time period and total) and percentage of the total number of extinct species relative to the total number of species in each vegetation type, both excluding (BM) and including CS data (BM+CS). Some species were extinct in more than one vegetation type, and a complete list of extinct species is presented in S8 Table. Vegetation types: EGL–Grassland; FES–Semideciduous Tropical Forest; FOD–Tropical Rainforest; FOM–Araucaria Moist Forest; SA–Savanna. Data presented in descending order of total number of regionally extinct species.

Vegetation Types	Number of regionally extinct species							
	BM				BM+CS			
	1960–1989	1990–2015	Total N° SP.	%	1960–1989	1990–2015	Total N° SP.	%
**FES**	19	9	28	5.2	16	3	19	3.3
**FOD**	1	15	16	3.2	0	3	3	0.6
**FOM**	3	1	4	0.8	2	1	3	0.6
**EGL**	3	1	4	0.8	1	0	1	0.2
**Extinction in State**	6	6	12	1.6	4	6	10	1.3

We identified 14 species that have recently colonized the Paraná (*Aratinga nenday*, *Buteo nitidus*, *Campylorhynchus turdinus*, *Fluvicola nengeta*, *Gampsonyx swainsonii*, *Icterus croconotus*, *Myiopsitta monachus*, *Myiozetetes cayanensis*, *Paroaria dominicana*, *Rhea americana*, *Saltator coerulescens*, *Schistochlamys melanopis*, *Theristicus caerulescens* and *Tyrannus albogularis*) as well as three non-native invasive species (*Columba livia*, *Estrilda astrild* and *Passer domesticus*; S3 Table). Two native allochthonous species with established populations were also detected: *Paroaria coronata* (all known records in the state are considered allochthonous) and *Amazona aestiva* (allochthonous records correspond to Grasslands, and all records from Tropical Rainforest and Araucaria Moist Forest; Semideciduous Tropical Forest records of this species are considered autochthonous). Two additional species, *Brotogeris tirica* and *Myiopsitta monachus*, were not classified as allochthonous, even though some local introductions occurred in the state. This decision was taken due to historical records and/or their occurrence in areas neighboring these records, making it impossible to accurately determine whether the records represent introduced birds.

Among the three time periods, we identified 151 (BM) and 163 (BM+CS) species with a number of records higher than that expected from the corresponding sampling effort increment in Paraná. Of these, 94 (BM) and 110 (BM+CS) were classified as species with a local population increase and 57 (BM) and 53 (BM+CS) as native invasive species (S9 Table). Considering vegetation types separately, 47 (BM) and 45 (BM+CS) species were classified as native invasive in FES, 37 (BM) and 34 (BM+CS) in FOM, 30 (BM) and 30 (BM+CS) in EGL and 31 (BM) and 18 (BM+CS) in FOD. The inclusion of CS data rendered differences in the number and identity of species in both categories, i.e., local population increase and native invasive species (S9 Table).

**Fig 3:**
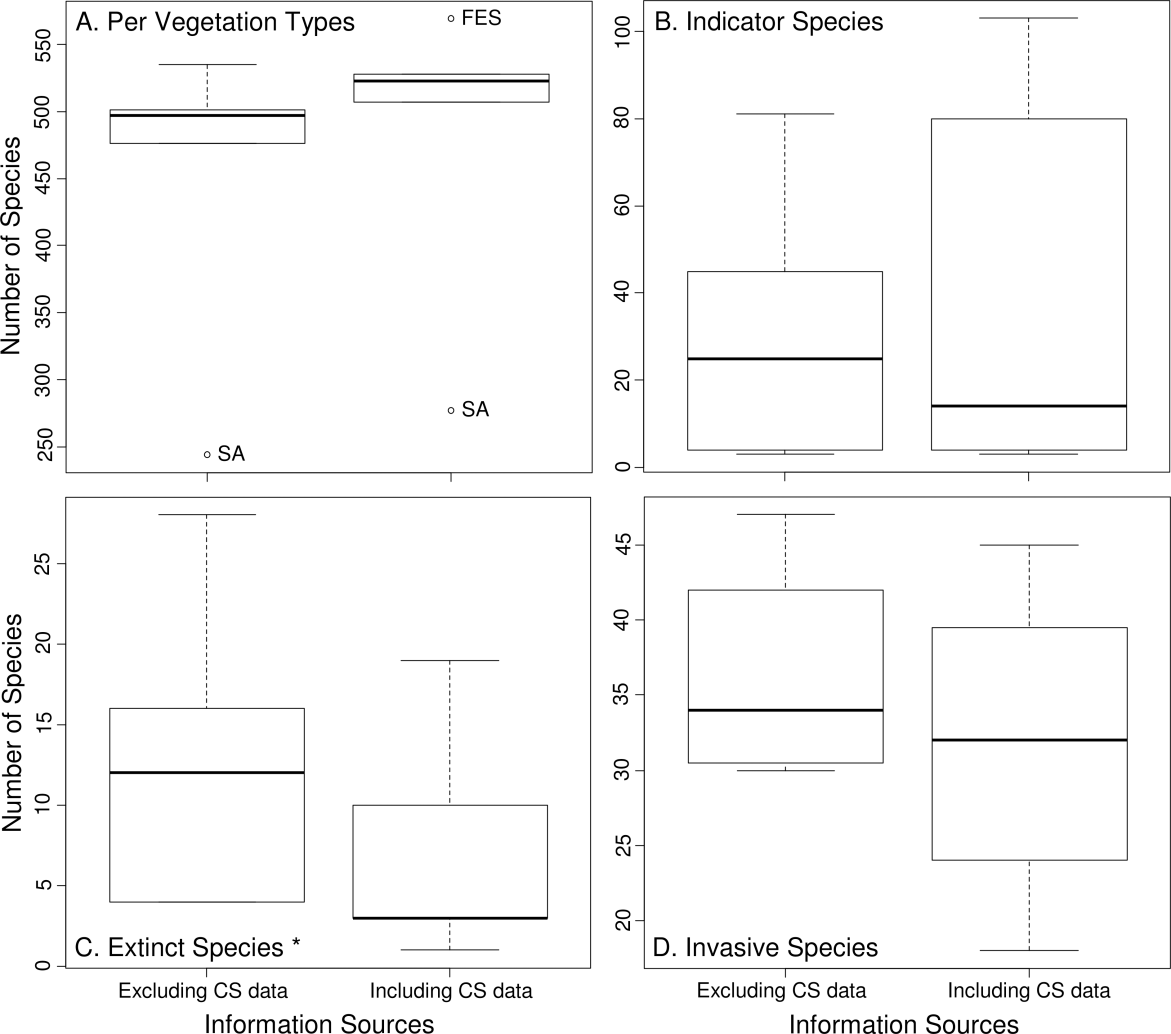
Comparisons between traditional scientific references data by themselves and including CS data. Boxplots comparing: (A) number of species per vegetation type, (B) number of indicator species per vegetation type, (C) number of extinct species per vegetation type and in the whole state of Paraná, and (D) number of native invasive species per vegetation type, both excluding and including CS data. * Statistically significant differences. Open circles represent extreme values found for some vegetation types. SA—Savanna, FES—Semideciduous Tropical Forest.

Comparison between datasets excluding and including CS data showed significant differences in species richness by vegetation type (W: 15, z: 2.023, *p*<0.05, Fig 3A) and in the number of locally and/or regionally extinct species (W: 15, z: 2.023, *p*<0.05, Fig 3B). There was no significant difference in the number of native invasive species (W: 6, z: 1.604, *p*>0.05, Fig 3C) or indicator species in each vegetation type (W: 10.5, z: 0.813, *p*>0.05, Fig 3D).

### Avifauna knowledge level

**Fig 4:**
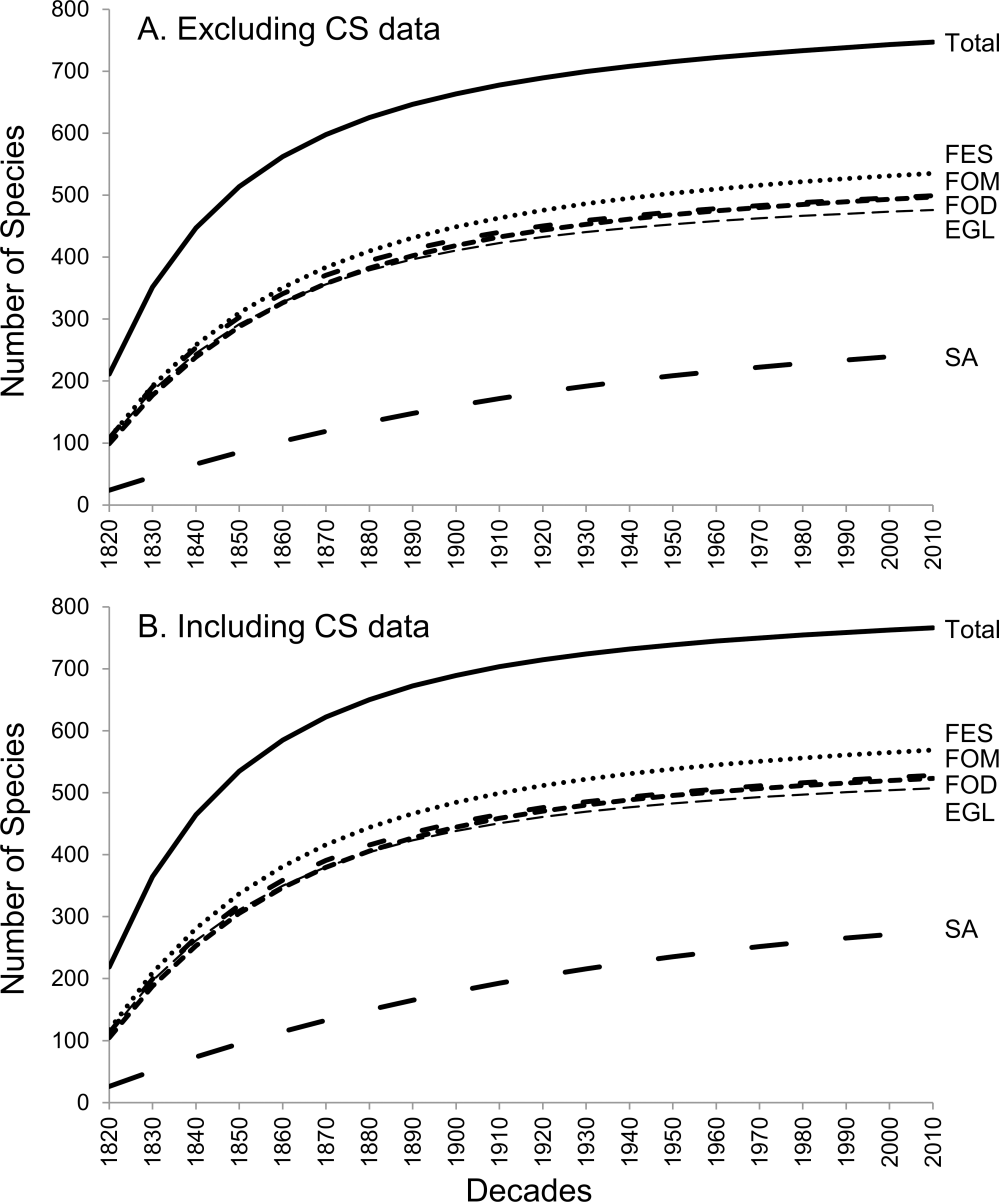
Sample-based rarefaction curves comparing bird species richness in Paraná. Specimen occurrences were compiled from the primary and secondary lists, using decades as sample units and starting at 1820, (A) excluding CS data, and (B) including CS data. EGL—Grassland, FES -Semideciduous Tropical Forest, FOD—Tropical Rainforest, FOM—Araucaria Moist Forest, SA—Savanna, Total—considering the whole state of Paraná.

The first available records on Paraná avifauna date from 1820, from Johann Natterer’s expedition to Brazil [1], [64]. Sample-based rarefaction curves, created decade by decade starting in 1820, showed a trend for stabilization when considering the entire state, and for each vegetation type, with the exception of SA (Fig 4). This result holds true regardless of the inclusion of CS data.

**Fig 5:**
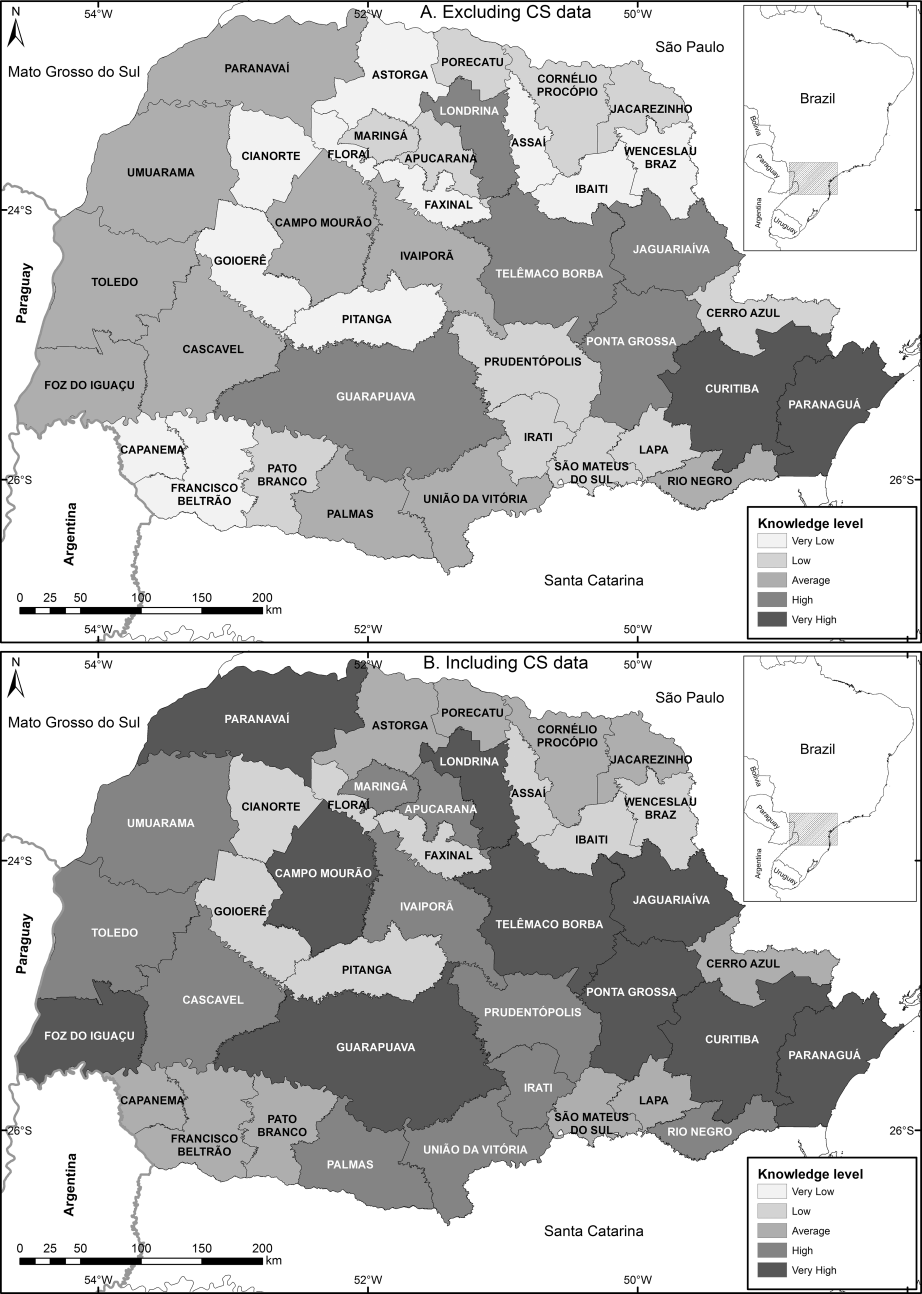
Mapping the level of avifaunal knowledge in Paraná. Maps indicate the level of avifaunal knowledge in the microregions of Paraná state considering (A) only data from traditional scientific references, and (B) including CS data.

The level of avifaunal knowledge in Paraná showed large variations among microregions, ranging from 2 species, 2 publications and 3 records in the microregion of Capanema; to 545 species, 154 publications and 7,660 records in the microregion of Paranaguá, considering only data from traditional scientific references (S10 Table). When we included CS data in this analysis, a notable increase was observed for all variables (e.g., Capanema microregion now has 222 species, 2 publications, 20 owners of images and sounds on Wiki Aves and 632 records; S10 Table). This same pattern was observed throughout the state, with the level of regional knowledge going from Low (BM) to High (BM+CS). The location of the microregions and their classification for the level of avifauna knowledge are shown in Fig 5.

**Fig 6:**
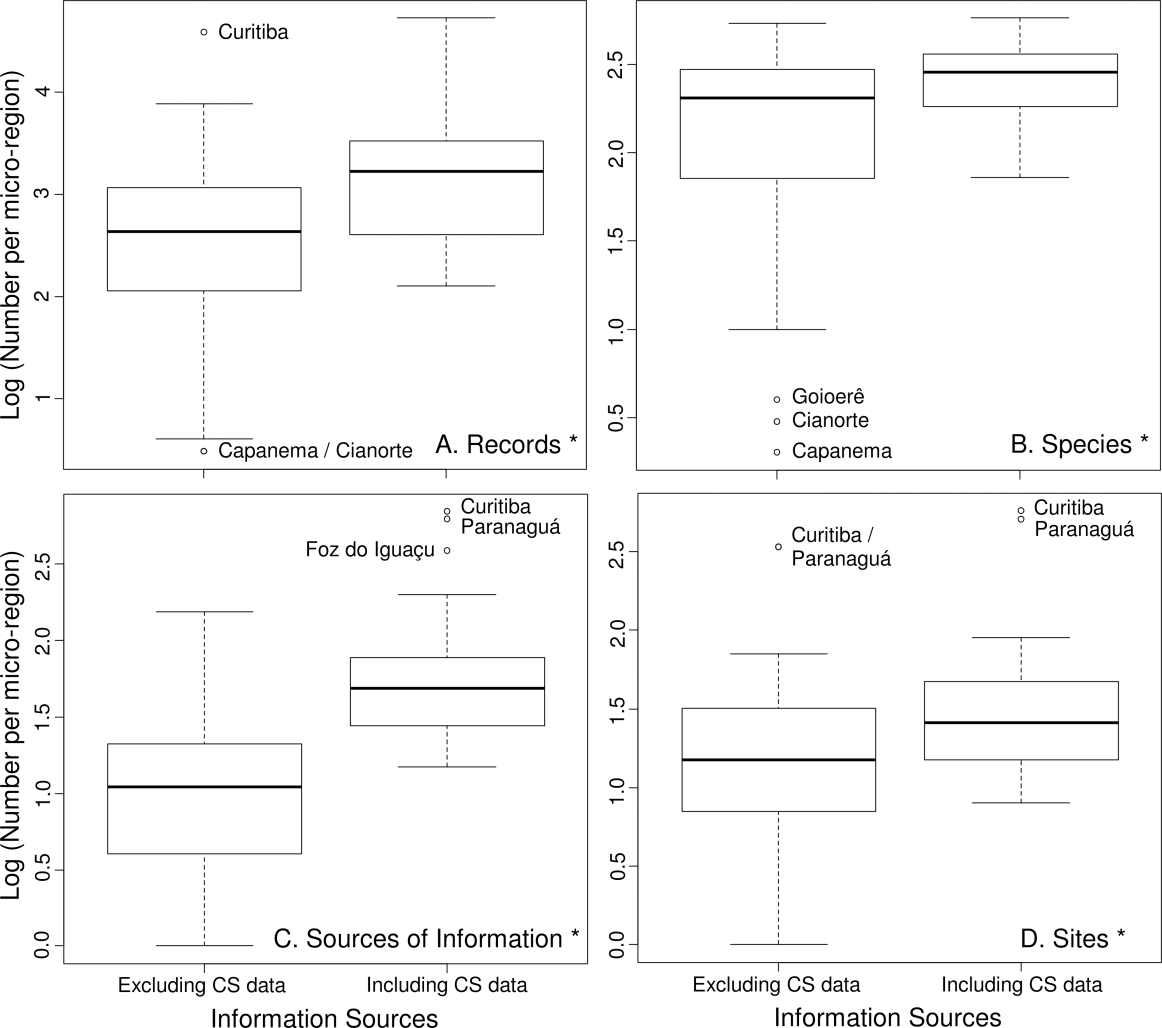
Comparisons between traditional scientific references data alone, and including CS data. Boxplots comparing: (A) number of records per microregion, (B) number of species per microregion, (C) number of sources of information per microregion, and (D) number of sites with records per microregion, both excluding and including CS data. For ease of representation, due to the large range of values, logarithmic scale was used. * statistically significant differences. Open circles represent extreme values found for microregions.

All variables under analysis showed significant differences when including CS data (Fig 6): number of records (W: 780, z: 5.443, *p*<0.05), number of species (W: 780, z: 5.443, *p*<0.05), number of sources of information (W: 780, z: 5.443, *p*<0.05) and number of sites (W: 780, z: 5.447, *p*<0.05).

## Discussion

### Species list

The growing number of birdwatchers and wildlife photographers has produced a considerable increase in the volume of information about Paraná’s avifauna (79,468 records for 675 species) in a short period of time (1986, first year for which CS data is available in Wiki Aves, to 2015, with 95% of the records ranging from 2010 to 2015), which quantitatively exceeds the last 195 years of formal ornithological research (70,346 records for 747 species). This demonstrates the great potential and importance of CS data for obtaining documented faunal records and making them freely available (e.g., through digital databases), especially for charismatic and easily observed groups, such as birds. On the other hand, considering that CS has been developed only recently, consulting traditional scientific references is still important, as they provide historical reference, and help understand trends and plan future conservation actions.

The effect of including CS data in qualitative and quantitative analyses, reported as positive by different authors (e.g., [18], [19] and [23]), is noticeable in almost all aspects evaluated here (see Figs 3 and 6), fills large gaps of information and brings important contributions to the data available from other sources. This kind of data can be used to track changes in populations, communities and ecosystems (see [65], [66], [67] and [68]), and allows understanding of ecological patterns at larger scales (e.g., changes in species distributions, migration patterns, effects of climate change and landscape modification, etc.) [23].

When we considered traditional scientific references data jointly with CS data, we found an increase in the number of bird species recorded in Paraná with respect to the latest list published [17], from 743 (cited as 744 in the original text) to 766. Of the 23 species included on the basis of this analysis, documentation for 14 came exclusively from CS data. Six other species were moved from the tertiary list to the primary list, and 22 were moved from the secondary list to the primary list based on image or voice records available in the Wiki Aves database.

The unreliable records—those of species geographically displaced in relation to habitat and the known distribution (S5 Table)—point to identification problems (e.g., *Trogon viridis* in FES [69], [70], [71], [72], misidentification of *Trogon rufus* [73]). The verification and correction of records, as pointed out previously [73], is crucial, as it would help to avoid replication of erroneous or dubious records and their misuse in ecological studies, resulting in erroneous or deceptive conclusions and recommendations [74]. Thus, we recommend that records contained in S5 Table are not to be replicated or used until new documented records are obtained (i.e., collected specimens, photographic images and/or voice recordings).

Species lists in both traditional scientific references data and CS data sources that are without proper documentation may contain misidentifications. Further, considering the large amount of data available in CS databases collected by non-experts, verification and validation of these records are important to detect misidentifications. In this sense, databases with available documentation for all records (e.g., Wiki Aves) can contribute to this detection. On the other hand, in databases where records can be contributed without any accompanying documentation (e.g., eBird, Táxeus) verification is hindered, rendering a higher chance of misidentifications going unrecognized. Even with the use of some mechanisms for validating records, such as verification of records by local specialists used in eBird, the lack of documentation of all records makes the proliferation of mistakes a more probable occurrence (for instance, misidentifications of species in a given region may go unnoticed and/or new records in an area may be marked as dubious).

The number of species with records in each vegetation type ranged from 506 (BM+CS) to 569 (BM+CS), except for SA, which reached 277 species (BM+CS). Despite little change in the total number of species, species composition varied significantly among EGL, FES, FOM and FOD. This result indicates a pattern of notable variation in the composition of bird communities which could be due to differences in the structure and composition of the vegetation, as pointed out by [75] and [76] and observed in several ecological studies (e.g., [77], [78], [79], [80], [81], [82]. The reduced number of species with records in SA may be associated with factors such as the small representation of this discontinuously distributed vegetation in the state [39], [51], coupled with the small number of studies carried out in this area compared to the effort expended in other vegetation types (S2 Table). This effect is also reflected in our rarefaction curve, which did not show a tendency towards stabilization of observed richness.

The reduced number of indicator and exclusive species in EGL (n = 3 and n = 1, respectively) and FOM (n = 4 and n = 0) suggests that these vegetation types house, almost exclusively, widely distributed species that are common to other vegetation types. However, the association between EGL and FOM is represented by 41 indicator and 5 exclusive species, thus indicating a bird community typical of these two vegetation types when taken together. This pattern is confirmed by the similarity overlap between the EGL and FOM points in Fig 2. Conversely, FES and FOD individually exhibit large numbers of indicator (n = 80 and n = 103, respectively) and exclusive species (n = 37 and n = 44), which indicates that each one is home to specific bird communities.

This result supports the traditional areas of bird endemism established by Cracraft [83], named *Paraná Center* (covering EGL and FOM) and *Serra do Mar Center* (FOD). Cracraft [83] also pointed that some species listed as endemic to *Paraná Center* are not restricted to this area, and the inclusion of *Serra do Mar Center* along with *Paraná Center* would comprise a well-defined area of endemism. However, the combination of these vegetation types (EGL, FOM and FOD), in our analysis, holds only 24 indicator and 3 exclusive species, fewer than FOD alone (n = 103 indicator and 44 exclusive) and EGL+ FOM together (n = 41 and 5). Also, a third vegetation type (FES), not recognized by Cracraft [83] as an area of bird endemism, showed a large number of indicator (n = 80) and exclusive species (n = 37) in this study.

Another bird endemism area, identified by Silva et al. [74] and named *Serra do Mar*, extends from the far east to the north-central region of Paraná state and covers FOD and part of EGL, FOM and SA (based on maps available in Silva et al. [74]). Together, these four vegetation types have only four indicator and no exclusive species, suggesting that the bird communities in these areas are not, in their majority, exclusive of this geographic sector. The two endemic species used by Silva et al. [74] to define this area, *Orthogonys chloricterus* and *Dacnis nigripes*, occur in regions that do not cover the north-central area of the state, and the species occurrences in these sectors are based on misidentifications published in [69] (see S5 Table). The differences found between bird endemism areas that cover the state of Paraná, proposed by other authors [74], [83], and the number of indicator and exclusive species obtained in our analysis, highlight the effect that variations in the databases (e.g., lack of records, misidentifications) can have on the detection and delimitation of these areas.

Considering the number of indicator and exclusive species for each vegetation type or their combinations, three areas with peculiar bird communities can be identified in Paraná, partly differing from those proposed by Silva et al. [74] and Cracraft [83], which cover the areas occupied by: 1—FES; 2—FOD; and 3—EGL + FOM. This division corresponds, within Paraná, to the ecoregions proposed by Olson et al. [84]—*High Paraná Atlantic forests* (FES), *Serra do Mar coastal forests* (FOD) and *Araucaria moist forests* (EGL + FOM). Also, they are compatible with biogeographical areas proposed for other non-vertebrate taxa [85], i.e., three provinces within the Paraná sub-region—*Paraná Forest* (FES), *Brazilian Atlantic Forest* (FOD) and *Araucaria angustifolia Forest* (EGL + FOM). These vegetation types are included within *Brazil’s Atlantic Forest* biodiversity hotspot [86], [87].

Associations between FES and FOD and between FES and FOM presented the second and third highest number of indicator (23 and 12, respectively) and exclusive species (3 and 2, respectively) among the pairs of vegetation types. Geographical proximity and the presence of large ecotone areas between FES and FOM [39], [51] might explain this association. However, the discontinuity between FES and FOD in Paraná [39], [51] suggests that the occurrence of indicator and exclusive species for these two vegetation types is related to other factors–both ecological and historical (e.g., dispersal history of vegetation types in southern Brazil, see [88], [89]). Given that processes in ecological systems are highly dependent on spatial scale [90], [91], [92], future studies using different sample unit sizes and/or different subdivisions of vegetation types should be conducted to evaluate how the spatial scale of analysis affects our understanding of the relationship between species and environments.

The percentage of extinct species in each vegetation type, including CS data, ranged from 0% in SA to 3.6% in FES. The absence of extinction events in SA can be associated with the small representation of this vegetation type [39], [51] and with the small number of studies conducted in this area, especially before 1990 (see S2 Table). Considering habitat loss to be a major cause of rising global extinction rates [93], [94], [95], [96], the highest percentage of extinct species in FES can be associated with accentuated suppression and fragmentation of this forest compared with other vegetation types [51], [97]. The areas occupied by FES are characterized by flat reliefs and fertile soil [51], which contributed to the almost total conversion of this forest into agricultural lands.

Similarly, the highest percentage of extinct species from 1960 to 1989 can be associated with accentuated suppression and fragmentation of the forests during this time period, compared with 1990–2015 [51], [52], [97]. In 1960–1989 from 65% to 85% of the forest had been suppressed [51], [52], and in 1990–2015 from 85% to 88% of the forest had been suppressed [53], [97]. Therefore, the time period from 1960 to 1989 is characterized by reaching and surpassing the percentage of forest cover at nearly the extinction threshold (i.e., around 30% of natural vegetation cover [54]), and a higher number of extinctions is expected in the 1960–1989 time period. Rates of local extinction increase as forest cover is reduced, when dispersal between fragments become highly limited by distance among remnants [54]. Studies with different taxonomic groups and in different parts of the world indicate that this is especially important in landscapes with less than 30% of their natural cover [98], [99], [100], [101], wherein the average distance between the fragments increases exponentially with habitat loss [54].

The current conservation status of the bird communities in Paraná - 69 threatened species [102]—added to the high level of threat and a large number of species and rate of endemism in the Atlantic Forest [86], reinforce that the vegetation types analyzed in the study area are some of the most threatened in the world [86], [87], [103].According to the International Union for Conservation of Nature (IUCN) [104], among the 25 species considered regionally extinct (BM+CS), four are categorized as threatened and 21 as least concern species at the time of this study. Also, according to local threatened species lists [102], nine are categorized as threatened and 16 as non-threatened species (i.e., near threatened, data deficient or not evaluated). The fact that the vast majority of the species are non-threatened suggests that the effects of landscape modifications are so pronounced that not only rare and admittedly threatened species are becoming locally or regionally extinct, but all species are being affected to some extent. Furthermore, considering the extent of these modifications, it can be expected that other non-bird taxa are also affected.

Among those species considered regionally extinct, a caveat should be made concerning migratory species and those whose geographical distribution only marginally encompass the state within the indicated vegetation types (S8 Table). The absence of recent records for these species may be associated with the irregular specimen occurrence (i.e., with a small chance of detection), and not due to local or regional extinction events. The inclusion of CS data altered the number of species considered regionally extinct (48 BM to 25 BM+CS) and extinct in the state (12 BM to 10 BM+CS), demonstrating a failure of traditional scientific references data alone to truly represent species’ occurrences in a timely manner, and reinforcing the importance of CS for accurate bird assessments in the state.

Of the 14 species classified as recently-colonizing, 13 had records in FES (*Aratinga nenday*, *Buteo nitidus*, *Campylorhynchus turdinus*, *Fluvicola nengeta*, *Gampsonyx swainsonii*, *Icterus croconotus*, *Myiopsitta monachus*, *Myiozetetes cayanensis*, *Paroaria dominicana*, *Rhea americana*, *Saltator coerulescens*, *Schistochlamys melanopis* and *Tyrannus albogularis*), five in FOM (*Fluvicola nengeta*, *Gampsonyx swainsonii*, *Myiopsitta monachus*, *Theristicus caerulescens* and *Tyrannus albogularis*), two in SA (*Schistochlamys melanopis* and *Gampsonyx swainsonii*), and one in EGL (*Myiopsitta monachus*) and FOD (*Fluvicola nengeta*). Whereas the original distributions of these species cover regions to the north and/or west of the state, and in the case of *Myiopsitta monachus*, *Rhea americana*, *Theristicus caerulescens* and *Saltator coerulescens* also to the south of Paraná [63], we identified a colonization trend, which has been occurring in the north-south direction, and more so in the northwest portion of the state. These species are commonly associated with open habitats (grasslands, savannas and wetlands) or areas with vegetation in the early stages of development and are found in the western, northwestern and northern Paraná, where original forest cover (FES) has been replaced by pastures and plantations. Such environments offer conditions that allow the establishment of new populations, increasing the species’ distribution area, especially for species that are able to exploit anthropogenic environments [105], [106], [107]. Still, recent expansions may also be associated with climate processes such as global warming [108], which allows lower latitudes and warmer climate taxa to expand their distributions to higher latitudes, previously inaccessible due to originally cooler weather [109], [110], [111], [112]. This pattern may corroborate the fact that observed colonization has occurred in the north-south direction, even though open habitats and species associated with them are also present in neighboring regions south of Paraná [84].

We expect that other bird species associated with open habitats, and also occurring north and northwest of Paraná, may colonize the state in the future. These colonization events should follow an expansion path from the boundaries of these species’ distribution, as a response to climate (i.e., global warming, see [108], [113], [114]), and habitat change (i.e., FES in Paraná) mediated by human action ("*Leading-edge dispersal*", [115]). Species with large distributions, high local abundance, with small body sizes, high fertility, high genetic variability, rapid dispersion rates and presenting commensalism with humans [116], [105], fall into this category and should be given special attention in future studies. This colonization process can contribute to the population reduction of native species populations by predation, competition, disease transmission and other problems related to biological invasions, aggravating the conservation status of the local avifauna and especially of endangered and endemic species [117], [118], [119], [120].

Some of the species considered as presenting local population expansion do not allow a direct interpretation of population growth based on the increase in record numbers. This is particularly true for species of conservation interest, as it could be just reflecting species oversampling (i.e., increased effort to record certain species, greater than the average effort for a given vegetation type). Threatened species (i.e., *Aburria jacutinga*, *Carpornis melanocephala*, *Ramphastos vitellinus*, and *Sporophila frontalis*) and uncommon species in the state (e.g., *Falco peregrinus*, *Ramphocaenus melanurus*, *Spizaetus tyrannus*) are examples of apparent local population expansion probably related to oversampling. Among birds classified as native invasive species there are many that thrive in anthropic areas (e.g., *Coragyps atratus*, *Patagioenas picazuro*, *Rupornis magnirostris*, *Sicalis flaveola*, *Tyrannus melancholicus*, *Vanellus chilensis*, *Zenaida auriculata*, *Zonotrichia capensis*; see [121]), including pastures, plantations and urban zones [25], [26]. In addition, four species known to tolerate some degree of disturbance, but not primarily associated to altered areas, were also listed as native invasive species: *Emberizoides herbicola*, *Nyctibius griseus*, *Pachyramphus validus* and *Xenops rutilans*.

The largest number of native invasive species in FES (45 species; BM+CS), followed by FOM (34, BM+CS) and EGL (30, BM+CS), may be related to the replacement of many of these formations by anthropogenic environments (pastures and crops) favoring their occupation by generalist birds [107], [121]. On the other hand, the lowest number of native invasive species in FOD (18 species; BM+CS) may reflect the higher degree of conservation of this vegetation type within the state [97].

The lists of regionally extinct, native and non-native invasive species is an important first step in understanding the processes leading to bird community changes [29]. A more detailed examination of the effects of habitat loss on birds is necessary to characterize the changes in species composition over time. Another aspect yet to be evaluated is the gradual replacement of regionally distinct communities (composed of many endemic species) by cosmopolitan communities, and how these changes may lead to increased similarity between vegetation types over time (i.e., biotic homogenization; [122], [105], [123]). The numbers of extinct, native and non-native invasive species observed in this study are indicative of this process, which is intensified with the reduction of natural environments mediated by human action. Habitat reduction can affect animal populations in many ways, especially reducing resource availability, such as food and nest-site sources [124], as well as modifying the behavior and distribution of territorial species. These changes can also favor the colonization of altered habitats by generalist birds that can cope and benefit from the new conditions imposed by anthropogenic action [125].

### Avifaunal knowledge level

Despite the fact that bird sampling efforts in the state were initiated over two centuries ago, the level of knowledge within microregions is quite heterogeneous. The lack of formal studies and, consequently, bird records in certain regions, when compared to other well-sampled areas, is evidence of this heterogeneity. The inclusion of CS data decreases this difference, reinforcing the importance of citizen scientists as sources of information, and pointing out that using multiple sources for species occurrence and distribution analyses is crucial [18].

First documentation for many species in the state have been obtained by citizens, as is the case for *Myiopsitta monachus* [126] and *Theristicus caerulescens* [127] in the micro region of Cascavel; *Myiozetetes cayanensis* in Capanema [128], *Contopus cooperi* in Prudentópolis [129] *Sporophila ruficollis* in Maringa [130], *Tyrannus albogularis* in Apucarana [131] and *Paroaria dominicana* in Jacarezinho [132], all available through Wiki Aves [27]. These examples demonstrate the importance of using CS data to characterize bird species composition of vast regions, especially in the Neotropics. Furthermore, this source of data can provide quicker biodiversity assessments, compared to data from traditional scientific references, and could be a particularly important tool when rapid decisions are needed (e.g., conservation actions).

The inclusion of CS data significantly alters the level of knowledge about birds, not only at the microregions level but throughout the state—from Low (BM) to High (BM+CS). The filling of information gaps in regions that had been under sampled by traditional research is one of the main contributions made by CS, which is especially important for studies that focus on species distribution and biogeography. The effect of using these data for large areas and with great biodiversity, as pointed out by [18], [19] and [23], was hereby confirmed. Nevertheless, even after the inclusion of this information, there are still microregions in Paraná with Low levels of knowledge. Bird species composition within these regions (Assaí, Cianorte, Faxinal, Floraí, Goioerê, Ibaiti, Pitanga and Wenceslau Braz) should be considered insufficiently known, deserving special attention of both researchers and citizens.

As a result of including CS, we can build a more realistic picture of the avifaunal composition in the state, compared to one that uses only data from traditional scientific references (technical literature and museum collections). This is evinced in the increased number of species in each microregion, in the whole state and in vegetation types individually found when including CS data. In addition, logistical and financial inability to carry out systematic sampling over large areas, especially in highly diverse regions such as the Neotropics, make the work of citizens, as demonstrated here, even more valuable.

## Conclusions

Our data indicated the presence of characteristic bird communities in three regions of Paraná state, covering the areas occupied by FES; FOD; and EGL + FOM. Among them, FES lost more species (local extinct species), and received more recently-colonizing and native invasive species compared to other vegetation types. This scenario reflects the intense forest loss that occurred in this region, resulting in a remarkable change of its birdlife. This change may be leading to a growing and irreversible process of biotic homogenization, increasing similarity with other typologies and reducing beta diversity. Still, the large number of indicator species in FES, and the presence of birds that are unique to it within the state, raises particular concern about the conservation of forest remnants of this vegetation type.

Paraná’s avifaunal knowledge significantly increased in the last decade and especially over the past five years, with many contributions of citizens (birdwatchers and nature photographers) documenting ornithological records. The production of such data and its unrestricted availability makes CS a very important and extremely useful tool that provides new information and contributes to close knowledge gaps about the occurrence and distribution of numerous species.

## Supporting information

S1 Table Natural history museums from which data was used to build the Paraná state bird database. (DOCX)

S2 Table Literature references used to build the Paraná state bird database. (DOCX)

S3 Table Primary and secondary (within square brackets) lists of species with records in Paraná state, indicating the number of records in each vegetation type (see also S1 Table and S1 Table). Notes: I–introduced non-native species with recently estabilished populations in the state; C–recently-colonizing species; N–species included in our primary and secondary lists but not mentioned in Scherer-Neto et al. [17]; N(S)–species included in our primary and secondary lists, and mentioned in the terciary list of Scherer-Neto et al. [17]; SP–species transferred from the secondary list of Scherer-Neto et al. [17] to our primary list. Environments and Vegetation types: MAR–Seabirds; EGL–Grassland; FES–Semideciduous Tropical Forest; FOD–Tropical Rainforest; FOM–Araucaria Moist Forest; SA–Savanna. Source: WA–Wiki Aves database. Nomenclature and taxonomic order follow CBRO [35].
(DOCX)

S4 Table Terciary list of bird species in the state of Paraná. Vegetation types of records (when available): **EGL**–Grassland; **FES**–Semideciduous Tropical Forest; **FOD**–Tropical Rainforest; **FOM**–Araucaria Moist Forest; **SA**–Savanna. Comments: “probably misidentification” and “probably scaped” are based on Klemann-Junior personal observations. Nomenclature and taxonomic order follow CBRO [35]. * Escaped from captivity, established population confirmation is required.
(DOCX)

S5 Table Individual occurrences that need further documentation in the vegetation types listed and were thus excluded from our database due to being displaced from their known geographic distribution within the Paraná state. Vegetation types: **EGL**–Grassland; **FES**–Semideciduous Tropical Forest; **FOD**–Tropical Rainforest; **FOM**–Araucaria Moist Forest; **SA**–Savanna. **Actual taxon:** misidentifications were corrected following author’s knowledge and expertise in Paraná avifauna, and/or when misidentifications were obvious given the habitat preferences among congeners. **Comments**: distributions based on [27], [61].
(DOCX)

S6 Table Years with bird records in each vegetation type in Paraná state considering only data from traditional scientific references (BM), and including CS data (BM+CS). Vegetation types: **EGL**–Grassland; **FES**–Semideciduous Tropical Forest; **FOD**–Tropical Rainforest; **FOM**–Araucaria Moist Forest; **SA**–Savanna.
(DOCX)

S7 Table Indicator species and their Indicator Values (IndVal) in each vegetation type in Paraná state followed by specificity, fidelity and *p*-values considering only data from traditional scientific references (BM), and including CS data (BM+CS). Vegetation type: **EGL**–Grassland; **FES**–Semideciduous Tropical Forest; **FOD**–Tropical Rainforest; **FOM**–Araucaria Moist Forest; **SA**–Savanna. The total number of species in each vegetation type is presented within brackets.
(DOCX)

S8 Table Extinct species in each vegetation type in Paraná state considerding only data from traditional scientific references (BM) and including CS data (BM+CS). *Species extinct in the state of Paraná. Excluded species: ^1^ Species of marginal occurrence in shown vegetation type. ^2^ Migrants, partial migrants and species that perform seasonal dispersal, nomadic or other with poorly known mobility [61]. ^3^ Seabirds. Vegetation type: **EGL**–Grassland; **FES**–Semideciduous Tropical Forest; **FOD**–Tropical Rainforest; **FOM**–Araucaria Moist Forest. Species are organized in alphabetical order. Global and of Paraná state (PR) threat categories of the species, according to IUCN [96] and Straube et al. [94]: **LC**–Least Concern; **NT**–Near Threatened; **VU**–Vulnerable; **EN**–Endangered; **CR**–Critically Endangered; **RE**–Regionally Extinct; **DD**–Data Deficient; - –Not Evaluated.
(DOCX)

S9 Table Species that show local population increases and native invasive species (regional population and geographic distribution increases) in each vegetation type in Paraná state considering only data from traditional scientific references (BM), and including CS data (BM+CS). Vegetation type (Veg. type): **EGL**–Grassland; **FES**–Semideciduous Tropical Forest; **FOD**–Tropical Rainforest; **FOM**–Araucaria Moist Forest.
(DOCX)

S10 Table Level of avifaunal knowledge in Paraná state. Number of records, species, sources of information and sites per microregions in Paraná considering only data from traditional scientific references (BM), and including CS data (BM+CS). Microregions are presented in alphabetical order.
(DOCX)
