# Supplementary material for: Traditional scientific data vs. uncoordinated citizen science effort: A review of the current status and comparison of data on avifauna in Southern Brazil
Source: PLoS One. 2017 Dec 11;12(12):e0188819. doi: 10.1371/journal.pone.0188819 (PMC5724844; doi:10.1371/journal.pone.0188819)
Supplement: S1 Table — (DOCX) [file pone.0188819.s001.docx]

1. **S1 Table.** Natural history museums from which data was used to build the Paraná state bird database.

| **Acronym** | **Museum** | **Consultation date** |
| --- | --- | --- |
| AMNH | American Museum of Natural History | 2015 Aug 31 |
| FMNH | The Field Museum of Chicago | 2015 Aug 31 |
| MCN-FZB | Museu de Ciências Naturais da Fundação Zoobotânica | 2014 Oct 13 |
| MCP | Museu de Ciências e Tecnologia da Pontifícia Universidade Católica do Rio Grande do Sul | 2014 Oct 24 |
| MHNCI | Museu de História Natural Capão da Imbuia | 2014 Jun 09 |
| MZUEL | Museu de Zoologia da Universidade Estadual de Londrina | 2015 Aug 31 |
| MZUSP | Museu de Zoologia da Universidade de São Paulo | 2012 Jul 08 |
| NMNH | Smithsonian Museum | 2015 Aug 31 |
