# Supplementary material for: Traditional scientific data vs. uncoordinated citizen science effort: A review of the current status and comparison of data on avifauna in Southern Brazil
Source: PLoS One. 2017 Dec 11;12(12):e0188819. doi: 10.1371/journal.pone.0188819 (PMC5724844; doi:10.1371/journal.pone.0188819)
Supplement: S2 Table — (DOCX) [file pone.0188819.s002.docx]

**S2 Table.** Literature references used to build the Paraná state bird database.

| **Nº** | **References** |
| --- | --- |
| 1 | Abe LM. Registro de *Psarocolius decumanus* Pallas, 1969, no Parque Estadual de Campinhos, Tunas do Paraná, PR (Aves: Passeriformes, Icteridae). Atualidades Orn. 1996;74:12. |
| 2 | Abe LM. Registro de "Saí-canário", *Thlypopsis sordida* d’Orbigny & Lafresnaye, 1837, na região sul do Estado do Paraná (Aves: Passeriformes: Emberizidae). Atualidades Orn. 1998;81:4. |
| 3 | Aguilar YH, Figueiredo C, Lopes ME. Estudos preliminares da biologia e estimativa populacional do *Phalacrocorax olivaceus* na Ilha do Biguá, Baía de Antonina, PR. Anais do XV Congresso Brasileiro de Zoologia; 1988; Curitiba, BR. Sociedade Brasileira de Zoologia; 1988. p 495. |
| 4 | Almeida A, Couto HTZ, Almeida ÁF. Camouflaging of seeds treated with pesticides mitigates the mortality of wild birds in wheat and rice crops. Sci agric. 2010*;*67(2):176-182. |
| 5 | Amorin RR, Scherer-Neto P, Rosa CR, Abilhoa V. Avifauna do Parque Estadual do Pico Paraná e áreas adjacentes, sul do Brasil. Natureza on line. 2012;10(3):118-125. |
| 6 | Andrade L, Cândido-Junior J F, Lui RL, Gorski F. Levantamento de corujas atropeladas na BR 277, trecho entre Cascavel e Foz do Iguaçu – PR. Anais do XI Congresso Brasileiro de Ornitologia; 2003 Nov 23-28; Feira de Santana, BR. Sociedade Brasileira de Ornitologia; 2003. p 73. |
| 7 | Anjos L. Aspectos etológicos do *Myiophobus fasciatus* (Aves, Tyrannidae) no Estado do Paraná, Brasil. Arq Biol Tecnol. 1984;27(3):401-405. |
| 8 | Anjos L. Aves do Capão da Imbuia. Curitiba, Paraná. Anais do XIII Congresso Brasileiro de Zoologia; 1986; Cuiabá, BR. Sociedade Brasileira de Zoologia; 1986. p 201. |
| 9 | Anjos L. Nota sobre um ninho em atividade de *Cyanocorax caeruleus* (Aves.Corvidae), na região de Palmeira. Estado do Paraná. Anais do XIV Congresso Brasileiro de Zoologia; 1987 Feb 01-06; Juiz de Fora, BR. Sociedade Brasileira de Zoologia; 1987. p 150. |
| 10 | Anjos L. Distribuição de aves em uma floresta de araucária da cidade de Curitiba (Sul do Brasil). Acta Biol Parana. 1990;19(1,2,3,4):51-63. |
| 11 | Anjos L. O ciclo anual de *Cyanocorax caeruleus* em floresta de araucária (Passeriformes: Corvidae). Ararajuba. 1991;2:19-23. |
| 12 | Anjos L. Análise preliminar das manifestações sonoras e do habitat de *Picumnus nebulosus*Sundevall (Aves, Picidae). Rev Bras Zool. 1999;16(2):433-439 |
| 13 | Anjos L. Bird communities in five Atlantic forest fragments in Southern Brazil. Ornitol Neotrop. 2001;12(1):11-27. |
| 14 | Anjos L. Forest bird communities in the Tibagi River Hydrographic Basin, Southern Brazil. Ecotropica. 2002;8:67‑79. |
| 15 | Anjos L. A eficiência do método de amostragem por pontos de escuta na avaliação da riqueza de aves. Rev Bras Ornitol. 2007;15(2):239‑243. |
| 16 | Anjos L, Bóçon R. Primeiros registros de *Biatas nigropectus* no estado do Paraná. Anais do II Congresso Brasileiro de Ornitologia; 1992. Campo Grande, BR. Sociedade Brasileira de Ornitologia; 1992. Resumo 53. |
| 17 | Anjos L, Boçon R. Bird Communities in Natural Forest Patches in Southern Brazil. Wilson Bull. 1999;111(3):397-414. |
| 18 | Anjos L, Ferreira ARJ. Registros de campo de *Hylocharis sapphirina* e *H. cyanus* na região de Londrina, norte do Estado do Paraná, sul do Brasil (Trochiliformes: Trochilidae). Ararajuba. 1998;6(1):51. |
| 19 | Anjos L, Graf V. Riqueza de aves da Fazenda Santa Rita, região dos Campos Gerais, Palmeira, Paraná, Brasil. Rev Bras Zool. 1993;10(4):673-698. |
| 20 | Anjos L, Laroca S. Abundância relativa e diversidade específica em duas comunidades urbanas de aves de Curitiba (sul do Brasil). Arq Biol Tecnol. 1989;32(4):637-643. |
| 21 | Anjos L, Schuchmann KL. Biogeographical affinities of the avifauna of the Tibagi river basin, Paraná drainage system, Southern Brazil. Ecotropica. 1997;3:43-65. |
| 22 | Anjos L, Schuchmann KL, Berndt R. Avifaunal composition, species richness, and status in the Tibagi river basin, Parana State, Southern Brazil. Ornitol Neotrop.1997;8(2):145-173. |
| 23 | Anjos L, Seger C. Análise da distribuição das aves em um trecho do rio Paraná, divisa entre os estados do Paraná e Mato Grosso do Sul. Arq Biol Tecnol. 1988;31(4):603-612. |
| 24 | Arakaki BR, Bochio G, Anjos L. 2013. Espécies que vivem em ambientes ripários de floresta contínua são menos sensíveis à fragmentação? Anais do XX Congresso Brasileiro de Ornitologia; 2013 Nov 04-07; Passo Fundo, BR. Sociedade Brasileira de Ornitologia; 2013. p 358-359. |
| 25 | Arakak, BR, Souza AR, Silva CAP, Jebai GT, Anjos L. Algumas espécies de aves como potenciais dispersores de sementes e agentes aceleradores da recomposição vegetal nativa. Anais do XVII Congresso Brasileiro de Ornitologia; 2009 Jun 28-Jul 03; Aracruz, BR. Sociedade Brasileira de Ornitologia; 2009. Resumos (Ecologia), EC128, p 43. |
| 26 | Arruda SD, Lara AI. Viuvinha-de-óculos *Hymenops perspicillata* (Tyrannidae) no estado do Paraná, Brasil. Anais do II Congresso Brasileiro de Ornitologia; 1992. Campo Grande, BR. Sociedade Brasileira de Ornitologia; 1992. Resumos R63. |
| 27 | Arruda SD, Luçolli SC. 1991. Ocorrência de aves ameaçadas de extinção em General Carneiro, centro-sul do Estado do Paraná. Anais do I Congresso Brasileiro de Ornitologia; 1991 Jul 21-27. Belém, BR. Sociedade Brasileira de Ornitologia; 1991. p 38. |
| 28 | Arzua M. Ectoparasitos de aves silvestres. Atualidades Orn. 1998;82:4. |
| 29 | Arzua M. Diversidade de carrapatos (Acari - Ixodidae) de remanescentes de Floresta Estacional Semidecidual e de Floresta Ombrófila Densa, no estado do Paraná [thesis]. Programa de Graduação em Ciências Biológicas – Entomologia: Universidade Federal do Paraná; 2007. |
| 30 | Arzua M, Barros DM, Linardi PM, Botelho JR. Noteworthy records of *Ixodes auritulus* Neumann, 1904 (Acari, Ixodida) on birds of Paraná, southern Brazil. Mem I Oswaldo Cruz. 1994;89(1):129. |
| 31 | Arzua M, Mitroszewski A. Avifauna do Bosque Reinhard Maack e a importância deste remanescente florestal urbano para a conservação de aves em Curitiba, Paraná. In: Straube FC, editor. Ornitologia sem fronteiras, incluindo os Resumos do IX Congresso Brasileiro de Ornitologia; 2001 Jul 22-27; Curitiba, BR. Fundação O Boticário de Proteção à Natureza; 2001. R10, p 15. |
| 32 | Arzua M, Scherer-Neto P, Cardoso DS, Mitroszewski A, Czelusniaki SM. Novo registro de *Thlypopsis sordida* no Paraná: um provável caso de expansão regional de distribuição. In: Straube FC, editor. Ornitologia sem fronteiras, incluindo os Resumos do IX Congresso Brasileiro de Ornitologia; 2001 Jul 22-27; Curitiba, BR. Fundação O Boticário de Proteção à Natureza; 2001. R11, p 16. |
| 33 | Arzua M, Scherer-Neto P, Figueiredo GJ, Carrano E, Ribas CF, Rachwald M. Novos registros de saí-canário *Thlypopsis sordida* (Emberizidae) no Estado do Paraná, com comentários sobre sua distribuição. Ararajuba. 2001;9(2):143-144 |
| 34 | Assis CV, Goulart E, Anjos L. Repertório vocal da gralha-picaça, *Cyanocorax chrysops* (Vieillot, 1818) (Passeriformes: Corvidae) de uma reserva urbana: Parque do Ingá, Maringá – Paraná – Brasil. Anais do XI Congresso Brasileiro de Ornitologia; 2003 Nov 23-28; Feira de Santana, BR. Sociedade Brasileira de Ornitologia; 2003. p 36. |
| 35 | Aurélio-Silva M. Comportamento de *Dryocopus lineatus* (Picidae) durante confecção de cavidade. Anais do Anais do XIII Congresso Brasileiro de Ornitologia; 2005 Out 30-Nov 04; Belém, BR. Sociedade Brasileira de Ornitologia; 2005. p 75. |
| 36 | Baijuk S, Jesus S. Registros de nidificação e reutilização de ninho do besourinho-de-bico-vermelho *Chlorostilbon lucidus* (Apodiformes: Trochilidae). Atualidades Orn. 2010;154:4-5. |
| 37 | Barbosa-da-Silva JC, Cândido-Junior JF, Escolari LF, Santos LL. Uso de ninhos artificiais de bambu por aves em Santa Helena-PR, Brasil. Anais do XVII Congresso Brasileiro de Ornitologia; 2009 Jun 28-Jul 03; Aracruz, BR. Sociedade Brasileira de Ornitologia; 2009. Resumos (Conservação), CS89, p. 31. |
| 38 | Barnett JM, Minns J, Kirwan GM, Remold H. Informações adicionais sobre as aves dos estados do Paraná, Santa Catarina e Rio Grande do Sul. Ararajuba. 2004;12(1):55-58 |
| 39 | Barros-Battesti DM, Arzua M, Pichorim M, Keirans JE. *Ixodes* (*Multidentatus*) *paranaensis* n. sp. (Acari: Ixodidae) a parasite of *Streptoprocne biscutata* (Sclater 1865) (Apodiformes: Apodidae) birds in Brazil. Mem I Oswaldo Cruz. 2003;98(1):93-102 |
| 40 | Bazilio S, Belin AAR, Candido-Junior JF. Cuiú-cuiús (*Pionopsitta pileata* Scopoli, 1769) alimentando-se de feloderme de *Eucalyptus urophylla* (Myrtaceae) na Araupel S/A, Quedas do Iguaçu – Região Sudoeste do Estado do Paraná. Anais do XI Congresso Brasileiro de Ornitologia; 2003 Nov 23-28; Feira de Santana, BR. Sociedade Brasileira de Ornitologia; 2003. p 38. |
| 41 | Belin AAR, Bazilio S, Cândido-Junior JF. Avifauna na Reserva Particular do Patrimônio Natural (RPPN) Corredor do Iguaçu da empresa Araupel S/A, região sudoeste do Estado do Paraná. Anais do XI Congresso Brasileiro de Ornitologia; 2003 Nov 23-28; Feira de Santana, BR. Sociedade Brasileira de Ornitologia; 2003. p 75. |
| 42 | Belin AAR, Bazilio S, Lima PCG. Avifauna do Parque Municipal das Araucárias (Guarapuava, PR), com abordagem na fragmentação da floresta ombrófila mista. Anais do XI Congresso Brasileiro de Ornitologia; 2003 Nov 23-28; Feira de Santana, BR. Sociedade Brasileira de Ornitologia; 2003. p 76. |
| 43 | Bencke GA, Dias RA, Fontana AS. Observações ornitológicas relevantes no Parque Nacional do Iguaçu e arredores, incluindo o primeiro registro de *Campylorhynchus turdinus* para o Paraná. Atualidades Orn. 2008;145:6-7. |
| 44 | Bichinski TAT. Comportamento reprodutivo de *Sporophila hypoxantha* (Passeriformes: Emberizidae) no estado do Paraná. Atualidades Orn. 2011;163:57-61. |
| 45 | Bispo AA. Dinâmica da avifauna de um remanescente florestal de pequeno porte no município de Tijucas do Sul, Paraná, Brasil. In: Straube FC, editor. Ornitologia sem fronteiras, incluindo os Resumos do IX Congresso Brasileiro de Ornitologia; 2001 Jul 22-27; Curitiba, BR. Fundação O Boticário de Proteção à Natureza; 2001. R18, p 23. |
| 46 | Bispo AA, Scherer-Neto P. Taxocenose de aves em um remanescente da Floresta com Araucária no sudeste do Paraná, Brasil. Biota Neotropica. 2010;10(1):121-130 |
| 47 | Boçon R. Riqueza e abundância de aves em três estágios sucessionais da Floresta Ombrófila Densa Submontana, Antonina, Paraná [thesis]. Programa de Pós Graduação em Ciências Biológicas – Zoologia: Universidade Federal do Paraná; 2010. |
| 48 | Boçon R, Anjos L, Seraffini PP, Santos LGC, Cavalli P. Diversidade e abundância da avifauna da Reserva Natural do Cachoeira, floresta atlântica, litoral do Paraná. Anais do XII Congresso Brasileiro de Ornitologia; 2004 Nov 21-26; Blumenau, BR. Sociedade Brasileira de Ornitologia; 2004. p 170. |
| 49 | Bóçon R, Lara AI, Seger C, Scherer-Neto P. Registros de quatro espécies de aves pouco comuns para o estado do Paraná. Anais do II Congresso Brasileiro de Ornitologia; 1992. Campo Grande, BR. Sociedade Brasileira de Ornitologia; 1992. Resumos R56. |
| 50 | Bóçon R, Sipinski EAB.Deslocamentos de *Amazona brasiliensis* em sua área de distribuição no estado do Paraná. Anais do XV Congresso Brasileiro de Ornitologia, Brasil; 2007 Jul 1-6; Porto Alegre, BR. Sociedade Brasileira de Ornitologia; 2007. PAINEL DM05, p 145-146. |
| 51 | Bóçon R, Sipinski EAB, Rivera R, Kawai A. A utilização de ninhos artificiais por *Amazona brasiliensis* em sítios reprodutivos na região da Ilha Rasa, Guaraqueçaba – Paraná. Anais do XII Congresso Brasileiro de Ornitologia; 2004 Nov 21-26; Blumenau, BR. Sociedade Brasileira de Ornitologia; 2004. p 171. |
| 52 | Boesing AL, Corrêa L, Woldan DRH, Bazílio S. Registros de aves raras na região sudoeste do Paraná, Brasil Anais do XV Congresso Brasileiro de Ornitologia, Brasil; 2007 Jul 1-6; Porto Alegre, BR. Sociedade Brasileira de Ornitologia; 2007. PAINEL CS07, p 205-206. |
| 53 | Bomfim NAB, Costa LCM. Descrição e frequência dos padrões motores relacionados às atividades de manutenção e locomoção de *Gallinula chloropus* (Gruiformes, Rallidae) em Curitiba, Paraná In: Straube FC, editor. Ornitologia sem fronteiras, incluindo os Resumos do IX Congresso Brasileiro de Ornitologia; 2001 Jul 22-27; Curitiba, BR. Fundação O Boticário de Proteção à Natureza; 2001. R19, p 24. |
| 54 | Bornia PCDAA. Estudo Comparativo da Avifauna do Bosque “Capão da Imbuía” Curitiba – PR – Brasil [monograph]. Graduação em Biologia – Setor de Ciências Biológicas e da Saúde: Pontifícia Universidade Católica do Paraná; 1999. |
| 55 | Bornschein MR. Formações Pioneiras do Litoral Centro-Sul do Paraná: Identificação, Quantificação de Áreas de Caracterização Ornitofaunística [dissertation]. Programa de Pós- graduação em Engenharia Florestal: Universidade Federal do Paraná. Universidade Federal do Paraná; 2001. |
| 56 | Bornschein MR. Relatório de avifauna. In: Mater Natura, editor. Diagnósticos para o Plano de Manejo do Parque Nacional de Ilha Grande. Curitiba, Paraná. 2003. |
| 57 | Bornschein MR, Maurício GN, Sobânia RLM. First records of the Silvery Grebe *Podiceps occipitalis* Garnot, 1826 in Brazil. Ararajuba. 2004;12(1):61-63 |
| 58 | Bornschein MR, Reinert BL. Acrescido de marinha em Pontal do Paraná: uma área a ser conservada para a manutenção das aves dos campos e banhados do litoral do Paraná, sul do Brasil. Anais do I Congresso Brasileiro de Unidades de Conservação; 1997; Curitiba, BR. Fundação Grupo Boticário; 1997. Volume II: Trabalhos técnicos, p 875-889. |
| 59 | Bornschein MR, Reinert BL. Aves de três remanescentes florestais do norte do Estado do Paraná, sul do Brasil, com sugestões para a conservação e manejo. Rev Bras Zool*.* 2000;17(3):615-636 |
| 60 | Bornschein MB, Reinert BL, Bóçon R. Novas informações sobre o ninho de e ovo da gralha-azul, *Cyanocorax caeruleus* (Corvidae). Ararajuba. 1996;4(1):32-34. |
| 61 | Bornschein MR, Reinert BL, Pichorim M. Aves dos campos e banhados do litoral do estado do Paraná. Anais do III Congresso Brasileiro de Ornitologia; 1993 Out 17-22. Pelotas, BR. Sociedade Brasileira de Ornitologia; 1993. Resumos P26. |
| 62 | Bornschein MR, Reinert BL, Pichorim M. Notas sobre algumas aves novas ou pouco conhecidas no sul do Brasil. Ararajuba. 1997;5(1):53-59 |
| 63 | Bornschein MR, Reinert BL, Pichorin M. Uma nova espécie de *Scytalopus* (Rhinocryptidae) descoberta no Estado do Paraná. Atualidades Orn. 1998;83:10. |
| 64 | Bornschein MR, Straube FC. Sobre o status atual de três espécies de aves no Estado do Paraná: *Crax fasciolata, Ara maracana* e *Psarocolius decumanus*. Anais do I Congresso Brasileiro de Ornitologia; 1991 Jul 21-27. Belém, BR. Sociedade Brasileira de Ornitologia; 1991. p.53. |
| 65 | Bornschein MR, Straube FC, Reinert BL, Pichorim M. Novos registros de aves para a Floresta Atlântica paranaense. Anais do III Congresso Brasileiro de Ornitologia; 1993 Out 17-22. Pelotas, BR. Sociedade Brasileira de Ornitologia; 1993. Resumos R44. |
| 66 | Boss RL, Sipinski AB, Bóçon R, Rivera R. Estudo preliminar da área de uso de filhotes de Amazona brasiliensis na Ilha Rasa, Paraná. Anais do XII Congresso Brasileiro de Ornitologia; 2004 Nov 21-26; Blumenau, BR. Sociedade Brasileira de Ornitologia; 2004. p 174. |
| 67 | Braga TV. O investimento em cuidado parental é igual entre os sexos em uma espécie monogâmica - *Furnarius rufus*? [dissertation]. Programa de Pós Graduação em Ecologia e Conservação: Universidade Federal do Paraná; 2012. |
| 68 | Brunetta B, Anjos L. Resultados preliminares de um estudo de ecologia da gralha-picaça (*Cyanocorax chrysops*) num remanescente florestal do norte do Paraná, Brasil. Anais do XV Congresso Brasileiro de Ornitologia, Brasil; 2007 Jul 1-6; Porto Alegre, BR. Sociedade Brasileira de Ornitologia; 2007. PAINEL EC34, p 253-254. |
| 69 | Buzzetti DRC, Uejima AMK, Gatto CAFR, Pichorim M. Dados preliminares sobre a ecologia de *Eleothreptus anomalus* (Caprimulgidae) no Parque Estadual de Vila Velha, Paraná. In: Straube FC, editor. Ornitologia sem fronteiras, incluindo os Resumos do IX Congresso Brasileiro de Ornitologia; 2001 Jul 22-27; Curitiba, BR. Fundação O Boticário de Proteção à Natureza; 2001. R32, p 42. |
| 70 | Campo-Martins F. Sucesso reprodutivo de *Sula leucogaster* (Pelecaniformes, Sulidae) nas Ilhas dos Currais, Paraná. In: Straube FC, editor. Ornitologia sem fronteiras, incluindo os Resumos do IX Congresso Brasileiro de Ornitologia; 2001 Jul 22-27; Curitiba, BR. Fundação O Boticário de Proteção à Natureza; 2001. R35, p 46. |
| 71 | Campos RP, Moura MO, Varassin IG. Elegibilidade de beija-flores pela coloração de flores na Reserva Natural Salto Morato, Paraná. Anais do XVII Congresso Brasileiro de Ornitologia; 2009 Jun 28-Jul 03; Aracruz, BR. Sociedade Brasileira de Ornitologia; 2009. Resumos (Sistemática, Evolução e Biogeografia), SEB345, p. 107. |
| 72 | Campos RP, Varassin IG. Potencial de aves dispersoras de *Miconia dodecandra* em floresta atlântica, Guaraqueçaba, PR. Anais do XVII Congresso Brasileiro de Ornitologia; 2009 Jun 28-Jul 03; Aracruz, BR. Sociedade Brasileira de Ornitologia; 2009. Resumos (Ecologia), EC142, p. 47. |
| 73 | Cândido-Junior JF, Conterno S. Levantamento preliminar da avifauna de mata do Parque Ambiental de Cascavel (Cascavel, Paraná). In: Straube FC, editor. Ornitologia sem fronteiras, incluindo os Resumos do IX Congresso Brasileiro de Ornitologia; 2001 Jul 22-27; Curitiba, BR. Fundação O Boticário de Proteção à Natureza; 2001. R37, p 49. |
| 74 | Carniel VL. Interação de aves costeiras com descartes oriundos da pesca artesanal no litoral centro sul paranaense [dissertation]. , Programa de Pós Graduação em Ciências Biológicas – Zoologia: Universidade Federal do Paraná; 2008. |
| 75 | Carniel VL, Krul R. Caracterização da colônia reprodutiva de *Larus dominicanus* no Arquipélago de Currais-PR. Anais do XV Congresso Brasileiro de Ornitologia, Brasil; 2007 Jul 1-6; Porto Alegre, BR. Sociedade Brasileira de Ornitologia; 2007. PAINEL BR17, p 112. |
| 76 | Carniel V, Krul R. A gaivota, *Larus dominicanus* no ambiente de entre-marés: influência da sazonalidade pesqueira nos padrões de interação com a pesca. Anais do XIII Congresso Brasileiro de Ornitologia; 2005 Out 30-Nov 04; Belém, BR. Sociedade Brasileira de Ornitologia; 2005. p 72. |
| 77 | Carniel V, Krul R. Interação de aves associadas a ecossistemas marinhos com a pesca no litoral centro-sul paranaense. Anais do XIII Congresso Brasileiro de Ornitologia; 2005 Out 30-Nov 04; Belém, BR. Sociedade Brasileira de Ornitologia; 2005. p 71. |
| 78 | Carniel V, Krul R. Kleptoparasitism in seabirds during interactions with artisanal fisheries on the coast of Paraná, south Brazil. Rev Bras Ornitol. 2011;19(4):461-468 |
| 79 | Carniel VL, Krul R. Interação de aves marinhas com diferentes artes de pesca praticadas pela frota artesanal no litoral centro-sul paranaense. Anais do XV Congresso Brasileiro de Ornitologia, Brasil; 2007 Jul 1-6; Porto Alegre, BR. Sociedade Brasileira de Ornitologia; 2007. PAINEL CP21, p 187-188. |
| 80 | Carrano E. Registros do mocho-diabo *Asio stygius* (Wagler, 1832) no Estado do Paraná. Atualidades Orn. 1998;85:2. |
| 81 | Carrano E. Composição e conservação da avifauna na Floresta Estadual do Palmito, município de Paranaguá, Paraná [dissertation]. Programa de Pós Graduação em Engenharia Florestal: Universidade Federal do Paraná; 2006. |
| 82 | Carrano E, Jablonski EF. Notas sobre a ocorrência da andorinha-do-mar-negra *Anous stolidus* (Linnaeus, 1758) (Aves-Laridae) para o Estado do Paraná, Brasil. Estud Biol. 1997;41:33-36. |
| 83 | Carrano E, Kuniyoshi YS. 2006. Frugivoria por aves na Floresta Estadual do Palmito, Paranaguá, Paraná. Anais do XIV Congresso Brasileiro de Ornitologia (História Natural); 2006 Jul 02-06; Ouro Preto, BR. Sociedade Brasileira de Ornitologia; 2006. R. 25, p 28. |
| 84 | Carrano E, Marins M. Dieta frugívora de *Ramphastos vitellinus* e *Selenidera maculirostris* na Floresta Estadual do Palmito, Paraná. Anais do XV Congresso Brasileiro de Ornitologia, Brasil; 2007 Jul 1-6; Porto Alegre, BR. Sociedade Brasileira de Ornitologia; 2007. PAINEL EC36, p 255. |
| 85 | Carrano E, Marins M. 2008. Frugivoria por aves em *Symplocos uniflora* (Symplocaceae) em floresta de baixada no município de Paranaguá, Paraná. Anais do XVI Congresso Brasileiro de Ornitologia; 2008 Jun 29-Jul 04; Palmas, BR. Sociedade Brasileira de Ornitologia; 2008. p 242. |
| 86 | Carrano E, Marins M. Frugivoria por traupídeos na planície litorânea paranaense. Anais do XVII Congresso Brasileiro de Ornitologia; 2009 Jun 28-Jul 03; Aracruz, BR. Sociedade Brasileira de Ornitologia; 2009. Resumos (Ecologia), EC146, p. 48. |
| 87 | Carrano E, Ribas CF. Novos registros de aves para a região de cerrado no Paraná. Atualidades Orn. 2000;94:12. |
| 88 | Carrano E, Santos REF, Patrial EW, Ribas CF, Klemann-Junior L. Composição e conservação de aves na Floresta Estadual do Palmito, município de Paranaguá, Paraná. Anais do XII Congresso Brasileiro de Ornitologia; 2004 Nov 21-26; Blumenau, BR. Sociedade Brasileira de Ornitologia; 2004. p 189. |
| 89 | Carrano E, Scherer-Neto P. Avifauna da Ilha Rasa, APA de Guaraqueçaba, Paraná. In: Straube FC, Argel M, Cândido JF, editors. Ornitologia brasileira no século XX:, incluindo os Resumos do VIII Congresso Brasileiro de Ornitologia; 2000 jul 09-14; Florianópolis, BR. Sociedade Brasileira de Ornitologia; 2000. R114:275-276. |
| 90 | Carrano E, Scherer-Neto P, Ribas CF. Descaracterização dos campos naturais no estado do Paraná e suas implicações na conservação da avifauna. Anais do XII Congresso Brasileiro de Ornitologia; 2004 Nov 21-26; Blumenau, BR. Sociedade Brasileira de Ornitologia; 2004. p 190. |
| 91 | Carrano E, Scherer-Neto P, Ribas CF, Klemann-Junior L. Novos registros de Falconiformes pouco comuns para os estados do Paraná e Santa Catarina. In: Straube FC, editor. Ornitologia sem fronteiras, incluindo os Resumos do IX Congresso Brasileiro de Ornitologia; 2001 Jul 22-27; Curitiba, BR. Fundação O Boticário de Proteção à Natureza; 2001. R40, p 52. |
| 92 | Carrano E, Straube FC. Sobre a distribuição e conservação de *Accipiter superciliosus* (Linnaeus, 1766) no Paraná (Accipitridae, Accipitriformes). Atualidades Orn. 2013;176:33-39. |
| 93 | Carvalho MO, Lazzarotto CM. Estudos de interações agonísticas entre espécies de beija-flores (Trochilidae) no zoológico de Curitiba, Paraná. Anais do I Congresso Brasileiro de Ornitologia; 1991 Jul 21-27. Belém, BR. Sociedade Brasileira de Ornitologia; 1991. p.28. |
| 94 | Chrostowski T. Kolekcja ornitologiczna ptaków paranskich. CR Soc Scient Var. 1912;5:452-500. |
| 95 | Chrostowski T. On some rare or little known species of southamerican birds. Ann Zool Mus Polon Hist Nat. 1921;1(1):31-40. |
| 96 | Clausi B. Contribuição à biologia de *Brotogeris tirica* (Gmelin, 1788), na região central da cidade de Curitiba, Paraná, Brasil. Anais do XI Congresso Brasileiro de Ornitologia; 2003 Nov 23-28; Feira de Santana, BR. Sociedade Brasileira de Ornitologia; 2003. p 139. |
| 97 | Clausi B. Dieta de *Brotogeris tirica* (Gmelin, 1788), na região central do município de Curitiba, Paraná. Anais do XI Congresso Brasileiro de Ornitologia; 2003 Nov 23-28; Feira de Santana, BR. Sociedade Brasileira de Ornitologia; 2003. p 138. |
| 98 | Clausi B. Aves mais frequentemente observadas se alimentando de frutas em duas áreas da região leste do estado do Paraná, Brasil. Anais do XIII Congresso Brasileiro de Ornitologia; 2005 Out 30-Nov 04; Belém, BR. Sociedade Brasileira de Ornitologia; 2005. p 184. |
| 99 | Clausi B, Baarstch C. Padrões de sazonalidade na produção de frutos em quatro florestas subtropicais em diferentes altitudes no leste do estado do Paraná e impactos na avifauna frugívora. Anais do XVII Congresso Brasileiro de Ornitologia; 2009 Jun 28-Jul 03; Aracruz, BR. Sociedade Brasileira de Ornitologia; 2009. Resumos (Ecologia), EC156, p. 50. |
| 100 | Conterno S, Cândido-Junior JF. Dados preliminares sobre a distribuição espacial da avifauna do Parque Ambiental de Cascavel, em Cascavel (Paraná). In: Straube FC, editor. Ornitologia sem fronteiras, incluindo os Resumos do IX Congresso Brasileiro de Ornitologia; 2001 Jul 22-27; Curitiba, BR. Fundação O Boticário de Proteção à Natureza; 2001. R53, p 69. |
| 101 | Corrêa L, Bazílio S. Registros de aves raras e ameaçadas em remanescentes florestais e reflorestamentos da empresa Araupel S.A. na região sudoeste do Paraná, Brasil. Anais do XVI Congresso Brasileiro de Ornitologia; 2008 Jun 29-Jul 04; Palmas, BR. Sociedade Brasileira de Ornitologia; 2008. p 397. |
| 102 | Corrêa L, Persegona JE, Roper JJ. O ciclo anual na composição e sazonalidade de uma comunidade de trochilídeos no sul do Brasil. Anais do XVII Congresso Brasileiro de Ornitologia; 2009 Jun 28-Jul 03; Aracruz, BR. Sociedade Brasileira de Ornitologia; 2009. Resumos (Ecologia), EC181, p. 57. |
| 103 | Dal'Maso A, Mikich SB. Avifauna em remanescentes da floresta ombrófila mista e plantios de *Pinus* spp. na região centro-sul do estado do Paraná. Anais do XII Congresso Brasileiro de Ornitologia; 2004 Nov 21-26; Blumenau, BR. Sociedade Brasileira de Ornitologia; 2004. p 204. |
| 104 | Dal'Maso A, Santos-Neto G, Pichorim M. Dieta do andorinhão-de-coleira-falha (*Streptoprocne biscutata*) (Sclater, 1865) (Aves, Apodidae) de duas localidades do estado do Paraná, Brasil. Anais do XI Congresso Brasileiro de Ornitologia; 2003 Nov 23-28; Feira de Santana, BR. Sociedade Brasileira de Ornitologia; 2003. p 140. |
| 105 | Deconto LR, Vallejos MAV. *Phacellodomus ferrugineigula* (Passeriformes: Furnariidae) no Paraná e comentários sobre sua distribuição no sul do Brasil. Atualidades Orn. 2010;157:10-11. |
| 106 | Dittrich J, Padial AA, Sipinski EAB, Abbud MC. Fontes de variação da morfometria de filhotes do papagaio-de-cara-roxa (*Amazona brasiliensis*). Anais do XX Congresso Brasileiro de Ornitologia; 2013 Nov 04-07; Passo Fundo, BR. Sociedade Brasileira de Ornitologia; 2013. p 273-274. |
| 107 | Dittrich J, Padial AA, Sipinski EAB, Abbud MC, Cavalheiro ML. Fontes de variação da morfometria de filhotes do papagaio-de-cara-roxa (*Amazona brasiliensis*). Anais do XX Congresso Brasileiro de Ornitologia; 2013 Nov 04-07; Passo Fundo, BR. Sociedade Brasileira de Ornitologia; 2013. p 81-82. |
| 108 | Domaniewski J. Übersicht der formen der gattung Picumnus Temm. Ann Zool Mus Polon Hist Nat. 1925;4(4):278-287 |
| 109 | Esclarski P, Menq W. Observações de *Spizaetus tyrannus* (Accipitriformes: Accipitridae) em Fênix, noroeste do Estado do Paraná. Atualidades Orn. 2011;160:20-21. |
| 110 | Esclarski P, Rosa T, Zanon C. Análise comparativa do comportamento da coruja buraqueira (*Athene cunicularia*) em ambientes urbanos e rurais. Anais do XX Congresso Brasileiro de Ornitologia; 2013 Nov 04-07; Passo Fundo, BR. Sociedade Brasileira de Ornitologia; 2013. p 453-454. |
| 111 | Favaro FL, Anjos L, Lopes EV, Mendonça LB, Volpato GH. Efeito do gradiente altitudinal/latitudinal sobre espécies de aves florestais da família Furnariidae na Bacia do Rio Tibagi, Paraná, Brasil. Rev Bras Zool. 2006;23(1):261-266 |
| 112 | Ferreira RC, Machado AA, Caxambu MG, Ide AL. Levantamento de espécies de aves e das espécies vegetais forrageadas na Estação Ecológica do Cerrado em Campo Mourão-PR. Atualidades Orn. 2005;127:28. |
| 113 | Festi L. Aves do ambiente praial, atividade alimentar e ocupação do espaço em relação a disponibilidade alimentar em praias oceânicas e estuarinas [dissertation]. Programa de Pós Graduação em Ecologia e Conservação: Universidade Federal do Paraná; 2011. |
| 114 | Festti L, Krul R. Aves do ambiente de entre-marés de Pontal do Sul, PR. Anais do XVI Congresso Brasileiro de Ornitologia; 2008 Jun 29-Jul 04; Palmas, BR. Sociedade Brasileira de Ornitologia; 2008. p 184. |
| 115 | Fieker AZ, Cândido-Junior JF, Almeida AC, Alves AC, Brocardo CR, Simão PXA, Reginato-Junior GB. Dieta do gavião-carijó, *Rupornis magnirostris* (Gmelin,1788), através da análise de conteúdos estomacais de animais atropelados. Anais do XVII Congresso Brasileiro de Ornitologia; 2009 Jun 28-Jul 03; Aracruz, BR. Sociedade Brasileira de Ornitologia; 2009. Resumos (Biologia Reprodutiva e Comportamento), BR21, p. 10. |
| 116 | Forcato A, Shiozawa MM, Saridakis DP, Tozato HC. Avifauna da Universidade Norte do Paraná, Campus Arapongas, PR, Brazil. UNOPAR. 2011;13(3):157-162. |
| 117 | Garcia PGF, Schanhofen CA. Salmonelose em aves marinhas na Baía de Paranaguá. Arq Biol Tecnol. 1982;25:237-242. |
| 118 | Ghizoni-Junior IR. Avifauna do município de Verê, sudoeste do Paraná. Anais do XII Congresso Brasileiro de Ornitologia; 2004 Nov 21-26; Blumenau, BR. Sociedade Brasileira de Ornitologia; 2004. p 230. |
| 119 | Gimenes MR, Anjos L. Distribuição espacial de aves em um fragmento florestal do campus da Universidade Estadual de Londrina, Norte do Paraná, Brasil. Rev Bras Zool. 2000;17(1):263-271 |
| 120 | Gimenes MR, Anjos L. Spatial distribution of birds on three islands in the upper river Paraná, Southern Brazil. Ornitol Neotrop. 2004;15(1):71-85. |
| 121 | Gimenes MR, Anjos L. Influence of lagoons size and prey availability on the wading birds (Ciconiiformes) in the upper Paraná river floodplain, Brazil. Braz Arch biol technol. 2006;49(3):463-473 |
| 122 | Gimenes MR, Anjos L. Quantitative analysis of foraging habitat use by Ciconiiformes in the upper Paraná river Floodplain, Brazil. Braz Arch biol technol. 2011;54(2):415-427 |
| 123 | Girardi F, Carrano E. First records of Masked Tityra *Tityra semifasciata* (Spix, 1825) for the state of Paraná, southern Brazil. Rev Bras Ornitol. 2014;22(4):416-418. |
| 124 | Gomes AB, Wasilewski M, Scherer-Neto P. Ocorrência do cisne-coscoroba *Coscoroba coscoroba* na Baía de Guaratuba, Paraná. Atualidades Orn. 2004;117:11. |
| 125 | Gomes ALM, Festti L, Rechetelo J, Ballabio TA, Carniel V, Krul R. Avaliação da comunidade de aves do entremarés de Pontal do Paraná, litoral paranaense, PR. Anais do XVII Congresso Brasileiro de Ornitologia; 2009 Jun 28-Jul 03; Aracruz, BR. Sociedade Brasileira de Ornitologia; 2009. Resumos (Ecologia), EC202, p. 62. |
| 126 | Gomes TM, Silva CAP, Jebai GT, Souza AR, Arakaki BR, Anjos L. Análise comparativa da densidade de espécies de aves no campus da Universidade Estadual de Londrina e no Lago Igapó I, na cidade de Londrina, norte do Paraná, Brasil. Anais do XV Congresso Brasileiro de Ornitologia, Brasil; 2007 Jul 1-6; Porto Alegre, BR. Sociedade Brasileira de Ornitologia; 2007. PAINEL EC11, p 235-236. |
| 127 | Gottschild A. Aspectos comportamentais de *Molothrus bonariensis* (Gmelin, 1789) ( Aves - Passeriformes - Icteridae) em Curitiba - Paraná. Atualidades Orn. 1998;87:2. |
| 128 | Gottschild A, Costa LCM. Estudo dos comportamentos de manutenção, agonístico e de reprodução do Molothrus bonariensis (Passeriformes, Icteridae) em Curitiba, Paraná. In: Straube FC, editor. Ornitologia sem fronteiras, incluindo os Resumos do IX Congresso Brasileiro de Ornitologia; 2001 Jul 22-27; Curitiba, BR. Fundação O Boticário de Proteção à Natureza; 2001. R89, p 114. |
| 129 | Guenther M, Nascimento-Júnior AF. A dispersão de sementes por aves e o processo de recuperação de matas ciliares, Marechal Cândido Rondon - Paraná. Anais do XII Congresso Brasileiro de Ornitologia; 2004 Nov 21-26; Blumenau, BR. Sociedade Brasileira de Ornitologia; 2004. p 239. |
| 130 | Guerra RS, Uejima AMK. Área de vida de *Amazona aestiva* na região norte de Curitiba, Paraná. In: Straube FC, editor. Ornitologia sem fronteiras, incluindo os Resumos do IX Congresso Brasileiro de Ornitologia; 2001 Jul 22-27; Curitiba, BR. Fundação O Boticário de Proteção à Natureza; 2001. R96, p 122. |
| 131 | Guimarães LR. Sobre alguns ectoparasitos de aves e mamíferos do litoral paranaense. Arq Mus Paran. 1945;4(7):179-190. |
| 132 | Gustman LGD, Bispo AA, Mikich SM. O uso de poleiros artificiais por aves em áreas degradadas da floresta estacional semidecidual. Anais do XV Congresso Brasileiro de Ornitologia, Brasil; 2007 Jul 1-6; Porto Alegre, BR. Sociedade Brasileira de Ornitologia; 2007. PAINEL EC38, p 256-257. |
| 133 | Hill III JR, Scherer-Neto P. Black vultures nesting on skyscrapers in southern Brazil. J Field Ornithol;62(2):173-176. |
| 134 | Hinkelmann C, Fiebig J. An early contribution to the avifauna of Paraná, Brazil. The Arkady Fiedler expedition of 1928/29. Bull Br Orn Club. 2001;121(2):116-127. |
| 135 | Hjort LC, Rechetelo J, Martins FA, Mestre LAM. Aves que se alimentam dos frutos de *Trema micrantha* em Palotina, Paraná. Anais do XX Congresso Brasileiro de Ornitologia; 2013 Nov 04-07; Passo Fundo, BR. Sociedade Brasileira de Ornitologia; 2013. p 76-77. |
| 136 | Ihering H von, Ihering R von. 1907. Catálogos da fauna brasileira editados pelo Museu Paulista, S.Paulo – Brazil. Volume I: As aves do Brazil. Tipografia do Diário Oficial, São Paulo; 1907. 485 pp. |
| 137 | Isfer O. Composição da avifauna do Parque Estadual Rio da Onça, Matinhos, Paraná, Brasil [monograph]. Curso de Biologia: Pontifícia Universidade Católica do Paraná; 1999. |
| 138 | Jaczewski T. The Polish Zoological Expedition to Brazil in the years 1921-1924. Itinerary and brief reports. Ann Zool Mus Polon Hist Nat. 1925;4(4):326-351. |
| 139 | Jebai GT, Arakaki BR, Silva CAP, Souza ÂR, Gomes TM, Anjos L. Análise comparativa da densidade de onze passeriformes em duas áreas urbanas em Londrina, norte do Paraná, Brasil. Rev Bras Ornitol. 2009;17(3-4):183-186 |
| 140 | Jesus S. Frugivoria e dispersão de sementes de *Myrsine coriacea* (Myrsinaceae) por aves. Anais do XIV Congresso Brasileiro de Ornitologia (Comportamento); 2006 Jul 02-06; Ouro Preto, BR. Sociedade Brasileira de Ornitologia; 2006. R. 3, p 7. |
| 141 | Jesus S. Utilização dos frutos de *Schinus terebinthifolius* (Anacardiaceae) por aves. Anais do XIV Congresso Brasileiro de Ornitologia (Comportamento); 2006 Jul 02-06; Ouro Preto, BR. Sociedade Brasileira de Ornitologia; 2006. R. 4, p. 8. |
| 142 | Jesus S. Dinâmicas populacionais de aves em remanescentes florestais urbanos [dissertation]. Programa de Pós Graduação em Ciências Biológicas – Zoologia: Universidade Federal do Paraná; 2008. |
| 143 | Jesus S, Casimiro MLL, Monteiro-Filho ELA. Comportamento cleptoparasita de *Pitangus sulphuratus* (Tyrannidae). Anais do XII Congresso Brasileiro de Ornitologia; 2004 Nov 21-26; Blumenau, BR. Sociedade Brasileira de Ornitologia; 2004. p 250. |
| 144 | Jesus S, Mikich SB. Registro de nidificação de *Dendrocolaptes platyrostris* (Dendrocolaptidae) em forro de edificação semi-rural. Rev Bras Ornitol. 2009;17(1):79-81 |
| 145 | Jesus S, Roper JJ, Batista L. Nidificação de *Florisuga fusca* (Aves: Trochilidae) no sul do Brasil. Anais do XV Congresso Brasileiro de Ornitologia, Brasil; 2007 Jul 1-6; Porto Alegre, BR. Sociedade Brasileira de Ornitologia; 2007. PAINEL BR09, p 106. |
| 146 | Jesus S, Roper JJ, Deconto LR, Machado T. Reprodução de *Thamnophilus caerulescens* (Aves: Thamnophilidae) em remanescentes florestais urbanos. Anais do XV Congresso Brasileiro de Ornitologia, Brasil; 2007 Jul 1-6; Porto Alegre, BR. Sociedade Brasileira de Ornitologia; 2007. PAINEL BR10, p 106-107. |
| 147 | Jesus S, Santos-Filho JF. Observações sobre a frugivoria por aves na erva-de-passarinho *Struthanthus vulgaris* (Loranthaceae) em área urbana. Atualidades Orn. 2011;161:4-6 |
| 148 | Kajiwara D, Urben-Filho A, Morato SAA. Dois registros recentes da águia-cinzenta (*Harpyhaliaetus coronatus*) no Estado do Paraná. In: Straube FC, editor. Ornitologia sem fronteiras, incluindo os Resumos do IX Congresso Brasileiro de Ornitologia; 2001 Jul 22-27; Curitiba, BR. Fundação O Boticário de Proteção à Natureza; 2001. R104, p 131. |
| 149 | Kaminski N, Carrano E. Comunidade de aves em um ecótone (Floresta Ombrófila Densa e F.O. Mista) na Serra do Cabral, município de Tijucas do Sul, Paraná. Anais do XII Congresso Brasileiro de Ornitologia; 2004 Nov 21-26; Blumenau, BR. Sociedade Brasileira de Ornitologia; 2004. p 252. |
| 150 | Kaminski N, Carrano E. Avifauna da Serra do Cabral e áreas adjacentes, Tijucas do Sul, Paraná [monograph]. Curso de Ciências Biológicas: Pontifícia Universidade Católica do Paraná; 2006. |
| 151 | Klemann-Junior L, Vieira JS. 2013. Assessing the extent of occurrence, area of occupancy, territory size, and population size of marsh tapaculo (*Scytalopus iraiensis*). Anim Biodivers Conserv. 2013;36(1):47-57. |
| 152 | Kohl LA, Treco FR. Levantamento da avifauna no interior do município de Francisco Beltrão/PR. Anais do XVII Congresso Brasileiro de Ornitologia; 2009 Jun 28-Jul 03; Aracruz, BR. Sociedade Brasileira de Ornitologia; 2009. Resumos (Faunística), FAU287, p. 87. |
| 153 | Kohler GU, Correa L, Belmonte-Lopes R, Bornschein MR, Reinert BL. First record of the Buff-breasted Sandpiper *Tryngites subruficollis* (Aves: Scolopacidae) in Santa Catarina state and an additional record for Paraná state, southern Brazil. Biotemas. 2010;23(2):223-225 |
| 154 | Koppe FR, Krul R. Estudo de uma população de *Speotyto cunicularia*: avaliação da dieta diferencial entre micro-habitats e das ações antrópicas, Pontal do Sul, PR. Anais do XII Congresso Brasileiro de Ornitologia; 2004 Nov 21-26; Blumenau, BR. Sociedade Brasileira de Ornitologia; 2004. p 253. |
| 155 | Krugel MM, Anjos L. Bird communities in forest remnants in the city of Maringá, Paraná State, Southern Brazil. Ornitol Neotrop. 2000;11(4):315-330. |
| 156 | Krügel MM, Behr ER. Consumo dos frutos de *Alchornea triplinervia* (Euphorbiaceae) por aves em fragmentos florestais urbanos de Maringá, Paraná. Biotemas. 1999;12(1):149-155 |
| 157 | Krul R. Avifauna de uma região de Cruz Machado, sul do Paraná. Anais do II Congresso Brasileiro de Ornitologia; 1992. Campo Grande, BR. Sociedade Brasileira de Ornitologia; 1992. Resumos R32. |
| 158 | Krul R. 2004. Aves marinhas costeiras do Paraná. In: Branco JO, editor. Aves marinhas e insulares brasileiras: bioecologia e conservação. Itajaí: Editora da UNIVALI; 2004. p. 37-56 |
| 159 | Krul R, Festti L, Gomes ALM, Carniel V, Rechetelo J, Mangini PR. Retorno do guará, *Eudocimus ruber*, ao litoral do Paraná, sul do Brasil: monitoramento da população e aspectos comportamentais. Anais do XVII Congresso Brasileiro de Ornitologia; 2009 Jun 28-Jul 03; Aracruz, BR. Sociedade Brasileira de Ornitologia; 2009. Resumos (Ecologia), EC178, p. 56. |
| 160 | Krul R, Moraes VS. Avifauna de capões de florestas com araucária. I.Parque Barigui, Curitiba, PR. Anais do II Congresso Brasileiro de Ornitologia; 1992. Campo Grande, BR. Sociedade Brasileira de Ornitologia; 1992. Resumo 33. |
| 161 | Krul R, Moraes VS. Aves do Parque Bariguí, Curitiba, PR. Biotemas. 1993;6(2):30-41. |
| 162 | Lange RB. Contribuição ao conhecimento da bionomia de aves: *Ramphastos dicolorus* L. (Ramphastidae), sua nidificação e ovos. Araucariana, série Zoologia. 1967;1:1-3. |
| 163 | Lange RB. Contribuição ao conhecimento da bionomia de aves: II. Observação do comportamento de *Tyto alba* (J.C.Gray). Estud Biol. 1981;7:1-27. |
| 164 | Lange RB, Lange MBR. Contribuição ao conhecimento da bionomia em Aves. III. Notas sobre a nidificação e alimentação de *Troglodytes aedon* Vieillot (Troglodytidae - Aves). Estud Biol. 1992;28:5-16. |
| 165 | Lanzer M, Vallejos MAV, Aurélio-Silva M. Primeiro registro documentado de *Accipiter poliogaster* (Temminck, 1824) no estado do Paraná, sul do Brasil (Falconiformes: Accipitridae). Rev Bras Ornitol. 2009;17(2):137-138 |
| 166 | Lara AI. Registros de *Netta peposaca* e *N. erythrophthalma* para o estado do Paraná. Anais do II Congresso Brasileiro de Ornitologia; 1992. Campo Grande, BR. Sociedade Brasileira de Ornitologia; 1992. Resumos R52 |
| 167 | Leite GA, Lima LC. Avifauna do Parque Ecológico Dr. Daisaku Ikeda – Londrina – Paraná. Anais do XVI Congresso Brasileiro de Ornitologia; 2008 Jun 29-Jul 04; Palmas, BR. Sociedade Brasileira de Ornitologia; 2008. p 317. |
| 168 | Leuchtenberger R, Roper JJ. A composição da dieta de *Procnias nudicollis* pode influir na qualidade do canto? Anais do XI Congresso Brasileiro de Ornitologia; 2003 Nov 23-28; Feira de Santana, BR. Sociedade Brasileira de Ornitologia; 2003. p 51. |
| 169 | Leuchtenberger R, Roper JJ. O canto de *Procnias nudicollis* informa à fêmea sobre a qualidade do cantor? Anais do XI Congresso Brasileiro de Ornitologia; 2003 Nov 23-28; Feira de Santana, BR. Sociedade Brasileira de Ornitologia; 2003. p 50. |
| 170 | Lima A, Abe LM, Fabri VC. Estudo comparativo da avifauna de um remanescente de Floresta Ombrófila Mista, o “Bosque Capão da Imbuia”, Curitiba, Paraná. Atualidades Orn. 2001;104:11. |
| 171 | Lima A, Fabri VC. Monitoramento da avifauna limícola e aquática do Parque Barigui (Curitiba, Paraná). In: Straube FC, editor. Ornitologia sem fronteiras, incluindo os Resumos do IX Congresso Brasileiro de Ornitologia; 2001 Jul 22-27; Curitiba, BR. Fundação O Boticário de Proteção à Natureza; 2001. R116, p 148. |
| 172 | Lima AMX. Sítios de nidificação de *Conopophaga melanops* (Conopophagidae) na Reserva Natural Salto Morato, Guaraqueçaba, PR. Anais do XV Congresso Brasileiro de Ornitologia, Brasil; 2007 Jul 1-6; Porto Alegre, BR. Sociedade Brasileira de Ornitologia; 2007. PAINEL BR14, p 109-110. |
| 173 | Lima AMX. Dinâmica populacional de aves de sub-bosque na Floresta Atlântica do Paraná [dissertation]. Programa de Pós Graduação em Ecologia e Conservação: Universidade Federal do Paraná; 2008. |
| 174 | Lima AMX, Rodrigues RG. Predação do arapaçu-de-bico-torto *Campylorhamphus falcularius* Vieillot 1822 (Dendrocolaptidae) sobre anfíbios anuros em um remanescente de Floresta com Araucárias. Rev Bras Ornitol. 2008;16(4):380-382 |
| 175 | Lima AMX, Rodrigues RG, Bittencourt S, Condrati LH, Roper JJ, Sanquetta CR. Levantamento preliminar da avifauna em remanescentes de floresta ombrófila mista próximos ao rio Iguaçu, ao sul do estado do Paraná, com novo registro para *Scytalopus iraiensis* Bornschein, Reinert e Pichorim, 1998 (Passeriformes: Rhinocryptidae). Anais do XII Congresso Brasileiro de Ornitologia; 2004 Nov 21-26; Blumenau, BR. Sociedade Brasileira de Ornitologia; 2004. p 265. |
| 176 | Lima AMX, Roper JJ. Fragmentos florestais em cidades como refúgio para aves. Anais do XI Congresso Brasileiro de Ornitologia; 2003 Nov 23-28; Feira de Santana, BR. Sociedade Brasileira de Ornitologia; 2003. p 179. |
| 177 | Lima AMX, Roper JJ. Nidoparasitismo por *Molothrus bonariensis* (Passeriformes: Icteridae) em fragmentos florestais em Curitiba, PR. Anais do XI Congresso Brasileiro de Ornitologia; 2003 Nov 23-28; Feira de Santana, BR. Sociedade Brasileira de Ornitologia; 2003. p 146. |
| 178 | Lima AMX, Roper JJ. Dinâmica populacional das aves de sub-bosque de floresta atlântica no Paraná. Anais do XV Congresso Brasileiro de Ornitologia, Brasil; 2007 Jul 1-6; Porto Alegre, BR. Sociedade Brasileira de Ornitologia; 2007. PAINEL EC23, p 245. |
| 179 | Lima AMX, Roper JJ. The use of playbacks can influence encounters with birds: an experiment. Rev Bras Ornitol. 2009;17(1):37-40 |
| 180 | Lopes EV, Anjos L. Registro de reprodução de *Nyctibius griseus* no campus da Universidade Estadual de Londrina, norte do Paraná. Ararajuba. 2005;13(1):109-112 |
| 181 | Lopes EV, Anjos L. A composição da avifauna do campus da Universidade Estadual de Londrina, norte do Paraná, Brasil. Rev Bras Zool. 2006;23(1):145-156 |
| 182 | Lopes EV, Anjos L, Fávaro FL, Volpato GH, Mendonça LB. Riqueza e abundância de aves papa-formiga (Thamnophilidae) florestais ao longo do gradiente altitudinal da bacia hidrográfica do rio Tibagi, Paraná, Brasil. Anais do XII Congresso Brasileiro de Ornitologia; 2004 Nov 21-26; Blumenau, BR. Sociedade Brasileira de Ornitologia; 2004. p 274. |
| 183 | Lopes EV, Anjos L, Loures-Ribeiro A, Gimenes MR, Mendonça LB, Volpato GH, Silva RJ. Efeito da fragmentação florestal sobre aves da família Formicariidae na região de Londrina, norte do Paraná. In: Straube FC, editor. Ornitologia sem fronteiras, incluindo os Resumos do IX Congresso Brasileiro de Ornitologia; 2001 Jul 22-27; Curitiba, BR. Fundação O Boticário de Proteção à Natureza; 2001. R117, p 149. |
| 184 | Lopes EV, Mendonça LB, Iborra GML, Anjos L. Comunidades de aves florestais do extremo noroeste do Paraná e sul do Mato Grosso do Sul: uma região de transição entre mata atlântica e cerrado. Anais do XVI Congresso Brasileiro de Ornitologia; 2008 Jun 29-Jul 04; Palmas, BR. Sociedade Brasileira de Ornitologia; 2008. p 202. |
| 185 | Lorenzetto A, Lindoso G, Pichorim M. A reprodução de bacurau-da-telha (*Caprimulgus longirostris*, Caprimulgidae) no estado do Paraná. Anais do XII Congresso Brasileiro de Ornitologia; 2004 Nov 21-26; Blumenau, BR. Sociedade Brasileira de Ornitologia; 2004. p 277. |
| 186 | Lorenzetto A, Pichorim M, Reinert BL, Salvo LM, Muller C, Lindoso G. Aspectos da ecologia da curucaca, *Theristicus caudatus* (Threskiornithidae), no Parque Estadual de Vila Velha, Paraná. Anais do XI Congresso Brasileiro de Ornitologia; 2003 Nov 23-28; Feira de Santana, BR. Sociedade Brasileira de Ornitologia; 2003. p 147. |
| 187 | Loures-Ribeiro A, Anjos L, Gimenes MR, Lopes EV, Mendonça LB, Silva RJ, Volpato GH. Registros de um ninho de *Ictinia plumbea* (Ordem: Falconiformes) em uma área urbana do município de Maringá (Paraná). In: Straube FC, editor. Ornitologia sem fronteiras, incluindo os Resumos do IX Congresso Brasileiro de Ornitologia; 2001 Jul 22-27; Curitiba, BR. Fundação O Boticário de Proteção à Natureza; 2001. R120, p 152. |
| 188 | Loures-Ribeiro A, Gimenes MR, Anjos L. Observações sobre o comportamento reprodutivo de *Ictinia plumbea* (Falconiformes: Accipitridae) no Campus da Universidade Estadual de Maringá, Paraná, Brasil. Ararajuba. 2003;11(1):85-87 |
| 189 | Luçolli SC. Ocorrência e distribuição da avifauna do Parque São Lourenço, Curitiba, PR. Anais do XV Congresso Brasileiro de Zoologia; 1988; Curitiba, BR. Sociedade Brasileira de Zoologia; 1988. p 504. |
| 190 | Luçolli SC. Incrementando a coleta de dados biológicos durante o processo de anilhamento de aves. Anais do I Congresso Brasileiro de Ornitologia; 1991 Jul 21-27. Belém, BR. Sociedade Brasileira de Ornitologia; 1991. p. 37. |
| 191 | Lugarini C, Serafini PP, Ribas JM, Teixeira VN, Sipinski E. Avaliação de endoparasitos em filhotes de papagaios-da-cara- roxa (*Amazona brasiliensis*) de vida livre. Anais do XII Congresso Brasileiro de Ornitologia; 2004 Nov 21-26; Blumenau, BR. Sociedade Brasileira de Ornitologia; 2004. p 280. |
| 192 | Macedo RC, Carrano E. Riqueza e estrutura da comunidade de aves no Campus II - PUCPR, Curitiba, Paraná. Anais do XVII Congresso Brasileiro de Ornitologia; 2009 Jun 28-Jul 03; Aracruz, BR. Sociedade Brasileira de Ornitologia; 2009. Resumos (Ecologia), EC190, p. 59. |
| 193 | Marcelino VR, Almeida AF. Levantamento da avifauna da Fazenda Figueira, Londrina, PR. Anais do XIV Congresso Brasileiro de Ornitologia (Faunística); 2006 Jul 02-06; Ouro Preto, BR. Sociedade Brasileira de Ornitologia; 2006.R. 13, p. 21. |
| 194 | Marcelino VR, Martins KG. Avifauna em Quatro Fazendas Madeireiras em Irati-PR. Floresta Ambient. 2014;21(2):127-139. |
| 195 | Marcelino VR, Martins KG, Ceruti F, Lopes GL. Aves da bacia do alto Imbituvão, Paraná. Anais do XX Congresso Brasileiro de Ornitologia; 2013 Nov 04-07; Passo Fundo, BR. Sociedade Brasileira de Ornitologia; 2013. p 270. |
| 196 | Marini MÂ, Reinert BL, Bornschein MR, Pinto JC, Pichorim MA. Ecological correlates of ectoparasitism of Atlantic Forest birds, Brazil. Ararajuba. 1996;4(2):93-102 |
| 197 | Marins M, Carrano E. Riqueza e abundância da família Trochilidae na Floresta Estadual do Palmito, Paranaguá, Paraná. Anais do XVI Congresso Brasileiro de Ornitologia; 2008 Jun 29-Jul 04; Palmas, BR. Sociedade Brasileira de Ornitologia; 2008. p 260. |
| 198 | Marques MCM, Britez RM. História natural e conservação da Ilha do Mel. Curitiba: Editora UFPR; 2005. 271p. |
| 199 | Marterer BE. 1990. Estudo populacional de *Zenaida auriculata chrysauchenia* (Reichenbach) (Aves, Columbidae) no norte e noroeste do Paraná. Anais do XVII Congresso Brasileiro de Zoologia; 1990 Jan 28-Fev 02; Londrina, BR. Sociedade Brasileira de Zoologia; 1990. p. 166. |
| 200 | Martins FC, Dias MM. Cuidado parental de *Sula leucogaster* (Boddaert) (Aves, Pelecaniformes, Sulidae) nas Ilhas dos Currais, Paraná, Brasil. Rev Bras Zool. 2003;20(4):583-589. |
| 201 | Martins GF, Cândido-Junior JF. Forrageio de aves em urtiga *Urera baccifera* no Parque Nacional do Iguaçu, Paraná, Brasil. Anais do XIV Congresso Brasileiro de Ornitologia (Comportamento); 2006 Jul 02-06; Ouro Preto, BR. Sociedade Brasileira de Ornitologia; 2006. R. 38, p. 42. |
| 202 | Mendonça LB, Anjos L. Feeding behavior of birds on *Erythrina speciosa* Andrews (Fabaceae) flowers in south Brazil. Anais do XI Congresso Brasileiro de Ornitologia; 2003 Nov 23-28; Feira de Santana, BR. Sociedade Brasileira de Ornitologia; 2003. p 56. |
| 203 | Mendonça LB, Anjos L. Feeding behavior of hummingbirds and perching birds on *Erythrina speciosa* Andrews (Fabaceae) flowers in an urban area, Londrina, Paraná, Brazil. Rev Bras Zool. 2006;23(1):42-49 |
| 204 | Mendonça LB, Anjos L, Volpato GH, Loures-Ribeiro A, Gimenes MR, Lopes EV. Uso de plantas nativas e exóticas pelos beija-flores (Trochilidae) numa área urbanizada em Londrina, norte do Paraná. In: Straube FC, editor. Ornitologia sem fronteiras, incluindo os Resumos do IX Congresso Brasileiro de Ornitologia; 2001 Jul 22-27; Curitiba, BR. Fundação O Boticário de Proteção à Natureza; 2001. R129, p 162. |
| 205 | Mestre LAM. Avifauna da Fazenda Arapongas - floresta com araucária - Lapa - Parana. Anais do XII Congresso Brasileiro de Ornitologia; 2004 Nov 21-26; Blumenau, BR. Sociedade Brasileira de Ornitologia; 2004. p 301. |
| 206 | Mestre LAM, Hjort LC, Martins FA. Comunidade de aves em Palotina, Paraná, Brasil. Anais do XX Congresso Brasileiro de Ornitologia; 2013 Nov 04-07; Passo Fundo, BR. Sociedade Brasileira de Ornitologia; 2013. p 113. |
| 207 | Mestre LAM, Krul R, Moraes VS. Mangrove bird community of Paranaguá Bay - Paraná, Brazil. Braz Arch Biol Technol. *2007;*50(1):75-83 |
| 208 | Mestre LAM, Torres RF, Festti L, Krul R, Roble-Junior JC, Faraco LF, Gomes BN. Estrutura da comunidade de aves em áreas modificadas e preservadas no parque nacional Saint-Hilaire/Lange – Paraná. Anais do XX Congresso Brasileiro de Ornitologia; 2013 Nov 04-07; Passo Fundo, BR. Sociedade Brasileira de Ornitologia; 2013. p 466-467. |
| 209 | Mikich SB. Aspectos de comportamento, frugivoria e utilização de habitat por tucanos de uma pequena reserva isolada do sul do Brasil (Piciformes, Ramphastidae). Anais do I Congresso Brasileiro de Ornitologia; 1991 Jul 21-27. Belém, BR. Sociedade Brasileira de Ornitologia; 1991. p.4. |
| 210 | Mikich SB. A importância da estatística nos estudos bioecológicos: análise do isolamento ecológico em ranfastídeos (Piciformes: Ramphastidae). Anais do II Congresso Brasileiro de Ornitologia; 1992. Campo Grande, BR. Sociedade Brasileira de Ornitologia; 1992. Resumos R54. |
| 211 | Mikich SB. A dieta frugívora de *Penelope superciliaris* (Cracidae) em remanescentes de Floresta Estacional Semidecidual no centro-oeste do Paraná, Brasil e sua relação com *Euterpe edulis* (Arecaceae). Ararajuba. 2002;10(2):207-217 |
| 212 | Milléo-Costa LC. Aspectos comportamentais de *Vanellus chilensis* (Wagler, 1827) (Charadriiformes, Aves). Anais do XII Congresso Brasileiro de Zoologia; 1985 Jan 27-Fev 01; Campinas, BR. Sociedade Brasileira de Zoologia; 1985. p. 260. |
| 213 | Milléo-Costa LC. 1986. Aspectos etológicos de *Vanellus chilensis* (Wagler, 1827) (Charadriiformes, Aves) relacionados com a territorialidade e ações agressivas intra-específicas. Anais do XIII Congresso Brasileiro de Zoologia; 1986; Cuiabá, BR. Sociedade Brasileira de Zoologia; 1986. Resumo 523, p.187. |
| 214 | Milléo-Costa LC. Aspectos do comportamento reprodutivo de *Vanellus chilensis* (Wagler, 1827) - (Charadriiformes, Charadriidae) em Curitiba, Paraná Brasil. Estud Biol. 1994;3(36):21-31. |
| 215 | Milléo-Costa LC. Manobras de distração de *Vanellus chilensis* (Wagler, 1827) (Charadriiformes, Charadriidae) em Curitiba, Paraná, Brasil. Estud Biol. 1994;3(36):33-42. |
| 216 | Milléo-Costa LC, Graf V. 1986. Estudo comportamental dos padrões motores de *Vanellus chilensis* (Charadriiformes, Aves) em habitat natural. Anais da 38ª Reunião Anual da SBPC; 1986 Jul 09-16; Curitiba, BR. Sociedade Brasileira para o Progresso da Ciência; 1986. Resumos G.1.11(30), p.1011. |
| 217 | Miranda TF, Carniel VL. Burlando o fratricídio obrigatório: experimento de manipulação de ninhadas com o atobá-marrom, *Sula leucogaster*. Anais do XX Congresso Brasileiro de Ornitologia; 2013 Nov 04-07; Passo Fundo, BR. Sociedade Brasileira de Ornitologia; 2013. p 329-330. |
| 218 | Mitroszewski A, Arzua M, Scherer-Neto P. Levantamento preliminar de aves em área adjacente ao Parque Estadual das Lauráceas, Vale do Ribeira, Paraná. Anais do XII Congresso Brasileiro de Ornitologia; 2004 Nov 21-26; Blumenau, BR. Sociedade Brasileira de Ornitologia; 2004. p 304. |
| 219 | Moraes VS. Avifauna da Ilha do Mel, litoral do Paraná. Arq Biol Tecnol. 1991;34(2):195-205. |
| 220 | Moraes VS. 1991b. Contribuição ao estudo do comportamento migratório de *Tangara peruviana* (Aves, Emberizidae). Anais do XVIII Congresso Brasileiro de Zoologia; 1991 Fev 26-Mar 01; Salvador, BR. Sociedade Brasileira de Zoologia; 1991. p.368. |
| 221 | Moraes VS. Notas sobre a ocorrência de alguns Charadriiformes no estado do Paraná. Anais do II Congresso Brasileiro de Ornitologia; 1992. Campo Grande, BR. Sociedade Brasileira de Ornitologia; 1992. Resumos R34. |
| 222 | Moraes VS. Novas observações sobre a avifauna da Ilha do Mel, Baía de Paranaguá, Paraná. Anais do II Congresso Brasileiro de Ornitologia; 1992. Campo Grande, BR. Sociedade Brasileira de Ornitologia; 1992. Resumos R35. |
| 223 | Moraes VS, Carvalho MO. Hábitos alimentares de *Milvago chimachima* (Falconidae, Falconiformes) em ambientes de beira-mar. Anais do I Congresso Brasileiro de Ornitologia; 1991 Jul 21-27. Belém, BR. Sociedade Brasileira de Ornitologia; 1991. p. 30. |
| 224 | Moraes VS, Krul R. Aves associadas a ecossistemas marinhos nos limites paranaenses. Anais do III Congresso Brasileiro de Ornitologia; 1993 Out 17-22. Pelotas, BR. Sociedade Brasileira de Ornitologia; 1993. Resumos R40. |
| 225 | Krul R, Moraes VS. Mortandades de aves marinhas em um eixo de praia arenosa do litoral do Paraná. Anais do III Congresso Brasileiro de Ornitologia; 1993 Out 17-22. Pelotas, BR. Sociedade Brasileira de Ornitologia; 1993. Resumos R25. |
| 226 | Moraes VS, Krul R. Aves associadas a ecossistemas de influência marítima no litoral do Paraná. Arq Biol Tecnol. 1995;38(1):121-134. |
| 227 | Moraes VSM, Krul R. Ocorrência e nidificação de *Macropsalis cragra* na ilha do Mel, Paraná, Brasil (Caprimulgiformes: Caprimulgidae). Ararajuba. 1995;3:79-80 |
| 228 | Moraes VS, Krul R. *Anous stolidus* (Linnaeus, 1758) (Charadriiformes: Sternidae) no sul do Brasil. Anais do V Congresso Brasileiro de Ornitologia; 1996 Jan 28-Fev 02; Campinas, BR. Sociedade Brasileira de Ornitologia; 1996. p.75. |
| 229 | Moraes VS, Krul R. Deslocamento de aves marinhas na costa brasileira: Expansão de limites de fronteira, rota migratória ou ocorrência acidental? Anais do VI Congresso Brasileiro de Ornitologia; 1997 Fev 24-28; Belo Horizonte, BR. Sociedade Brasileira de Ornitologia; 1997. p. 149. |
| 230 | Moraes VS, Krul R. Efeitos da ocupação antrópica sobre comunidades de aves de ilhas das baías de Laranjeiras e Guaraqueçaba - PR. Biotemas. 1999;12(2):101-118 |
| 231 | Moraes VS, Pichorim M. Oviposição da batuíra-da-praia *Charadrius collaris* na Ilha do Mel, Paraná. Anais do I Congresso Brasileiro de Ornitologia; 1991 Jul 21-27. Belém, BR. Sociedade Brasileira de Ornitologia; 1991. p. 29. |
| 232 | Munson ES, Robinson WD. Extensive folivory by thickbilled saltators (*Saltator maxillosus*) in southern Brazil. Auk. 1992;109(4):917-920. |
| 233 | Naumburg EMB. Studies of birds from Eastern Brazil and Paraguay, based on a collection made by Emil Kaempfer. B Am Mus Nat Hist. 1937;74:139-205. |
| 234 | Naumburg EMB. Studies of birds from Eastern Brazil and Paraguay, based on a collection made by Emil Kaempfer. B Am Mus Nat Hist. 1939;76:231-276. |
| 235 | Oliveira RC, Arasaki MO. Novos registros de *Notharchus swainsoni* (Galbuliformes: Bucconidae) na região noroeste do Estado do Paraná e ampliação da área de ocorrência no estado. Atualidades Orn. 2011;162: 6-7. |
| 236 | Oliveira SL. Ciclo reprodutivo e densidade populacional da araponga (Aves:Cotingidae): uma abordagem metodológica [dissertation]. Programa de Pós Graduação em Ecologia e Conservação: Universidade Federal do Paraná; 2012. |
| 237 | Oliveira TCG. Estudo comparativo das relações intra-específicas do *Phalacrocorax brasilianus* (Gmelin, 1789) em Curitiba e no litoral do estado do Paraná, Brasil [dissertation]. Programa de Pós Graduação em Ciências Biológicas – Zoologia: Universidade Federal do Paraná; 2005. |
| 238 | Oliveira TCG, Costa LCM. Análise comportamental de *Phalacrocorax brasilianus* na Ilha dos Ratos (Baía de Guaratuba-PR) e nos Parques Municipal do Barigui e Municipal São Lourenço, Curitiba, Paraná. In: Straube FC, editor. Ornitologia sem fronteiras, incluindo os Resumos do IX Congresso Brasileiro de Ornitologia; 2001 Jul 22-27; Curitiba, BR. Fundação O Boticário de Proteção à Natureza; 2001. R147, p 182. |
| 239 | Oliveira TCG, Milléo-Costa LC. Distribuição espacial do *Phalacrocorax brasilianus* por níveis de ocupação nos galhos das árvores do Parque São Lourenço, Curitiba, Paraná, Brasil. Atualidades Orn. 2002;108:3. |
| 240 | Oliveira TCG, Milléo-Costa LC, Graf V. Atividades comportamentais associadas com o deslocamento do *Phalacrocorax brasilianus* na Baía de Guaratuba, litoral sul do Estado do Paraná, Brasil. Atualidades Orn. 2004;121:4. |
| 241 | Olmos F. Revisão dos registros de *Stercorarius pomarinus* no Brasil, com notas sobre registros de *S. longicaudus* e *S. parasiticus* (Charadriiformes: Stercorariidae). Nattereria. 2000;1:29-33. |
| 242 | Olmos F. Revisão dos registros de *Procellaria conspicillata* no Brasil, com novas observações sobre sua distribuição. Nattereria. 2001;2:16-18. |
| 243 | Parrini R, Pacheco JF, Soares BR. Observações sobre a dieta e comportamento alimentar de *Saltator maxillosus* (Passeriformes: Cardinalidae) na Floresta Atlântica serrana das Regiões Sudeste e Sul do Brasil. Atualidades Orn. 2009;150:33-36. |
| 244 | Patrial EW. Registro documentado de *Anhima cornuta* (Aves: Anhimidae) para o Estado do Paraná. Atualidades Orn. 2008;146:41-42. |
| 245 | Patrial EW. *Arremon semitorquatus* Swainson, 1838 (Passeriformes: Emberizidae) no norte do Paraná: novo registro documentado e comentários sobre sua distribuição geográfica meridional. Atualidades Orn. 2010;154:18-21. |
| 246 | Pedroso-Junior NN. Microhabitat occupation by birds in a restinga fragment of Paraná coast, PR, Brazil. Braz Arch Biol Technol. 2003;46(1):83-90 |
| 247 | Pedroso-Junior NN. Tipificação da avifauna associada a uma faixa de restinga no litoral paranaense. In: Straube FC, editor. Ornitologia sem fronteiras, incluindo os Resumos do IX Congresso Brasileiro de Ornitologia; 2001 Jul 22-27; Curitiba, BR. Fundação O Boticário de Proteção à Natureza; 2001. R152, p 188. |
| 248 | Pelanda AA, Rechetelo J, Ballabio TA, Carniel V, Krul R. Ocorrência e mortalidade de *Spheniscus magellanicus* no litoral paranaense. Anais do XV Congresso Brasileiro de Ornitologia, Brasil; 2007 Jul 1-6; Porto Alegre, BR. Sociedade Brasileira de Ornitologia; 2007. PAINEL DM15, p 153. |
| 249 | Pelanda A, Carrano E. Composição e a importância da preservação de rapinantes diurnos (Aves: Accipitridae e Falconidae) em um trecho do alto rio Iguaçu, estado do Paraná. Revista Meio Ambiente e Sustentabilidade. 2013;3(2):176-196 |
| 250 | Pelzeln A von. Zur ornithologie brasiliensis. Resultate von Johann Natterers reisen in den Jahren 1817 bis 1835. Viena, Witwe & Sohn; 1871. 462 p. |
| 251 | Pereira LMS, Costa LCM. Hábitos comportamentais de coruja-buraqueira, *Speotyto cunicularia* (Aves, Strigidae) em Curitiba, Paraná. In: Straube FC, editor. Ornitologia sem fronteiras, incluindo os Resumos do IX Congresso Brasileiro de Ornitologia; 2001 Jul 22-27; Curitiba, BR. Fundação O Boticário de Proteção à Natureza; 2001. R153, p 189. |
| 252 | Persegona JE, Roper JJ. Interações estacionais entre beija-flores no sul do Brasil. Anais do XVII Congresso Brasileiro de Ornitologia; 2009 Jun 28-Jul 03; Aracruz, BR. Sociedade Brasileira de Ornitologia; 2009. Resumos (Biologia Reprodutiva e Comportamento), BR51, p. 18. |
| 253 | Philippsen JS, Ramos CCO, Benedito E, Zawadzki CH. Avifauna predominante no campus-sede da Universidade Estadual de Maringá (PR): composição, riqueza e guilda alimentar. Anais do XVI Congresso Brasileiro de Ornitologia; 2008 Jun 29-Jul 04; Palmas, BR. Sociedade Brasileira de Ornitologia; 2008. p 190. |
| 254 | Piacentini VQ, Varassin IG. Influência da floração de bromélias sobre uma comunidade de beija-flores no sul do Brasil. Anais do XIII Congresso Brasileiro de Ornitologia; 2005 Out 30-Nov 04; Belém, BR. Sociedade Brasileira de Ornitologia; 2005. p 168. |
| 255 | Pichorim M. Biologia reprodutiva do bacurau-tesoura-gigante (*Macropsalis forcipata*, Caprimulgidae) no morro Anhangava, Paraná, Sul do Brasil. Ararajuba. 2002:10(2):149-165 |
| 256 | Pichorim M. The breeding biology of the biscutate swift (*Streptoprocne biscutata*) in Southern Brazil. Ornitol Neotrop. 2002:13(1): 61-84. |
| 257 | Pichorim M. 2011. The influence of clutch and brood sizes on nesting success of the biscutate swift, *Streptoprocne biscutata* (Aves: Apodidae). Rev Bras Zool. 2011;28(2):186-192 |
| 258 | Pichorim M, Bóçon R. Estudo da composição avifaunística dos municípios de Rio Azul e Mallet, Paraná, Brasil. Acta Biol Leopoldensia. 1996;18(1):129-144. |
| 259 | Pichorim M, Bornschein MR. Primeiros registros de *Panyptila cayennensis* no Paraná e comentários sobre a suposta ocorrência de *Tachornis squamata* no estado (Apodidae). In: Straube FC, editor. Ornitologia sem fronteiras, incluindo os Resumos do IX Congresso Brasileiro de Ornitologia; 2001 Jul 22-27; Curitiba, BR. Fundação O Boticário de Proteção à Natureza; 2001. R157, p 311-312. |
| 260 | Pichorim M, Bornschein MR, Reinert BL. Aspectos da biologia reprodutiva de *Knipolegus nigerrimus.* Ararajuba. 1996;4(1):29-31. |
| 261 | Pichorim M, Lorenzetto A, Bornschein M R. Reprodução e novos registros de *Cypseloides fumigatus* (Apodidae) no Estado do Paraná. In: Straube FC, editor. Ornitologia sem fronteiras, incluindo os Resumos do IX Congresso Brasileiro de Ornitologia; 2001 Jul 22-27; Curitiba, BR. Fundação O Boticário de Proteção à Natureza; 2001. R158, p 195. |
| 262 | Pichorim M, Monteiro Filho ELA, Lorenzetto A, Bispo AA, Dal'Maso A. Sucesso reprodutivo em três colônias de *Streptoprocne biscutata* (Apodidae) do leste do estado do Paraná, Sul do Brasil. Anais do XI Congresso Brasileiro de Ornitologia; 2003 Nov 23-28; Feira de Santana, BR. Sociedade Brasileira de Ornitologia; 2003. p 11. |
| 263 | Pichorim M, Roper JJ, Monteiro-Filho EL. Tamanho da ninhada e sua importância no crescimento de *Streptoprocne biscutata* (Apodidae). Anais do XI Congresso Brasileiro de Ornitologia; 2003 Nov 23-28; Feira de Santana, BR. Sociedade Brasileira de Ornitologia; 2003. p 10. |
| 264 | Pinto OMO, Camargo EA. Lista anotada de aves colecionadas nos limites ocidentais do Estado do Paraná. Pap Avulsos de Zoologia (São Paulo). 1956;12(9):215-234. |
| 265 | Poletto F, Anjos L, Lopes EV, Fávaro F, Volpato GH, Serafini P. Caracterização do microhabitats segregação ecológica de cinco espécies de arapaçus (Aves: Dendrocolaptidae) em um fragmento florestal da região de Londrina, norte do Paraná. Anais do XI Congresso Brasileiro de Ornitologia; 2003 Nov 23-28; Feira de Santana, BR. Sociedade Brasileira de Ornitologia; 2003. p 31. |
| 266 | Poletto F, Anjos L, Lopes EV, Volpato GH, Serafini PP, Favaro FL. Caracterização do microhabitat e vulnerabilidade de cinco espécies de arapaçus (Aves: Dendrocolaptidae) em fragmento florestal do norte do estado do Paraná, sul do Brasil. Ararajuba. 2004;12(2):89-96 |
| 267 | Ramos CCO, Benedito E, Zawadzki CH. Dieta e conteúdo calórico de aves atropeladas na região central do estado do Paraná, Brasil. Biotemas. 2011;24(4):153-170 |
| 268 | Ramos CCO, Philippsen JS, Anjos L. Riqueza da avifauna à margem de dois riachos na planície alagável do alto rio Paraná, Paraná, Brasil. Anais do XVI Congresso Brasileiro de Ornitologia; 2008 Jun 29-Jul 04; Palmas, BR. Sociedade Brasileira de Ornitologia; 2008. p 259. |
| 269 | Raposo MA. A new species of Arremon (Aves: Emberizidae) from Brazil. Ararajuba. Rev Bras Ornitol. 1997;5(1):1-9. |
| 270 | Raposo MA, Parrini R. On the validity of the Half-collared Sparrow *Arremon semitorquatus* Swainson, 1837. Bull Br Orn Club. 1997;117(4):294-298. |
| 271 | Rechetelo J, Ballabio TA, Festti L, Gomes ALM, Carniel V, Krul R. Mortalidade de aves marinhas em um trecho do litoral paranaense, Pontal do Paraná, PR. Anais do XVII Congresso Brasileiro de Ornitologia; 2009 Jun 28-Jul 03; Aracruz, BR. Sociedade Brasileira de Ornitologia; 2009. Resumos (Ecologia), EC203, p. 62. |
| 272 | Rechetelo J, Monteiro-Filho ELA, Krul R. Avaliação da estação reprodutiva de *Nyctanassa violacea* em uma área de manguezal no estado do Paraná. Anais do XV Congresso Brasileiro de Ornitologia, Brasil; 2007 Jul 1-6; Porto Alegre, BR. Sociedade Brasileira de Ornitologia; 2007. PAINEL BR30, p 121-122. |
| 273 | Reinert BL, Bornschein MR. Descrição do macho adulto de *Stymphalornis acutirostris* (Aves: Formicariidae). Ararajuba. 1996;4(2):103-105 |
| 274 | Reinert BL, Bornschein MR. Alimentação da gralha-azul (*Cyanocorax caeruleus*, Corvidae). Ornitol Neotrop. 1998;9(2): 213-217. |
| 275 | Reinert BL, Bornschein MR, Firkowski C. Distribuição, tamanho populacional, hábitat e conservação do bicudinho-do-brejo *Stymphalornis acutirostris* Bornschein, Reinert e Teixeira, 1995 (Thamnophilidae). Rev Bras Ornitol. 2007;15(4):493-519 |
| 276 | Ribas CF. Levantamento prévio de passeriformes da Reserva Particular do Patrimônio Natural Papagaios Velhos, município de Palmeira-PR, Brasil. Atualidades Orn. 1998;81:2. |
| 277 | Ribas CF, Santos REF. Novo registro documentado do caburé-acanelado *Aegolius harrisii* (Cassin, 1849) para o estado do Paraná. Atualidades Orn. 2007;140:4-5. |
| 278 | Ribas CF, Santos REF. Observações do gavião-de-penacho *Spizaetus ornatus* (Daundin, 1800) nos estados de São Paulo e Paraná. Atualidades Orn. 2008;145:12-13. |
| 279 | Rodrigues LC, Almeida AF, Kikuti P, Speltz RM. 1981. Estudo comparativo da avifauna em mata natural e em plantio homogêneo de *Araucaria angustifolia* (Bert.) O.Ktze. IPEC, Circular Técnica. 1981;132:1-7. |
| 280 | Rodriguez MN, Roper JJ. 2011. An experimental test of the benefits of hatching asynchrony in the Rufous Hornero (*Furnarius rufus*). Rev Bras Ornitol. 2011;19(1):17-21. |
| 281 | Roos AL, Piacentini VQ. Revisão dos registros sulbrasileiros do gênero *Phoebetria* Reichenbach, 1853 e primeiro registro documentado de *Phoebetria palpebrata* (Forster, 1785) (Procellariiformes: Diomedeidae) para Santa Catarina. Ararajuba. 2003;11(2):223-225 |
| 282 | Ruschi A. Os nomes vulgares dos beija-flores do Estado do Paraná. Bol Mus Biol Mello Leitão (Divulgação). 1964;24:1-3. |
| 283 | Saboia J, Roper JJ. Influência do comportamento alimentar de aves frugívoras na dispersão de sementes em uma área de Floresta Ombrófila Mista, Tijucas do Sul, Paraná. In: Straube FC, editor. Ornitologia sem fronteiras, incluindo os Resumos do IX Congresso Brasileiro de Ornitologia; 2001 Jul 22-27; Curitiba, BR. Fundação O Boticário de Proteção à Natureza; 2001. R181, p 223. |
| 284 | Sabóia J, Sobânia R. Avifauna do Parque Ecoturístico São Luiz de Tolosa (Rio Negro, Paraná) e adjacências. Anais do XI Congresso Brasileiro de Ornitologia; 2003 Nov 23-28; Feira de Santana, BR. Sociedade Brasileira de Ornitologia; 2003. p 120. |
| 285 | Santana CR, Anjos L. Associação de aves a agrupamentos de bambu na porção sul da Mata Atlântica, Londrina, Estado do Paraná, Brasil. Biota Neotropica. 2010;10(2):39-44 |
| 286 | Santos CAK. Aves que nidificam em cavidades na Reserva Natural Salto Morato - Guaraqueçaba (PR) [dissertation]. Programa de Pós Graduação em Ecologia e Conservação: Universidade Federal do Paraná; 2007. |
| 287 | Santos REF. Novo registro documentado do galito *Alectrurus tricolor* (Vieillot, 1816) para o estado do Paraná, Brasil. Atualidades Orn. 2007;140:12-13. |
| 288 | Santos REF. Atropelamento de *Aegolius harrisii* na BR-280 e ampliação de sua distribuição no estado do Paraná. Atualidades Orn. 2009;147: 44-45. |
| 289 | Santos REF, Patrial EW, Carrano E. Composição, estrutura e conservação da avifauna do distrito do Bugre, Balsa Nova, Paraná, Brasil. Anais do XII Congresso Brasileiro de Ornitologia; 2004 Nov 21-26; Blumenau, BR. Sociedade Brasileira de Ornitologia; 2004. p 361. |
| 290 | Santos WM, Copatti JF. Registro documentado de *Spizaetus melanoleucus* (Falconiformes: Accipitridae) na Reserva Biológica das Perobas, estado do Paraná. Atualidades Orn. 2009;151: 18-19. |
| 291 | Sberze M, Niedfeld M, Uejima A, Monteiro-Filho E. Distribuição e ambiente utilizado por *Caprimulgus longirostris* (Bonaparte, 1825) no Parque Estadual de Vila Velha, Ponta Grossa, Paraná. Anais do XVI Congresso Brasileiro de Ornitologia; 2008 Jun 29-Jul 04; Palmas, BR. Sociedade Brasileira de Ornitologia; 2008. p 217. |
| 292 | Scherer-Neto P. Aves do Paraná. Nilópolis: Fundação Zoobotânica Mário Nardelli; 1980. 32 p. |
| 293 | Scherer-Neto P. Aspectos bionômicos e desenvolvimento de *Theristicus caudatus* (Boddaert, 1783) (Aves, Threskiornithidae). Dusenia. 1982;13(4):145-149. |
| 294 | Scherer-Neto P. Avifauna do extinto Parque Nacional de 7 Quedas, Guaíra, estado do Paraná. Arq Biol Tecnol. 1983;26(4):488-494. |
| 295 | Scherer-Neto P. Lista de aves do Estado do Paraná. Curitiba: Secretaria de Estado da Cultura e do Esporte. Folheto, sanfonado com 14 páginas; 1983. |
| 296 | Scherer-Neto P. 1983b. Observações sobre nidificação e filhotes de bacurau-pequeno *Caprimulgus parvulus* Gould, 1837, na natureza. Anais do X Congresso Brasileiro de Zoologia; 1983 Jan 30-Fev 04; Belo Horizonte, BR. Sociedade Brasileira de Zoologia; 1983. Resumo 275, p. 351. |
| 297 | Scherer-Neto P. Anilhamento de aves marinhas na Ilha dos Currais, Estado do Paraná. Anais do 1º Encontro Nacional de Anilhadores de Aves; 1985 Jan 21-24; Viçosa, BR. Universidade Federal de Viçosa; 1985. p. 64. |
| 298 | Scherer-Neto P. Lista de aves do Estado do Paraná. Curitiba: Prefeitura Municipal de Curitiba. Folheto, brochura com 13 p.; 1985. |
| 299 | Scherer-Neto P. Notas bionômicas sobre *Amazona brasiliensis* (Linnaeus, 1758) (Psittacidae, Aves). Anais do XII Congresso Brasileiro de Zoologia; 1985 Jan 27-Fev 01; Campinas, BR. Sociedade Brasileira de Zoologia; 1985. p. 262. |
| 300 | Scherer-Neto P. Notas bionômicas sobre o "mocho-diabo" *Asio stygius* (Wagler, 1832), no Paraná. Anais Soc Sul-Riogrand Ornit. 1985;6:15-18. |
| 301 | Scherer-Neto P. Nova ocorrência da "pomba-antártica" (*Chionis alba* Gmelin, 1789), no sul do Brasil. Anais Soc Sul-Riogrand Ornit. 1985; 6:19-20. |
| 302 | Scherer-Neto P. Nota sobre aspectos migratórios de *Fregata magnificens* (Matthews, 1914) (Fregatidae, Aves). Anais do II Encontro Nacional de Anilhadores de Aves; 1987 Jul 27-31; Rio de Janeiro, BR. Universidade Federal do Rio de Janeiro; 1987. R.34. |
| 303 | Scherer-Neto P. Contribuição à biologia do papagaio-de-cara-roxa *Amazona brasiliensis* (Linnaeus, 1758) (Psitacidae, Aves) [dissertation]. Programa de Pós Graduação em Ciências Biológicas – Zoologia: Universidade Federal do Paraná; 1989. |
| 304 | Scherer-Neto P, Anjos L, Straube FC. Composição ornitofaunística do Parque Florestal de Caxambu, Castro, Paraná. Anais do XIV Congresso Brasileiro de Zoologia; 1987 Feb 01-06; Juiz de Fora, BR. Sociedade Brasileira de Zoologia; 1987. p 154. |
| 305 | Scherer-Neto P, Anjos L, Straube F. Avifauna do Parque Estadual de Vila Velha, Estado do Paraná. Arq Biol Tecnol. 1994;37(1):223-229. |
| 306 | Scherer-Neto P, Bispo AA. Avifauna do Parque Estadual de Vila Rica do Espírito Santo, Fênix, Paraná. Biota Neotropica. 2011;11(3):317-329 |
| 307 | Scherer-Neto P, Carrano E. *Gampsonyx swainsonii* (Vigors, 1825) no Estado do Paraná. Atualidades Orn. 1998;82:2. |
| 308 | Scherer-Neto P, Carrano E. Ocorrência da lavadeira-mascarada *Fluvicola nengeta* (Linnaeus, 1766) no Estado do Paraná. Atualidades Orn. 1998;82: 11. |
| 309 | Scherer-Neto P, Carrano E, Moura-Britto M, Girardi F, Klemann-Junior L, Amorin R, Torre GD, Macedo LFF. Atualização do conhecimento sobre a avifauna do Parque Estadual do Guartelá, Paraná, Brasil. In: Carpanezzi OTB, Campos JB, editors. Coletânea de Pesquisas: Parques Estaduais de Vila Velha, Cerrado e Guartelá. Curitiba: Idealle Editora e Publicidade Ltda; 2011. p. 346-355. |
| 310 | Scherer-Neto P, Carrano E, Ribas CF. Avifauna da Estação Ecológica do Caiuá (Diamante do Norte, Paraná) e regiões adjacentes. In: Straube FC, editor. Ornitologia sem fronteiras, incluindo os Resumos do IX Congresso Brasileiro de Ornitologia; 2001 Jul 22-27; Curitiba, BR. Fundação O Boticário de Proteção à Natureza; 2001. R192, p 236. |
| 311 | Scherer -Neto P, Carrano E, Ribas CF. Diagnóstico da avifauna na região estuarina da Baía de Antonina, Paraná. Anais do XII Congresso Brasileiro de Ornitologia; 2004 Nov 21-26; Blumenau, BR. Sociedade Brasileira de Ornitologia; 2004. p 311. |
| 312 | Scherer-Neto P, Carrano E, Ribas CF. Monitoramento da avifauna aquática e limícola no trecho superior do rio Iguaçu, Paraná. Anais do XII Congresso Brasileiro de Ornitologia; 2004 Nov 21-26; Blumenau, BR. Sociedade Brasileira de Ornitologia; 2004. p 313. |
| 313 | Scherer-Neto P, Carrano E, Ribas CF. Composição e conservação da avifauna da Estação ecológica do Caiuá, noroeste do Paraná e regiões adjacentes. Cadernos de Biodiversidade. 2008;6(1):32. |
| 314 | Scherer-Neto P, Kajiwara D. *Pipra fasciicauda* (Pipridae, Aves) no Parque Estadual de Vila Rica do Espírito Santo, Fênix, Paraná. Atualidades Orn. 1997;75: 7. |
| 315 | Scherer-Neto P, Kajiwara D, Carrano E, Abe LM. Novos registros de gavião-pega-macaco *Spizaetus tyrannus* (Wied, 1820) para os Estados do Paraná e Santa Catarina. Atualidades Orn. 1997;79:11. |
| 316 | Scherer-Neto P, Klemann-Junior L, Torre GD, Amorin R, Carrano E. Inventários da avifauna do Parque Estadual de Vila Velha, uma atualização do conhecimento. In: Carpanezzi OTB, Campos JB, editors. Coletânea de Pesquisas: Parques Estaduais de Vila Velha, Cerrado e Guartelá. Curitiba: Idealle Editora e Publicidade Ltda; 2011. p. 75-79. |
| 317 | Scherer-Neto P, Mueller JA. Aspectos bionômicos de cuiucuiu *Pionopsitta pileata* Scopoli, 1769 com ênfase à fase evolutiva de filhotes. Anais do X Congresso Brasileiro de Zoologia; 1983 Jan 30-Fev 04; Belo Horizonte, BR. Sociedade Brasileira de Zoologia; 1983. Resumo 267, p. 342. |
| 318 | Scherer-Neto P, Mueller JA. Aspectos bionômicos de cuiucuiu *Pionopsitta pileata* (Scopoli, 1769) (Psittacidae, Aves). Arq Biol Tecnol. 1984;27(3):391-397. |
| 319 | Scherer-Neto P, Ramos FF, Gonçalves VP. Registro documentado do pisa-na-água *Phalaropus tricolor* (Vieillot, 1819) no estado do Paraná, Brasil. Atualidades Orn. 2008;143: 7. |
| 320 | Scherer-Neto P, Ribas CF. Registro de harpia *Harpia harpyja* no litoral sul do Brasil. Atualidades Orn. 2004;122:2. |
| 321 | Scherer-Neto P, Ribas CF, Carrano E, Kajiwara D, Latenek SR. Morfometria, peso e mudas do macuquinho-da-várzea *Scytalopus iraiensis*. In: Straube FC, editor. Ornitologia sem fronteiras, incluindo os Resumos do IX Congresso Brasileiro de Ornitologia; 2001 Jul 22-27; Curitiba, BR. Fundação O Boticário de Proteção à Natureza; 2001. R193, p 227. |
| 322 | Scherer-Neto P, Silva-Júnior A, Macedo LFF, Ramos FF, Klemann-Júnior L. Composição e distribuição da avifauna na área do Aterro Sanitário da Caximba, Região Metropolitana de Curitiba. Atualidades Orn. 2012;168:42-51 |
| 323 | Scherer-Neto P, Straube F. Notas sobre o status de alguns Suboscines raros no Estado do Paraná (Passeriformes, Aves). Anais do XV Congresso Brasileiro de Zoologia; 1988; Curitiba, BR. Sociedade Brasileira de Zoologia; 1988. p 501. |
| 324 | Scherer-Neto P, Straube FC. Aves do Paraná: história, lista anotada e bibliografia. Campo Largo: Logos Press; 1995. 79 p. |
| 325 | Scherer-Neto P, Straube FC, Bornschein MR. Composição avifaunística dos cerrados do Estado do Paraná: levantamento e conservação. Anais do I Congresso Brasileiro de Ornitologia; 1991 Jul 21-27. Belém, BR. Sociedade Brasileira de Ornitologia; 1991. p.15. |
| 326 | Scherer-Neto P, Straube FC, Carrano E, Urben-Filho A. Lista das aves do Paraná: edição comemorativa do Centenário da Ornitologia do Parana. Curitiba: Hori Consultoria Ambiental, 2011. 130p. |
| 327 | Scherer-Neto P, Terto AC, Carrano E. Ocorrência, ecologia e conservação de arara-vermelha-grande *Ara chloropterus* e arara-canindé *Ara ararauna* no estado do Paraná. Cadernos de Biodiversidade. 2009;6(2):22-29. |
| 328 | Scherer-Neto P, Terto AC, Ramos FF, Lima JAC. Ocorrência de grandes bandos de aves aquáticas e limícolas no noroeste do estado do Paraná. Atualidades Orn. 2009;148:7-9. |
| 329 | Scherer-Neto P, Toledo MCB. Avaliação populacional do papagaio-de-cara–roxa (*Amazona brasiliensis*) (Psittacidae) no estado do Paraná, Brasil. Ornitol Neotrop. 2007;18(3):379-393. |
| 330 | Schlichting MS, Bispo AA. Distribuição da avifauna em um remanescente de floresta com araucária no sul do Brasil. Anais do XVII Congresso Brasileiro de Ornitologia; 2009 Jun 28-Jul 03; Aracruz, BR. Sociedade Brasileira de Ornitologia; 2009. Resumos (Ecologia), EC231, p. 69. |
| 331 | Sedane JCS, Zanelatto RC, Bassfeld JC. 1992. Treating stray penguins *Spheniscus magellanicus* in subtropical environment. Anais do XIX Congresso Brasileiro de Zoologia; 1992 Jul 26-31; Belém, BR. Sociedade Brasileira de Zoologia; 1992. p.143. |
| 332 | Seger C, Lara AI, Arruda SD, Bóçon R, Antonelli-Filho R, Scherer-Neto P. Avifauna dos Refúgios Biológicos de Bela Vista e Santa Helena, Itaipu Binacional, oeste do Paraná. Anais do III Congresso Brasileiro de Ornitologia; 1993 Out 17-22. Pelotas, BR. Sociedade Brasileira de Ornitologia; 1993. Resumos P36. |
| 333 | Serafini PP, Anjos L, Arzua M, Volpato GH, Lopes EV, Poletto F. Primeiro registro de ectoparasitismo por *Ornithonyssus sylviarum* (Macronyssidae) em ninhegos de *Coragyps atratus* em um remanescente de Floresta Atlântica no sul do Brasil. Anais do XI Congresso Brasileiro de Ornitologia; 2003 Nov 23-28; Feira de Santana, BR. Sociedade Brasileira de Ornitologia; 2003. p 124. |
| 334 | Serafini PP, Anjos L, Volpato GH, Lopes EV, Favaro F, Poletto F. Tamanho médio de bandos para dez espécies de Psittacidae no norte do Estado do Paraná. Anais do XI Congresso Brasileiro de Ornitologia; 2003 Nov 23-28; Feira de Santana, BR. Sociedade Brasileira de Ornitologia; 2003. p 199. |
| 335 | Serafini PP, Mendonça LB, Anjos L, Volpato GH, Boçon R, Lopes EV, Favaro FL, Bisheimer MV. Monitoramento populacional de psitacídeos em unidades de conservação no estado do Paraná. Anais do XII Congresso Brasileiro de Ornitologia; 2004 Nov 21-26; Blumenau, BR. Sociedade Brasileira de Ornitologia; 2004. p 369. |
| 336 | Shibuya FLS. O ninho de joão-de-barro (*Furnarius rufus*) é uma câmara de incubação [dissertation]. Programa de Pós Graduação em Ecologia e Conservação: Universidade Federal do Paraná; 2012. |
| 337 | Sick H. Ornitologia Brasileira: uma Introdução. Brasília: Universidade de Brasília; 1985. Vol. 1 e 2. 827 p. |
| 338 | Silva CRS, Anjos L. Análise da abundância de aves associadas ao bambu em um remanescente de floresta estacional decidual no norte do estado do Paraná. Anais do XVI Congresso Brasileiro de Ornitologia; 2008 Jun 29-Jul 04; Palmas, BR. Sociedade Brasileira de Ornitologia; 2008. p 173. |
| 339 | Silva FCA. Ecologia alimentar de *Athene cunicularia* e *Tyto alba* (Aves, Strigiformes) na cidade de Curitiba e Região Metropolitana, Estado do Paraná [dissertation]. Programa de Pós Graduação em Ciências Biológicas-Zoologia: Universidade Federal do Paraná; 2006. |
| 340 | Silva GG, Guadagnin DL. Estimativa de densidade de *Zenaida auriculata* em áreas agrícolas no sul do Brasil e sua relação com a paisagem. Anais do XX Congresso Brasileiro de Ornitologia; 2013 Nov 04-07; Passo Fundo, BR. Sociedade Brasileira de Ornitologia; 2013. p 152-153. |
| 341 | Silva J, Serafini PP, Passos FC, Boçon R, Anjos L. Registros preliminares da abundância de Ramphastidae na Floresta Ombrófila Densa, APA de Guaraqueçaba – PR. Anais do XIV Congresso Brasileiro de Ornitologia (População e Comunidade); 2006 Jul 02-06; Ouro Preto, BR. Sociedade Brasileira de Ornitologia; 2006. R. 21, p. 24. |
| 342 | Silva JCA, Serafini PP, Passos FC, Boçon R, Anjos L. Abundância e densidade populacional de Ramphastidae na floresta ombrófila densa, APA de Guaraqueçaba – PR. Anais do XV Congresso Brasileiro de Ornitologia, Brasil; 2007 Jul 1-6; Porto Alegre, BR. Sociedade Brasileira de Ornitologia; 2007. PAINEL EC21, p 243-244. |
| 343 | Silva JCB, Andrade AL, Cândido-Junior JF. Avifauna aquática em balneários de três localidades do extremo oeste do estado do Paraná. Anais do XV Congresso Brasileiro de Ornitologia, Brasil; 2007 Jul 1-6; Porto Alegre, BR. Sociedade Brasileira de Ornitologia; 2007. PAINEL FA12, p 77-78. |
| 344 | Silva JVC, Bochio GM, McCrate G, Anjos L. Uma análise do efeito de borda em aves em um remanescente florestal no norte do Paraná. Anais do XV Congresso Brasileiro de Ornitologia, Brasil; 2007 Jul 1-6; Porto Alegre, BR. Sociedade Brasileira de Ornitologia; 2007. PAINEL CS25, p 219. |
| 345 | Silva SM, Souza WS. Contribuição ao conhecimento do desenvolvimento da coruja-das-torres *Tyto alba tuidara* (Gray, 1849) - Aves, Strigiformes. Anais da 38ª Reunião Anual da SBPC; 1986 Jul 09-16; Curitiba, BR. Sociedade Brasileira para o Progresso da Ciência; 1986. Resumos G.1.11 (49), p.1017. |
| 346 | Sipinski EAB, Abbud MC, Monteiro-Filho ELA. A influência das mudanças climáticas na biologia reprodutiva do papagaio-de-cara-roxa (*Amazona brasiliensis*). Anais do XX Congresso Brasileiro de Ornitologia; 2013 Nov 04-07; Passo Fundo, BR. Sociedade Brasileira de Ornitologia; 2013. p 207-208. |
| 347 | Sipinski EAB, Bóçon R. Estimativa populacional de *Amazona brasiliensis* (papagaio-de-cara-roxa) na área de ocorrência no estado do Paraná. Anais do XI Congresso Brasileiro de Ornitologia; 2003 Nov 23-28; Feira de Santana, BR. Sociedade Brasileira de Ornitologia; 2003. p 203. |
| 348 | Sipinski EAB, Bóçon R. Utilização de bromélias (*Vriesea gigantea* – Bromeliaceae) na nidificação de Amazona brasiliensis na Ilha Rasa, Paraná, Brasil. Anais do XV Congresso Brasileiro de Ornitologia, Brasil; 2007 Jul 1-6; Porto Alegre, BR. Sociedade Brasileira de Ornitologia; 2007. PAINEL BR18, p 112-113. |
| 349 | Sipinski EAB, Firkowski C, Roper J. Aspectos da reprodução do papagaio-de-cara-roxa (*Amazona brasiliensis*) na Ilha Rasa, litoral norte do Paraná. Anais do XI Congresso Brasileiro de Ornitologia; 2003 Nov 23-28; Feira de Santana, BR. Sociedade Brasileira de Ornitologia; 2003. p 15. |
| 350 | Sipinski EAB, Firkowski C, James R. Análise da predação de ninhos de *Amazona brasiliensis*, Ilha Rasa, litoral norte do Paraná, Brasil. Anais do XII Congresso Brasileiro de Ornitologia; 2004 Nov 21-26; Blumenau, BR. Sociedade Brasileira de Ornitologia; 2004. p 385. |
| 351 | Soares ES, Anjos L. 1999. Efeito da fragmentação florestal sobre aves escaladoras de tronco e galho na região de Londrina, norte do estado do Paraná, Brasil. Ornitol Neotrop. 1999;10(1):61-68. |
| 352 | Sobânia R, Sabóia J, Bispo AA, Dal'Maso A. Dois novos registros de *Accipiter poliogaster* para o Estado do Paraná, Brasil. Anais do XI Congresso Brasileiro de Ornitologia; 2003 Nov 23-28; Feira de Santana, BR. Sociedade Brasileira de Ornitologia; 2003. p 128. |
| 353 | Sobotka DD. Comportamento de casais e estudo de paternidade em uma parcela da população de bicudinhos-do-brejo (*Stymphalornis acutirostris* - Thamnophilidae - Aves), em Guaratuba, Paraná, Brasil [dissertation]. Programa de Pós Graduação em Ecologia e Conservação: Universidade Federal do Paraná; 2011. |
| 354 | Sobotka DD, Barbola IF, Bornschein MR. O uso da araucária (*Araucaria angustifolia*) para a nidificação de aves. Anais do XV Congresso Brasileiro de Ornitologia, Brasil; 2007 Jul 1-6; Porto Alegre, BR. Sociedade Brasileira de Ornitologia; 2007. PAINEL EC44, p 261. |
| 355 | Steffan K. Vogelleben am Agua do Quati (Brasilien). Gefied Welt. 1974;98(6):102-104. |
| 356 | Straube FC. Nova ocorrência de *Onychorhynchus coronatus swainsoni* (Pelzeln, 1858) para o Estado do Paraná. Anais da 38ª Reunião Anual da SBPC; 1986 Jul 09-16; Curitiba, BR. Sociedade Brasileira para o Progresso da Ciência; 1986. Resumos G.1.11 (39). |
| 357 | Straube FC. Contribuição ao conhecimento da avifauna da região sudoeste do Estado do Paraná (Brasil). Biotemas. 1988;1(1):63-75. |
| 358 | Straube FC. Notas bionômicas sobre *Conopophaga melanops* (Vieillot, 1818) no Estado do Paraná. Biotemas. 1989;2(1):91-95. |
| 359 | Straube FC. Sobre a distribuição geográfica de *Macropsalis creagra* (Bonaparte, 1850) no Estado do Paraná, Brasil. Sulórnis. 1989;10:12-21. |
| 360 | Straube FC. Conservação de aves no litoral-sul do Estado do Paraná (Brasil). Arq Biol Tecnol. 1990;33(1):159-173. |
| 361 | Straube FC. Notas sobre a distribuição de *Eleothreptus anomalus* (Gould, 1837) e *Caprimulgus longirostris longirostris* Bonaparte, 1825 no Brasil (Aves; Caprimulgidae). Acta Biol Leopold. 1990;12(2):301-312. |
| 362 | Straube FC. Novos registros de duas aves raras no Estado do Paraná: *Crypturellus noctivagus* e *Tigrisoma fasciatum*. Ararajuba. 1991;2:93-94. |
| 363 | Straube FC. Avifauna da Área Especial de Interesse Turístico do Marumbi (Paraná, Brasil). Atualidades Orn. 2003;113:12. |
| 364 | Straube FC. Avifauna da Fazenda Barra Mansa (Arapoti, Paraná), com anotações sobre a ocupação de monoculturas de essências arbóreas. Atualidades Orn. 2008;142:46-50. |
| 365 | Straube FC. Fontes históricas sobre a presença de araras no estado do Paraná. Atualidades Orn. 2010;156:64-87. |
| 366 | Straube FC, Aguiar MR, Lara AI. Ornitofauna de São Mateus do Sul, Paraná. Anais do XIV Congresso Brasileiro de Zoologia; 1987 Feb 01-06; Juiz de Fora, BR. Sociedade Brasileira de Zoologia; 1987. p 151. |
| 367 | Straube FC, Aguiar MR, Meijer AAR. Composição ornitofaunística da Área Especial de Interesse Turístico do Marumbi (Serra do Mar, Paraná). Anais do XV Congresso Brasileiro de Zoologia; 1988; Curitiba, BR.Sociedade Brasileira de Zoologia; 1988. p 493. |
| 368 | Straube FC, Arruda SD. Coletânea da avifauna da porção sul do estado do Paraná. Anais do I Congresso Brasileiro de Ornitologia; 1991 Jul 21-27. Belém, BR. Sociedade Brasileira de Ornitologia; 1991. p 21. |
| 369 | Straube FC, Bornschein MR. A contribuição de André Mayer à História Natural no Paraná. I. Sobre uma coleção de aves do extremo noroeste do Paraná e sul do Mato Grosso do Sul. Arq Biol Tecnol. 1989;32(2):441-471. |
| 370 | Straube FC, Bornschein MR. *Cranioleuca obsoleta siemiradzkii* Sztolcman, 1926: um jovem de *Cranioleuca pallida* (Wied, 1831). Anais do I Congresso Brasileiro de Ornitologia; 1991 Jul 21-27. Belém, BR. Sociedade Brasileira de Ornitologia; 1991. p.32. |
| 371 | Straube FC, Bornschein MR. Novos registros de *Chloroceryle inda* (Linnaeus, 1766) e *Chloroceryle aenea* (Pallas, 1764) para o Estado do Paraná, sul do Brasil (Alcedinidae, Aves). Acta Biol Leopold. 1991;13(1):81-84. |
| 372 | Straube FC, Bornschein MR. Novos registros de *Puffinus gravis* (O'Reilly, 1818) na costa brasileira (Procellariidae). Anais do I Congresso Brasileiro de Ornitologia; 1991 Jul 21-27. Belém, BR. Sociedade Brasileira de Ornitologia; 1991. p.32. |
| 373 | Straube FC, Bornschein MR. *Synallaxis hypospodia* Sclater, 1874: aspectos da bionomia e sucessão de plumagem. Anais do I Congresso Brasileiro de Ornitologia; 1991 Jul 21-27. Belém, BR. Sociedade Brasileira de Ornitologia; 1991. p.11. |
| 374 | Straube FC, Bornschein MR. News or noteworthy records of birds from northwestern Parana and adjacent areas. Bull Br Orn Club. 1995;115(4):219-225. |
| 375 | Straube FC, Bornschein MR, Scherer-Neto P. Coletânea da avifauna da região noroeste do Estado do Paraná e áreas limítrofes (Brasil). Arq Biol Tecnol. 1996;39(1):193-214. |
| 376 | Straube FC, Bornschein MR, Teixeira DM. Nova ocorrência de *Vultur gryphus* em território brasileiro. Anais do I Congresso Brasileiro de Ornitologia; 1991 Jul 21-27. Belém, BR. Sociedade Brasileira de Ornitologia; 1991. p.31. |
| 377 | Straube FC, Bornschein MR, Teixeira DM. The nest of large-billed antwren *Herpsilochmus longirostris*. Bull Br Orn Club. 1992;112(4):277-279. |
| 378 | Straube FC, Carrano E, Santos REF, Scherer-Neto P, Ribas CF, Meijer AAR, Vallejos MAV, Lanzer M, Klemann-Junior L, Aurelio-Silva M, Urben-Filho A, Arzua M, Lima AMX, Sobânia RLM, Deconto LR, Bispo AÂ, Jesus S, Abilhôa V. Aves de Curitiba: coletânea de registros. 2ª Edição. Curitiba: Hori Consultoria Ambiental; 2014. 527 p. |
| 379 | Straube FC, Krul R, Carrano E. Coletânea da avifauna da região sul do estado do Paraná (Brasil). Atualidades Orn. 2005;125: 10. |
| 380 | Straube FC, Scherer-Neto P. Novas observações sobre o cunhataí *Triclaria malachitacea* (Spix, 1824) nos Estados do Paraná e São Paulo (Psittacidae, Aves). Acta Biol Leopold. 1995;17(1):147-152. |
| 381 | Straube FC, Urben-Filho A. Uma revisão crítica sobre o grau de conhecimento da avifauna do Parque Nacional do Iguaçu (Paraná, Brasil) e áreas adjacentes. Atualidades Orn. 2004;118:6. |
| 382 | Straube FC, Urben-Filho A. Avifauna da Reserva Natural Salto Morato (Guaraqueçaba, Paraná). Atualidades Orn. 2005;124: 10. |
| 383 | Straube FC, Urben-Filho A. Observações sobre a avifauna de pequenos remanescentes florestais na região noroeste do Paraná (Brasil). Atualidades Orn. 2005;123: 10. |
| 384 | Straube FC, Urben-Filho A. Lista de campo: Aves da Reserva Natural Salto Morato (Guaraqueçaba, Paraná); Checklist: Birds of Reserva Natural Salto Morato (Guaraqueçaba, Paraná). Curitiba: Fundação O Boticário de Proteção à Natureza; 2007. |
| 385 | Straube FC, Urben-Filho A. Notas sobre a avifauna de nove localidades na Bacia do Rio Piquiri (Região Oeste do Paraná, Brasil). Atualidades Orn. 2008;142:33-37. |
| 386 | Straube FC, Urben-Filho A. Comentários e retificações sobre o único registro de *Morphnus guianensis* (Accipitriformes Accipitridae) para o Paraná. Atualidades Orn. 2010;157:4-6. |
| 387 | Straube FC, Urben-Filho A, Cândido-Junior JF. Novas informações sobre a avifauna do Parque Nacional do Iguaçu (Paraná). Atualidades Orn. 2004;120:10. |
| 388 | Straube FC, Urben-Filho A, Deconto LR, Patrial EW. *Fluvicola nengeta* (Linnaeus, 1766) nos estados do Paraná e Mato Grosso do Sul e sua expansão de distribuição geográfica pelo sul do Brasil. Atualidades Orn. 2007;138:29. |
| 389 | Straube FC, Urben-Filho A, Gatto C. A avifauna do parque estadual do Cerrado (Jaguariaíva, Paraná) e a conservação do cerrado em seu limite meridional de ocorrência. Atualidades Orn. 2005;127:29. |
| 390 | Straube FC, Urben-Filho A, Piacentini VQ. O beija-flor-tesoura *Eupetomena macroura* (Gmelin, 1788) e sua ampliação de distribuição pelo sul do Brasil. Atualidades Orn. 2006;132: 29. |
| 391 | Straube FC, Vallejos MAV, Deconto LR, Urben Filho A. IPAVE – 2012: inventário participativo das aves do Paraná. Curitiba: Hori Consultoria Ambiental; 2013. 221 p. |
| 392 | Straube FC, Willis EO, Oniki Y. Aves colecionadas na localidade de Fazenda Caiuá (Paraná, Brasil) por Adolph Hempel, com discussão sobre a sua localização exata. Ararajuba. 2002;10(2):167-172. |
| 393 | Sztolcman J. Étude des collections ornithologiques de Paraná. Ann Zool Mus Polon Hist Nat. 1926;5:107-196. |
| 394 | Sztolcman, J. Revision des oiseaux neotropicaux de la collection du Musée Plonais d'Histoire Naturelle à Varsovie. I. Ann Zool Mus Polon Hist Nat. 1926;5(4):197-234 |
| 395 | Teixeira TAB. Nidificação da saíra-viúva *Pipraeidea melanonota* (Passeriformes: Thraupidae) na região dos Campos Gerais – PR. Atualidades Orn. 2009;152:12-13. |
| 396 | Terto AC, Ramos FF, Lima JAC, Scherer-Neto P, Candido J. Um ninho de tuiuiu *Jabiru mycteria* no noroeste do Paraná. Atualidades Orn. 2008;146:20-21. |
| 397 | Tiepolo LM, Milléo-Costa LC. Estudo do comportamento de *Furnarius rufus* Gmelin, 1788 (Aves - Passeriformes - Furnariidae) em Curitiba, Paraná, Brasil. Atualidades Orn. 1998;82:5. |
| 398 | Torre GM, Carrano E. Estimativa populacional de arapaçus (Dendrocolaptidae) na Floresta Estadual do Palmito, Paranaguá, Paraná. Anais do XVII Congresso Brasileiro de Ornitologia; 2009 Jun 28-Jul 03; Aracruz, BR. Sociedade Brasileira de Ornitologia; 2009. Resumos (Ecologia), EC147, p. 48. |
| 399 | Tossulino MP, Scherer-Neto P. Análise do impacto ambiental sobre a avifauna na Represa do Rio Passaúna. Anais do I Congresso Brasileiro de Ornitologia; 1991 Jul 21-27. Belém, BR. Sociedade Brasileira de Ornitologia; 1991. p. 12. |
| 400 | Uejima AMK, Bornschein MR. As aves dos Campos Gerais. In: Melo MS, Moro RS, Guimarães GB, editors. Patrimônio Natural dos Campos Gerais do Paraná. Ponta Grossa, UEPG; 2007. p. 109-121. |
| 401 | Uejima A, Roper JJ, Sabóia J, Monteiro T, Neidfeld M. Efeito do tamanho do fragmento na predação em ninhos artificiais no Parque Estadual de Vila Velha, Ponta Grossa, Paraná. Anais do XI Congresso Brasileiro de Ornitologia; 2003 Nov 23-28; Feira de Santana, BR. Sociedade Brasileira de Ornitologia; 2003. p 209. |
| 402 | Urben-Filho A, Abe LM. Inventário preliminar da avifauna da Fazenda Primavera (Adrianópolis, Paraná). In: Straube FC, editor. Ornitologia sem fronteiras, incluindo os Resumos do IX Congresso Brasileiro de Ornitologia; 2001 Jul 22-27; Curitiba, BR. Fundação O Boticário de Proteção à Natureza; 2001. R216, p 263-264. |
| 403 | Urben-Filho A, Straube FC, Gatto CAFR. Primeiro registro de *Chamaeza meruloides* para o Paraná. In: Straube FC, editor. Ornitologia sem fronteiras, incluindo os Resumos do IX Congresso Brasileiro de Ornitologia; 2001 Jul 22-27; Curitiba, BR. Fundação O Boticário de Proteção à Natureza; 2001. R217, p 381-382. |
| 404 | Valle AO, Sipinski EAB, Bóçon R. Informações sobre a alimentação de *Amazona brasiliensis* (papagaio-de-cara-roxa), (Linnaeus, 1758) na região de Guaraqueçaba, Paraná, Brasil. Anais do XII Congresso Brasileiro de Ornitologia; 2004 Nov 21-26; Blumenau, BR. Sociedade Brasileira de Ornitologia; 2004. p 403. |
| 405 | Vallejos MAV. O papel da heterogeneidade espacial na manutenção da riqueza avifaunística em uma área úmida em Curitiba [monograph]. Curso de Ciências Biológicas: Universidade Federal do Paraná; 2007. |
| 406 | Vallejos MAV, Deconto LR. Ocorrências extra-litorâneas de chimango *Milvago chimango* (Falconiformes: Falconidae) no Paraná. Atualidades Orn. 2009;152:39-40. |
| 407 | Vallejos MAV, Lanzer M, Aurélio-Silva M. Observações sobre um ninho de gavião-relógio *Micrastur semitorquatus* (Vieillot, 1817) no sul do Brasil. Anais do XVI Congresso Brasileiro de Ornitologia; 2008 Jun 29-Jul 04; Palmas, BR. Sociedade Brasileira de Ornitologia; 2008. p 60. |
| 408 | Vallejos MAV, Lanzer M, Aurélio-Silva M, Silva-da-Rocha LF. Nidificação de gavião-relógio *Micrastur semitorquatus* (Vieillot, 1817) em uma gruta no Sul do Brasil. Rev Bras Ornitol. 2008;16(3):268-270 |
| 409 | Vallejos MAV, Molin T, Vieira-da-Rocha MC, Deconto LR, Floriano-Cunha R, Sobânia RLM, Scherer-Neto P. Registro documentado da marreca-colhereira *Anas platalea* (Anseriformes: Anatidae) no Estado do Paraná. Atualidades Orn. 2009;151:20-21. |
| 410 | Vargas JJ, Whitacre D, Mosquera R, Albuquerque J, Piana R, Thiollay J, Márquez C, Sánchez JE, Lezama-Lopes M, Midence S, Matola S, Aguilar S, Rettig N, Sanaiotti T. Estado y distribución actual del águila arpía (*Harpia harpyja*) en Centro y Sur América. Ornitol Neotrop. 2006;17(1):39-55. |
| 411 | Veiga LA, Pardo E. Ocorrência de um caso de albinismo em sabiá laranjeira *Turdus rufiventris*. Anais do XVII Congresso Brasileiro de Zoologia; 1990 Jan 28-Fev 02; Londrina, BR. Sociedade Brasileira de Zoologia; 1990. p. 169. |
| 412 | Veiga LA, Pardo E. Ocorrência de um caso de albinismo em sabiá laranjeira. Arq Biol Tecnol. 1990;33(2):329-333. |
| 413 | Vogel HF, Zawadzki CH, Metri R. Coexistência entre *Turdus leucomelas* Vieillot, 1818 e *Turdus rufiventris* Vieillot, 1818 (Aves: Passeriformes) em um fragmento urbano de Floresta com Araucárias, Sul do Brasil. Biota Neotropica. 2011;11(3):35-45. |
| 414 | Volpato GH. Comunidades de aves em mosaico de habitat formado por Floresta Ombrófila Mista e plantações com *Araucaria angustifolia* e com *Pinus elliotti* no sul do estado do Paraná, Brasil [thesis]. Programa de Pós Graduação em Ciências Biológicas – Zoologia: Universidade Federal do Paraná; 2009. |
| 415 | Volpato GH, Anjos L. Análise das estratégias de forrageamento das aves que se alimentam no solo na Universidade Estadual de Londrina, Estado do Paraná. Ararajuba. 2001;9(2):95-99 |
| 416 | Volpato GH, Anjos L, Lopes EV, Fávaro FL, Mendonça LB. Abundância e distribuição da família Dendrocolaptidae na Floresta Atlântica da bacia hidrográfica do Rio Tibagi, Paraná, Brasil. Anais do XII Congresso Brasileiro de Ornitologia; 2004 Nov 21-26; Blumenau, BR. Sociedade Brasileira de Ornitologia; 2004. p 412. |
| 417 | Volpato GH, Anjos L, Mendonça LB, Loures-Ribeiro A, Gimenez MR, Lopes EV. Análise das estratégias de forrageamento das aves que se alimentam no solo na Universidade Estadual de Londrina, Paraná. In: Straube FC, editor. Ornitologia sem fronteiras, incluindo os Resumos do IX Congresso Brasileiro de Ornitologia; 2001 Jul 22-27; Curitiba, BR. Fundação O Boticário de Proteção à Natureza; 2001. R228, p 279. |
| 418 | Wasilewski M, Silva FC, Nascimento M, Scherer-Neto P. Ocorrência e registro documentado de guará *Eudocimus ruber* (Threskionithidae, Aves) na Baía de Guaratuba, Paraná. Atualidades Orn. 2008;145:10-11. |
| 419 | Westcott PW. Descrição das aves encontradas na área urbana de Londrina - Paraná. Primeira parte - espécies não Passeriformes. Semina. 1980;6(2):59-66. |
| 420 | Westcott PW. Kleptoparasitism and territoriality by *Myiornis auricularis* (Passeriformes, Tyrannidae). Anais do XII Congresso Brasileiro de Zoologia; 1985 Jan 27-Fev 01; Campinas, BR. Sociedade Brasileira de Zoologia; 1985. p. 282. |
| 421 | Westcott PW. Flutuação populacional de beija-flores (Aves, Trochilidae) na região de Londrina - Pr. Anais do XIII Congresso Brasileiro de Zoologia; 1986; Cuiabá, BR. Sociedade Brasileira de Zoologia; 1986. Resumo 691, p. 244. |
