# Supplementary material for: Traditional scientific data vs. uncoordinated citizen science effort: A review of the current status and comparison of data on avifauna in Southern Brazil
Source: PLoS One. 2017 Dec 11;12(12):e0188819. doi: 10.1371/journal.pone.0188819 (PMC5724844; doi:10.1371/journal.pone.0188819)
Supplement: S3 Table — Notes: I–introduced non-native species with recently estabilished populations in the state; C–recently-colonizing species; N–species included in our primary and secondary lists but not mentioned in Scherer-Neto et al. [17]; N(S)–species included in our primary and secondary lists, and mentioned in the terciary list of Scherer-Neto et al. [17]; SP–species transferred from the secondary list of Scherer-Neto et al. [17] to our primary list. Environments and Vegetation types: MAR–Seabirds; EGL–Grassland; FES–Semideciduous Tropical Forest; FOD–Tropical Rainforest; FOM–Araucaria Moist Forest; SA–Savanna. Source: WA–Wiki Aves database. Nomenclature and taxonomic order follow CBRO [35]. (DOCX) [file pone.0188819.s003.docx]

**S3 Table**. Primary and secondary (within square brackets) lists of species with records in Paraná state, indicating the number of records in each vegetation type (see also S1 Table and S1 Table). Notes: I – introduced non-native species with recently estabilished populations in the state; C – recently-colonizing species; N – species included in our primary and secondary lists but not mentioned in Scherer-Neto et al. [17]; N(S) – species included in our primary and secondary lists, and mentioned in the terciary list of Scherer-Neto et al. [17]; SP – species transferred from the secondary list of Scherer-Neto et al. [17] to our primary list. Environments and Vegetation types: MAR – Seabirds; EGL – Grassland; FES – Semideciduous Tropical Forest; FOD – Tropical Rainforest; FOM – Araucaria Moist Forest; SA – Savanna. Source: WA – Wiki Aves database. Nomenclature and taxonomic order follow CBRO [35].

| **Taxon name** | **MAR** | **EGL** | **FES** | **FOD** | **FOM** | **SA** | **Total** | **Notes** | **Source (see S1 Table and S2 Table)** |
| --- | --- | --- | --- | --- | --- | --- | --- | --- | --- |
| Rheiformes Forbes, 1884 |  |  |  |  |  |  |  |  |  |
| Rheidae Bonaparte, 1849 |  |  |  |  |  |  |  |  |  |
| *Rhea americana* (Linnaeus, 1758) |  |  | 8 |  |  |  | **8** | **C** | 310, 391, WA |
| Tinamiformes Huxley, 1872 |  |  |  |  |  |  |  |  |  |
| Tinamidae Gray, 1840 |  |  |  |  |  |  |  |  |  |
| *Tinamus solitarius* (Vieillot, 1819) |  | 4 | 17 | 35 | 4 |  | **60** |  | 13, 14, 19, 21, 41, 47, 52, 81, 173, 313, 357, 360, 363, 369, 379, 382, 391, 392, 402, MHNCI, MZUSP, WA |
| *Crypturellus obsoletus* (Temminck, 1815) |  | 40 | 45 | 31 | 105 | 8 | **229** |  | 5, 13, 14, 17, 19, 21, 46, 47, 59, 81, 87, 94, 134, 137, 150, 194, 205, 206, 258, 294, 304, 305, 306, 309, 313, 316, 357, 360, 363, 364, 378, 379, 382, 383, 385, 387, 389, 391, 393, 414, FMNH, MHNCI, WA |
| *Crypturellus undulatus* (Temminck, 1815) |  |  | 6 |  |  |  | **6** |  | 13, 14, 21, 264, 369, MHNCI, MZUSP |
| *Crypturellus noctivagus* (Wied, 1820) |  |  |  | 28 |  |  | **28** |  | 47, 360, 362, 382, 391, MHNCI, WA |
| *Crypturellus parvirostris* (Wagler, 1827) |  | 5 | 55 | 4 | 21 | 7 | **92** |  | 13, 14, 21, 22, 23, 59, 155, 181, 258, 294, 306, 309, 313, 357, 363, 364, 375, 378, 379, 383, 385, 387, 389, 391, MHNCI, MZUSP, WA |
| *Crypturellus tataupa* (Temminck, 1815) |  | 6 | 82 | 13 | 17 | 2 | **120** |  | 13, 14, 21, 22, 23, 47, 59, 81, 134, 181, 258, 306, 309, 313, 316, 357, 363, 364, 369, 375, 378, 379, 382, 383, 385, 387, 389, 391, 393, MHNCI, WA |
| *Rhynchotus rufescens* (Temminck, 1815) |  | 30 | 60 |  | 61 | 8 | **159** |  | 19, 21, 22, 23, 94, 138, 150, 194, 205, 267, 305, 306, 309, 316, 357, 364, 375, 378, 379, 383, 385, 389, 391, MHNCI, MZUSP, WA |
| *Nothura maculosa* (Temminck, 1815) |  | 53 | 63 |  | 70 | 4 | **190** |  | 4, 19, 21, 22, 23, 59, 134, 181, 250, 267, 294, 305, 309, 316, 357, 364, 375, 378, 379, 383, 385, 389, 391, 419, MHNCI, MZUEL, MZUSP, WA |
| *Taoniscus nanus* (Temminck, 1815) |  | 1 |  |  |  |  | **1** |  | 250 |
| Anseriformes Linnaeus, 1758 |  |  |  |  |  |  |  |  |  |
| Anhimidae Stejneger, 1885 |  |  |  |  |  |  |  |  |  |
| *Anhima cornuta* (Linnaeus, 1766) |  |  | 14 |  |  |  | **14** |  | 244, 264, 369, MHNCI, MZUSP, WA |
| *Chauna torquata* (Oken, 1816) |  |  |  | 2 |  |  | **2** |  | 324, 360 |
| Anatidae Leach, 1820 |  |  |  |  |  |  |  |  |  |
| Dendrocygninae Reichenbach, 1850 |  |  |  |  |  |  |  |  |  |
| *Dendrocygna bicolor* (Vieillot, 1816) |  | 27 | 7 | 5 | 21 | 1 | **61** |  | 62, 81, 294, 312, 328, 375, 378, 391, 405, MHNCI, WA |
| *Dendrocygna viduata* (Linnaeus, 1766) |  | 119 | 143 | 6 | 99 | 8 | **375** |  | 21, 81, 87, 120, 181, 206, 306, 309, 316, 322, 328, 364, 375, 378, 379, 383, 389, 391, 405, 419, MHNCI, WA |
| *Dendrocygna autumnalis* (Linnaeus, 1758) |  | 7 | 19 | 1 |  | 4 | **31** |  | 268, 324, 328, WA |
| Anserinae Vigors, 1825 |  |  |  |  |  |  |  |  |  |
| *Coscoroba coscoroba* (Molina, 1782) |  | 17 |  | 4 | 1 |  | **22** |  | 65, 124, 378, MHNCI, WA |
| Anatinae Leach, 1820 |  |  |  |  |  |  |  |  |  |
| *Cairina moschata* (Linnaeus, 1758) |  | 59 | 70 | 21 | 62 | 1 | **213** |  | 19, 21, 23, 81, 94, 120, 138, 207, 264, 268, 294, 306, 313, 316, 357, 360, 363, 369, 375, 378, 379, 382, 383, 387, 391, 393, 405, MHNCI, MZUSP, WA |
| *Sarkidiornis sylvicola* Ihering & Ihering, 1907 |  | 2 | 3 | 1 | 4 |  | **10** |  | 21, 81, 136, 312, 328, 378, WA |
| *Callonetta leucophrys* (Vieillot, 1816) |  | 1 |  |  | 3 |  | **4** |  | 378, WA |
| *Amazonetta brasiliensis* (Gmelin, 1789) |  | 310 | 143 | 24 | 240 | 9 | **726** |  | 19, 21, 23, 46, 59, 120, 137, 150, 161, 171, 181, 194, 246, 247, 267, 294, 305, 306, 309, 313, 316, 322, 328, 357, 360, 363, 364, 375, 378, 379, 382, 383, 385, 387, 389, 391, 393, 399, 405, 419, MHNCI, MZUEL, WA |
| *Anas flavirostris* Vieillot, 1816 |  | 4 |  |  | 3 |  | **7** | **SP** | 312, 378, 379, WA |
| *Anas georgica* Gmelin, 1789 |  | 13 |  | 1 | 13 |  | **27** |  | 309, 324, 378, 379, 391, MHNCI, WA |
| *Anas bahamensis* Linnaeus, 1758 |  | 53 | 2 | 6 | 16 | 7 | **84** |  | 207, 324, 378, 391, 405, WA |
| *Anas versicolor* Vieillot, 1816 |  | 118 | 2 |  | 27 |  | **147** |  | 57, 316, 378, 391, 405, MHNCI, WA |
| [*Anas discors*] Linnaeus, 1766 |  |  |  |  | 1 |  | **1** |  | 378 |
| *Anas platalea* Vieillot, 1816 |  | 2 |  |  | 2 |  | **4** |  | 378, 409, MHNCI, WA |
| *Netta erythrophthalma* (Wied, 1832) |  | 4 | 1 |  | 2 |  | **7** |  | 166, 378, WA |
| *Netta peposaca* (Vieillot, 1816) |  | 53 | 5 |  | 31 |  | **89** |  | 166, 312, 378, 391, 405, MHNCI, WA |
| *Mergus octosetaceus* Vieillot, 1817 |  |  | 3 |  | 1 |  | **4** |  | 21, 138, 392, 393 |
| *Nomonyx dominica* (Linnaeus, 1766) |  |  | 5 | 3 | 11 |  | **19** |  | 21, 94, 363, 369, 378, 391, MHNCI, WA |
| *Oxyura vittata* (Philippi, 1860) |  | 10 |  |  |  |  | **10** | **N(S)** | 378, WA |
| Galliformes Linnaeus, 1758 |  |  |  |  |  |  |  |  |  |
| Cracidae Rafinesque, 1815 |  |  |  |  |  |  |  |  |  |
| *Penelope superciliaris* Temminck, 1815 |  |  | 99 | 7 | 19 |  | **125** |  | 13, 14, 21, 25, 42, 47, 59, 73, 81, 83, 85, 118, 134, 155, 206, 211, 267, 294, 306, 313, 357, 369, 375, 379, 383, 385, 387, 391, 392, 393, MHNCI, MZUSP, WA |
| *Penelope obscura* Temminck, 1815 |  | 189 | 39 | 91 | 170 | 4 | **493** |  | 5, 14, 17, 19, 21, 47, 59, 87, 134, 137, 138, 150, 157, 176, 194, 205, 258, 284, 305, 309, 316, 357, 360, 363, 364, 367, 369, 375, 378, 379, 382, 385, 387, 389, 391, 393, 414, FMNH, MHNCI, MZUEL, MZUSP, WA |
| *Aburria jacutinga* (Spix, 1825) |  |  | 23 | 34 | 11 |  | **68** |  | 13, 14, 21, 41, 47, 52, 101, 138, 357, 368, 369, 379, 382, 387, 391, 393, 402, FMNH, MHNCI, MZUSP, NMNH, WA |
| *Ortalis squamata* (Lesson, 1829) |  |  |  | 75 |  |  | **75** |  | 47, 134, 137, 360, 382, 391, MHNCI, WA |
| *Crax fasciolata* Spix, 1825 |  |  | 7 |  |  |  | **7** |  | 13, 14, 21, 64, 313, 369, MHNCI, MZUSP |
| Odontophoridae Gould, 1844 |  |  |  |  |  |  |  |  |  |
| *Odontophorus capueira* (Spix, 1825) |  | 18 | 19 | 31 | 39 |  | **107** |  | 5, 13, 14, 17, 19, 21, 22, 47, 81, 85, 134, 205, 219, 294, 306, 309, 313, 316, 357, 360, 363, 364, 369, 375, 378, 379, 382, 385, 391, 393, 414, MHNCI, MZUSP, WA |
| Podicipediformes Fürbringer, 1888 |  |  |  |  |  |  |  |  |  |
| Podicipedidae Bonaparte, 1831 |  |  |  |  |  |  |  |  |  |
| *Rollandia rolland* (Quoy & Gaimard, 1824) |  |  | 1 |  | 1 |  | **2** |  | 324, 378, MHNCI |
| *Tachybaptus dominicus* (Linnaeus, 1766) |  | 29 | 69 | 4 | 53 | 2 | **157** |  | 19, 21, 59, 134, 155, 309, 316, 322, 363, 369, 375, 378, 379, 383, 391, 393, 405, 419, MHNCI, WA |
| *Podilymbus podiceps* (Linnaeus, 1758) |  | 89 | 23 | 6 | 94 |  | **212** |  | 21, 46, 306, 309, 316, 360, 369, 375, 378, 379, 387, 391, 405, 419, MHNCI, WA |
| *Podicephorus major* (Boddaert, 1783) |  |  |  | 4 |  |  | **4** |  | 324, WA |
| *Podiceps occipitalis* Garnot, 1826 |  | 1 |  |  | 9 |  | **10** |  | 57, 378, MHNCI, WA |
| Phoenicopteriformes Fürbringer, 1888 |  |  |  |  |  |  |  |  |  |
| Phoenicopteridae Bonaparte, 1831 |  |  |  |  |  |  |  |  |  |
| *Phoenicopterus chilensis* Molina, 1782 |  |  |  | 1 |  |  | **1** |  | WA |
| *Phoenicoparrus andinus* (Philippi, 1854) |  |  |  | 2 |  |  | **2** |  | 89, MHNCI |
| Sphenisciformes Sharpe, 1891 |  |  |  |  |  |  |  |  |  |
| Spheniscidae Bonaparte, 1831 |  |  |  |  |  |  |  |  |  |
| *Spheniscus magellanicus* (Forster, 1781) | 21 |  |  |  |  |  | **21** |  | 219, 226, 248, 271, 331, 391, MHNCI, MZUEL, WA |
| Procellariiformes Fürbringer, 1888 |  |  |  |  |  |  |  |  |  |
| Diomedeidae Gray, 1840 |  |  |  |  |  |  |  |  |  |
| *Thalassarche chlororhynchos* (Gmelin, 1789) | 6 |  |  |  |  |  | **6** |  | 62, 226, 324 |
| *Thalassarche melanophris* (Temminck, 1828) | 5 |  |  |  |  |  | **5** |  | 62, 271, WA |
| [*Thalassarche chrysostoma*] (Forster, 1785) | 1 |  |  |  |  |  | **1** |  | 324 |
| *Thalassarche cauta* (Gould, 1841) | 1 |  |  |  |  |  | **1** |  | 326 |
| [*Diomedea exulans*] Linnaeus, 1758 | 1 |  |  |  |  |  | **1** |  | 117 |
| Procellariidae Leach, 1820 |  |  |  |  |  |  |  |  |  |
| *Macronectes giganteus* (Gmelin, 1789) | 4 |  |  |  |  |  | **4** |  | 226, 271, MHNCI |
| [*Macronectes halli*] Mathews, 1912 | 2 |  |  |  |  |  | **2** |  | 229, 391 |
| *Fulmarus glacialoides* (Smith, 1840) | 5 |  |  |  |  |  | **5** |  | 226, MHNCI |
| *Daption capense* (Linnaeus, 1758) | 1 |  |  |  |  |  | **1** |  | 326 |
| [*Pterodroma mollis*] (Gould, 1844) | 2 |  |  |  |  |  | **2** |  | 229, 326 |
| [*Pterodroma incerta*] (Schlegel, 1863) | 6 |  |  |  |  |  | **6** |  | 324, 326 |
| [*Pachyptila vittata*] (Forster, 1777) | 1 |  |  |  |  |  | **1** |  | 229 |
| *Pachyptila belcheri* (Mathews, 1912) | 1 |  |  |  |  |  | **1** |  | MHNCI |
| *Procellaria aequinoctialis* Linnaeus, 1758 | 8 |  |  |  |  |  | **8** |  | 226, 271, 391, MHNCI, WA |
| *Procellaria conspicillata* Gould, 1844 | 9 |  |  |  |  |  | **9** | **SP** | 242, WA |
| *Calonectris borealis* (Cory, 1881) | 1 |  |  |  |  |  | **1** | **N** | WA |
| *Calonectris edwardsii* (Oustalet, 1883) | 2 |  |  |  |  |  | **2** | **SP** | 226, WA |
| [*Puffinus griseus*] (Gmelin, 1789) | 1 |  |  |  |  |  | **1** |  | 225 |
| *Puffinus gravis* (O'Reilly, 1818) | 2 |  |  |  |  |  | **2** |  | 271, 372 |
| *Puffinus puffinus* (Brünnich, 1764) | 4 |  |  |  |  |  | **4** |  | 226, 271, 391, MHNCI |
| [*Puffinus lherminieri*] Lesson, 1839 | 2 |  |  |  |  |  | **2** |  | 326 |
| Hydrobatidae Mathews, 1912 |  |  |  |  |  |  |  |  |  |
| Oceanitinae Forbes, 1882 |  |  |  |  |  |  |  |  |  |
| *Oceanites oceanicus* (Kuhl, 1820) | 1 |  |  |  |  |  | **1** |  | MHNCI |
| Ciconiiformes Bonaparte, 1854 |  |  |  |  |  |  |  |  |  |
| Ciconiidae Sundevall, 1836 |  |  |  |  |  |  |  |  |  |
| *Ciconia maguari* (Gmelin, 1789) |  |  | 17 |  | 3 |  | **20** |  | 120, 121, 122, 328, 375, 379, WA |
| *Jabiru mycteria* (Lichtenstein, 1819) |  |  | 15 |  |  | 1 | **16** |  | 120, 121, 122, 324, 328, 396, WA |
| *Mycteria americana* Linnaeus, 1758 |  | 12 | 41 |  | 10 | 4 | **67** |  | 21, 120, 121, 122, 312, 316, 328, 375, 391, WA |
| Suliformes Sharpe, 1891 |  |  |  |  |  |  |  |  |  |
| Fregatidae Degland & Gerbe, 1867 |  |  |  |  |  |  |  |  |  |
| *Fregata magnificens* Mathews, 1914 | 145 |  |  |  |  |  | **145** |  | 74, 76, 77, 78, 79, 81, 131, 136, 137, 158, 219, 226, 230, 250, 297, 302, 360, 391, MHNCI, WA |
| Sulidae Reichenbach, 1849 |  |  |  |  |  |  |  |  |  |
| [*Sula dactylatra*] Lesson, 1831 | 1 |  |  |  |  |  | **1** |  | 229 |
| *Sula leucogaster* (Boddaert, 1783) | 140 |  |  |  |  |  | **140** |  | 70, 74, 78, 79, 117, 131, 136, 137, 158, 200, 217, 219, 226, 230, 250, 271, 297, 360, 391, MHNCI, WA |
| Phalacrocoracidae Reichenbach, 1849 |  |  |  |  |  |  |  |  |  |
| *Phalacrocorax brasilianus* (Gmelin, 1789) |  | 238 | 184 | 138 | 236 |  | **796** |  | 3, 19, 21, 23, 54, 74, 76, 77, 78, 79, 81, 94, 120, 134, 137, 138, 155, 161, 171, 181, 189, 198, 207, 219, 226, 230, 237, 238, 239, 240, 250, 306, 309, 313, 316, 322, 343, 357, 360, 363, 364, 369, 375, 378, 379, 382, 383, 385, 387, 391, 399, 405, AMNH, MHNCI, WA |
| Anhingidae Reichenbach, 1849 |  |  |  |  |  |  |  |  |  |
| *Anhinga anhinga* (Linnaeus, 1766) |  | 15 | 44 | 5 | 13 |  | **77** |  | 117, 120, 138, 306, 313, 357, 360, 375, 378, 387, 391, 393, WA |
| Pelecaniformes Sharpe, 1891 |  |  |  |  |  |  |  |  |  |
| Ardeidae Leach, 1820 |  |  |  |  |  |  |  |  |  |
| *Tigrisoma lineatum* (Boddaert, 1783) |  | 3 | 53 | 17 | 12 |  | **85** |  | 21, 22, 120, 121, 122, 137, 250, 268, 306, 313, 363, 369, 378, 382, 383, 387, 391, MHNCI, MZUSP, WA |
| *Tigrisoma fasciatum* (Such, 1825) |  |  | 5 | 11 |  |  | **16** |  | 362, 375, 387, 391, 393, WA |
| *Cochlearius cochlearius* (Linnaeus, 1766) |  |  | 2 | 2 |  |  | **4** |  | 369, MHNCI, WA |
| [*Botaurus pinnatus*] (Wagler, 1829) |  |  | 1 | 1 |  |  | **2** |  | 23, 391 |
| *Ixobrychus exilis* (Gmelin, 1789) |  | 1 |  | 10 |  |  | **11** | **SP** | 312, WA |
| *Ixobrychus involucris* (Vieillot, 1823) |  | 3 | 2 | 1 | 5 |  | **11** |  | 21, 312, 378, MHNCI |
| *Nycticorax nycticorax* (Linnaeus, 1758) |  | 249 | 88 | 68 | 221 |  | **626** |  | 21, 22, 23, 81, 120, 121, 122, 155, 158, 161, 171, 189, 207, 219, 226, 230, 294, 306, 313, 322, 357, 360, 363, 364, 375, 378, 379, 382, 383, 385, 387, 391, MHNCI, WA |
| *Nyctanassa violacea* (Linnaeus, 1758) |  |  |  | 98 |  |  | **98** |  | 207, 219, 226, 250, 272, 311, 360, 391, MHNCI, WA |
| *Butorides striata* (Linnaeus, 1758) |  | 148 | 176 | 61 | 195 | 2 | **582** |  | 21, 22, 23, 46, 54, 59, 81, 87, 94, 120, 121, 122, 134, 137, 138, 150, 155, 161, 181, 189, 207, 226, 250, 264, 306, 309, 313, 316, 322, 357, 360, 363, 364, 369, 375, 378, 379, 382, 383, 385, 387, 391, 405, 419, MHNCI, MZUSP, WA |
| *Bubulcus ibis* (Linnaeus, 1758) |  | 74 | 128 | 41 | 127 | 1 | **371** |  | 19, 22, 59, 74, 81, 87, 116, 122, 150, 155, 181, 194, 198, 219, 226, 246, 309, 313, 316, 322, 360, 363, 375, 378, 379, 382, 383, 385, 387, 391, 405, MHNCI, WA |
| *Ardea cocoi* Linnaeus, 1766 |  | 62 | 69 | 69 | 69 | 1 | **270** |  | 19, 21, 22, 120, 121, 122, 137, 138, 198, 207, 219, 226, 230, 250, 268, 306, 313, 316, 328, 354, 357, 360, 375, 378, 379, 383, 387, 391, 393, MHNCI, WA |
| *Ardea alba* Linnaeus, 1758 |  | 347 | 167 | 101 | 249 | 4 | **868** |  | 19, 21, 22, 46, 54, 74, 76, 77, 78, 81, 120, 121, 122, 137, 150, 155, 158, 161, 171, 181, 189, 198, 207, 219, 226, 230, 246, 250, 305, 306, 309, 313, 316, 322, 328, 357, 360, 363, 364, 375, 378, 379, 382, 383, 385, 387, 391, 405, MHNCI, WA |
| *Syrigma sibilatrix* (Temminck, 1824) |  | 147 | 86 | 38 | 229 | 4 | **504** |  | 10, 17, 19, 20, 21, 22, 54, 81, 94, 122, 134, 137, 150, 205, 246, 305, 309, 313, 316, 322, 357, 360, 363, 364, 375, 378, 379, 382, 383, 385, 389, 391, 405, FMNH, MHNCI, MZUSP, WA |
| *Pilherodius pileatus* (Boddaert, 1783) |  |  | 3 | 8 |  |  | **11** |  | 310, 324, 382, WA |
| *Egretta thula* (Molina, 1782) |  | 163 | 112 | 118 | 173 | 2 | **568** |  | 19, 21, 22, 46, 74, 76, 77, 78, 81, 87, 113, 120, 121, 122, 137, 155, 161, 171, 189, 198, 207, 219, 226, 230, 246, 267, 306, 309, 313, 316, 322, 328, 343, 357, 360, 363, 369, 375, 378, 379, 382, 385, 387, 391, 405, 419, MHNCI, WA |
| *Egretta caerulea* (Linnaeus, 1758) |  | 7 | 3 | 183 | 4 |  | **197** |  | 74, 81, 113, 136, 161, 198, 207, 219, 226, 230, 250, 378, 391, MHNCI, WA |
| Threskiornithidae Poche, 1904 |  |  |  |  |  |  |  |  |  |
| *Eudocimus ruber* (Linnaeus, 1758) |  |  |  | 68 |  |  | **68** |  | 159, 250, 324, 360, 391, 418, MHNCI, WA |
| *Plegadis chihi* (Vieillot, 1817) |  | 100 | 18 | 3 | 31 | 16 | **168** |  | 21, 309, 322, 324, 375, 378, 391, 405, MHNCI, WA |
| *Mesembrinibis cayennensis* (Gmelin, 1789) |  | 16 | 26 | 7 | 65 |  | **114** |  | 21, 52, 94, 101, 120, 121, 122, 250, 312, 316, 369, 375, 378, 379, 387, 391, MHNCI, MZUSP, WA |
| *Phimosus infuscatus* (Lichtenstein, 1823) |  | 168 | 31 | 59 | 38 |  | **296** |  | 194, 322, 378, 391, 405, MHNCI, WA |
| *Theristicus caerulescens* (Vieillot, 1817) |  |  |  |  | 1 |  | **1** | **N, C** | WA |
| *Theristicus caudatus* (Boddaert, 1783) |  | 183 | 48 | 24 | 289 | 10 | **554** |  | 5, 17, 19, 21, 122, 138, 150, 186, 194, 205, 250, 264, 293, 305, 309, 316, 322, 357, 360, 364, 375, 378, 379, 385, 389, 391, MHNCI, MZUSP, WA |
| *Platalea ajaja* Linnaeus, 1758 |  | 42 | 14 | 69 | 59 |  | **184** |  | 81, 120, 121, 122, 207, 250, 328, 360, 378, 391, 405, WA |
| Cathartiformes Seebohm, 1890 |  |  |  |  |  |  |  |  |  |
| Cathartidae Lafresnaye, 1839 |  |  |  |  |  |  |  |  |  |
| *Cathartes aura* (Linnaeus, 1758) |  | 80 | 112 | 99 | 80 | 8 | **379** |  | 5, 17, 19, 21, 23, 47, 59, 74, 76, 77, 78, 81, 94, 120, 134, 137, 150, 194, 195, 198, 207, 219, 226, 230, 246, 290, 294, 305, 306, 309, 313, 316, 322, 357, 360, 363, 364, 375, 378, 379, 382, 383, 385, 387, 389, 391, 419, MHNCI, WA |
| *Cathartes burrovianus* Cassin, 1845 |  |  | 30 |  |  |  | **30** |  | 313, 324, 374, 375, 383, 387, MHNCI, WA |
| *Coragyps atratus* (Bechstein, 1793) |  | 333 | 233 | 109 | 331 | 10 | **1016** |  | 5, 10, 17, 19, 21, 23, 47, 59, 74, 76, 77, 78, 81, 114, 116, 119, 120, 133, 134, 137, 138, 150, 152, 155, 161, 170, 181, 194, 198, 207, 219, 226, 230, 271, 294, 305, 306, 309, 313, 316, 322, 333, 357, 360, 363, 364, 375, 378, 379, 382, 383, 385, 387, 389, 391, 419, WA |
| *Sarcoramphus papa* (Linnaeus, 1758) |  | 20 | 36 | 4 | 33 |  | **93** |  | 19, 21, 41, 52, 59, 219, 250, 305, 306, 309, 313, 316, 357, 369, 375, 379, 387, 391, 393, MHNCI, WA |
| Accipitriformes Bonaparte, 1831 |  |  |  |  |  |  |  |  |  |
| Pandionidae Bonaparte, 1854 |  |  |  |  |  |  |  |  |  |
| *Pandion haliaetus* (Linnaeus, 1758) |  | 7 | 43 | 13 | 5 |  | **68** |  | 21, 311, 313, 378, 391, WA |
| Accipitridae Vigors, 1824 |  |  |  |  |  |  |  |  |  |
| *Leptodon cayanensis* (Latham, 1790) |  | 15 | 44 | 11 | 41 |  | **111** |  | 5, 19, 21, 22, 59, 136, 137, 150, 249, 258, 294, 309, 313, 360, 363, 364, 378, 379, 382, 385, 391, MHNCI, MZUSP, WA |
| *Chondrohierax uncinatus* (Temminck, 1822) |  |  | 2 | 9 | 1 |  | **12** |  | 81, 91, 194, 198, 219, MHNCI, WA |
| *Elanoides forficatus* (Linnaeus, 1758) |  | 47 | 34 | 51 | 108 | 1 | **241** |  | 5, 21, 22, 46, 47, 81, 134, 137, 150, 155, 181, 249, 306, 309, 313, 360, 363, 364, 375, 378, 379, 382, 385, 391, MHNCI, WA |
| *Gampsonyx swainsonii* Vigors, 1825 |  |  | 43 |  | 1 |  | **44** | **C** | 307, 310, 313, MHNCI, WA |
| *Elanus leucurus* (Vieillot, 1818) |  | 225 | 117 | 5 | 185 | 2 | **534** |  | 5, 10, 19, 21, 22, 54, 116, 150, 181, 219, 249, 305, 306, 309, 313, 316, 357, 363, 364, 375, 378, 379, 382, 383, 385, 387, 391, 405, 419, MHNCI, WA |
| *Harpagus diodon* (Temminck, 1823) |  | 14 | 18 | 24 | 22 |  | **78** |  | 5, 13, 19, 21, 22, 47, 49, 59, 81, 88, 150, 155, 249, 258, 306, 313, 378, 379, 383, 391, 393, 419, MHNCI, WA |
| [*Circus cinereus*] Vieillot, 1816 |  |  |  | 1 |  |  | **1** |  | 61 |
| *Circus buffoni* (Gmelin, 1788) |  | 29 | 29 | 3 | 12 | 12 | **85** |  | 193, 206, 324, 378, 391, 409, MHNCI, WA |
| *Accipiter poliogaster* (Temminck, 1824) |  | 9 |  | 5 | 8 |  | **22** |  | 41, 81, 91, 165, 194, 284, 316, 352, 363, 391, WA |
| *Accipiter superciliosus* (Linnaeus, 1766) |  |  |  | 12 | 11 |  | **23** |  | 92, 94, 195, 378, 391, 393, WA |
| *Accipiter striatus* Vieillot, 1808 |  | 57 | 33 | 7 | 66 |  | **163** |  | 21, 22, 46, 81, 94, 137, 142, 150, 190, 194, 249, 258, 309, 313, 316, 363, 378, 379, 387, 391, 393, FMNH, MHNCI, WA |
| *Accipiter bicolor* (Vieillot, 1817) |  | 5 |  | 24 | 8 |  | **37** |  | 81, 87, 88, 101, 249, 250, 378, 379, 382, 393, MHNCI, WA |
| *Ictinia plumbea* (Gmelin, 1788) |  | 12 | 196 | 4 | 87 | 1 | **300** |  | 13, 19, 21, 22, 59, 134, 138, 155, 187, 188, 206, 264, 294, 306, 309, 313, 360, 369, 375, 379, 382, 383, 385, 387, 391, 393, FMNH, MHNCI, MZUSP, WA |
| *Busarellus nigricollis* (Latham, 1790) |  | 1 | 10 |  |  |  | **11** |  | 313, 324, 374, 375, MHNCI, WA |
| *Rostrhamus sociabilis* (Vieillot, 1817) |  | 6 | 74 | 8 | 1 |  | **89** |  | 120, 136, 249, 294, 313, 360, 375, 378, 382, 387, 391, MHNCI, WA |
| *Geranospiza caerulescens* (Vieillot, 1817) |  | 19 | 21 | 5 | 22 |  | **67** |  | 21, 120, 304, 306, 309, 313, 316, 375, 378, 379, 383, 391, 409, MHNCI, WA |
| *Buteogallus aequinoctialis* (Gmelin, 1788) |  |  |  | 22 |  |  | **22** |  | 81, 136, 219, 250, 311, 391, WA |
| *Heterospizias meridionalis* (Latham, 1790) |  | 74 | 32 | 10 | 74 | 2 | **192** |  | 5, 17, 19, 21, 22, 249, 250, 309, 316, 357, 364, 369, 375, 378, 379, 382, 383, 385, 389, 391, MHNCI, MZUSP, WA |
| *Amadonastur lacernulatus* (Temminck, 1827) |  | 1 |  | 40 | 1 |  | **42** |  | 47, 81, 88, 198, 219, 360, 378, 382, 391, MHNCI, WA |
| *Urubitinga urubitinga* (Gmelin, 1788) |  | 19 | 24 | 23 | 18 |  | **84** |  | 21, 81, 120, 226, 249, 357, 369, 378, 379, 387, 391, 392, MHNCI, WA |
| *Urubitinga coronata* (Vieillot, 1817) |  | 21 | 4 |  | 4 |  | **29** |  | 90, 91, 148, 193, 250, 309, 310, 316, 387, MZUSP, WA |
| *Rupornis magnirostris* (Gmelin, 1788) |  | 438 | 367 | 105 | 477 | 12 | **1399** |  | 5, 10, 13, 14, 17, 19, 21, 22, 23, 35, 46, 47, 54, 59, 81, 112, 115, 116, 119, 120, 134, 136, 137, 138, 142, 150, 155, 161, 170, 181, 190, 194, 198, 207, 219, 226, 230, 249, 250, 253, 267, 294, 305, 306, 309, 313, 316, 322, 350, 357, 360, 363, 364, 369, 375, 378, 379, 382, 383, 385, 387, 389, 391, 393, 397, 405, 414, 419, FMNH, MHNCI, MZUSP, WA |
| *Parabuteo unicinctus* (Temminck, 1824) |  |  | 1 | 1 | 2 |  | **4** |  | 5, 91, 393, WA |
| *Parabuteo leucorrhous* (Quoy & Gaimard, 1824) |  | 22 | 2 | 8 | 23 |  | **55** |  | 13, 19, 21, 103, 134, 157, 306, 309, 363, 364, 379, 382, MHNCI, WA |
| *Geranoaetus albicaudatus* (Vieillot, 1816) |  | 52 | 64 | 4 | 30 | 5 | **155** |  | 17, 19, 21, 22, 90, 118, 222, 249, 250, 309, 313, 316, 357, 364, 378, 379, 383, 389, 391, 419, MHNCI, WA |
| *Geranoaetus melanoleucus* (Vieillot, 1819) |  | 33 | 1 |  | 8 | 2 | **44** |  | 19, 21, 22, 250, 289, 306, 309, 316, 360, 379, 389, 391, WA |
| *Pseudastur polionotus* (Kaup, 1847) |  | 12 | 2 | 31 | 40 |  | **85** |  | 27, 47, 103, 149, 150, 249, 289, 309, 356, 357, 360, 363, 367, 375, 378, 379, 382, 391, MHNCI, WA |
| *Buteo nitidus* (Latham, 1790) |  |  | 3 |  |  |  | **3** | **C** | 13, 21, 22, 383 |
| *Buteo platypterus* (Vieillot, 1823) |  |  | 1 |  |  |  | **1** | **N** | WA |
| *Buteo brachyurus* Vieillot, 1816 |  | 104 | 81 | 22 | 96 | 1 | **304** |  | 21, 59, 81, 150, 181, 192, 198, 206, 249, 258, 306, 309, 313, 316, 364, 378, 379, 383, 387, 389, 391, WA |
| *Buteo swainsoni* Bonaparte, 1838 |  | 2 | 2 | 1 | 2 |  | **7** |  | 21, 324, 378, 393 |
| *Buteo albonotatus* Kaup, 1847 |  | 3 | 4 |  | 2 |  | **9** |  | 309, 313, 316, 378, 379, WA |
| *Morphnus guianensis* (Daudin, 1800) |  |  | 1 |  |  |  | **1** |  | 386 |
| *Harpia harpyja* (Linnaeus, 1758) |  |  | 1 | 1 | 6 |  | **8** |  | 320, 324, 368, 410, MHNCI, MZUSP |
| *Spizaetus tyrannus* (Wied, 1820) |  | 34 | 4 | 25 | 52 |  | **115** |  | 5, 47, 81, 109, 149, 150, 249, 289, 290, 309, 315, 316, 363, 367, 378, 379, 382, 387, 391, MHNCI, WA |
| *Spizaetus melanoleucus* (Vieillot, 1816) |  | 2 | 7 | 21 | 11 |  | **41** |  | 21, 22, 379, 382, MHNCI, WA |
| *Spizaetus ornatus* (Daudin, 1800) |  | 3 | 9 | 8 | 5 |  | **25** |  | 87, 278, 324, 387, 393, MHNCI, WA |
| Gruiformes Bonaparte, 1854 |  |  |  |  |  |  |  |  |  |
| Aramidae Bonaparte, 1852 |  |  |  |  |  |  |  |  |  |
| *Aramus guarauna* (Linnaeus, 1766) |  | 7 | 149 | 27 | 2 |  | **185** |  | 21, 120, 294, 306, 316, 369, 375, 378, 382, 387, 391, 419, MHNCI, WA |
| Rallidae Rafinesque, 1815 |  |  |  |  |  |  |  |  |  |
| [*Micropygia schomburgkii*] (Cabanis, 1848) |  | 1 |  |  |  |  | **1** |  | 400 |
| *Rallus longirostris* Boddaert, 1783 |  |  |  | 17 |  |  | **17** |  | 324, 360, WA |
| [*Aramides ypecaha*] (Vieillot, 1819) |  |  | 3 | 1 | 1 |  | **5** |  | 62, 193, 324, 378 |
| *Aramides mangle* (Spix, 1825) |  |  | 1 | 5 |  |  | **6** | **SP** | 62, 81, 324, WA |
| *Aramides cajaneus* (Statius Muller, 1776) |  | 11 | 21 | 56 | 2 |  | **90** |  | 10, 13, 17, 19, 20, 21, 81, 137, 181, 198, 207, 219, 226, 230, 250, 264, 294, 305, 306, 313, 357, 363, 369, 375, 378, 391, 393, 419, MHNCI, MZUSP, WA |
| *Aramides saracura* (Spix, 1825) |  | 322 | 142 | 72 | 402 | 4 | **942** |  | 5, 13, 14, 19, 21, 46, 47, 54, 59, 81, 87, 94, 134, 138, 142, 150, 155, 161, 170, 173, 181, 194, 219, 258, 267, 305, 306, 309, 316, 322, 357, 360, 363, 364, 378, 379, 382, 383, 385, 387, 389, 391, 392, 393, 405, FMNH, MHNCI, WA |
| *Amaurolimnas concolor* (Gosse, 1847) |  |  | 3 | 16 |  |  | **19** |  | 59, 81, 134, 391, WA |
| *Laterallus melanophaius* (Vieillot, 1819) |  | 18 | 21 | 44 | 25 | 2 | **110** |  | 21, 22, 59, 181, 246, 309, 363, 378, 379, 382, 391, 405, MHNCI, WA |
| *Laterallus exilis* (Temminck, 1831) |  |  |  | 14 |  |  | **14** |  | 326, WA |
| *Laterallus leucopyrrhus* (Vieillot, 1819) |  | 7 |  | 1 | 15 |  | **23** |  | 316, 378, 405, MHNCI, WA |
| *Porzana flaviventer* (Boddaert, 1783) |  |  | 3 | 5 |  |  | **8** |  | 62, 324, 391 |
| *Porzana albicollis* (Vieillot, 1819) |  | 17 | 19 | 32 | 41 | 1 | **110** |  | 21, 59, 137, 246, 247, 305, 316, 322, 375, 378, 379, 382, 383, 391, MZUSP, WA |
| *Neocrex erythrops* (Sclater, 1867) |  |  |  |  | 1 |  | **1** |  | MHNCI |
| *Pardirallus maculatus* (Boddaert, 1783) |  | 1 | 5 |  | 2 | 1 | **9** |  | 21, 324, 375, 378, 419, MHNCI |
| *Pardirallus nigricans* (Vieillot, 1819) |  | 145 | 70 | 23 | 165 | 2 | **405** |  | 21, 22, 46, 59, 81, 136, 137, 150, 161, 230, 250, 306, 309, 316, 322, 357, 360, 363, 364, 375, 378, 379, 382, 383, 385, 387, 391, 392, 393, 405, 419, MHNCI, MZUSP, WA |
| *Pardirallus sanguinolentus* (Swainson, 1837) |  | 42 | 9 | 3 | 31 | 5 | **90** |  | 21, 87, 219, 306, 316, 360, 378, 379, 391, 405, MHNCI, WA |
| *Gallinula galeata* (Lichtenstein, 1818) |  | 369 | 152 | 43 | 274 | 6 | **844** |  | 5, 21, 46, 53, 54, 59, 150, 161, 171, 181, 189, 194, 246, 306, 309, 313, 316, 322, 357, 363, 375, 378, 379, 383, 385, 387, 391, 399, 405, 419, MHNCI, WA |
| *Gallinula melanops* (Vieillot, 1819) |  | 22 |  | 1 | 14 |  | **37** |  | 378, MHNCI, WA |
| *Porphyrio martinicus* (Linnaeus, 1766) |  | 21 | 45 | 17 | 43 |  | **126** |  | 21, 137, 306, 322, 375, 378, 391, 405, 419, MHNCI, WA |
| *Porphyrio flavirostris* (Gmelin, 1789) |  | 1 | 2 | 1 |  |  | **4** |  | 21, 58, 309 |
| *Fulica armillata* Vieillot, 1817 |  | 7 | 1 | 2 | 3 |  | **13** |  | 332, 378, WA |
| [*Fulica rufifrons*] Philippi & Landbeck, 1861 |  |  |  |  | 1 |  | **1** |  | 379 |
| *Fulica leucoptera* Vieillot, 1817 |  | 30 | 5 | 1 | 8 |  | **44** |  | 21, 324, 378, 419, MHNCI, WA |
| Heliornithidae Gray, 1840 |  |  |  |  |  |  |  |  |  |
| *Heliornis fulica* (Boddaert, 1783) |  |  | 5 | 1 |  |  | **6** |  | 155, 310, 311, 369, MHNCI, WA |
| Charadriiformes Huxley, 1867 |  |  |  |  |  |  |  |  |  |
| Charadrii Huxley, 1867 |  |  |  |  |  |  |  |  |  |
| Charadriidae Leach, 1820 |  |  |  |  |  |  |  |  |  |
| *Vanellus cayanus* (Latham, 1790) |  |  | 9 |  | 2 |  | **11** |  | 94, 369, 375, 378, 392, 393, MHNCI |
| *Vanellus chilensis* (Molina, 1782) |  | 609 | 287 | 91 | 565 | 14 | **1566** |  | 5, 10, 19, 20, 21, 22, 23, 59, 81, 113, 114, 116, 119, 120, 125, 137, 138, 150, 155, 161, 170, 171, 181, 189, 194, 198, 207, 212, 213, 214, 215, 216, 219, 226, 246, 247, 250, 294, 305, 306, 309, 313, 316, 322, 357, 360, 363, 364, 375, 378, 379, 382, 383, 385, 387, 389, 391, 393, 405, 419, MHNCI, MZUEL, WA |
| *Pluvialis dominica* (Statius Muller, 1776) |  | 9 | 1 | 18 | 11 | 6 | **45** |  | 114, 226, 250, 378, 391, FMNH, MHNCI, WA |
| *Pluvialis squatarola* (Linnaeus, 1758) | 9 |  |  |  |  |  | **9** | **SP** | 62, 114, 226, 391, WA |
| *Charadrius semipalmatus* Bonaparte, 1825 |  | 1 | 1 | 82 | 1 |  | **85** |  | 113, 117, 125, 198, 207, 219, 226, 230, 246, 250, 369, 378, 391, MHNCI, MZUSP, WA |
| *Charadrius collaris* Vieillot, 1818 |  | 3 | 7 | 47 | 3 |  | **60** |  | 113, 114, 117, 120, 125, 134, 198, 219, 226, 231, 246, 250, 343, 369, 375, 378, 391, MHNCI, WA |
| *Charadrius modestus* Lichtenstein, 1823 |  |  | 1 | 3 |  |  | **4** |  | 226, 294, MHNCI, WA |
| Haematopodidae Bonaparte, 1838 |  |  |  |  |  |  |  |  |  |
| *Haematopus palliatus* Temminck, 1820 | 49 |  |  |  |  |  | **49** |  | 113, 219, 226, 311, 391, MHNCI, WA |
| Recurvirostridae Bonaparte, 1831 |  |  |  |  |  |  |  |  |  |
| *Himantopus melanurus* Vieillot, 1817 |  | 149 | 80 | 34 | 115 | 17 | **395** |  | 21, 120, 171, 309, 316, 322, 328, 343, 375, 378, 379, 391, 405, 419, MHNCI, MZUSP, WA |
| Chionidae Lesson, 1828 |  |  |  |  |  |  |  |  |  |
| [*Chionis albus*] (Gmelin, 1789) | 2 |  |  |  |  |  | **2** |  | 301, 324 |
| Scolopaci Steijneger, 1885 |  |  |  |  |  |  |  |  |  |
| Scolopacidae Rafinesque, 1815 |  |  |  |  |  |  |  |  |  |
| *Gallinago paraguaiae* (Vieillot, 1816) |  | 52 | 8 | 28 | 71 | 5 | **164** |  | 19, 21, 87, 94, 246, 247, 250, 305, 309, 316, 357, 375, 378, 379, 382, 391, 405, AMNH, MHNCI, MZUSP, WA |
| *Gallinago undulata* (Boddaert, 1783) |  | 5 |  |  | 2 |  | **7** |  | 19, 21, 94, 250, MHNCI, WA |
| *Limosa haemastica* (Linnaeus, 1758) |  | 3 |  | 1 | 3 | 1 | **8** |  | 378, MHNCI, WA |
| [*Numenius phaeopus*] (Linnaeus, 1758) | 4 |  |  |  |  |  | **4** |  | 62, 324 |
| *Bartramia longicauda* (Bechstein, 1812) |  | 7 | 4 | 4 | 5 |  | **20** |  | 19, 21, 62, 309, 312, 378, 379, MHNCI, WA |
| *Actitis macularius* (Linnaeus, 1766) |  | 5 | 7 | 35 | 6 |  | **53** |  | 21, 81, 136, 207, 226, 230, 250, 378, 379, 391, 419, WA |
| *Tringa solitaria* Wilson, 1813 |  | 68 | 72 | 5 | 104 | 1 | **250** |  | 21, 94, 136, 152, 171, 181, 246, 250, 309, 313, 316, 322, 369, 378, 391, 419, MHNCI, MZUEL, MZUSP, WA |
| *Tringa melanoleuca* (Gmelin, 1789) |  | 38 | 8 | 21 | 82 | 3 | **152** |  | 21, 171, 207, 309, 312, 322, 378, 379, 391, 405, 419, MHNCI, WA |
| *Tringa semipalmata* (Gmelin, 1789) | 2 |  |  |  |  |  | **2** | **SP** | 224, WA |
| *Tringa flavipes* (Gmelin, 1789) |  | 145 | 39 | 24 | 102 | 25 | **335** |  | 19, 21, 114, 120, 171, 198, 207, 219, 226, 246, 294, 309, 312, 316, 322, 328, 369, 375, 378, 383, 391, 405, 419, MHNCI, WA |
| *Arenaria interpres* (Linnaeus, 1758) | 6 |  |  |  |  |  | **6** |  | 62, 226, 311, 324, WA |
| *Calidris canutus* (Linnaeus, 1758) | 5 |  |  |  |  |  | **5** |  | 114, 224, 324, WA |
| *Calidris alba* (Pallas, 1764) | 13 |  |  |  |  |  | **13** |  | 198, 219, 221, 226, 391, WA |
| *Calidris pusilla* (Linnaeus, 1766) | 2 |  |  |  |  |  | **2** | **N** | WA |
| [*Calidris minutilla*] (Vieillot, 1819) | 1 |  |  |  |  |  | **1** |  | 226 |
| *Calidris fuscicollis* (Vieillot, 1819) |  | 16 | 9 | 23 | 31 | 10 | **89** |  | 21, 113, 198, 219, 226, 294, 322, 328, 378, 391, 419, WA |
| [*Calidris bairdii*] (Coues, 1861) | 1 |  |  |  |  |  | **1** |  | 226 |
| *Calidris melanotos* (Vieillot, 1819) |  | 38 | 6 | 9 | 31 | 20 | **104** |  | 21, 219, 312, 378, 391, WA |
| *Calidris himantopus* (Bonaparte, 1826) |  | 1 | 1 | 3 | 1 | 4 | **10** | **SP** | 62, 312, 324, 378, WA |
| *Calidris subruficollis* (Vieillot, 1819) |  | 1 |  | 6 | 1 |  | **8** |  | 62, 153, 312, 324, 378, 391, WA |
| *Phalaropus tricolor* (Vieillot, 1819) |  | 13 | 2 | 4 | 6 | 9 | **34** |  | 226, 319, 324, 378, 391, WA |
| Jacanidae Chenu & Des Murs, 1854 |  |  |  |  |  |  |  |  |  |
| *Jacana jacana* (Linnaeus, 1766) |  | 234 | 178 | 68 | 258 | 12 | **750** |  | 5, 21, 59, 81, 87, 94, 120, 134, 136, 137, 138, 150, 155, 161, 171, 181, 194, 246, 305, 306, 309, 313, 316, 322, 357, 360, 363, 369, 375, 378, 379, 382, 383, 385, 387, 389, 391, 399, 405, 419, AMNH, MHNCI, MZUSP, NMNH, WA |
| Rostratulidae Mathews, 1914 |  |  |  |  |  |  |  |  |  |
| *Nycticryphes semicollaris* (Vieillot, 1816) |  |  |  | 1 |  |  | **1** |  | 61 |
| Lari Sharpe, 1891 |  |  |  |  |  |  |  |  |  |
| Stercorariidae Gray, 1870 |  |  |  |  |  |  |  |  |  |
| *Stercorarius chilensis* Bonaparte, 1857 | 7 |  |  |  |  |  | **7** | **N** | WA |
| *Stercorarius maccormicki* Saunders, 1893 | 2 |  |  |  |  |  | **2** |  | 226, WA |
| *Stercorarius antarcticus* (Lesson, 1831) | 4 |  |  |  |  |  | **4** |  | 226, WA |
| *Stercorarius pomarinus* (Temminck, 1815) | 5 |  |  |  |  |  | **5** |  | 241, WA |
| *Stercorarius parasiticus* (Linnaeus, 1758) | 5 |  |  |  |  |  | **5** | **SP** | 241, 324, WA |
| [*Stercorarius longicaudus*] Vieillot, 1819 | 1 |  |  |  |  |  | **1** |  | 326 |
| Laridae Rafinesque, 1815 |  |  |  |  |  |  |  |  |  |
| *Chroicocephalus maculipennis* (Lichtenstein, 1823) | 20 |  |  |  |  |  | **20** |  | 114, 117, 198, 219, 226, WA |
| [*Chroicocephalus cirrocephalus*] (Vieillot, 1818) | 4 |  |  |  |  |  | **4** |  | 198, 219, 224, 226 |
| *Larus dominicanus* Lichtenstein, 1823 | 202 |  |  |  |  |  | **202** |  | 74, 75, 76, 77, 78, 79, 113, 114, 125, 137, 158, 198, 219, 226, 230, 246, 271, 360, 391, MHNCI, WA |
| Sternidae Vigors, 1825 |  |  |  |  |  |  |  |  |  |
| *Anous stolidus* (Linnaeus, 1758) | 2 |  |  |  |  |  | **2** |  | 82, 228 |
| *Sternula antillarum* Lesson, 1847 | 4 |  |  |  |  |  | **4** | **N** | WA |
| *Sternula superciliaris* (Vieillot, 1819) |  |  | 5 | 20 |  |  | **25** |  | 116, 120, 226, 230, 343, MHNCI, WA |
| *Phaetusa simplex* (Gmelin, 1789) |  | 5 | 31 | 2 | 1 |  | **39** |  | 120, 369, 375, 378, 391, MHNCI, WA |
| *Sterna hirundo* Linnaeus, 1758 | 4 |  |  |  |  |  | **4** |  | 230, 391, WA |
| *Sterna paradisaea* Pontoppidan, 1763 |  |  | 1 |  |  |  | **1** | **N** | WA |
| *Sterna hirundinacea* Lesson, 1831 | 25 |  |  |  |  |  | **25** |  | 74, 78, 79, 158, 226, 230, 391, MHNCI, WA |
| *Sterna trudeaui* Audubon, 1838 | 9 |  |  |  |  |  | **9** |  | WA |
| *Thalasseus acuflavidus* (Cabot, 1847) | 94 |  |  |  |  |  | **94** |  | 74, 76, 77, 78, 79, 125, 136, 158, 198, 219, 226, 230, 250, 271, 360, 391, MHNCI, WA |
| *Thalasseus maximus* (Boddaert, 1783) | 34 |  |  |  |  |  | **34** |  | 74, 78, 79, 136, 226, 250, 311, MHNCI, WA |
| Rynchopidae Bonaparte, 1838 |  |  |  |  |  |  |  |  |  |
| *Rynchops niger* Linnaeus, 1758 |  |  | 16 | 65 | 17 | 1 | **99** |  | 74, 120, 207, 226, 343, 369, 375, 378, 391, MHNCI, WA |
| Columbiformes Latham, 1790 |  |  |  |  |  |  |  |  |  |
| Columbidae Leach, 1820 |  |  |  |  |  |  |  |  |  |
| *Columbina minuta* (Linnaeus, 1766) |  |  | 7 |  |  |  | **7** |  | 13, 21, 324, MHNCI |
| *Columbina talpacoti* (Temminck, 1811) |  | 387 | 191 | 67 | 364 | 6 | **1015** |  | 5, 8, 10, 13, 17, 19, 20, 21, 23, 46, 54, 59, 81, 116, 119, 120, 132, 134, 137, 150, 155, 170, 181, 194, 198, 207, 267, 294, 305, 306, 309, 313, 316, 322, 357, 360, 363, 364, 369, 375, 378, 379, 382, 383, 385, 387, 389, 391, 392, 393, 405, 419, FMNH, MHNCI, MZUSP, WA |
| *Columbina squammata* (Lesson, 1831) |  | 45 | 84 | 1 | 87 | 3 | **220** |  | 13, 21, 23, 120, 181, 194, 205, 309, 313, 316, 357, 364, 369, 375, 378, 379, 383, 385, 389, 391, 392, 393, FMNH, MHNCI, WA |
| *Columbina picui* (Temminck, 1813) |  | 11 | 139 | 2 | 37 | 2 | **191** |  | 13, 21, 23, 59, 119, 120, 137, 155, 181, 294, 306, 309, 313, 360, 364, 375, 378, 379, 383, 385, 387, 389, 391, 415, 419, MHNCI, WA |
| *Claravis pretiosa* (Ferrari-Perez, 1886) |  | 1 | 14 | 1 | 8 |  | **24** |  | 5, 13, 21, 304, 306, 313, 366, 379, 385, 391, MHNCI, WA |
| [*Claravis geoffroyi*] (Temminck, 1811) |  |  |  | 5 |  |  | **5** |  | 81, 88, 324, 360 |
| *Columba livia* Gmelin, 1789 |  | 574 | 64 | 17 | 265 |  | **920** | **I** | 5, 20, 116, 181, 194, 378, 391, 419, WA |
| *Patagioenas speciosa* (Gmelin, 1789) |  |  | 5 |  |  |  | **5** |  | 13, 17, 19, 21, 22, 310, 313, 324, 374 |
| *Patagioenas picazuro* (Temminck, 1813) |  | 329 | 247 | 28 | 313 | 13 | **930** |  | 4, 5, 13, 14, 15, 17, 19, 21, 22, 23, 46, 54, 59, 81, 116, 119, 120, 150, 155, 181, 194, 258, 294, 305, 306, 309, 313, 316, 322, 357, 364, 375, 378, 379, 383, 385, 387, 389, 391, 405, 414, 419, MHNCI, WA |
| *Patagioenas cayennensis* (Bonnaterre, 1792) |  | 26 | 86 | 48 | 50 | 8 | **218** |  | 13, 14, 17, 19, 21, 22, 23, 47, 59, 81, 94, 119, 120, 136, 155, 181, 194, 198, 207, 230, 250, 258, 264, 305, 306, 309, 313, 316, 357, 364, 369, 375, 378, 379, 383, 385, 389, 391, 392, 393, MHNCI, MZUSP, WA |
| *Patagioenas plumbea* (Vieillot, 1818) |  | 18 | 5 | 51 | 41 |  | **115** |  | 5, 13, 14, 17, 19, 21, 22, 47, 81, 83, 94, 134, 137, 198, 219, 258, 306, 309, 316, 360, 363, 369, 378, 379, 382, 391, 392, 393, MHNCI, WA |
| *Zenaida auriculata* (Des Murs, 1847) |  | 415 | 427 | 12 | 389 | 13 | **1256** |  | 4, 5, 13, 17, 19, 21, 22, 23, 59, 81, 94, 116, 119, 120, 132, 150, 155, 167, 170, 181, 188, 194, 199, 205, 206, 250, 267, 304, 305, 306, 309, 313, 316, 322, 340, 357, 364, 375, 378, 379, 382, 383, 385, 387, 389, 391, 415, 419, MHNCI, WA |
| *Leptotila verreauxi* Bonaparte, 1855 |  | 138 | 116 | 34 | 208 | 2 | **498** |  | 5, 13, 14, 17, 19, 21, 23, 46, 47, 54, 59, 81, 94, 119, 120, 134, 142, 150, 152, 155, 181, 190, 194, 198, 219, 230, 250, 258, 264, 305, 306, 309, 313, 316, 322, 357, 360, 363, 364, 369, 375, 378, 379, 382, 383, 385, 387, 391, 392, 393, 414, FMNH, MHNCI, MZUSP, WA |
| *Leptotila rufaxilla* (Richard & Bernard, 1792) |  | 67 | 43 | 30 | 113 |  | **253** |  | 5, 13, 14, 17, 19, 21, 23, 46, 47, 81, 94, 119, 136, 137, 142, 150, 155, 181, 190, 194, 198, 207, 219, 230, 294, 305, 306, 309, 313, 316, 322, 357, 360, 363, 364, 375, 378, 379, 382, 383, 385, 391, 392, 393, 414, MHNCI, MZUSP, WA |
| *Geotrygon violacea* (Temminck, 1809) |  |  | 1 |  | 2 |  | **3** |  | 385, 392, 393 |
| *Geotrygon montana* (Linnaeus, 1758) |  | 43 | 16 | 27 | 35 |  | **121** |  | 10, 13, 14, 17, 19, 20, 21, 47, 81, 134, 137, 150, 170, 173, 181, 192, 198, 205, 250, 279, 294, 306, 309, 316, 360, 363, 364, 378, 379, 382, 387, 391, 392, MHNCI, MZUSP, WA |
| Cuculiformes Wagler, 1830 |  |  |  |  |  |  |  |  |  |
| Cuculidae Leach, 1820 |  |  |  |  |  |  |  |  |  |
| Cuculinae Leach, 1820 |  |  |  |  |  |  |  |  |  |
| [*Micrococcyx cinereus*] (Vieillot, 1817) |  |  | 1 | 2 |  |  | **3** |  | 65, 137, 313 |
| *Piaya cayana* (Linnaeus, 1766) |  | 291 | 268 | 49 | 342 | 7 | **957** |  | 5, 10, 13, 14, 15, 17, 19, 21, 23, 46, 47, 54, 59, 81, 94, 119, 134, 137, 150, 155, 161, 170, 181, 194, 198, 219, 264, 267, 294, 305, 306, 309, 313, 316, 322, 357, 363, 364, 375, 378, 379, 382, 383, 385, 387, 389, 391, 393, 419, FMNH, MHNCI, MZUSP, WA |
| *Coccyzus melacoryphus* Vieillot, 1817 |  | 31 | 62 | 13 | 77 | 1 | **184** |  | 21, 23, 81, 134, 150, 155, 181, 207, 264, 267, 306, 309, 313, 322, 364, 366, 378, 379, 383, 387, 391, 392, 393, 419, MHNCI, MZUSP, WA |
| *Coccyzus americanus* (Linnaeus, 1758) |  | 13 | 1 | 2 | 25 |  | **41** |  | 14, 17, 19, 21, 54, 192, 378, 379, MHNCI, WA |
| *Coccyzus euleri* Cabanis, 1873 |  |  | 6 | 3 | 5 |  | **14** |  | 21, 43, 81, 264, 306, 369, 379, MHNCI, MZUSP, WA |
| [*Coccyzus erythropthalmus*] (Wilson, 1811) |  |  | 1 |  |  |  | **1** |  | 310, 313 |
| Crotophaginae Swainson, 1837 |  |  |  |  |  |  |  |  |  |
| *Crotophaga major* Gmelin, 1788 |  | 4 | 74 |  | 18 |  | **96** |  | 21, 23, 94, 118, 134, 264, 294, 306, 313, 369, 375, 379, 383, 385, 387, 391, 392, 393, MHNCI, MZUSP, WA |
| *Crotophaga ani* Linnaeus, 1758 |  | 147 | 277 | 54 | 266 | 7 | **751** |  | 5, 10, 13, 19, 21, 23, 29, 59, 81, 112, 116, 119, 120, 132, 134, 135, 137, 150, 152, 155, 161, 170, 181, 198, 219, 246, 267, 294, 306, 309, 313, 316, 322, 357, 360, 363, 364, 369, 375, 378, 379, 382, 383, 385, 387, 389, 391, 392, 393, 405, 415, 417, 419, FMNH, MHNCI, WA |
| *Guira guira* (Gmelin, 1788) |  | 292 | 276 | 47 | 418 | 7 | **1040** |  | 5, 10, 17, 19, 21, 23, 42, 46, 54, 59, 81, 94, 116, 119, 120, 134, 137, 150, 152, 155, 161, 170, 181, 198, 219, 267, 294, 305, 306, 309, 313, 316, 322, 354, 357, 360, 363, 364, 369, 375, 378, 379, 382, 383, 385, 387, 389, 391, 392, 405, 419, MHNCI, WA |
| Taperinae Verheyen, 1956 |  |  |  |  |  |  |  |  |  |
| *Tapera naevia* (Linnaeus, 1766) |  | 72 | 124 | 29 | 143 | 4 | **372** |  | 5, 17, 19, 21, 23, 59, 81, 120, 137, 150, 155, 181, 194, 207, 305, 306, 309, 313, 316, 322, 357, 360, 363, 364, 375, 378, 379, 382, 383, 385, 387, 389, 391, 393, 414, 419, MHNCI, WA |
| *Dromococcyx phasianellus* (Spix, 1824) |  | 1 | 1 |  | 5 |  | **7** |  | MHNCI, WA |
| *Dromococcyx pavoninus* Pelzeln, 1870 |  | 12 | 58 | 7 | 36 |  | **113** |  | 21, 47, 81, 88, 138, 150, 155, 305, 306, 313, 316, 363, 378, 379, 382, 383, 385, 387, 391, 392, 393, MHNCI, WA |
| Strigiformes Wagler, 1830 |  |  |  |  |  |  |  |  |  |
| Tytonidae Mathews, 1912 |  |  |  |  |  |  |  |  |  |
| *Tyto furcata* (Temminck, 1827) |  | 84 | 39 | 9 | 103 | 1 | **236** |  | 6, 17, 19, 21, 81, 116, 150, 163, 207, 219, 267, 277, 309, 316, 339, 345, 357, 360, 363, 375, 378, 379, 382, 383, 385, 389, 391, MCP, MHNCI, MZUEL, MZUSP, WA |
| Strigidae Leach, 1820 |  |  |  |  |  |  |  |  |  |
| *Megascops choliba* (Vieillot, 1817) |  | 126 | 120 | 23 | 133 | 2 | **404** |  | 5, 17, 19, 21, 46, 47, 59, 81, 87, 134, 137, 150, 181, 207, 230, 264, 277, 286, 294, 305, 306, 309, 313, 316, 357, 360, 363, 364, 369, 375, 378, 379, 382, 383, 385, 387, 389, 391, 393, 419, MHNCI, MZUEL, MZUSP, WA |
| *Megascops atricapilla* (Temminck, 1822) |  | 14 | 4 | 33 | 11 |  | **62** |  | 21, 87, 364, 369, 378, 382, 391, MHNCI, WA |
| *Megascops sanctaecatarinae* (Salvin, 1897) |  | 89 |  | 7 | 47 |  | **143** |  | 46, 81, 150, 277, 363, 378, 379, 391, 393, MHNCI, WA |
| *Pulsatrix perspicillata* (Latham, 1790) |  |  | 4 |  |  |  | **4** |  | 13, 21, 22, 313, 392 |
| *Pulsatrix koeniswaldiana* (Bertoni & Bertoni, 1901) |  | 16 | 21 | 19 | 17 | 2 | **75** |  | 13, 14, 19, 21, 22, 42, 59, 87, 118, 150, 286, 289, 294, 309, 363, 378, 379, 382, 389, 391, MHNCI, WA |
| *Bubo virginianus* (Gmelin, 1788) |  | 4 | 1 |  |  |  | **5** | **N(S)** | 21, 22, WA |
| *Strix hylophila* Temminck, 1825 |  | 27 | 8 | 9 | 63 |  | **107** |  | 13, 14, 17, 19, 21, 22, 46, 47, 81, 87, 94, 118, 137, 150, 267, 277, 294, 306, 309, 313, 316, 357, 360, 363, 364, 378, 379, 382, 387, 391, 393, MHNCI, MZUSP, WA |
| *Strix virgata* (Cassin, 1849) |  | 8 | 17 | 25 | 4 |  | **54** |  | 6, 13, 19, 21, 22, 81, 101, 134, 286, 313, 363, 378, 379, 391, MHNCI, WA |
| *Strix huhula* Daudin, 1800 |  |  | 3 |  |  |  | **3** |  | 59, 306, 324 |
| *Glaucidium minutissimum* (Wied, 1830) |  |  |  | 8 |  |  | **8** |  | 47, 48, 382, 391, 402, WA |
| *Glaucidium brasilianum* (Gmelin, 1788) |  | 8 | 47 | 5 | 31 |  | **91** |  | 13, 14, 17, 19, 21, 22, 47, 59, 94, 134, 230, 264, 306, 309, 313, 357, 375, 379, 383, 387, 391, 392, 393, 419, MHNCI, MZUSP, WA |
| *Athene cunicularia* (Molina, 1782) |  | 317 | 320 | 121 | 375 | 6 | **1139** |  | 4, 5, 6, 10, 19, 21, 22, 47, 59, 81, 110, 113, 116, 120, 134, 137, 138, 150, 154, 181, 219, 226, 246, 247, 250, 251, 267, 277, 294, 305, 306, 309, 313, 316, 322, 339, 357, 360, 363, 364, 375, 378, 379, 382, 383, 385, 389, 391, 393, 419, FMNH, MHNCI, MZUSP, WA |
| *Aegolius harrisii* (Cassin, 1849) |  | 8 |  | 1 | 14 |  | **23** |  | 277, 288, 324, 378, MHNCI, WA |
| *Asio clamator* (Vieillot, 1808) |  | 83 | 29 | 9 | 68 |  | **189** |  | 6, 10, 19, 21, 22, 54, 81, 94, 150, 170, 192, 267, 277, 306, 363, 364, 378, 382, 391, MHNCI, MZUEL, WA |
| *Asio stygius* (Wagler, 1832) |  | 18 | 1 | 18 | 32 |  | **69** |  | 21, 80, 137, 198, 207, 230, 277, 300, 305, 316, 324, 378, 382, 391, FMNH, MHNCI, MZUSP, WA |
| *Asio flammeus* (Pontoppidan, 1763) |  | 27 | 23 |  | 11 | 3 | **64** |  | 19, 21, 90, 250, 289, 309, 316, 325, 378, 383, 389, 391, 405, MHNCI, WA |
| Nyctibiiformes Yuri, Kimball, Harshman, Bowie, Braun, Chojnowski, Han, Hackett, Huddleston, Moore, Reddy, Sheldon, Steadman, Witt & Braun, 2013 |  |  |  |  |  |  |  |  |  |
| Nyctibiidae Chenu & Des Murs, 1851 |  |  |  |  |  |  |  |  |  |
| *Nyctibius aethereus* (Wied, 1820) |  |  | 4 |  |  |  | **4** |  | 21, 22, 136, 374, WA |
| *Nyctibius griseus* (Gmelin, 1789) |  | 86 | 148 | 63 | 94 | 1 | **392** |  | 5, 13, 19, 21, 22, 47, 59, 81, 87, 134, 155, 180, 181, 198, 207, 219, 264, 306, 313, 316, 360, 363, 364, 375, 378, 379, 382, 383, 385, 387, 389, 391, MHNCI, WA |
| Caprimulgiformes Ridgway, 1881 |  |  |  |  |  |  |  |  |  |
| Caprimulgidae Vigors, 1825 |  |  |  |  |  |  |  |  |  |
| *Nyctiphrynus ocellatus* (Tschudi, 1844) |  |  | 12 |  | 10 |  | **22** |  | 193, 374, WA |
| *Antrostomus rufus* (Boddaert, 1783) |  | 1 | 7 | 1 |  |  | **9** |  | 21, 22, 81, 313, 316, 375, 419 |
| *Antrostomus sericocaudatus* Cassin, 1849 |  | 1 | 3 | 5 | 7 |  | **16** | **SP** | 324, 378, 387, MHNCI, WA |
| *Lurocalis semitorquatus* (Gmelin, 1789) |  | 45 | 34 | 23 | 68 |  | **170** |  | 10, 13, 17, 19, 21, 22, 47, 54, 59, 81, 87, 118, 150, 181, 198, 207, 230, 250, 294, 305, 306, 309, 313, 316, 360, 363, 375, 378, 379, 382, 383, 385, 387, 391, MHNCI, WA |
| *Hydropsalis albicollis* (Gmelin, 1789) |  | 75 | 123 | 32 | 138 | 5 | **373** |  | 5, 19, 21, 22, 54, 59, 81, 87, 94, 116, 119, 120, 136, 137, 150, 155, 181, 198, 207, 267, 305, 306, 309, 313, 316, 357, 360, 363, 364, 375, 378, 379, 382, 383, 385, 387, 389, 391, 393, 419, MHNCI, MZUSP, WA |
| *Hydropsalis parvula* (Gould, 1837) |  | 18 | 26 | 2 | 19 |  | **65** |  | 19, 21, 22, 120, 205, 250, 296, 305, 309, 316, 364, 375, 378, 385, 391, 393, MHNCI, WA |
| *Hydropsalis anomala* (Gould, 1838) |  | 32 |  |  | 7 | 1 | **40** |  | 63, 69, 250, 287, 316, 324, 361, 378, 391, MHNCI, MZUSP, WA |
| *Hydropsalis longirostris* (Bonaparte, 1825) |  | 27 |  | 5 | 6 |  | **38** |  | 5, 175, 185, 289, 291, 309, 316, 324, 361, 363, 391, MHNCI, WA |
| *Hydropsalis torquata* (Gmelin, 1789) |  | 11 | 8 | 14 | 8 | 6 | **47** |  | 21, 81, 306, 309, 313, 378, 379, 382, 389, 391, MHNCI, WA |
| *Hydropsalis forcipata* (Nitzsch, 1840) |  | 12 | 3 | 17 | 66 |  | **98** |  | 5, 19, 21, 22, 27, 41, 94, 134, 150, 157, 219, 227, 255, 258, 277, 289, 306, 309, 313, 316, 357, 359, 363, 367, 378, 379, 382, 383, 391, MHNCI, WA |
| *Chordeiles nacunda* (Vieillot, 1817) |  | 43 | 27 | 11 | 34 | 1 | **116** |  | 19, 21, 22, 87, 134, 250, 309, 316, 363, 375, 378, 379, 382, 383, 419, MHNCI, MZUSP, WA |
| *Chordeiles minor* (Forster, 1771) |  |  | 7 |  | 1 |  | **8** |  | 21, 22, 181, 379, MHNCI, WA |
| *Chordeiles acutipennis* (Hermann, 1783) |  | 1 | 7 | 19 | 3 | 2 | **32** |  | 21, 22, 62, 207, 325, WA |
| Apodiformes Peters, 1940 |  |  |  |  |  |  |  |  |  |
| Apodidae Olphe-Galliard, 1887 |  |  |  |  |  |  |  |  |  |
| *Cypseloides fumigatus* (Streubel, 1848) |  | 13 | 2 | 10 | 10 | 2 | **37** |  | 19, 21, 250, 261, 309, 316, 325, 378, 379, MHNCI, MZUSP, WA |
| *Cypseloides senex* (Temminck, 1826) |  | 14 | 76 |  | 23 | 3 | **116** |  | 21, 294, 309, 316, 325, 379, 387, 389, 391, MHNCI, MZUSP, WA |
| *Streptoprocne zonaris* (Shaw, 1796) |  | 146 | 26 | 43 | 112 | 2 | **329** |  | 5, 10, 19, 20, 21, 23, 47, 59, 81, 134, 137, 150, 161, 198, 207, 219, 246, 259, 293, 305, 306, 309, 313, 316, 357, 360, 363, 364, 369, 375, 378, 379, 382, 385, 389, 391, 419, FMNH, MHNCI, MZUSP, WA |
| *Streptoprocne biscutata* (Sclater, 1866) |  | 43 | 3 | 9 | 17 |  | **72** |  | 5, 39, 104, 256, 257, 259, 262, 263, 309, 316, 378, 391, FMNH, MHNCI, MZUSP, WA |
| *Chaetura cinereiventris* Sclater, 1862 |  | 82 | 16 | 36 | 93 | 1 | **228** |  | 19, 21, 22, 47, 59, 81, 150, 155, 259, 306, 309, 313, 316, 357, 360, 363, 378, 379, 382, 389, 391, MHNCI, WA |
| *Chaetura meridionalis* Hellmayr, 1907 |  | 151 | 48 | 28 | 93 | 1 | **321** |  | 10, 19, 21, 22, 59, 137, 150, 160, 161, 181, 198, 207, 219, 230, 294, 306, 360, 363, 364, 375, 378, 379, 382, 383, 387, 391, 405, 419, MHNCI, WA |
| *Panyptila cayennensis* (Gmelin, 1789) |  |  |  | 23 | 1 |  | **24** | **SP** | 150, 259, 391, WA |
| Trochilidae Vigors, 1825 |  |  |  |  |  |  |  |  |  |
| Phaethornithinae Jardine, 1833 |  |  |  |  |  |  |  |  |  |
| *Ramphodon naevius* (Dumont, 1818) |  |  |  | 199 | 1 |  | **200** |  | 47, 48, 71, 81, 137, 173, 197, 208, 254, 360, 363, 382, 391, MHNCI, MZUSP, WA |
| [*Glaucis hirsutus*] (Gmelin, 1788) |  |  |  | 3 |  |  | **3** |  | 55, 391 |
| *Phaethornis squalidus* (Temminck, 1822) |  | 5 | 12 | 58 | 4 |  | **79** |  | 19, 21, 59, 81, 134, 193, 197, 254, 306, 360, 363, 378, 379, 382, 383, 387, 391, 392, 421, MHNCI, WA |
| *Phaethornis pretrei* (Lesson & Delattre, 1839) |  | 12 | 86 |  | 15 | 1 | **114** |  | 14, 17, 19, 21, 47, 119, 120, 155, 181, 203, 204, 294, 306, 309, 313, 357, 375, 389, 391, 419, 421, MHNCI, MZUEL, WA |
| *Phaethornis eurynome* (Lesson, 1832) |  | 42 | 40 | 43 | 52 | 1 | **178** |  | 13, 14, 21, 47, 48, 81, 87, 119, 134, 137, 150, 181, 197, 306, 309, 313, 357, 360, 363, 364, 378, 379, 382, 383, 385, 387, 391, 393, MHNCI, MZUSP, WA |
| Trochilinae Vigors, 1825 |  |  |  |  |  |  |  |  |  |
| *Eupetomena macroura* (Gmelin, 1788) |  | 168 | 194 | 56 | 107 | 10 | **535** |  | 5, 21, 59, 81, 102, 116, 118, 155, 181, 197, 203, 204, 309, 316, 325, 363, 378, 382, 389, 390, 391, 421, MHNCI, WA |
| *Aphantochroa cirrochloris* (Vieillot, 1818) |  |  | 2 | 211 | 2 |  | **215** |  | 5, 81, 134, 137, 197, 207, 254, 360, 363, 378, 382, 391, MHNCI, WA |
| *Florisuga fusca* (Vieillot, 1817) |  | 74 | 68 | 96 | 82 |  | **320** |  | 5, 10, 21, 43, 47, 54, 59, 81, 87, 102, 119, 134, 137, 142, 145, 155, 170, 173, 181, 197, 198, 202, 203, 204, 219, 230, 246, 252, 254, 306, 313, 357, 360, 363, 364, 378, 382, 383, 385, 391, 419, 421, FMNH, MHNCI, WA |
| *Colibri serrirostris* (Vieillot, 1816) |  | 125 | 15 |  | 99 | 7 | **246** |  | 5, 10, 17, 19, 20, 21, 93, 102, 150, 170, 252, 305, 306, 309, 316, 363, 364, 378, 379, 389, 391, 393, 419, 421, FMNH, MHNCI, MZUSP, WA |
| *Anthracothorax nigricollis* (Vieillot, 1817) |  | 74 | 119 | 94 | 88 | 1 | **376** |  | 13, 14, 21, 47, 81, 102, 134, 155, 181, 197, 204, 254, 363, 364, 375, 378, 379, 382, 383, 389, 391, 393, 419, 421, FMNH, MHNCI, WA |
| [*Chrysolampis mosquitus*] (Linnaeus, 1758) |  |  | 4 | 1 |  |  | **5** |  | 21, 324, 419, 421 |
| *Stephanoxis lalandi* (Vieillot, 1818) |  | 64 | 15 | 10 | 114 |  | **203** |  | 17, 19, 21, 22, 46, 93, 102, 134, 138, 150, 161, 170, 194, 205, 250, 284, 304, 305, 306, 309, 316, 357, 360, 363, 364, 378, 379, 382, 383, 385, 391, 393, 421, FMNH, MHNCI, MZUSP, WA |
| *Lophornis magnificus* (Vieillot, 1817) |  | 5 |  | 1 |  |  | **6** |  | 363, FMNH, MZUSP |
| *Lophornis chalybeus* (Vieillot, 1822) |  | 2 |  | 117 |  |  | **119** |  | 81, 197, 254, 363, 382, 391, MHNCI, WA |
| *Chlorostilbon lucidus* (Shaw, 1812) |  | 329 | 189 | 18 | 307 | 10 | **853** |  | 5, 10, 13, 17, 19, 20, 21, 22, 23, 36, 47, 54, 59, 81, 93, 94, 102, 112, 134, 137, 150, 155, 170, 181, 194, 197, 204, 219, 246, 294, 305, 306, 309, 313, 316, 322, 357, 363, 364, 375, 378, 379, 382, 383, 385, 389, 391, 392, 393, 419, 421, FMNH, MHNCI, MZUSP, WA |
| *Thalurania furcata* (Gmelin, 1788) |  | 2 |  |  |  |  | **2** |  | 324, MZUSP |
| *Thalurania glaucopis* (Gmelin, 1788) |  | 104 | 111 | 229 | 88 | 2 | **534** |  | 14, 17, 19, 21, 22, 46, 47, 59, 71, 81, 92, 102, 134, 137, 150, 173, 181, 197, 198, 208, 219, 230, 252, 254, 305, 306, 309, 313, 316, 322, 360, 363, 364, 369, 378, 379, 382, 387, 389, 391, 393, 414, 419, FMNH, MHNCI, MZUSP, WA |
| *Hylocharis sapphirina* (Gmelin, 1788) |  | 1 | 5 |  |  |  | **6** |  | 18, 21, 22, 355 |
| [*Hylocharis cyanus*] (Vieillot, 1818) |  |  | 2 |  | 2 |  | **4** |  | 18, 21, 379 |
| *Hylocharis chrysura* (Shaw, 1812) |  | 2 | 229 | 1 | 33 | 2 | **267** |  | 21, 22, 112, 120, 155, 181, 202, 203, 204, 264, 294, 306, 313, 375, 383, 385, 391, 393, 419, 421, MCP, MHNCI, MZUSP, WA |
| *Leucochloris albicollis* (Vieillot, 1818) |  | 390 | 41 | 20 | 392 | 2 | **845** |  | 5, 10, 13, 14, 17, 19, 20, 21, 22, 46, 47, 54, 93, 94, 102, 134, 150, 161, 170, 181, 190, 194, 197, 198, 203, 204, 230, 250, 252, 305, 306, 309, 316, 322, 357, 360, 363, 364, 378, 379, 382, 383, 389, 391, 393, 414, 421, FMNH, MHNCI, MZUSP, WA |
| *Polytmus guainumbi* (Pallas, 1764) |  | 1 | 1 |  |  |  | **2** | **SP** | 326, WA |
| *Amazilia versicolor* (Vieillot, 1818) |  | 18 | 61 | 126 | 37 |  | **242** |  | 19, 21, 81, 102, 134, 136, 181, 194, 197, 198, 207, 219, 230, 246, 250, 252, 254, 264, 360, 363, 378, 382, 391, 392, 393, 419, MHNCI, MZUSP, WA |
| *Amazilia fimbriata* (Gmelin, 1788) |  | 2 | 4 | 89 | 3 |  | **98** |  | 13, 21, 59, 134, 137, 254, 306, 309, 360, 363, 364, 378, 382, 391, WA |
| *Amazilia lactea* (Lesson, 1832) |  | 10 | 84 | 9 | 18 | 3 | **124** |  | 21, 59, 155, 181, 203, 204, 325, 364, 391, 419, 421, MHNCI, MZUSP, WA |
| *Clytolaema rubricauda* (Boddaert, 1783) |  | 74 |  | 47 | 14 |  | **135** |  | 47, 81, 102, 149, 150, 197, 360, 363, 378, 382, 391, MHNCI, WA |
| *Heliothryx auritus* (Gmelin, 1788) |  |  |  | 11 |  |  | **11** |  | 382, 402, MHNCI, WA |
| [*Heliomaster longirostris*] (Audebert & Vieillot, 1801) |  |  | 2 |  |  |  | **2** |  | 324, 374 |
| *Heliomaster squamosus* (Temminck, 1823) |  |  | 7 |  | 1 |  | **8** | **SP** | 21, 181, 203, 204, 419, WA |
| *Heliomaster furcifer* (Shaw, 1812) |  |  | 23 |  | 10 |  | **33** |  | 421, WA |
| *Calliphlox amethystina* (Boddaert, 1783) |  | 54 | 3 | 8 | 67 |  | **132** |  | 19, 21, 22, 93, 94, 102, 134, 150, 250, 309, 316, 363, 364, 378, 379, 382, 391, 419, 421, FMNH, MHNCI, WA |
| Trogoniformes A. O. U., 1886 |  |  |  |  |  |  |  |  |  |
| Trogonidae Lesson, 1828 |  |  |  |  |  |  |  |  |  |
| *Trogon viridis* Linnaeus, 1766 |  |  |  | 229 |  |  | **229** |  | 47, 48, 81, 98, 137, 198, 219, 230, 360, 363, 382, 391, MHNCI, MZUSP, WA |
| *Trogon surrucura* Vieillot, 1817 |  | 158 | 282 | 40 | 307 | 3 | **790** |  | 5, 13, 14, 15, 17, 19, 21, 22, 23, 46, 59, 81, 87, 94, 134, 136, 150, 194, 198, 258, 264, 305, 306, 309, 313, 316, 357, 360, 363, 364, 369, 375, 378, 379, 382, 383, 385, 387, 389, 391, 393, 414, 419, FMNH, MHNCI, MZUSP, WA |
| *Trogon rufus* Gmelin, 1788 |  | 52 | 63 | 36 | 58 |  | **209** |  | 5, 14, 17, 19, 21, 22, 47, 137, 149, 150, 195, 230, 306, 309, 313, 360, 363, 367, 378, 379, 382, 387, 391, 393, 414, MHNCI, WA |
| Coraciiformes Forbes, 1844 |  |  |  |  |  |  |  |  |  |
| Alcedinidae Rafinesque, 1815 |  |  |  |  |  |  |  |  |  |
| *Megaceryle torquata* (Linnaeus, 1766) |  | 97 | 110 | 65 | 180 | 4 | **456** |  | 21, 22, 23, 46, 54, 74, 81, 87, 94, 120, 134, 137, 150, 155, 161, 181, 194, 198, 207, 219, 226, 230, 250, 264, 294, 305, 306, 309, 313, 316, 322, 357, 360, 363, 364, 369, 375, 378, 379, 382, 383, 385, 387, 389, 391, 405, 419, MHNCI, MZUEL, MZUSP, WA |
| *Chloroceryle amazona* (Latham, 1790) |  | 99 | 157 | 39 | 171 | 1 | **467** |  | 21, 22, 23, 46, 74, 81, 87, 94, 120, 134, 136, 137, 138, 150, 155, 181, 194, 198, 207, 219, 250, 264, 305, 306, 309, 313, 316, 322, 357, 360, 363, 369, 375, 378, 379, 382, 383, 385, 387, 389, 391, 405, FMNH, MHNCI, MZUSP, WA |
| *Chloroceryle aenea* (Pallas, 1764) |  |  |  | 14 |  |  | **14** |  | 81, 88, 137, 207, 311, 360, 371, 382, MHNCI, WA |
| *Chloroceryle americana* (Gmelin, 1788) |  | 93 | 121 | 51 | 150 | 1 | **416** |  | 10, 19, 21, 22, 46, 59, 74, 81, 87, 94, 120, 134, 136, 138, 150, 155, 161, 181, 207, 219, 230, 264, 305, 306, 309, 313, 316, 322, 357, 360, 363, 364, 375, 378, 379, 382, 383, 387, 389, 391, FMNH, MHNCI, MZUSP, WA |
| *Chloroceryle inda* (Linnaeus, 1766) |  |  |  | 12 |  |  | **12** |  | 81, 88, 311, 371, 382, 391, MHNCI, WA |
| Momotidae Gray, 1840 |  |  |  |  |  |  |  |  |  |
| *Baryphthengus ruficapillus* (Vieillot, 1818) |  | 24 | 163 | 10 | 62 |  | **259** |  | 13, 14, 15, 21, 24, 47, 59, 118, 134, 136, 138, 155, 184, 264, 306, 309, 313, 369, 375, 378, 379, 382, 383, 385, 387, 391, 392, 393, 414, FMNH, MHNCI, MZUEL, MZUSP, WA |
| *Momotus momota* (Linnaeus, 1766) |  |  | 6 |  |  |  | **6** |  | 310, 324, 374, MHNCI, WA |
| Galbuliformes Fürbringer, 1888 |  |  |  |  |  |  |  |  |  |
| Galbulidae Vigors, 1825 |  |  |  |  |  |  |  |  |  |
| *Jacamaralcyon tridactyla* (Vieillot, 1817) |  |  | 11 |  |  |  | **11** |  | 136, 138, 264, 392, 393, MHNCI, MZUSP |
| *Galbula ruficauda* Cuvier, 1816 |  |  | 13 |  |  |  | **13** |  | 21, 120, 136, 264, 310, 392, MZUSP, WA |
| Bucconidae Horsfield, 1821 |  |  |  |  |  |  |  |  |  |
| *Notharchus swainsoni* (Gray, 1846) |  |  | 35 | 30 | 9 |  | **74** |  | 13, 14, 21, 47, 81, 134, 137, 138, 235, 294, 306, 313, 363, 369, 375, 382, 391, 392, 393, MHNCI, WA |
| *Nystalus chacuru* (Vieillot, 1816) |  | 38 | 44 |  | 136 | 3 | **221** |  | 4, 21, 23, 134, 136, 138, 205, 267, 309, 313, 316, 357, 363, 364, 375, 379, 383, 385, 389, 391, 393, 419, FMNH, MHNCI, MZUSP, WA |
| *Malacoptila striata* (Spix, 1824) |  | 2 | 12 | 79 | 23 |  | **116** |  | 47, 81, 134, 136, 137, 138, 264, 309, 360, 363, 364, 369, 382, 391, 392, 393, FMNH, MHNCI, MZUSP, WA |
| *Nonnula rubecula* (Spix, 1824) |  | 8 | 52 | 14 | 58 |  | **132** |  | 21, 47, 49, 94, 95, 119, 264, 306, 364, 379, 382, 391, 393, MHNCI, MZUSP, WA |
| Piciformes Meyer & Wolf, 1810 |  |  |  |  |  |  |  |  |  |
| Ramphastidae Vigors, 1825 |  |  |  |  |  |  |  |  |  |
| *Ramphastos toco* Statius Muller, 1776 |  |  | 109 |  |  |  | **109** |  | 23, 138, 264, 267, 309, 313, 369, 387, 391, 393, MHNCI, MZUSP, WA |
| *Ramphastos vitellinus* Lichtenstein, 1823 |  |  |  | 102 |  |  | **102** |  | 47, 81, 83, 84, 98, 286, 341, 342, 360, 363, 382, 391, MHNCI, WA |
| *Ramphastos dicolorus* Linnaeus, 1766 |  | 193 | 145 | 89 | 315 | 3 | **745** |  | 5, 13, 14, 17, 19, 21, 22, 47, 73, 81, 94, 123, 134, 136, 137, 150, 162, 194, 205, 250, 258, 264, 286, 294, 305, 309, 316, 341, 342, 357, 360, 363, 364, 369, 378, 379, 382, 383, 385, 387, 389, 391, 392, 393, 414, FMNH, MHNCI, MZUSP, WA |
| *Selenidera maculirostris* (Lichtenstein, 1823) |  |  | 195 | 79 | 9 |  | **283** |  | 13, 14, 15, 21, 47, 59, 81, 83, 84, 85, 98, 136, 137, 181, 209, 210, 286, 306, 341, 342, 344, 363, 369, 379, 382, 383, 385, 387, 391, 414, 419, MHNCI, MZUSP, WA |
| *Pteroglossus bailloni* (Vieillot, 1819) |  | 10 | 42 | 32 | 53 |  | **137** |  | 13, 14, 19, 21, 22, 41, 47, 52, 59, 134, 136, 138, 195, 205, 209, 264, 286, 294, 306, 341, 342, 360, 363, 378, 382, 383, 391, 393, 414, FMNH, MCN-FZB, MHNCI, MZUSP, WA |
| *Pteroglossus aracari* (Linnaeus, 1758) |  |  | 17 |  |  |  | **17** |  | 13, 14, 21, 136, 209, 210, 294, 306, 375, 383, 391, 392, MZUSP, WA |
| *Pteroglossus castanotis* Gould, 1834 |  |  | 210 |  | 33 | 1 | **244** |  | 152, 264, 268, 294, 313, 369, 375, 383, 387, 391, MHNCI, MZUSP, WA |
| Picidae Leach, 1820 |  |  |  |  |  |  |  |  |  |
| *Picumnus cirratus* Temminck, 1825 |  | 5 | 47 |  | 3 | 5 | **60** |  | 13, 23, 120, 136, 375, 389, 391, FMNH, MCP, MHNCI, MZUSP, WA |
| *Picumnus temminckii* Lafresnaye, 1845 |  | 306 | 148 | 139 | 378 | 2 | **973** |  | 5, 13, 14, 17, 19, 21, 23, 46, 47, 54, 59, 81, 94, 108, 119, 134, 137, 150, 155, 161, 170, 173, 176, 181, 194, 206, 207, 230, 279, 294, 305, 306, 309, 313, 316, 322, 351, 357, 360, 363, 364, 378, 379, 382, 383, 385, 387, 391, 393, 405, 414, MHNCI, MZUEL, MZUSP, WA |
| *Picumnus albosquamatus* d'Orbigny, 1840 |  |  | 51 |  |  |  | **51** |  | 13, 120, 264, 313, 375, 383, 391, MCP, MHNCI, MZUSP, WA |
| *Picumnus nebulosus* Sundevall, 1866 |  | 37 | 3 | 2 | 98 |  | **140** |  | 12, 14, 17, 19, 21, 42, 95, 108, 190, 289, 305, 306, 309, 316, 357, 363, 364, 378, 379, 391, 393, 419, MHNCI, WA |
| *Melanerpes candidus* (Otto, 1796) |  | 138 | 185 | 12 | 166 | 5 | **506** |  | 5, 13, 17, 21, 22, 23, 59, 116, 120, 129, 150, 155, 181, 194, 205, 207, 250, 286, 306, 309, 313, 316, 322, 357, 363, 364, 369, 375, 378, 379, 382, 383, 385, 387, 389, 391, 419, MHNCI, WA |
| *Melanerpes flavifrons* (Vieillot, 1818) |  | 30 | 201 | 54 | 154 |  | **439** |  | 13, 14, 15, 17, 19, 21, 22, 23, 24, 47, 59, 81, 94, 120, 134, 136, 137, 155, 181, 250, 264, 286, 294, 306, 309, 313, 316, 351, 357, 360, 363, 375, 378, 379, 382, 383, 385, 387, 391, 392, 393, 414, FMNH, MHNCI, MZUSP, WA |
| *Veniliornis passerinus* (Linnaeus, 1766) |  |  | 36 |  |  |  | **36** |  | 120, 264, 313, 324, 374, 375, 383, 391, MHNCI, MZUSP, WA |
| *Veniliornis spilogaster* (Wagler, 1827) |  | 307 | 120 | 44 | 367 | 6 | **844** |  | 5, 10, 13, 14, 17, 19, 20, 21, 22, 46, 47, 54, 59, 81, 94, 119, 134, 137, 150, 155, 161, 170, 176, 181, 190, 194, 198, 207, 219, 250, 264, 286, 305, 306, 309, 313, 316, 322, 351, 357, 360, 363, 364, 375, 378, 379, 382, 383, 385, 387, 389, 391, 392, 393, 414, FMNH, MHNCI, MZUSP, WA |
| *Piculus flavigula* (Boddaert, 1783) |  | 1 | 1 | 42 |  |  | **44** |  | 47, 81, 134, 286, 363, 382, 391, 392, MHNCI, WA |
| *Piculus aurulentus* (Temminck, 1821) |  | 90 | 7 | 14 | 153 |  | **264** |  | 5, 13, 14, 17, 19, 21, 22, 46, 54, 134, 150, 161, 190, 250, 286, 289, 305, 309, 316, 351, 357, 360, 363, 364, 378, 379, 382, 387, 391, 393, 414, FMNH, MHNCI, MZUSP, WA |
| *Colaptes melanochloros* (Gmelin, 1788) |  | 189 | 202 | 29 | 244 | 3 | **667** |  | 13, 14, 15, 17, 19, 21, 22, 23, 35, 47, 59, 81, 94, 118, 120, 134, 136, 137, 140, 150, 170, 181, 194, 250, 258, 267, 294, 305, 306, 309, 313, 316, 322, 351, 357, 363, 364, 369, 375, 378, 379, 382, 383, 385, 387, 389, 391, 392, 414, 419, FMNH, MHNCI, MZUSP, NMNH, WA |
| *Colaptes campestris* (Vieillot, 1818) |  | 423 | 240 | 53 | 515 | 9 | **1240** |  | 5, 10, 17, 19, 21, 22, 23, 46, 54, 59, 81, 94, 116, 120, 134, 137, 138, 143, 150, 155, 161, 170, 181, 190, 194, 198, 246, 250, 267, 305, 306, 309, 313, 316, 322, 357, 360, 363, 364, 369, 375, 378, 379, 382, 383, 385, 389, 391, 393, 405, 419, FMNH, MHNCI, MZUSP, WA |
| *Celeus flavescens* (Gmelin, 1788) |  | 9 | 166 | 128 | 18 | 3 | **324** |  | 13, 14, 21, 22, 47, 59, 81, 134, 136, 137, 138, 155, 181, 264, 286, 294, 306, 309, 313, 351, 360, 363, 364, 369, 375, 382, 383, 385, 387, 389, 391, 392, 393, FMNH, MHNCI, MZUSP, WA |
| *Dryocopus galeatus* (Temminck, 1822) |  | 2 | 6 | 3 | 33 |  | **44** |  | 101, 134, 136, 138, 195, 264, 324, 368, 391, 414, FMNH, MHNCI, MZUSP, WA |
| *Dryocopus lineatus* (Linnaeus, 1766) |  | 90 | 197 | 32 | 152 | 6 | **477** |  | 13, 14, 17, 19, 21, 22, 23, 35, 47, 59, 73, 81, 87, 119, 134, 136, 137, 138, 150, 155, 161, 181, 194, 198, 206, 207, 219, 258, 264, 286, 306, 309, 313, 316, 351, 357, 360, 363, 364, 369, 375, 378, 379, 382, 383, 385, 387, 389, 391, 392, 393, 414, 419, FMNH, MHNCI, MZUSP, WA |
| *Campephilus robustus* (Lichtenstein, 1818) |  | 27 | 43 | 88 | 87 | 1 | **246** |  | 17, 19, 21, 27, 41, 47, 59, 81, 94, 134, 136, 137, 157, 198, 222, 230, 258, 268, 286, 309, 313, 316, 357, 360, 363, 364, 369, 378, 379, 382, 387, 389, 391, 392, 393, 414, FMNH, MHNCI, MZUSP, WA |
| [*Campephilus melanoleucos*] (Gmelin, 1788) |  | 2 | 2 |  |  |  | **4** |  | 13, 14, 21, 310, 313 |
| Cariamiformes Furbringer, 1888 |  |  |  |  |  |  |  |  |  |
| Cariamidae Bonaparte, 1850 |  |  |  |  |  |  |  |  |  |
| *Cariama cristata* (Linnaeus, 1766) |  | 25 | 23 |  | 9 | 9 | **66** |  | 19, 21, 59, 193, 309, 316, 325, 387, 389, 391, MHNCI, WA |
| Falconiformes Bonaparte, 1831 |  |  |  |  |  |  |  |  |  |
| Falconidae Leach, 1820 |  |  |  |  |  |  |  |  |  |
| *Ibycter americanus* (Boddaert, 1783) |  |  | 3 |  |  |  | **3** |  | 324, 369, 392, MHNCI |
| *Caracara plancus* (Miller, 1777) |  | 413 | 230 | 73 | 411 | 10 | **1137** |  | 5, 10, 17, 19, 20, 21, 46, 59, 81, 94, 113, 116, 120, 134, 137, 138, 142, 150, 155, 161, 170, 181, 194, 198, 207, 219, 226, 230, 246, 249, 305, 306, 309, 313, 316, 322, 357, 360, 363, 364, 375, 378, 379, 382, 383, 385, 387, 389, 391, 397, 405, 419, MHNCI, MZUEL, WA |
| *Milvago chimachima* (Vieillot, 1816) |  | 217 | 154 | 132 | 348 | 9 | **860** |  | 5, 10, 17, 19, 21, 23, 46, 54, 81, 94, 113, 120, 131, 134, 136, 137, 150, 161, 170, 181, 194, 198, 207, 219, 223, 226, 230, 246, 249, 271, 305, 306, 309, 313, 316, 322, 357, 360, 363, 364, 375, 378, 379, 382, 383, 385, 387, 389, 391, 405, 414, 419, FMNH, MHNCI, MZUSP, WA |
| *Milvago chimango* (Vieillot, 1816) |  | 33 |  | 6 | 26 |  | **65** |  | 198, 219, 226, 309, 391, 406, MHNCI, WA |
| *Herpetotheres cachinnans* (Linnaeus, 1758) |  | 12 | 29 | 23 | 13 | 2 | **79** |  | 13, 14, 17, 19, 21, 23, 81, 87, 219, 306, 309, 313, 375, 378, 382, 383, 389, 391, WA |
| *Micrastur ruficollis* (Vieillot, 1817) |  | 25 | 18 | 24 | 45 |  | **112** |  | 13, 14, 17, 19, 21, 46, 47, 81, 87, 94, 134, 136, 150, 207, 219, 249, 264, 286, 309, 313, 316, 363, 364, 378, 379, 382, 383, 387, 391, 393, 414, MHNCI, MZUSP, WA |
| *Micrastur semitorquatus* (Vieillot, 1817) |  | 13 | 30 | 10 | 26 |  | **79** |  | 5, 13, 14, 21, 47, 136, 249, 258, 284, 286, 306, 309, 313, 357, 360, 363, 364, 369, 375, 378, 379, 382, 385, 387, 391, 407, 408, 414, MHNCI, MZUSP, WA |
| *Falco sparverius* Linnaeus, 1758 |  | 194 | 192 | 8 | 324 | 5 | **723** |  | 4, 5, 10, 17, 19, 21, 23, 54, 81, 94, 116, 134, 136, 138, 150, 181, 205, 249, 290, 294, 305, 306, 309, 313, 316, 322, 357, 364, 369, 375, 378, 379, 382, 383, 385, 389, 391, 393, 419, MHNCI, MZUSP, WA |
| *Falco rufigularis* Daudin, 1800 |  | 1 | 28 |  | 11 |  | **40** |  | 23, 94, 123, 206, 313, 375, 379, 393, MHNCI, WA |
| *Falco femoralis* Temminck, 1822 |  | 56 | 81 | 8 | 60 | 4 | **209** |  | 17, 19, 21, 134, 150, 181, 219, 249, 305, 309, 313, 316, 364, 369, 375, 378, 379, 383, 385, 387, 389, 391, MCP, MHNCI, MZUSP, WA |
| *Falco peregrinus* Tunstall, 1771 |  | 27 | 66 | 1 | 18 | 1 | **113** |  | 19, 21, 23, 149, 150, 181, 226, 249, 309, 316, 375, 378, 383, 391, MHNCI, WA |
| Psittaciformes Wagler, 1830 |  |  |  |  |  |  |  |  |  |
| Psittacidae Rafinesque, 1815 |  |  |  |  |  |  |  |  |  |
| *Ara ararauna* (Linnaeus, 1758) |  | 2 | 28 |  | 1 |  | **31** |  | 313, 324, 327, 365, WA |
| *Ara chloropterus* Gray, 1859 |  | 2 | 66 |  |  |  | **68** |  | 136, 138, 250, 264, 294, 310, 313, 327, 357, 365, 369, 375, 393, AMNH, MHNCI, MZUSP, WA |
| *Primolius maracana* (Vieillot, 1816) |  | 2 | 18 |  | 16 |  | **36** |  | 21, 22, 64, 94, 134, 250, 310, 313, 334, 369, 375, 379, 383, 392, 393, FMNH, MHNCI, MZUSP, WA |
| *Psittacara leucophthalmus* (Statius Muller, 1776) |  | 7 | 208 |  | 62 | 1 | **278** |  | 13, 14, 15, 21, 22, 23, 43, 59, 94, 116, 119, 120, 134, 136, 155, 181, 264, 294, 306, 313, 334, 357, 375, 379, 383, 385, 387, 391, 392, 393, FMNH, MHNCI, MZUSP, WA |
| *Aratinga nenday* (Vieillot, 1823) |  |  | 3 |  |  |  | **3** | **N(S), C** | WA |
| *Aratinga auricapillus* (Kuhl, 1820) |  | 1 | 65 |  | 28 |  | **94** |  | 13, 14, 15, 21, 22, 134, 136, 138, 181, 324, 334, 335, 357, 368, 379, 391, 392, FMNH, MHNCI, MZUSP, WA |
| *Eupsittula aurea* (Gmelin, 1788) |  | 1 | 23 |  |  |  | **24** |  | 21, 22, 264, 310, 313, 374, 375, MHNCI, MZUSP, WA |
| *Pyrrhura frontalis* (Vieillot, 1817) |  | 165 | 148 | 56 | 242 | 4 | **615** |  | 5, 10, 13, 14, 15, 17, 19, 21, 22, 23, 43, 46, 47, 54, 59, 81, 94, 119, 134, 137, 150, 170, 181, 190, 194, 250, 258, 264, 267, 286, 294, 304, 305, 306, 309, 313, 316, 334, 335, 357, 360, 363, 364, 369, 375, 378, 379, 382, 383, 385, 387, 389, 391, 392, 414, 419, FMNH, MHNCI, MZUSP, WA |
| *Myiopsitta monachus* (Boddaert, 1783) |  | 3 | 29 |  | 12 |  | **44** | **N(S), C** | 391, WA |
| *Forpus xanthopterygius* (Spix, 1824) |  | 18 | 82 | 115 | 17 | 1 | **233** |  | 13, 14, 17, 19, 21, 22, 23, 47, 59, 81, 120, 134, 137, 181, 207, 230, 264, 294, 306, 313, 334, 360, 363, 375, 378, 382, 387, 391, 392, MHNCI, MZUSP, WA |
| *Brotogeris tirica* (Gmelin, 1788) |  | 537 | 19 | 142 | 276 |  | **974** |  | 10, 13, 14, 17, 19, 20, 21, 22, 47, 48, 54, 59, 81, 96, 97, 134, 136, 137, 170, 230, 250, 286, 309, 316, 322, 334, 335, 360, 363, 378, 382, 391, 392, FMNH, MHNCI, WA |
| *Brotogeris chiriri* (Vieillot, 1818) |  |  | 99 |  | 3 |  | **102** |  | 23, 306, 313, 375, 383, 391, MHNCI, WA |
| *Touit melanonotus* (Wied, 1820) |  |  |  | 5 |  |  | **5** | **SP** | 391, WA |
| *Pionopsitta pileata* (Scopoli, 1769) |  | 69 | 29 | 32 | 48 | 1 | **179** |  | 13, 14, 17, 19, 21, 22, 27, 38, 40, 41, 47, 52, 54, 59, 81, 134, 138, 150, 181, 258, 267, 286, 294, 306, 309, 313, 316, 317, 318, 334, 335, 357, 360, 363, 378, 379, 382, 387, 389, 391, MHNCI, MZUSP, WA |
| *Pionus maximiliani* (Kuhl, 1820) |  | 86 | 194 | 68 | 158 | 1 | **507** |  | 5, 13, 14, 15, 17, 19, 21, 22, 24, 43, 46, 47, 59, 81, 94, 119, 120, 134, 136, 137, 150, 155, 181, 194, 207, 230, 258, 264, 294, 305, 306, 309, 313, 316, 334, 335, 357, 360, 363, 364, 369, 375, 378, 379, 382, 383, 385, 387, 389, 391, 392, 414, 419, FMNH, MHNCI, MZUSP, WA |
| *Amazona vinacea* (Kuhl, 1820) |  | 37 | 4 | 10 | 63 | 2 | **116** |  | 14, 17, 19, 21, 27, 59, 103, 149, 150, 157, 175, 250, 258, 309, 378, 379, 385, 389, 391, 402, FMNH, MHNCI, MZUSP, WA |
| *Amazona brasiliensis* (Linnaeus, 1758) |  |  |  | 156 |  |  | **156** |  | 50, 51, 66, 81, 88, 98, 106, 107, 191, 198, 207, 219, 230, 250, 286, 299, 303, 329, 346, 347, 348, 349, 350, 360, 382, 391, 404, MHNCI, WA |
| *Amazona amazonica* (Linnaeus, 1766) |  |  | 2 | 1 |  |  | **3** | **SP** | 294, 313, WA |
| *Amazona aestiva* (Linnaeus, 1758) |  | 182 | 85 | 3 | 112 | 4 | **386** |  | 13, 14, 17, 19, 21, 22, 23, 130, 155, 181, 250, 264, 267, 294, 309, 313, 334, 357, 364, 369, 375, 378, 379, 383, 389, 391, 419, MHNCI, MZUSP, WA |
| *Triclaria malachitacea* (Spix, 1824) |  | 4 |  | 24 | 2 |  | **30** |  | 47, 48, 81, 286, 324, 335, 378, 380, 382, 402, MHNCI, WA |
| Passeriformes Linnaeus, 1758 |  |  |  |  |  |  |  |  |  |
| Tyranni Wetmore & Miller, 1926 |  |  |  |  |  |  |  |  |  |
| Thamnophilida Patterson, 1987 |  |  |  |  |  |  |  |  |  |
| Thamnophilidae Swainson, 1824 |  |  |  |  |  |  |  |  |  |
| Thamnophilinae Swainson, 1824 |  |  |  |  |  |  |  |  |  |
| *Terenura maculata* (Wied, 1831) |  |  | 5 | 46 |  |  | **51** |  | 47, 234, 360, 363, 382, 387, 391, MHNCI, MZUSP, WA |
| *Myrmotherula unicolor* (Ménétriès, 1835) |  |  |  | 91 |  |  | **91** |  | 47, 81, 134, 137, 230, 363, 382, 391, MCP, MHNCI, MZUSP, WA |
| *Formicivora rufa* (Wied, 1831) |  |  | 5 |  |  |  | **5** |  | 313, MCN-FZB, MHNCI, WA |
| *Stymphalornis acutirostris* Bornschein, Reinert & Teixeira, 1995 |  |  |  | 166 |  |  | **166** |  | 273, 275, 311, 353, 391, MCP, MHNCI, WA |
| *Rhopias gularis* (Spix, 1825) |  | 20 | 4 | 60 | 5 |  | **89** |  | 5, 47, 136, 173, 196, 357, 363, 382, 391, 392, 393, MCP, MHNCI, MZUSP, WA |
| *Dysithamnus stictothorax* (Temminck, 1823) |  |  |  | 60 |  |  | **60** |  | 47, 360, 363, 382, 391, 402, MCP, MHNCI, WA |
| *Dysithamnus mentalis* (Temminck, 1823) |  | 79 | 134 | 68 | 132 | 1 | **414** |  | 13, 14, 15, 17, 19, 21, 23, 24, 29, 46, 47, 48, 59, 81, 119, 120, 134, 137, 150, 155, 160, 161, 181, 182, 194, 196, 206, 234, 250, 264, 267, 279, 305, 306, 309, 313, 316, 344, 360, 363, 364, 375, 378, 379, 382, 383, 385, 387, 389, 391, 392, 393, 414, MCP, MHNCI, MZUSP, WA |
| *Dysithamnus xanthopterus* Burmeister, 1856 |  |  |  | 21 |  |  | **21** |  | 5, 234, 324, 363, 391, MCP, MHNCI, WA |
| *Herpsilochmus atricapillus* Pelzeln, 1868 |  |  | 1 |  |  |  | **1** |  | 264, 324 |
| *Herpsilochmus longirostris* Pelzeln, 1868 |  |  | 38 |  |  |  | **38** |  | 120, 264, 324, 375, 377, 383, MHNCI, WA |
| *Herpsilochmus rufimarginatus* (Temminck, 1822) |  | 1 | 40 | 88 | 3 |  | **132** |  | 13, 14, 21, 47, 59, 81, 137, 155, 234, 264, 313, 363, 382, 383, 385, 387, 391, 392, MHNCI, WA |
| *Thamnophilus doliatus* (Linnaeus, 1764) |  | 2 | 297 |  |  | 3 | **302** |  | 13, 21, 23, 29, 59, 112, 120, 155, 181, 253, 264, 306, 313, 369, 375, 383, 391, MCP, MHNCI, WA |
| *Thamnophilus ruficapillus* Vieillot, 1816 |  | 184 | 43 | 6 | 193 | 12 | **438** |  | 10, 13, 19, 21, 23, 59, 120, 150, 161, 181, 250, 267, 309, 316, 322, 357, 360, 363, 364, 378, 379, 382, 383, 385, 389, 391, 393, 405, MHNCI, WA |
| *Thamnophilus pelzelni* Hellmayr, 1924 |  |  | 16 |  |  |  | **16** |  | 324, 374, 391, MCP, MHNCI, WA |
| *Thamnophilus caerulescens* Vieillot, 1816 |  | 295 | 197 | 125 | 437 | 11 | **1065** |  | 5, 10, 13, 14, 17, 19, 20, 21, 29, 46, 47, 54, 59, 81, 94, 100, 112, 119, 134, 137, 142, 146, 150, 155, 161, 170, 176, 177, 181, 182, 183, 190, 194, 198, 207, 219, 230, 233, 250, 264, 267, 279, 305, 306, 309, 316, 322, 344, 357, 360, 363, 364, 375, 378, 379, 382, 383, 385, 387, 389, 391, 392, 393, 414, FMNH, MCP, MHNCI, MZUEL, WA |
| *Taraba major* (Vieillot, 1816) |  |  | 18 |  |  |  | **18** |  | 264, 268, 374, 375, MCN-FZB, MHNCI, WA |
| *Hypoedaleus guttatus* (Vieillot, 1816) |  |  | 114 | 65 | 10 |  | **189** |  | 13, 14, 15, 21, 47, 59, 81, 134, 136, 137, 181, 182, 264, 306, 344, 363, 371, 379, 382, 385, 387, 391, 392, 393, MHNCI, WA |
| *Batara* *cinerea* (Vieillot, 1819) |  | 54 | 4 | 24 | 68 |  | **150** |  | 14, 17, 19, 21, 22, 46, 59, 94, 150, 182, 183, 219, 250, 309, 316, 357, 360, 363, 364, 378, 379, 382, 387, 391, 393, 414, FMNH, MHNCI, MZUSP, WA |
| *Mackenziaena leachii* (Such, 1825) |  | 36 | 20 | 11 | 87 | 4 | **158** |  | 5, 13, 14, 17, 19, 21, 22, 46, 87, 94, 134, 190, 233, 250, 306, 309, 357, 360, 363, 364, 368, 378, 379, 382, 385, 387, 389, 391, 393, 414, FMNH, MHNCI, WA |
| *Mackenziaena severa* (Lichtenstein, 1823) |  | 9 | 114 | 28 | 43 |  | **194** |  | 5, 13, 14, 19, 21, 22, 46, 59, 81, 100, 134, 138, 150, 155, 181, 182, 183, 233, 285, 306, 338, 360, 363, 364, 378, 379, 382, 383, 385, 387, 391, 393, MCP, MHNCI, MZUSP, WA |
| *Biatas nigropectus* (Lafresnaye, 1850) |  | 15 |  | 11 | 11 |  | **37** |  | 14, 16, 17, 19, 21, 22, 49, 101, 385, 391, MHNCI, WA |
| *Myrmoderus squamosus* (Pelzeln, 1868) |  | 3 | 11 | 111 | 4 |  | **129** |  | 5, 47, 81, 134, 137, 179, 360, 363, 382, 383, 387, 391, 392, MCP, MHNCI, MZUSP, WA |
| *Pyriglena leucoptera* (Vieillot, 1818) |  | 26 | 101 | 118 | 49 | 3 | **297** |  | 13, 14, 15, 17, 19, 21, 29, 47, 59, 73, 81, 87, 100, 119, 134, 137, 181, 182, 198, 219, 234, 264, 279, 285, 306, 309, 313, 357, 360, 363, 364, 379, 382, 383, 385, 387, 391, 392, 393, FMNH, MCP, MHNCI, WA |
| *Drymophila ferruginea* (Temminck, 1822) |  |  |  | 114 |  |  | **114** |  | 47, 81, 134, 363, 382, 391, MHNCI, MZUSP, WA |
| *Drymophila rubricollis* (Bertoni, 1901) |  | 23 | 32 | 12 | 48 |  | **115** |  | 10, 19, 21, 136, 182, 183, 201, 234, 285, 306, 363, 364, 379, 382, 385, 387, 391, 393, 414, MHNCI, MZUSP, WA |
| *Drymophila ochropyga* (Hellmayr, 1906) |  |  | 24 | 27 | 14 |  | **65** |  | 363, 382, 391, 393, 402, MCP, MHNCI, WA |
| *Drymophila malura* (Temminck, 1825) |  | 53 | 19 | 23 | 103 | 7 | **205** |  | 5, 13, 14, 17, 19, 21, 150, 183, 190, 194, 196, 234, 279, 285, 309, 316, 360, 363, 364, 368, 378, 379, 382, 385, 387, 389, 391, 393, 414, FMNH, MCP, MHNCI, MZUEL, MZUSP, WA |
| *Drymophila squamata* (Lichtenstein, 1823) |  |  |  | 114 | 1 |  | **115** |  | 47, 81, 134, 137, 173, 179, 196, 360, 363, 382, 391, MCP, MHNCI, MZUSP, WA |
| Conopophagidae Sclater & Salvin, 1873 |  |  |  |  |  |  |  |  |  |
| *Conopophaga lineata* (Wied, 1831) |  | 161 | 102 | 48 | 199 | 3 | **513** |  | 5, 13, 14, 17, 19, 21, 23, 29, 46, 47, 59, 81, 119, 134, 137, 150, 155, 161, 181, 183, 190, 196, 205, 219, 233, 264, 279, 285, 294, 305, 306, 309, 313, 316, 357, 360, 363, 364, 375, 378, 379, 382, 383, 385, 387, 389, 391, 392, 393, 414, MCP, MHNCI, MZUSP, WA |
| *Conopophaga melanops* (Vieillot, 1818) |  |  |  | 135 |  |  | **135** |  | 47, 81, 134, 172, 173, 178, 179, 356, 358, 360, 363, 382, 391, MHNCI, MZUSP, WA |
| Furnariida Sibley, Ahlquist & Monroe, 1988 |  |  |  |  |  |  |  |  |  |
| Grallarioidea Sclater & Salvin, 1873 |  |  |  |  |  |  |  |  |  |
| Grallariidae Sclater & Salvin, 1873 |  |  |  |  |  |  |  |  |  |
| *Grallaria varia* (Boddaert, 1783) |  | 16 | 10 | 19 | 16 |  | **61** |  | 13, 14, 21, 47, 136, 149, 150, 183, 196, 289, 306, 309, 360, 363, 364, 379, 382, 383, 387, 391, 393, MHNCI, WA |
| *Hylopezus nattereri* (Pinto, 1937) |  | 25 |  | 7 | 32 |  | **64** |  | 14, 17, 19, 21, 138, 149, 150, 183, 234, 258, 285, 289, 360, 363, 364, 379, 382, 391, 393, MHNCI, WA |
| Rhinocryptidae Wetmore, 1926 (1837) |  |  |  |  |  |  |  |  |  |
| Scytalopodinae Müller, 1846 |  |  |  |  |  |  |  |  |  |
| *Merulaxis ater* Lesson, 1830 |  |  |  | 26 |  |  | **26** |  | 5, 47, 363, 391, MCP, MHNCI, WA |
| *Eleoscytalopus indigoticus* (Wied, 1831) |  | 9 | 39 | 86 | 41 |  | **175** |  | 13, 14, 19, 21, 47, 81, 136, 137, 150, 205, 233, 289, 306, 360, 363, 364, 379, 382, 385, 387, 391, 393, 414, MCP, MHNCI, MZUEL, WA |
| *Scytalopus speluncae* (Ménétriès, 1835) |  | 32 |  | 24 | 31 |  | **87** |  | 19, 21, 309, 363, 378, 379, 391, 393, MCP, MHNCI, WA |
| *Scytalopus pachecoi* Maurício, 2005 |  |  |  |  | 1 |  | **1** |  | MHNCI |
| *Scytalopus iraiensis* Bornschein, Reinert & Pichorim, 1998 |  | 53 |  |  | 31 |  | **84** |  | 63, 90, 151, 175, 277, 316, 321, 378, 379, 391, MCP, MHNCI, WA |
| Rhinocryptinae Wetmore, 1926 (1837) |  |  |  |  |  |  |  |  |  |
| *Psilorhamphus guttatus* (Ménétriès, 1835) |  | 5 | 32 | 21 | 11 |  | **69** |  | 13, 14, 19, 21, 22, 47, 134, 234, 360, 363, 379, 382, 385, 391, MCP, MHNCI, WA |
| Furnarioidea Gray, 1840 |  |  |  |  |  |  |  |  |  |
| Formicariidae Gray, 1840 |  |  |  |  |  |  |  |  |  |
| *Formicarius colma* Boddaert, 1783 |  |  | 1 | 61 |  |  | **62** |  | 21, 47, 81, 88, 134, 137, 360, 363, 382, 391, MCP, MHNCI, WA |
| *Chamaeza campanisona* (Lichtenstein, 1823) |  | 36 | 29 | 38 | 56 | 1 | **160** |  | 13, 14, 17, 19, 21, 24, 47, 48, 59, 94, 136, 138, 150, 183, 194, 205, 234, 250, 258, 306, 309, 316, 357, 360, 363, 364, 378, 379, 382, 383, 385, 387, 389, 391, 392, 393, 403, 414, MHNCI, WA |
| *Chamaeza meruloides* Vigors, 1825 |  |  |  | 8 | 5 |  | **13** |  | 382, 391, 402, 403, MHNCI, WA |
| *Chamaeza ruficauda* (Cabanis & Heine, 1859) |  | 8 |  | 7 | 9 |  | **24** |  | 14, 17, 19, 21, 138, 196, 360, 363, 379, 391, 393, MHNCI, WA |
| Scleruridae Swainson, 1827 |  |  |  |  |  |  |  |  |  |
| *Sclerurus scansor* (Ménétriès, 1835) |  | 43 | 7 | 25 | 57 |  | **132** |  | 13, 14, 17, 19, 21, 46, 47, 87, 94, 134, 150, 194, 196, 205, 264, 305, 309, 316, 357, 360, 363, 364, 378, 379, 382, 385, 391, 392, 393, 414, FMNH, MHNCI, WA |
| Dendrocolaptidae Gray, 1840 |  |  |  |  |  |  |  |  |  |
| Sittasominae Ridgway, 1911 |  |  |  |  |  |  |  |  |  |
| *Dendrocincla turdina* (Lichtenstein, 1820) |  | 8 | 23 | 93 | 1 |  | **125** |  | 13, 14, 17, 19, 21, 22, 47, 81, 137, 196, 265, 266, 286, 306, 309, 316, 351, 360, 363, 382, 387, 391, 392, 398, 416, MHNCI, MZUSP, WA |
| *Sittasomus griseicapillus* (Vieillot, 1818) |  | 140 | 123 | 36 | 219 | 2 | **520** |  | 5, 13, 14, 15, 17, 19, 21, 22, 23, 46, 47, 59, 81, 94, 95, 134, 137, 150, 155, 161, 190, 194, 196, 264, 265, 266, 279, 286, 305, 306, 309, 310, 313, 316, 351, 357, 360, 363, 364, 375, 378, 379, 382, 383, 385, 387, 389, 391, 392, 393, 398, 414, 416, FMNH, MCP, MHNCI, WA |
| Dendrocolaptinae Gray, 1840 |  |  |  |  |  |  |  |  |  |
| *Xiphorhynchus fuscus* (Vieillot, 1818) |  | 61 | 45 | 102 | 97 | 1 | **306** |  | 5, 13, 14, 17, 19, 21, 22, 46, 47, 59, 81, 134, 137, 150, 161, 196, 264, 265, 266, 279, 286, 309, 313, 316, 351, 360, 363, 364, 378, 379, 382, 385, 387, 391, 392, 393, 398, 414, 416, MHNCI, MZUSP, WA |
| *Campylorhamphus falcularius* (Vieillot, 1822) |  | 15 | 40 | 7 | 44 |  | **106** |  | 19, 21, 134, 150, 174, 190, 196, 205, 286, 309, 316, 363, 378, 379, 382, 391, 393, 414, MHNCI, WA |
| *Campylorhamphus trochilirostris* (Lichtenstein, 1820) |  |  | 19 |  |  |  | **19** |  | 23, 120, 264, 268, 375, MHNCI, WA |
| *Lepidocolaptes angustirostris* (Vieillot, 1818) |  | 20 | 14 |  | 6 | 13 | **53** |  | 13, 14, 19, 21, 22, 205, 316, 324, 325, 389, 391, 416, MHNCI, WA |
| *Lepidocolaptes falcinellus* (Cabanis & Heine, 1859) |  | 144 | 6 | 11 | 190 | 2 | **353** |  | 14, 17, 19, 21, 22, 46, 134, 137, 150, 161, 194, 250, 279, 284, 286, 294, 305, 306, 309, 316, 357, 363, 364, 378, 379, 382, 391, 393, 414, 416, AMNH, FMNH, MHNCI, WA |
| *Dendrocolaptes platyrostris* Spix, 1825 |  | 99 | 124 | 30 | 173 | 2 | **428** |  | 5, 13, 14, 15, 17, 19, 21, 22, 46, 47, 59, 81, 134, 136, 144, 150, 181, 190, 194, 196, 205, 258, 264, 265, 266, 279, 286, 294, 305, 306, 309, 313, 316, 351, 357, 360, 363, 364, 375, 378, 379, 382, 383, 385, 387, 389, 391, 393, 398, 414, 416, FMNH, MHNCI, WA |
| *Xiphocolaptes albicollis* (Vieillot, 1818) |  | 30 | 46 | 48 | 65 |  | **189** |  | 5, 13, 14, 17, 19, 21, 22, 29, 46, 47, 59, 81, 94, 134, 150, 155, 190, 250, 258, 264, 265, 266, 286, 294, 306, 313, 316, 351, 357, 360, 363, 364, 378, 379, 382, 383, 387, 391, 392, 393, 398, 414, 416, MHNCI, WA |
| Xenopidae Bonaparte, 1854 |  |  |  |  |  |  |  |  |  |
| *Xenops minutus* (Sparrman, 1788) |  | 8 | 8 | 59 | 2 |  | **77** |  | 14, 17, 19, 21, 47, 81, 134, 136, 137, 196, 264, 360, 363, 379, 382, 383, 387, 391, MHNCI, WA |
| *Xenops rutilans* Temminck, 1821 |  | 29 | 60 | 37 | 63 | 1 | **190** |  | 13, 14, 17, 19, 21, 47, 59, 81, 94, 111, 137, 150, 155, 181, 194, 250, 279, 306, 313, 316, 351, 357, 360, 364, 378, 379, 382, 383, 391, 393, 414, MHNCI, WA |
| Furnariidae Gray, 1840 |  |  |  |  |  |  |  |  |  |
| Furnariinae Gray, 1840 |  |  |  |  |  |  |  |  |  |
| *Furnarius rufus* (Gmelin, 1788) |  | 590 | 313 | 69 | 484 | 7 | **1463** |  | 5, 8, 10, 13, 17, 19, 20, 21, 23, 29, 46, 54, 59, 67, 81, 116, 119, 120, 126, 132, 137, 139, 150, 152, 155, 161, 170, 181, 189, 194, 198, 246, 267, 280, 294, 305, 306, 309, 313, 316, 322, 336, 354, 357, 360, 363, 364, 375, 378, 379, 382, 383, 385, 387, 389, 391, 397, 405, 415, FMNH, MHNCI, MZUEL, WA |
| *Phleocryptes melanops* (Vieillot, 1817) |  |  |  | 24 |  |  | **24** |  | 61, 311, MHNCI, WA |
| *Lochmias nematura* (Lichtenstein, 1823) |  | 102 | 60 | 36 | 117 | 3 | **318** |  | 14, 17, 19, 21, 47, 59, 111, 134, 136, 150, 161, 190, 196, 250, 304, 305, 309, 316, 360, 363, 364, 378, 379, 382, 385, 387, 389, 391, 393, FMNH, MHNCI, WA |
| Philydorinae Sclater & Salvin, 1873 |  |  |  |  |  |  |  |  |  |
| *Clibanornis rectirostris* (Wied, 1831) |  |  | 16 |  |  |  | **16** |  | 264, 268, 369, 375, MHNCI, WA |
| *Clibanornis dendrocolaptoides* (Pelzeln, 1859) |  | 95 |  |  | 121 |  | **216** |  | 14, 17, 19, 21, 27, 45, 46, 95, 111, 150, 190, 250, 258, 284, 289, 304, 309, 316, 368, 378, 379, 391, 393, MHNCI, WA |
| *Automolus leucophthalmus* (Wied, 1821) |  | 3 | 102 | 72 | 12 | 1 | **190** |  | 13, 14, 21, 29, 47, 59, 81, 87, 111, 120, 134, 136, 137, 196, 198, 264, 285, 306, 360, 363, 364, 369, 378, 382, 383, 385, 387, 391, 392, 393, MHNCI, MZUSP, WA |
| *Anabazenops fuscus* (Vieillot, 1816) |  |  |  | 21 |  |  | **21** |  | 360, 363, 382, 391, MHNCI, WA |
| *Anabacerthia amaurotis* (Temminck, 1823) |  |  |  | 21 |  |  | **21** |  | 13, 14, 17, 19, 21, 81, 111, 194, 196, 205, 324, 363, 367, 382, 391, 414, MHNCI, WA |
| *Anabacerthia lichtensteini* (Cabanis & Heine, 1859) |  | 1 | 58 | 36 | 10 |  | **105** |  | 13, 14, 15, 21, 47, 59, 81, 111, 137, 264, 313, 363, 382, 383, 385, 387, 391, 392, 393, 414, MHNCI, WA |
| *Philydor atricapillus* (Wied, 1821) |  | 4 | 12 | 136 | 3 |  | **155** |  | 19, 21, 47, 81, 134, 137, 173, 198, 219, 264, 316, 360, 363, 382, 387, 391, 392, 393, 414, MHNCI, WA |
| *Philydor rufum* (Vieillot, 1818) |  | 50 | 61 | 25 | 116 |  | **252** |  | 13, 14, 17, 19, 21, 47, 81, 94, 111, 134, 136, 150, 194, 196, 250, 264, 305, 306, 309, 313, 316, 351, 357, 360, 363, 364, 369, 378, 379, 382, 385, 387, 391, 392, 393, 414, MHNCI, WA |
| *Heliobletus contaminatus* Berlepsch, 1885 |  | 94 | 6 | 11 | 95 |  | **206** |  | 13, 14, 17, 19, 21, 46, 94, 95, 111, 134, 150, 196, 250, 285, 305, 306, 309, 316, 357, 360, 363, 364, 368, 378, 379, 382, 391, 393, 414, FMNH, MHNCI, WA |
| *Syndactyla rufosuperciliata* (Lafresnaye, 1832) |  | 127 | 36 | 14 | 155 | 1 | **333** |  | 13, 14, 17, 19, 21, 46, 111, 134, 136, 150, 161, 190, 194, 196, 250, 267, 279, 305, 306, 309, 316, 357, 360, 363, 364, 378, 379, 382, 385, 389, 391, 393, FMNH, MHNCI, WA |
| *Syndactyla dimidiata* (Pelzeln, 1859) |  |  | 1 |  |  |  | **1** |  | 21, 264 |
| *Cichlocolaptes leucophrus* (Jardine & Selby, 1830) |  | 4 |  | 53 | 7 |  | **64** |  | 19, 21, 46, 47, 81, 134, 360, 363, 378, 382, 391, MHNCI, WA |
| Synallaxiinae De Selys-Longchamps, 1839 (1836) |  |  |  |  |  |  |  |  |  |
| *Leptasthenura striolata* (Pelzeln, 1856) |  | 11 |  |  | 42 |  | **53** |  | 10, 14, 17, 19, 21, 136, 138, 194, 195, 250, 368, 378, 379, 393, MHNCI, WA |
| *Leptasthenura setaria* (Temminck, 1824) |  | 235 | 5 |  | 285 | 1 | **526** |  | 5, 10, 14, 17, 19, 21, 42, 46, 54, 95, 111, 136, 138, 150, 157, 160, 161, 170, 279, 284, 289, 305, 309, 316, 322, 363, 364, 378, 379, 385, 389, 391, 393, 414, FMNH, MHNCI, WA |
| [*Phacellodomus rufifrons*] (Wied, 1821) |  |  | 1 |  |  |  | **1** |  | 310, 313 |
| *Phacellodomus striaticollis* (d'Orbigny & Lafresnaye, 1838) |  | 6 |  |  | 9 |  | **15** |  | 10, 20, 90, 250, 378, 379 |
| *Phacellodomus ruber* (Vieillot, 1817) |  |  | 22 |  |  |  | **22** |  | 120, 324, 374, 375, 391, MHNCI, WA |
| *Phacellodomus ferrugineigula* (Pelzeln, 1858) |  | 12 |  | 2 | 6 |  | **20** |  | 105, 391, MHNCI, WA |
| *Anumbius annumbi* (Vieillot, 1817) |  | 84 |  | 3 | 73 | 5 | **165** |  | 17, 19, 21, 136, 138, 150, 250, 305, 309, 316, 354, 357, 363, 364, 378, 379, 389, 391, 393, MHNCI, WA |
| *Certhiaxis cinnamomeus* (Gmelin, 1788) |  | 94 | 93 | 48 | 122 | 10 | **367** |  | 21, 23, 46, 161, 246, 247, 279, 306, 309, 316, 322, 360, 364, 375, 378, 382, 383, 387, 391, 405, MHNCI, WA |
| *Synallaxis ruficapilla* Vieillot, 1819 |  | 146 | 85 | 91 | 189 | 2 | **513** |  | 5, 13, 14, 17, 19, 21, 46, 47, 59, 81, 111, 112, 119, 134, 137, 150, 161, 181, 190, 194, 196, 250, 279, 285, 305, 306, 309, 313, 316, 322, 338, 357, 360, 363, 364, 378, 379, 382, 383, 385, 387, 389, 391, 392, 393, 414, FMNH, MHNCI, MZUEL, MZUSP, WA |
| *Synallaxis cinerascens* Temminck, 1823 |  | 94 | 47 | 5 | 133 |  | **279** |  | 13, 14, 17, 19, 21, 46, 111, 136, 142, 150, 161, 194, 250, 264, 285, 305, 309, 313, 316, 357, 363, 364, 378, 379, 382, 385, 387, 391, 393, 414, FMNH, MHNCI, WA |
| *Synallaxis frontalis* Pelzeln, 1859 |  | 9 | 126 |  | 21 | 4 | **160** |  | 13, 14, 21, 23, 59, 112, 120, 155, 181, 279, 305, 306, 313, 316, 322, 375, 378, 383, 385, 387, 389, 391, MHNCI, WA |
| *Synallaxis albescens* Temminck, 1823 |  | 1 | 17 |  | 4 | 10 | **32** |  | 325, 375, 389, 391, MHNCI, WA |
| *Synallaxis spixi* Sclater, 1856 |  | 188 | 47 | 57 | 268 | 10 | **570** |  | 5, 10, 13, 14, 17, 19, 20, 21, 46, 47, 54, 81, 137, 150, 161, 181, 194, 198, 205, 219, 230, 246, 305, 306, 309, 316, 322, 357, 360, 363, 364, 378, 379, 382, 383, 385, 387, 389, 391, 393, 405, 414, FMNH, MHNCI, WA |
| *Synallaxis hypospodia* Sclater, 1874 |  |  | 6 |  |  |  | **6** |  | 373, 375, MCN-FZB, MHNCI |
| [*Synallaxis albilora*] Pelzeln, 1856 |  |  | 1 |  |  |  | **1** |  | 313 |
| *Cranioleuca vulpina* (Pelzeln, 1856) |  |  | 33 |  |  |  | **33** |  | 21, 120, 264, 375, 391, MHNCI, WA |
| *Cranioleuca obsoleta* (Reichenbach, 1853) |  | 354 | 39 | 10 | 308 | 1 | **712** |  | 10, 13, 14, 17, 19, 20, 21, 46, 54, 95, 111, 134, 150, 161, 170, 190, 250, 267, 284, 285, 305, 306, 309, 316, 322, 357, 360, 363, 364, 368, 378, 379, 382, 383, 385, 389, 391, 393, MHNCI, WA |
| *Cranioleuca pallida* (Wied, 1831) |  | 103 |  | 7 | 40 |  | **150** |  | 10, 14, 17, 19, 21, 46, 54, 111, 305, 316, 360, 363, 364, 370, 378, 379, 382, 391, 414, MHNCI, WA |
| Tyrannida Wetmore & Miller, 1926 |  |  |  |  |  |  |  |  |  |
| Pipridae Rafinesque, 1815 |  |  |  |  |  |  |  |  |  |
| Piprinae Rafinesque, 1815 |  |  |  |  |  |  |  |  |  |
| *Pipra fasciicauda* Hellmayr, 1906 |  |  | 116 |  |  |  | **116** |  | 21, 22, 264, 294, 306, 310, 313, 314, 383, 387, 391, 392, MHNCI, WA |
| *Manacus manacus* (Linnaeus, 1766) |  |  | 22 | 144 | 2 |  | **168** |  | 13, 14, 21, 29, 47, 81, 85, 88, 98, 134, 136, 137, 230, 264, 313, 360, 363, 369, 382, 387, 391, MHNCI, WA |
| Ilicurinae Prum, 1992 |  |  |  |  |  |  |  |  |  |
| *Ilicura* *militaris* (Shaw & Nodder, 1809) |  |  | 1 | 134 | 1 |  | **136** |  | 81, 264, 363, 382, 391, MHNCI, WA |
| *Chiroxiphia caudata* (Shaw & Nodder, 1793) |  | 144 | 87 | 197 | 207 | 8 | **643** |  | 5, 13, 14, 17, 19, 21, 22, 29, 46, 47, 48, 59, 72, 81, 85, 87, 88, 94, 98, 134, 137, 150, 161, 173, 193, 194, 196, 198, 208, 219, 230, 250, 264, 279, 305, 306, 309, 313, 316, 330, 349, 357, 360, 363, 364, 378, 379, 382, 385, 387, 389, 391, 392, 393, 414, MHNCI, MZUSP, WA |
| *Antilophia galeata* (Lichtenstein, 1823) |  |  | 7 |  |  |  | **7** |  | 264, WA |
| Cotingoidea Bonaparte, 1849 |  |  |  |  |  |  |  |  |  |
| Oxyruncidae Ridgway, 1906 (1831) |  |  |  |  |  |  |  |  |  |
| *Oxyruncus cristatus* Swainson, 1821 |  |  | 8 | 42 |  |  | **50** |  | 13, 14, 21, 47, 81, 134, 306, 360, 363, 367, 371, 375, 382, 387, 391, MHNCI, WA |
| Onychorhynchidae Tello, Moyle, Marchese & Cracraft, 2009 |  |  |  |  |  |  |  |  |  |
| *Onychorhynchus swainsoni* (Pelzeln, 1858) |  | 2 | 1 | 36 | 6 |  | **45** |  | 21, 81, 218, 279, 323, 356, 363, 382, 391, 393, MHNCI, WA |
| *Myiobius barbatus* (Gmelin, 1789) |  |  |  | 57 |  |  | **57** |  | 21, 81, 134, 173, 196, 382, 391, MHNCI, MZUSP, WA |
| *Myiobius atricaudus* Lawrence, 1863 |  | 2 |  | 10 | 3 |  | **15** |  | 19, 21, 47, 363, 364, 382, MHNCI, WA |
| Tityridae Gray, 1840 |  |  |  |  |  |  |  |  |  |
| Schiffornithinae Sibley & Ahlquist, 1985 |  |  |  |  |  |  |  |  |  |
| *Schiffornis virescens* (Lafresnaye, 1838) |  | 47 | 41 | 62 | 86 | 3 | **239** |  | 5, 13, 14, 17, 19, 21, 22, 24, 47, 81, 85, 98, 134, 137, 150, 161, 196, 198, 205, 219, 250, 264, 279, 305, 306, 309, 313, 316, 357, 360, 363, 364, 378, 379, 382, 385, 387, 389, 391, 393, 414, MHNCI, WA |
| [*Laniisoma elegans*] (Thunberg, 1823) |  | 7 | 1 |  |  |  | **8** |  | 8, 10, 306, 324, 378 |
| Tityrinae Gray, 1840 |  |  |  |  |  |  |  |  |  |
| *Tityra inquisitor* (Lichtenstein, 1823) |  | 11 | 117 | 41 | 49 | 1 | **219** |  | 13, 14, 19, 21, 22, 47, 81, 85, 123, 134, 152, 155, 181, 219, 264, 294, 306, 313, 360, 363, 364, 375, 378, 379, 382, 383, 391, 392, 393, 414, FMNH, MHNCI, WA |
| *Tityra cayana* (Linnaeus, 1766) |  | 47 | 66 | 49 | 95 | 1 | **258** |  | 13, 14, 17, 19, 21, 22, 23, 29, 47, 59, 81, 87, 94, 134, 150, 155, 181, 198, 250, 258, 305, 306, 309, 313, 316, 357, 360, 363, 364, 375, 378, 379, 382, 383, 385, 389, 391, 393, 414, FMNH, MHNCI, WA |
| *Tityra semifasciata* (Spix, 1825) |  |  | 6 |  |  |  | **6** | **N** | 123, WA |
| *Pachyramphus viridis* (Vieillot, 1816) |  | 18 | 30 | 14 | 57 |  | **119** |  | 13, 14, 17, 19, 21, 22, 46, 81, 134, 150, 250, 306, 313, 360, 363, 364, 378, 379, 382, 383, 387, 391, 393, 414, FMNH, MHNCI, WA |
| *Pachyramphus castaneus* (Jardine & Selby, 1827) |  | 55 | 49 | 38 | 95 | 2 | **239** |  | 13, 14, 17, 19, 21, 22, 46, 47, 150, 250, 294, 306, 309, 316, 360, 363, 364, 378, 379, 382, 383, 385, 389, 391, 392, 414, MHNCI, WA |
| *Pachyramphus polychopterus* (Vieillot, 1818) |  | 120 | 65 | 26 | 159 | 1 | **371** |  | 5, 10, 13, 14, 17, 19, 21, 22, 46, 47, 54, 59, 81, 134, 150, 155, 170, 181, 198, 205, 264, 267, 305, 306, 309, 313, 316, 322, 363, 364, 375, 378, 379, 382, 383, 385, 389, 391, 393, 414, FMNH, MHNCI, WA |
| *Pachyramphus marginatus* (Lichtenstein, 1823) |  |  |  | 13 |  |  | **13** |  | 384, WA |
| *Pachyramphus validus* (Lichtenstein, 1823) |  | 174 | 186 | 75 | 167 | 6 | **608** |  | 13, 14, 17, 19, 21, 22, 46, 59, 81, 150, 155, 181, 250, 294, 305, 306, 309, 313, 316, 357, 360, 363, 364, 375, 378, 379, 382, 383, 385, 387, 389, 391, 392, 393, 414, FMNH, MHNCI, WA |
| *Xenopsaris albinucha* (Burmeister, 1869) |  |  | 1 |  |  |  | **1** | **N** | WA |
| Cotingidae Bonaparte, 1849 |  |  |  |  |  |  |  |  |  |
| Cotinginae Bonaparte, 1849 |  |  |  |  |  |  |  |  |  |
| *Lipaugus lanioides* (Lesson, 1844) |  |  |  | 25 |  |  | **25** |  | 47, 48, 324, 382, 391, 402, MHNCI, WA |
| *Procnias nudicollis* (Vieillot, 1817) |  | 92 | 8 | 92 | 70 |  | **262** |  | 5, 14, 17, 19, 21, 46, 47, 81, 94, 98, 99, 134, 137, 150, 155, 168, 169, 198, 205, 219, 236, 289, 294, 305, 306, 309, 313, 316, 357, 360, 363, 364, 375, 378, 379, 382, 387, 391, 392, 414, FMNH, MHNCI, WA |
| *Pyroderus scutatus* (Shaw, 1792) |  | 40 | 13 | 34 | 42 |  | **129** |  | 8, 10, 21, 41, 47, 52, 81, 99, 101, 103, 134, 136, 149, 150, 170, 192, 250, 363, 369, 378, 379, 382, 387, 391, 392, 393, 402, 414, MHNCI, WA |
| *Carpornis cucullata* (Swainson, 1821) |  | 10 | 1 | 82 | 53 |  | **146** |  | 5, 81, 88, 98, 99, 134, 149, 150, 196, 250, 363, 369, 378, 382, 391, MHNCI, WA |
| *Carpornis melanocephala* (Wied, 1820) |  |  |  | 31 |  |  | **31** |  | 47, 81, 360, 382, 391, WA |
| *Phibalura flavirostris* Vieillot, 1816 |  | 12 | 7 |  | 22 |  | **41** |  | 21, 22, 27, 99, 134, 157, 250, 306, 309, 378, 379, 392, 393, FMNH, MHNCI, WA |
| Tyrannoidea Vigors, 1825 |  |  |  |  |  |  |  |  |  |
| Pipritidae Ohlson, Irestedt, Ericson & Fjeldså, 2013 |  |  |  |  |  |  |  |  |  |
| *Piprites chloris* (Temminck, 1822) |  |  | 9 | 14 | 2 |  | **25** |  | 13, 14, 21, 134, 264, 306, 360, 363, 379, 382, 387, 391, 392, 393, MHNCI, WA |
| *Piprites pileata* (Temminck, 1822) |  | 4 |  |  | 13 |  | **17** |  | 19, 21, 136, 250, 368, 378, 393, MHNCI, WA |
| Platyrinchidae Bonaparte, 1854 |  |  |  |  |  |  |  |  |  |
| *Platyrinchus mystaceus* Vieillot, 1818 |  | 78 | 40 | 58 | 125 | 2 | **303** |  | 13, 14, 17, 19, 21, 46, 47, 59, 81, 87, 94, 134, 136, 137, 150, 196, 198, 205, 219, 264, 279, 285, 305, 309, 313, 316, 357, 360, 363, 364, 375, 378, 379, 382, 383, 385, 387, 389, 391, 393, 414, FMNH, MHNCI, WA |
| *Platyrinchus leucoryphus* Wied, 1831 |  |  |  | 17 |  |  | **17** |  | 47, 81, 196, 323, 324, 363, 367, MHNCI, WA |
| Tachurididae Ohlson, Irestedt, Ericson & Fjeldså, 2013 |  |  |  |  |  |  |  |  |  |
| *Tachuris rubrigastra* (Vieillot, 1817) |  |  |  | 40 |  |  | **40** |  | 61, 311, MHNCI, WA |
| Rhynchocyclidae Berlepsch, 1907 |  |  |  |  |  |  |  |  |  |
| Pipromorphinae Wolters, 1977 |  |  |  |  |  |  |  |  |  |
| *Mionectes rufiventris* Cabanis, 1846 |  | 43 | 19 | 100 | 83 |  | **245** |  | 5, 13, 14, 17, 19, 21, 43, 46, 47, 81, 85, 88, 134, 137, 150, 194, 196, 198, 222, 230, 250, 279, 305, 306, 309, 313, 316, 357, 360, 363, 364, 378, 379, 382, 385, 387, 391, 392, 393, 414, MHNCI, MZUSP, WA |
| *Leptopogon amaurocephalus* Tschudi, 1846 |  | 52 | 118 | 73 | 145 | 2 | **390** |  | 13, 14, 15, 17, 19, 21, 24, 29, 46, 47, 59, 81, 87, 119, 134, 136, 137, 150, 155, 181, 194, 196, 198, 230, 250, 264, 279, 306, 309, 313, 316, 357, 360, 363, 364, 378, 379, 382, 383, 385, 387, 389, 391, 393, 414, MHNCI, WA |
| *Corythopis delalandi* (Lesson, 1830) |  | 20 | 109 |  | 13 |  | **142** |  | 13, 21, 59, 181, 233, 264, 285, 306, 309, 369, 379, 385, 387, 391, 392, 393, MHNCI, WA |
| *Phylloscartes eximius* (Temminck, 1822) |  | 13 | 10 |  | 33 |  | **56** |  | 13, 14, 17, 19, 21, 22, 136, 264, 289, 306, 309, 357, 378, 379, 391, 392, 393, FMNH, MHNCI, WA |
| *Phylloscartes ventralis* (Temminck, 1824) |  | 103 | 19 | 15 | 145 | 1 | **283** |  | 5, 10, 13, 14, 17, 19, 20, 21, 22, 46, 81, 94, 150, 155, 161, 181, 194, 196, 250, 305, 306, 309, 316, 357, 363, 364, 378, 379, 382, 383, 387, 389, 391, 393, 394, 414, FMNH, MHNCI, WA |
| *Phylloscartes kronei* Willis & Oniki, 1992 |  |  |  | 113 |  |  | **113** |  | 81, 88, 134, 137, 311, 324, 382, 391, MHNCI, WA |
| *Phylloscartes paulista* Ihering & Ihering, 1907 |  |  | 3 | 59 | 3 |  | **65** |  | 21, 47, 157, 363, 379, 382, 392, 393, MHNCI, WA |
| *Phylloscartes oustaleti* (Sclater, 1887) |  | 6 | 2 | 67 |  |  | **75** |  | 14, 17, 19, 21, 22, 173, 360, 363, 367, 382, 391, MHNCI, WA |
| *Phylloscartes difficilis* (Ihering & Ihering, 1907) |  |  |  | 21 | 3 |  | **24** |  | 5, 196, 323, 363, 391, 393, MHNCI, WA |
| *Phylloscartes sylviolus* (Cabanis & Heine, 1859) |  |  | 2 | 16 | 6 |  | **24** |  | 21, 22, 306, 391, WA |
| Rhynchocyclinae Berlepsch, 1907 |  |  |  |  |  |  |  |  |  |
| *Tolmomyias sulphurescens* (Spix, 1825) |  | 47 | 87 | 48 | 78 | 2 | **262** |  | 13, 14, 17, 19, 21, 46, 47, 59, 81, 134, 136, 137, 150, 181, 194, 196, 264, 294, 305, 306, 309, 313, 316, 363, 364, 375, 378, 379, 382, 383, 385, 387, 391, 392, 393, 414, MHNCI, MZUEL, WA |
| Todirostrinae Tello, Moyle, Marchese & Cracraft, 2009 |  |  |  |  |  |  |  |  |  |
| *Todirostrum poliocephalum* (Wied, 1831) |  | 2 | 11 | 131 | 14 |  | **158** |  | 23, 47, 59, 81, 137, 360, 363, 382, 391, MHNCI, WA |
| *Todirostrum cinereum* (Linnaeus, 1766) |  | 39 | 218 | 7 | 54 | 1 | **319** |  | 13, 21, 59, 120, 155, 181, 309, 313, 374, 375, 378, 383, 391, 393, MHNCI, WA |
| *Poecilotriccus plumbeiceps* (Lafresnaye, 1846) |  | 124 | 80 | 17 | 165 | 3 | **389** |  | 13, 14, 17, 19, 21, 23, 46, 81, 87, 150, 155, 161, 181, 190, 194, 196, 198, 206, 285, 305, 306, 309, 313, 316, 338, 357, 360, 363, 364, 378, 379, 382, 383, 385, 387, 389, 391, 393, 405, FMNH, MHNCI, MZUEL, WA |
| *Poecilotriccus latirostris* (Pelzeln, 1868) |  |  | 4 |  |  |  | **4** |  | 313, 324, 374, 375, MHNCI, WA |
| *Myiornis auricularis* (Vieillot, 1818) |  | 13 | 69 | 107 | 34 |  | **223** |  | 13, 14, 17, 19, 21, 47, 59, 81, 119, 134, 136, 150, 155, 181, 264, 279, 285, 306, 309, 313, 338, 360, 363, 364, 375, 378, 379, 382, 383, 385, 387, 391, 392, 393, 414, 420, FMNH, MHNCI, WA |
| *Hemitriccus diops* (Temminck, 1822) |  | 6 | 38 | 20 | 32 |  | **96** |  | 21, 196, 250, 279, 285, 313, 338, 364, 378, 379, 382, 385, 387, 391, 393, MHNCI, WA |
| *Hemitriccus obsoletus* (Miranda-Ribeiro, 1906) |  | 28 | 6 | 13 | 14 |  | **61** |  | 13, 14, 17, 19, 21, 22, 45, 46, 196, 363, 379, 391, MHNCI, WA |
| *Hemitriccus orbitatus* (Wied, 1831) |  |  | 4 | 58 |  |  | **62** |  | 47, 81, 137, 264, 363, 382, 391, 392, 393, MHNCI, WA |
| *Hemitriccus nidipendulus* (Wied, 1831) |  | 9 | 4 | 25 | 3 |  | **41** |  | 13, 14, 17, 19, 21, 196, 279, 324, 382, 392, 402, MHNCI, WA |
| *Hemitriccus margaritaceiventer* (d'Orbigny & Lafresnaye, 1837) |  |  | 46 |  | 5 |  | **51** |  | 21, 264, 313, 385, 391, 393, MHNCI, WA |
| *Hemitriccus kaempferi* (Zimmer, 1953) |  |  |  | 21 |  |  | **21** |  | 81, WA |
| Tyrannidae Vigors, 1825 |  |  |  |  |  |  |  |  |  |
| Hirundineinae Tello, Moyle, Marchese & Cracraft, 2009 |  |  |  |  |  |  |  |  |  |
| *Hirundinea ferruginea* (Gmelin, 1788) |  | 59 | 18 | 40 | 94 | 11 | **222** |  | 17, 19, 21, 94, 120, 136, 137, 219, 246, 305, 306, 309, 316, 360, 363, 364, 369, 378, 379, 382, 389, 391, 393, MHNCI, WA |
| Elaeniinae Cabanis & Heine, 1860 |  |  |  |  |  |  |  |  |  |
| *Euscarthmus meloryphus* Wied, 1831 |  | 11 | 31 |  | 26 | 5 | **73** |  | 21, 155, 306, 309, 313, 316, 375, 378, 379, 383, 385, 389, 391, 392, 393, MHNCI, WA |
| *Tyranniscus burmeisteri* (Cabanis & Heine, 1859) |  | 15 | 7 | 7 | 31 |  | **60** |  | 14, 17, 19, 21, 59, 250, 364, 366, 378, 379, 391, 392, 393, MHNCI, WA |
| *Camptostoma obsoletum* (Temminck, 1824) |  | 218 | 108 | 51 | 284 | 11 | **672** |  | 10, 13, 14, 17, 19, 20, 21, 23, 46, 54, 59, 81, 112, 120, 134, 137, 150, 155, 181, 190, 194, 198, 207, 230, 246, 250, 305, 306, 309, 313, 316, 322, 357, 360, 363, 364, 375, 378, 379, 382, 383, 385, 387, 389, 391, 392, 393, 414, FMNH, MHNCI, WA |
| *Elaenia flavogaster* (Thunberg, 1822) |  | 100 | 109 | 38 | 91 | 8 | **346** |  | 5, 10, 13, 19, 21, 23, 25, 54, 59, 81, 112, 120, 135, 137, 150, 155, 181, 194, 246, 267, 306, 309, 313, 316, 322, 357, 360, 363, 364, 375, 378, 379, 382, 383, 385, 387, 389, 391, MHNCI, WA |
| *Elaenia spectabilis* Pelzeln, 1868 |  |  | 65 |  | 15 | 1 | **81** |  | 120, 375, 391, MHNCI, WA |
| *Elaenia chilensis* Hellmayr, 1927 |  |  |  |  | 2 |  | **2** | **N(S)** | WA |
| *Elaenia parvirostris* Pelzeln, 1868 |  | 120 | 38 | 18 | 160 | 4 | **340** |  | 10, 17, 19, 20, 21, 81, 147, 150, 155, 181, 250, 306, 309, 313, 316, 363, 364, 378, 379, 389, 391, 405, FMNH, MHNCI, WA |
| *Elaenia mesoleuca* (Deppe, 1830) |  | 130 | 10 | 9 | 119 | 3 | **271** |  | 10, 13, 14, 17, 19, 20, 21, 46, 81, 94, 150, 207, 250, 309, 316, 360, 363, 364, 378, 382, 389, 391, 392, 393, 414, FMNH, MHNCI, NMNH, WA |
| *Elaenia cristata* Pelzeln, 1868 |  |  | 1 |  |  | 2 | **3** | **SP** | 389, WA |
| *Elaenia chiriquensis* Lawrence, 1865 |  | 30 | 4 | 1 | 20 | 7 | **62** |  | 17, 19, 21, 264, 309, 316, 325, 389, 391, MHNCI, WA |
| *Elaenia obscura* (d'Orbigny & Lafresnaye, 1837) |  | 63 | 18 | 25 | 51 | 4 | **161** |  | 17, 19, 21, 46, 59, 81, 134, 150, 161, 198, 222, 250, 267, 306, 309, 313, 316, 363, 364, 375, 378, 379, 382, 391, 393, FMNH, MHNCI, WA |
| *Suiriri suiriri* (Vieillot, 1818) |  | 5 |  |  |  | 6 | **11** |  | 21, 309, 316, 325, MHNCI |
| *Myiopagis caniceps* (Swainson, 1835) |  | 15 | 49 | 13 | 59 | 1 | **137** |  | 13, 14, 17, 19, 21, 47, 59, 81, 150, 155, 156, 306, 309, 313, 316, 364, 375, 378, 379, 382, 383, 385, 387, 391, 393, 414, MCP, MHNCI, WA |
| *Myiopagis viridicata* (Vieillot, 1817) |  | 3 | 66 |  | 22 |  | **91** |  | 13, 14, 21, 59, 87, 134, 155, 264, 306, 309, 313, 375, 379, 383, 385, 387, 391, 393, MHNCI, WA |
| *Capsiempis flaveola* (Lichtenstein, 1823) |  |  | 107 |  | 45 |  | **152** |  | 13, 14, 23, 59, 134, 155, 181, 195, 206, 264, 285, 306, 313, 375, 379, 383, 385, 387, 391, 392, 393, MHNCI, MZUEL, WA |
| *Phaeomyias murina* (Spix, 1825) |  | 8 | 8 |  |  | 2 | **18** |  | 306, 309, 325, 391, MHNCI, WA |
| *Phyllomyias virescens* (Temminck, 1824) |  | 20 | 5 | 9 | 54 |  | **88** |  | 21, 95, 134, 137, 150, 250, 306, 316, 363, 364, 378, 379, 382, 391, 393, MHNCI, WA |
| *Phyllomyias fasciatus* (Thunberg, 1822) |  | 37 | 7 | 19 | 49 |  | **112** |  | 14, 17, 19, 21, 46, 81, 134, 150, 155, 194, 279, 360, 363, 378, 379, 382, 387, 391, 393, 414, FMNH, MHNCI, WA |
| *Phyllomyias griseocapilla* Sclater, 1862 |  | 7 |  | 37 | 3 |  | **47** |  | 21, 81, 363, 382, 391, MHNCI, WA |
| *Culicivora caudacuta* (Vieillot, 1818) |  | 39 |  |  | 8 | 1 | **48** |  | 21, 90, 250, 287, 309, 316, 378, 391, MHNCI, WA |
| *Polystictus pectoralis* (Vieillot, 1817) |  |  |  | 2 |  | 8 | **10** | **SP** | 61, 246, WA |
| *Pseudocolopteryx sclateri* (Oustalet, 1892) |  |  | 4 |  |  |  | **4** |  | MCN-FZB |
| *Pseudocolopteryx flaviventris* (d'Orbigny & Lafresnaye, 1837) |  |  |  | 5 |  |  | **5** |  | 58, WA |
| *Serpophaga nigricans* (Vieillot, 1817) |  | 85 | 8 | 24 | 123 | 1 | **241** |  | 19, 21, 46, 87, 112, 134, 276, 305, 306, 309, 313, 316, 322, 360, 378, 379, 382, 387, 391, 393, 405, MHNCI, WA |
| *Serpophaga subcristata* (Vieillot, 1817) |  | 268 | 115 | 12 | 296 | 7 | **698** |  | 10, 13, 17, 19, 20, 21, 46, 54, 59, 81, 112, 120, 150, 155, 161, 170, 181, 198, 207, 219, 230, 246, 304, 305, 306, 309, 313, 316, 322, 354, 360, 364, 375, 378, 379, 382, 389, 391, 393, 405, FMNH, MHNCI, WA |
| Tyranninae Vigors, 1825 |  |  |  |  |  |  |  |  |  |
| *Attila phoenicurus* Pelzeln, 1868 |  | 57 | 4 | 23 | 48 |  | **132** |  | 19, 21, 46, 47, 81, 134, 150, 155, 194, 198, 205, 250, 306, 360, 363, 364, 366, 378, 379, 382, 391, MHNCI, WA |
| *Attila rufus* (Vieillot, 1819) |  |  |  | 108 |  |  | **108** |  | 5, 47, 81, 134, 137, 196, 207, 219, 230, 250, 360, 363, 382, 391, MHNCI, WA |
| *Legatus leucophaius* (Vieillot, 1818) |  | 45 | 39 | 72 | 59 |  | **215** |  | 19, 21, 22, 47, 59, 81, 94, 134, 137, 150, 181, 198, 207, 250, 264, 305, 306, 309, 313, 316, 357, 360, 363, 364, 375, 378, 379, 382, 383, 385, 387, 391, 392, 393, FMNH, MHNCI, WA |
| *Ramphotrigon megacephalum* (Swainson, 1835) |  |  | 3 | 16 | 3 |  | **22** |  | 21, 150, 379, 382, 391, 393, MHNCI, WA |
| *Myiarchus swainsoni* Cabanis & Heine, 1859 |  | 74 | 66 | 21 | 126 | 7 | **294** |  | 10, 13, 14, 17, 19, 21, 23, 59, 81, 134, 150, 155, 156, 181, 198, 250, 264, 305, 306, 309, 313, 316, 322, 357, 360, 363, 364, 375, 378, 379, 382, 383, 385, 389, 391, 392, 393, 414, FMNH, MHNCI, WA |
| *Myiarchus ferox* (Gmelin, 1789) |  | 22 | 64 | 41 | 35 |  | **162** |  | 19, 21, 23, 59, 81, 150, 207, 264, 294, 306, 309, 316, 322, 378, 379, 382, 391, 392, FMNH, MHNCI, WA |
| *Myiarchus tyrannulus* (Statius Muller, 1776) |  | 8 | 37 |  | 4 | 14 | **63** |  | 306, 309, 325, 375, 389, 391, 393, MHNCI, WA |
| *Sirystes sibilator* (Vieillot, 1818) |  | 18 | 92 | 77 | 33 | 2 | **222** |  | 5, 13, 14, 21, 22, 47, 59, 81, 87, 134, 137, 181, 198, 250, 264, 306, 313, 357, 360, 363, 364, 375, 378, 379, 382, 383, 385, 387, 389, 391, 392, 393, MHNCI, WA |
| *Casiornis rufus* (Vieillot, 1816) |  |  | 8 |  |  |  | **8** |  | 324, 375, WA |
| *Pitangus sulphuratus* (Linnaeus, 1766) |  | 515 | 372 | 109 | 502 | 9 | **1507** |  | 5, 8, 10, 13, 14, 17, 19, 20, 21, 22, 23, 25, 29, 46, 54, 59, 81, 94, 112, 113, 116, 119, 120, 126, 129, 132, 134, 135, 137, 138, 139, 143, 147, 150, 155, 156, 161, 170, 181, 189, 194, 198, 207, 219, 226, 230, 246, 247, 253, 267, 276, 294, 304, 305, 306, 309, 313, 316, 322, 349, 354, 357, 360, 363, 364, 369, 375, 378, 379, 382, 383, 385, 387, 389, 391, 392, 393, 405, 414, 415, 417, FMNH, MHNCI, WA |
| *Machetornis rixosa* (Vieillot, 1819) |  | 292 | 212 | 57 | 298 | 1 | **860** |  | 5, 10, 17, 19, 20, 21, 22, 23, 37, 59, 81, 112, 114, 116, 119, 120, 126, 139, 143, 150, 155, 161, 181, 246, 305, 306, 309, 313, 316, 322, 357, 360, 363, 364, 375, 378, 379, 382, 383, 385, 391, 405, 415, 417, MHNCI, WA |
| *Myiodynastes maculatus* (Statius Muller, 1776) |  | 190 | 232 | 74 | 238 | 4 | **738** |  | 10, 13, 14, 17, 19, 20, 21, 22, 23, 25, 46, 47, 59, 81, 112, 119, 120, 126, 132, 134, 137, 139, 150, 155, 156, 161, 181, 194, 198, 219, 250, 264, 294, 305, 306, 309, 313, 316, 322, 357, 360, 363, 364, 369, 375, 378, 379, 382, 383, 385, 387, 389, 391, 392, 393, 414, FMNH, MHNCI, WA |
| *Megarynchus pitangua* (Linnaeus, 1766) |  | 78 | 200 | 49 | 127 | 2 | **456** |  | 5, 10, 13, 14, 15, 17, 19, 21, 22, 23, 24, 46, 47, 59, 81, 83, 87, 94, 119, 126, 134, 137, 139, 150, 155, 156, 160, 161, 181, 194, 198, 230, 264, 294, 305, 306, 309, 313, 316, 349, 357, 360, 363, 364, 375, 378, 379, 382, 383, 385, 387, 389, 391, 414, FMNH, MHNCI, WA |
| *Myiozetetes cayanensis* (Linnaeus, 1766) |  |  | 1 |  |  |  | **1** | **N, C** | WA |
| *Myiozetetes similis* (Spix, 1825) |  | 34 | 153 | 109 | 66 |  | **362** |  | 13, 14, 21, 22, 23, 47, 59, 81, 92, 119, 120, 134, 135, 137, 138, 155, 173, 181, 246, 267, 294, 306, 309, 313, 316, 357, 360, 363, 375, 378, 379, 382, 383, 385, 387, 391, 392, 393, MHNCI, WA |
| *Tyrannus albogularis* Burmeister, 1856 |  |  | 12 |  | 1 |  | **13** | **N, C** | WA |
| *Tyrannus melancholicus* Vieillot, 1819 |  | 404 | 201 | 104 | 469 | 8 | **1186** |  | 5, 10, 13, 14, 17, 19, 20, 21, 22, 23, 46, 47, 54, 59, 81, 112, 113, 116, 119, 120, 129, 132, 134, 135, 137, 139, 147, 150, 155, 156, 161, 170, 173, 177, 181, 194, 198, 207, 219, 226, 230, 246, 250, 264, 276, 279, 294, 305, 306, 309, 313, 316, 322, 349, 360, 363, 364, 375, 378, 379, 382, 383, 385, 387, 389, 391, 392, 393, 405, 414, FMNH, MHNCI, WA |
| *Tyrannus savana* Vieillot, 1808 |  | 344 | 277 | 43 | 438 | 8 | **1110** |  | 5, 10, 17, 19, 20, 21, 22, 23, 46, 59, 81, 116, 119, 120, 126, 132, 134, 150, 155, 156, 170, 176, 181, 194, 198, 207, 219, 246, 250, 267, 294, 305, 306, 309, 313, 316, 322, 360, 363, 364, 369, 375, 378, 379, 382, 383, 385, 389, 391, 393, 405, FMNH, MHNCI, MZUEL, WA |
| *Tyrannus tyrannus* (Linnaeus, 1766) |  |  |  | 2 | 2 |  | **4** |  | 58, 378, WA |
| *Griseotyrannus aurantioatrocristatus* (d'Orbigny & Lafresnaye, 1837) |  |  | 10 |  |  |  | **10** | **SP** | 181, 310, 313, WA |
| *Empidonomus varius* (Vieillot, 1818) |  | 130 | 190 | 60 | 158 |  | **538** |  | 5, 13, 14, 17, 19, 21, 22, 23, 47, 59, 81, 87, 120, 134, 150, 155, 156, 160, 161, 181, 198, 250, 294, 305, 306, 309, 313, 316, 360, 363, 364, 375, 378, 379, 382, 383, 385, 391, 393, FMNH, MHNCI, WA |
| *Conopias trivirgatus* (Wied, 1831) |  |  | 7 | 56 |  |  | **63** |  | 21, 47, 81, 120, 123, 134, 137, 306, 363, 382, 383, 391, 392, MHNCI, MZUSP, WA |
| Fluvicolinae Swainson, 1832 |  |  |  |  |  |  |  |  |  |
| *Colonia colonus* (Vieillot, 1818) |  | 42 | 108 | 105 | 99 | 1 | **355** |  | 13, 14, 17, 19, 21, 47, 59, 81, 87, 94, 119, 134, 136, 137, 152, 155, 181, 194, 264, 294, 305, 306, 309, 313, 316, 322, 357, 360, 363, 364, 375, 378, 379, 382, 383, 385, 387, 391, 393, 414, FMNH, MHNCI, WA |
| *Myiophobus fasciatus* (Statius Muller, 1776) |  | 182 | 63 | 44 | 233 | 5 | **527** |  | 7, 10, 13, 14, 17, 19, 20, 21, 23, 54, 59, 81, 134, 150, 155, 170, 181, 194, 219, 267, 279, 305, 306, 309, 313, 316, 322, 357, 360, 364, 375, 378, 379, 382, 383, 385, 389, 391, 393, 405, FMNH, MHNCI, WA |
| *Pyrocephalus rubinus* (Boddaert, 1783) |  | 134 | 253 | 56 | 156 | 1 | **600** |  | 10, 19, 20, 21, 22, 81, 116, 134, 137, 150, 155, 161, 181, 198, 219, 246, 250, 294, 305, 306, 309, 313, 316, 322, 357, 360, 363, 364, 369, 375, 378, 379, 383, 391, 393, MHNCI, WA |
| *Fluvicola albiventer* (Spix, 1825) |  |  | 16 |  |  |  | **16** |  | 21, 120, 374, 375, MHNCI, WA |
| *Fluvicola nengeta* (Linnaeus, 1766) |  | 86 | 84 | 97 | 25 | 3 | **295** | **C** | 308, 378, 388, 391, MHNCI, WA |
| *Arundinicola leucocephala* (Linnaeus, 1764) |  | 34 | 60 | 15 | 30 | 2 | **141** |  | 21, 22, 59, 120, 132, 305, 316, 322, 375, 378, 383, 391, MHNCI, WA |
| *Gubernetes yetapa* (Vieillot, 1818) |  | 18 | 68 |  | 9 |  | **95** |  | 21, 23, 90, 294, 369, 375, 383, 391, MHNCI, WA |
| *Alectrurus tricolor* (Vieillot, 1816) |  | 11 | 1 |  |  | 1 | **13** |  | 19, 21, 90, 250, 287, 305, 316, 375 |
| *Cnemotriccus fuscatus* (Wied, 1831) |  | 17 | 77 | 64 | 28 | 1 | **187** |  | 5, 13, 17, 19, 21, 46, 47, 59, 81, 112, 134, 137, 150, 155, 198, 206, 250, 264, 279, 285, 306, 309, 313, 316, 360, 363, 364, 375, 378, 379, 382, 383, 385, 391, 392, 414, MHNCI, WA |
| *Lathrotriccus euleri* (Cabanis, 1868) |  | 131 | 73 | 60 | 118 | 8 | **390** |  | 10, 13, 14, 17, 19, 21, 46, 47, 59, 81, 119, 137, 150, 155, 161, 181, 198, 279, 285, 294, 306, 309, 313, 316, 322, 338, 357, 360, 363, 364, 375, 378, 379, 382, 383, 385, 387, 389, 391, 392, 393, 414, FMNH, MHNCI, MZUSP, WA |
| *Contopus cooperi* (Nuttall, 1831) |  |  |  |  | 1 |  | **1** | **N** | WA |
| *Contopus cinereus* (Spix, 1825) |  | 22 | 20 | 19 | 60 |  | **121** |  | 10, 17, 19, 20, 21, 47, 54, 59, 81, 94, 134, 137, 147, 150, 190, 250, 305, 306, 309, 313, 316, 357, 363, 364, 375, 378, 379, 382, 385, 391, 392, 393, 414, MHNCI, WA |
| *Lessonia rufa* (Gmelin, 1789) |  |  |  | 2 |  |  | **2** |  | 62, 324 |
| *Knipolegus cyanirostris* (Vieillot, 1818) |  | 40 | 9 | 8 | 97 |  | **154** |  | 5, 10, 17, 19, 21, 22, 46, 150, 190, 198, 219, 250, 306, 309, 316, 357, 360, 363, 364, 378, 379, 382, 391, 392, 393, MHNCI, WA |
| *Knipolegus lophotes* Boie, 1828 |  | 80 | 1 | 2 | 29 | 11 | **123** |  | 5, 17, 19, 21, 250, 305, 309, 316, 364, 378, 389, 391, FMNH, MHNCI, WA |
| *Knipolegus nigerrimus* (Vieillot, 1818) |  | 56 |  | 33 | 11 |  | **100** |  | 17, 19, 21, 23, 260, 305, 309, 316, 363, 391, FMNH, MHNCI, WA |
| *Hymenops perspicillatus* (Gmelin, 1789) |  | 1 | 6 | 6 | 3 | 9 | **25** |  | 26, 378, MHNCI, WA |
| *Satrapa icterophrys* (Vieillot, 1818) |  | 177 | 46 | 39 | 195 |  | **457** |  | 10, 17, 19, 20, 21, 22, 54, 81, 132, 137, 150, 161, 181, 194, 198, 219, 246, 306, 309, 313, 316, 322, 360, 363, 364, 375, 378, 379, 382, 391, 405, MHNCI, WA |
| *Xolmis cinereus* (Vieillot, 1816) |  | 80 | 8 | 1 | 76 | 6 | **171** |  | 17, 19, 21, 22, 134, 205, 305, 309, 313, 316, 357, 364, 375, 378, 379, 383, 389, 391, 393, 394, MHNCI, WA |
| *Xolmis velatus* (Lichtenstein, 1823) |  | 47 | 99 |  | 26 | 8 | **180** |  | 21, 94, 309, 313, 325, 379, 389, 391, MHNCI, WA |
| *Xolmis irupero* (Vieillot, 1823) |  |  |  | 1 | 5 |  | **6** | **SP** | 292, WA |
| *Xolmis dominicanus* (Vieillot, 1823) |  | 29 | 2 | 2 | 29 | 1 | **63** |  | 5, 19, 21, 134, 250, 277, 287, 305, 309, 316, 357, 363, 375, 378, 379, 391, MHNCI, WA |
| *Muscipipra vetula* (Lichtenstein, 1823) |  | 24 | 4 | 18 | 78 |  | **124** |  | 5, 14, 17, 19, 21, 22, 81, 94, 137, 150, 194, 195, 306, 309, 360, 363, 364, 378, 379, 391, 392, FMNH, MHNCI, WA |
| Passeri Linnaeus, 1758 |  |  |  |  |  |  |  |  |  |
| Corvida Wagler 1830 |  |  |  |  |  |  |  |  |  |
| Vireonidae Swainson, 1837 |  |  |  |  |  |  |  |  |  |
| *Cyclarhis gujanensis* (Gmelin, 1789) |  | 295 | 188 | 41 | 342 | 8 | **874** |  | 5, 10, 13, 14, 17, 19, 21, 23, 29, 45, 46, 47, 54, 59, 81, 94, 119, 134, 137, 139, 150, 155, 161, 170, 176, 181, 190, 194, 196, 198, 219, 230, 250, 276, 279, 305, 306, 309, 313, 316, 322, 330, 357, 360, 363, 364, 375, 378, 379, 382, 383, 385, 387, 389, 391, 393, 414, FMNH, MHNCI, WA |
| *Vireo chivi* (Vieillot, 1817) |  | 154 | 69 | 120 | 224 | 5 | **572** |  | 5, 10, 13, 14, 17, 19, 20, 21, 29, 46, 47, 54, 59, 81, 85, 134, 137, 138, 140, 142, 150, 155, 156, 170, 177, 181, 194, 198, 207, 219, 230, 250, 279, 294, 305, 306, 309, 313, 316, 322, 357, 360, 363, 364, 375, 378, 379, 382, 387, 389, 391, 392, 393, 414, FMNH, MHNCI, WA |
| *Hylophilus poicilotis* Temminck, 1822 |  | 71 | 15 | 46 | 81 | 1 | **214** |  | 5, 13, 14, 17, 19, 21, 46, 47, 81, 134, 137, 150, 196, 219, 250, 294, 309, 313, 316, 357, 360, 363, 364, 378, 379, 382, 387, 391, 393, 414, FMNH, MHNCI, WA |
| *Hylophilus amaurocephalus* (Nordmann, 1835) |  | 2 | 2 |  | 1 | 2 | **7** |  | 87, 118, 306, 389, WA |
| Corvidae Leach, 1820 |  |  |  |  |  |  |  |  |  |
| *Cyanocorax cyanomelas* (Vieillot, 1818) |  |  | 13 |  |  |  | **13** | **SP** | 294, 310, 324, 374, 375, WA |
| *Cyanocorax caeruleus* (Vieillot, 1818) |  | 80 | 2 | 216 | 103 |  | **401** |  | 5, 9, 11, 14, 17, 19, 21, 46, 47, 60, 81, 94, 134, 136, 137, 138, 150, 173, 195, 207, 230, 250, 258, 274, 289, 305, 309, 316, 350, 354, 357, 363, 378, 379, 382, 391, 393, 414, FMNH, MHNCI, WA |
| *Cyanocorax cristatellus* (Temminck, 1823) |  | 5 | 10 |  |  | 10 | **25** |  | 21, 325, 389, 391, MHNCI, WA |
| *Cyanocorax chrysops* (Vieillot, 1818) |  | 175 | 418 | 4 | 254 | 4 | **855** |  | 13, 14, 17, 19, 21, 23, 34, 35, 68, 94, 123, 134, 136, 138, 142, 150, 155, 181, 194, 250, 258, 264, 267, 294, 305, 306, 309, 313, 316, 357, 364, 369, 375, 378, 379, 383, 385, 387, 389, 391, 392, 401, 414, FMNH, MHNCI, WA |
| Passerida Linnaeus, 1758 |  |  |  |  |  |  |  |  |  |
| Hirundinidae Rafinesque, 1815 |  |  |  |  |  |  |  |  |  |
| *Pygochelidon cyanoleuca* (Vieillot, 1817) |  | 336 | 133 | 92 | 352 | 9 | **922** |  | 5, 10, 19, 20, 21, 23, 46, 54, 59, 81, 114, 134, 137, 142, 150, 155, 161, 170, 181, 194, 198, 207, 219, 226, 230, 246, 294, 305, 306, 309, 313, 316, 322, 357, 360, 363, 364, 375, 378, 379, 382, 383, 385, 387, 389, 391, 393, 397, 405, FMNH, MCP, MHNCI, WA |
| [*Pygochelidon melanoleuca*] (Wied, 1820) |  |  | 2 |  |  |  | **2** |  | 381, 387 |
| *Alopochelidon fucata* (Temminck, 1822) |  | 34 | 6 |  | 23 |  | **63** |  | 19, 21, 23, 87, 134, 305, 309, 316, 375, 378, 379, MHNCI, WA |
| [*Atticora tibialis*] (Cassin, 1853) |  |  | 1 |  |  |  | **1** |  | 324, 374 |
| *Stelgidopteryx ruficollis* (Vieillot, 1817) |  | 110 | 107 | 80 | 203 | 6 | **506** |  | 19, 21, 23, 59, 81, 120, 150, 155, 173, 181, 194, 198, 207, 219, 226, 305, 306, 309, 313, 316, 322, 357, 360, 363, 364, 375, 378, 379, 382, 383, 385, 387, 389, 391, FMNH, MHNCI, WA |
| *Progne tapera* (Vieillot, 1817) |  | 90 | 83 | 33 | 111 | 3 | **320** |  | 19, 21, 81, 120, 150, 181, 219, 294, 306, 309, 313, 316, 322, 363, 375, 378, 379, 382, 383, 389, 391, 405, MHNCI, WA |
| *Progne* *subis* (Linnaeus, 1758) |  |  | 13 |  |  |  | **13** |  | 324, 355, WA |
| *Progne chalybea* (Gmelin, 1789) |  | 202 | 134 | 69 | 294 | 2 | **701** |  | 5, 19, 23, 54, 59, 81, 94, 116, 136, 137, 150, 155, 161, 170, 173, 181, 194, 198, 207, 219, 226, 246, 306, 309, 313, 316, 357, 360, 363, 364, 369, 375, 378, 379, 382, 383, 385, 387, 389, 391, 392, 393, MHNCI, WA |
| [*Progne elegans*] Baird, 1865 |  |  | 1 |  |  |  | **1** |  | 326 |
| *Tachycineta albiventer* (Boddaert, 1783) |  | 34 | 179 | 5 | 63 | 1 | **282** |  | 21, 23, 59, 120, 134, 138, 155, 264, 294, 306, 309, 369, 375, 378, 379, 383, 385, 387, 391, 392, 393, MHNCI, WA |
| *Tachycineta leucorrhoa* (Vieillot, 1817) |  | 174 | 72 | 17 | 182 | 4 | **449** |  | 19, 21, 59, 81, 150, 181, 246, 294, 305, 306, 309, 313, 316, 322, 357, 360, 363, 364, 375, 378, 379, 382, 387, 389, 391, 405, FMNH, MHNCI, WA |
| *Tachycineta leucopyga* (Meyen, 1834) |  |  | 1 | 2 |  |  | **3** | **SP** | 58, 324, WA |
| *Riparia riparia* (Linnaeus, 1758) |  |  | 9 | 3 |  |  | **12** |  | 222, 294, 360, 363, 375, MHNCI, WA |
| *Hirundo rustica* Linnaeus, 1758 |  | 4 | 55 | 37 | 18 |  | **114** |  | 5, 21, 23, 81, 181, 306, 309, 322, 375, 378, 382, 383, 391, MHNCI, WA |
| *Petrochelidon pyrrhonota* (Vieillot, 1817) |  | 15 | 14 |  | 10 | 1 | **40** |  | 19, 21, 23, 267, 309, 379, 389, 391, WA |
| Troglodytidae Swainson, 1831 |  |  |  |  |  |  |  |  |  |
| *Troglodytes musculus* Naumann, 1823 |  | 445 | 264 | 91 | 453 | 13 | **1266** |  | 5, 10, 13, 14, 17, 19, 20, 21, 23, 37, 46, 47, 54, 59, 81, 112, 119, 120, 126, 132, 134, 137, 139, 150, 152, 155, 161, 164, 170, 173, 177, 181, 189, 190, 194, 198, 207, 219, 226, 230, 246, 267, 294, 305, 306, 309, 313, 316, 322, 357, 360, 363, 364, 375, 378, 379, 382, 383, 385, 387, 389, 391, 392, 393, 405, 414, 415, FMNH, MHNCI, WA |
| *Cistothorus platensis* (Latham, 1790) |  | 31 |  |  | 4 | 3 | **38** |  | 21, 90, 250, 287, 309, 316, 324, 325, 378, 379, MHNCI, WA |
| *Campylorhynchus turdinus* (Wied, 1831) |  |  | 11 |  |  |  | **11** | **C, SP** | 43, WA |
| *Cantorchilus leucotis* (Lafresnaye, 1845) |  |  | 30 |  |  |  | **30** |  | 23, 264, 294, 375, MCN-FZB, MHNCI, WA |
| *Cantorchilus longirostris* (Vieillot, 1819) |  | 1 |  | 119 |  |  | **120** |  | 47, 62, 81, 134, 137, 230, 360, 363, 382, 391, MHNCI, WA |
| Donacobiidae Aleixo & Pacheco, 2006 |  |  |  |  |  |  |  |  |  |
| *Donacobius atricapilla* (Linnaeus, 1766) |  |  | 111 |  | 10 | 1 | **122** |  | 21, 23, 59, 120, 181, 264, 306, 313, 369, 375, 383, 391, MCN-FZB, MCP, MHNCI, WA |
| Polioptilidae Baird, 1858 |  |  |  |  |  |  |  |  |  |
| *Ramphocaenus melanurus* Vieillot, 1819 |  |  |  | 55 |  |  | **55** |  | 47, 81, 137, 360, 363, 382, 391, 402, MHNCI, WA |
| *Polioptila lactea* Sharpe, 1885 |  | 5 | 5 |  | 35 |  | **45** |  | 13, 14, 21, 52, 101, 195, 379, 393, MHNCI, WA |
| Turdidae Rafinesque, 1815 |  |  |  |  |  |  |  |  |  |
| *Turdus flavipes* Vieillot, 1818 |  | 109 | 3 | 158 | 61 |  | **331** |  | 5, 10, 14, 17, 19, 20, 21, 46, 47, 54, 81, 85, 98, 134, 137, 150, 170, 196, 198, 208, 219, 230, 250, 309, 316, 360, 363, 364, 378, 379, 382, 391, 392, 393, FMNH, MHNCI, WA |
| *Turdus leucomelas* Vieillot, 1818 |  | 76 | 335 | 28 | 139 | 14 | **592** |  | 13, 14, 21, 23, 24, 25, 29, 59, 112, 119, 120, 126, 135, 139, 142, 155, 156, 181, 194, 201, 222, 264, 267, 294, 306, 309, 310, 313, 316, 357, 363, 364, 375, 378, 379, 382, 383, 385, 387, 389, 391, 392, 413, 415, MHNCI, WA |
| *Turdus fumigatus* Lichtenstein, 1823 |  |  | 1 |  |  |  | **1** | **N(S)** | WA |
| *Turdus rufiventris* Vieillot, 1818 |  | 788 | 183 | 126 | 615 | 16 | **1728** |  | 5, 8, 10, 13, 14, 17, 19, 20, 21, 23, 28, 29, 30, 31, 45, 46, 47, 54, 59, 81, 85, 94, 98, 112, 116, 119, 120, 134, 137, 140, 142, 147, 150, 155, 156, 161, 170, 173, 181, 189, 190, 194, 196, 198, 205, 207, 219, 230, 246, 250, 264, 267, 279, 283, 294, 305, 306, 309, 313, 316, 322, 349, 357, 360, 363, 364, 375, 378, 379, 382, 383, 385, 387, 389, 391, 393, 397, 411, 412, 413, 414, FMNH, MHNCI, WA |
| *Turdus amaurochalinus* Cabanis, 1850 |  | 273 | 197 | 115 | 399 | 8 | **992** |  | 5, 10, 13, 14, 17, 19, 20, 21, 23, 28, 46, 47, 54, 59, 81, 94, 112, 119, 126, 132, 134, 137, 139, 150, 155, 156, 170, 173, 181, 190, 194, 198, 207, 219, 230, 246, 250, 267, 294, 305, 306, 309, 313, 316, 322, 357, 360, 363, 364, 375, 378, 379, 382, 383, 387, 389, 391, 392, 393, 405, 414, 415, FMNH, MHNCI, WA |
| *Turdus subalaris* (Seebohm, 1887) |  | 75 | 47 | 3 | 94 | 2 | **221** |  | 10, 13, 17, 19, 20, 21, 46, 54, 59, 81, 150, 155, 161, 170, 194, 306, 309, 313, 363, 378, 379, 383, 389, 391, 393, FMNH, MHNCI, WA |
| *Turdus albicollis* Vieillot, 1818 |  | 94 | 58 | 105 | 145 | 1 | **403** |  | 5, 13, 14, 17, 19, 21, 28, 29, 30, 46, 47, 48, 54, 59, 72, 81, 85, 88, 94, 98, 112, 134, 137, 142, 150, 155, 156, 173, 181, 194, 196, 198, 208, 219, 230, 279, 305, 306, 309, 313, 316, 322, 360, 363, 364, 369, 378, 379, 382, 385, 387, 391, 393, 414, MHNCI, MZUSP, WA |
| Mimidae Bonaparte, 1853 |  |  |  |  |  |  |  |  |  |
| [*Mimus gilvus*] (Vieillot, 1807) |  |  |  | 2 |  |  | **2** |  | 62 |
| *Mimus saturninus* (Lichtenstein, 1823) |  | 160 | 319 | 22 | 261 | 13 | **775** |  | 5, 19, 21, 59, 112, 116, 120, 132, 150, 181, 267, 305, 306, 309, 313, 316, 322, 357, 364, 375, 378, 379, 383, 385, 389, 391, 415, 417, FMNH, MHNCI, WA |
| *Mimus triurus (*Vieillot, 1818) |  |  | 9 | 28 | 7 |  | **44** |  | 23, 61, 246, 391, WA |
| Motacillidae Horsfield, 1821 |  |  |  |  |  |  |  |  |  |
| *Anthus lutescens* Pucheran, 1855 |  | 48 | 54 | 16 | 67 | 9 | **194** |  | 19, 21, 59, 134, 181, 222, 246, 247, 305, 309, 316, 378, 379, 383, 385, 389, 391, 405, MCN-FZB, MHNCI, WA |
| [*Anthus correndera*] Vieillot, 1818 |  |  |  | 3 |  |  | **3** |  | 21, 62, 324 |
| *Anthus nattereri* Sclater, 1878 |  | 18 |  |  | 5 |  | **23** |  | 19, 21, 90, 309, 378, 379, 393, WA |
| *Anthus hellmayri* Hartert, 1909 |  | 63 |  | 1 | 34 |  | **98** |  | 19, 21, 250, 309, 316, 378, 379, 391, FMNH, MHNCI, WA |
| Passerellidae Cabanis & Heine, 1850 |  |  |  |  |  |  |  |  |  |
| *Zonotrichia capensis* (Statius Muller, 1776) |  | 448 | 172 | 106 | 586 | 12 | **1324** |  | 5, 8, 10, 14, 17, 19, 20, 21, 23, 46, 54, 59, 81, 94, 113, 119, 120, 129, 132, 134, 137, 150, 155, 161, 170, 177, 181, 189, 190, 194, 198, 219, 230, 246, 247, 267, 276, 279, 294, 305, 306, 309, 313, 316, 322, 330, 357, 360, 363, 364, 375, 378, 379, 382, 383, 385, 387, 389, 391, 392, 405, 414, 415, FMNH, MHNCI, WA |
| *Ammodramus humeralis* (Bosc, 1792) |  | 98 | 145 |  | 136 | 20 | **399** |  | 19, 21, 23, 59, 132, 150, 155, 181, 194, 250, 294, 305, 309, 313, 316, 322, 364, 375, 378, 379, 383, 385, 389, 391, 393, MHNCI, WA |
| *Arremon semitorquatus* Swainson, 1838 |  | 6 | 30 | 34 | 10 |  | **80** |  | 21, 219, 245, 269, 270, 364, 382, 391, 392, MHNCI, WA |
| *Arremon flavirostris* Swainson, 1838 |  |  | 97 |  |  |  | **97** |  | 13, 19, 21, 59, 119, 264, 269, 294, 309, 313, 357, 375, 379, 383, 385, 387, 391, 393, MHNCI, WA |
| Parulidae Wetmore, Friedmann, Lincoln, Miller, Peters, van Rossem, Van Tyne & Zimmer 1947 |  |  |  |  |  |  |  |  |  |
| *Setophaga pitiayumi* (Vieillot, 1817) |  | 242 | 139 | 89 | 370 | 13 | **853** |  | 5, 10, 13, 14, 17, 19, 20, 21, 22, 23, 46, 47, 54, 59, 81, 95, 112, 119, 123, 134, 137, 150, 155, 161, 170, 181, 194, 198, 201, 207, 219, 230, 264, 294, 305, 306, 309, 313, 316, 322, 357, 360, 363, 364, 375, 378, 379, 382, 383, 385, 387, 389, 391, 392, 393, 414, FMNH, MHNCI, WA |
| [*Setophaga striata*] (Forster, 1772) |  | 1 |  |  |  |  | **1** |  | 87 |
| *Geothlypis aequinoctialis* (Gmelin, 1789) |  | 165 | 119 | 91 | 224 | 7 | **606** |  | 5, 10, 17, 19, 21, 22, 23, 29, 46, 59, 81, 112, 134, 137, 150, 161, 181, 198, 207, 219, 230, 246, 250, 267, 306, 309, 316, 322, 357, 360, 363, 364, 375, 378, 379, 382, 385, 387, 389, 391, 392, 393, 405, FMNH, MHNCI, WA |
| *Basileuterus culicivorus* (Deppe, 1830) |  | 202 | 206 | 115 | 281 | 8 | **812** |  | 5, 10, 13, 14, 15, 17, 19, 20, 21, 22, 23, 24, 29, 46, 47, 48, 54, 59, 81, 112, 119, 134, 136, 137, 142, 150, 155, 161, 173, 178, 181, 190, 194, 196, 198, 208, 219, 230, 264, 279, 294, 305, 306, 309, 313, 316, 322, 330, 357, 360, 363, 364, 375, 378, 379, 382, 383, 385, 387, 389, 391, 392, 393, 414, FMNH, MHNCI, WA |
| *Myiothlypis flaveola* Baird, 1865 |  |  | 69 |  |  |  | **69** |  | 59, 324, 391, WA |
| *Myiothlypis leucoblephara* (Vieillot, 1817) |  | 223 | 75 | 14 | 293 | 4 | **609** |  | 5, 10, 13, 14, 17, 19, 20, 21, 22, 24, 29, 45, 46, 47, 119, 134, 142, 150, 161, 170, 181, 190, 194, 196, 250, 264, 279, 305, 306, 309, 316, 322, 330, 357, 360, 363, 364, 378, 379, 382, 385, 387, 389, 391, 393, 414, FMNH, MHNCI, WA |
| [*Myiothlypis leucophrys*] (Pelzeln, 1868) |  |  | 1 |  |  |  | **1** |  | 56 |
| *Myiothlypis rivularis* (Wied, 1821) |  | 7 | 11 | 178 | 6 | 1 | **203** |  | 21, 47, 81, 134, 136, 137, 173, 230, 357, 360, 363, 378, 379, 382, 387, 389, 391, 392, MHNCI, WA |
| Icteridae Vigors, 1825 |  |  |  |  |  |  |  |  |  |
| *Psarocolius decumanus* (Pallas, 1769) |  |  | 10 |  | 8 |  | **18** |  | x1, 21, 264, 369, 393, FMNH, MHNCI, WA |
| *Procacicus solitarius* (Vieillot, 1816) |  |  | 14 |  |  |  | **14** |  | 264, 313, 374, 375, 391, MCN-FZB, MHNCI, WA |
| *Cacicus chrysopterus* (Vigors, 1825) |  | 138 | 24 | 16 | 186 | 1 | **365** |  | 5, 14, 17, 19, 21, 46, 87, 94, 118, 134, 194, 205, 207, 250, 258, 279, 294, 305, 309, 316, 357, 360, 363, 364, 378, 379, 382, 385, 391, 393, 414, FMNH, MHNCI, NMNH, WA |
| *Cacicus haemorrhous* (Linnaeus, 1766) |  | 168 | 296 | 117 | 236 | 2 | **819** |  | 5, 13, 14, 15, 17, 19, 21, 43, 47, 59, 81, 94, 123, 134, 137, 138, 150, 155, 156, 181, 207, 264, 294, 306, 309, 313, 316, 322, 354, 357, 360, 363, 364, 369, 375, 378, 379, 382, 383, 385, 387, 389, 391, 392, 393, 414, FMNH, MHNCI, WA |
| *Icterus pyrrhopterus* (Vieillot, 1819) |  | 9 | 118 | 1 | 54 |  | **182** |  | 21, 23, 87, 118, 120, 194, 264, 294, 306, 313, 364, 375, 379, 383, 385, 391, MHNCI, WA |
| *Icterus croconotus* (Wagler, 1829) |  |  | 10 |  |  |  | **10** | **C** | 313, WA |
| *Gnorimopsar chopi* (Vieillot, 1819) |  | 72 | 49 | 4 | 95 | 5 | **225** |  | 5, 17, 19, 21, 23, 94, 134, 137, 150, 181, 194, 276, 305, 309, 313, 316, 357, 364, 369, 375, 378, 379, 383, 385, 387, 389, 391, 392, 393, FMNH, MHNCI, WA |
| *Amblyramphus holosericeus* (Scopoli, 1786) |  |  | 18 |  | 1 |  | **19** |  | 59, 310, MCN-FZB, WA |
| *Agelasticus cyanopus* (Vieillot, 1819) |  |  | 7 | 23 |  |  | **30** |  | 21, 246, 374, 375, 391, MCP, MHNCI, WA |
| *Agelasticus thilius* (Molina, 1782) |  |  |  | 33 |  |  | **33** |  | 21, 62, 311, MHNCI, WA |
| *Chrysomus ruficapillus* (Vieillot, 1819) |  | 124 | 95 | 3 | 101 | 6 | **329** |  | 21, 81, 316, 322, 374, 375, 378, 391, 405, MCP, MHNCI, WA |
| *Pseudoleistes guirahuro* (Vieillot, 1819) |  | 176 | 76 | 3 | 165 | 2 | **422** |  | 19, 21, 81, 150, 250, 276, 305, 306, 309, 316, 322, 357, 364, 378, 379, 389, 391, FMNH, MHNCI, WA |
| *Agelaioides badius* (Vieillot, 1819) |  | 23 | 13 | 2 | 91 |  | **129** |  | 21, 219, 316, 378, 391, MHNCI, WA |
| *Molothrus rufoaxillaris* Cassin, 1866 |  | 17 | 13 |  | 19 | 1 | **50** |  | 21, 87, 150, 309, 324, 374, 375, 378, 379, 391, WA |
| *Molothrus oryzivorus* (Gmelin, 1788) |  | 15 | 46 | 5 | 19 | 1 | **86** |  | 21, 43, 118, 155, 306, 309, 313, 374, 378, 379, 391, 393, WA |
| *Molothrus bonariensis* (Gmelin, 1789) |  | 476 | 206 | 85 | 472 | 6 | **1245** |  | 5, 10, 17, 19, 20, 21, 23, 46, 54, 59, 81, 116, 119, 120, 127, 128, 134, 137, 142, 150, 155, 161, 170, 176, 177, 181, 189, 198, 207, 219, 246, 250, 276, 305, 306, 309, 313, 316, 322, 357, 360, 363, 364, 369, 375, 378, 379, 382, 383, 385, 391, 392, 393, 405, 415, FMNH, MHNCI, MZUEL, WA |
| *Sturnella superciliaris* (Bonaparte, 1850) |  | 74 | 218 | 49 | 115 | 11 | **467** |  | 19, 21, 23, 59, 150, 181, 246, 309, 313, 316, 360, 363, 364, 375, 378, 379, 382, 383, 385, 389, 391, 393, 405, MHNCI, WA |
| [*Dolichonyx oryzivorus*] (Linnaeus, 1758) |  | 3 | 1 |  |  |  | **4** |  | 19, 21, 324 |
| Mitrospingidae Barker, Burns, Klicka, Lanyon & Lovette, 2013 |  |  |  |  |  |  |  |  |  |
| *Orthogonys chloricterus* (Vieillot, 1819) |  |  | 1 | 96 |  |  | **97** |  | 47, 81, 137, 360, 363, 369, 382, 391, MHNCI, WA |
| Thraupidae Cabanis, 1847 |  |  |  |  |  |  |  |  |  |
| *Coereba flaveola* (Linnaeus, 1758) |  | 60 | 154 | 161 | 113 | 3 | **491** |  | 13, 21, 47, 54, 81, 87, 112, 134, 137, 150, 155, 161, 181, 194, 198, 202, 203, 219, 230, 246, 294, 306, 309, 313, 363, 364, 378, 379, 382, 385, 391, 393, MHNCI, WA |
| *Saltatricula atricollis* (Vieillot, 1817) |  | 1 |  |  |  |  | **1** |  | 87 |
| *Saltator coerulescens* Vieillot, 1817 |  |  | 3 |  |  |  | **3** | **N, C** | 201, WA |
| *Saltator similis* d'Orbigny & Lafresnaye, 1837 |  | 191 | 144 | 65 | 322 | 12 | **734** |  | 5, 10, 13, 14, 17, 19, 20, 21, 23, 25, 29, 46, 47, 54, 59, 81, 83, 85, 98, 112, 119, 137, 150, 155, 156, 161, 170, 181, 190, 194, 196, 198, 219, 230, 250, 267, 276, 279, 305, 306, 309, 313, 316, 322, 357, 360, 363, 364, 375, 378, 379, 382, 383, 385, 387, 389, 391, 414, FMNH, MHNCI, WA |
| *Saltator maxillosus* Cabanis, 1851 |  | 49 |  | 9 | 77 |  | **135** |  | 19, 21, 46, 94, 95, 150, 190, 232, 243, 309, 316, 363, 364, 378, 379, 382, 391, 393, MHNCI, WA |
| *Saltator fuliginosus* (Daudin, 1800) |  | 8 | 30 | 40 | 36 |  | **114** |  | 13, 14, 17, 19, 21, 29, 47, 59, 81, 134, 138, 360, 363, 382, 391, 393, MHNCI, WA |
| *Orchesticus abeillei* (Lesson, 1839) |  | 3 |  | 39 | 8 |  | **50** |  | 5, 13, 21, 250, 360, 363, 378, 379, 382, 391, MHNCI, WA |
| *Nemosia pileata* (Boddaert, 1783) |  |  | 87 | 1 |  |  | **88** |  | 21, 23, 120, 155, 181, 294, 306, 313, 375, 383, 385, 391, MHNCI, WA |
| *Thlypopsis sordida* (d'Orbigny & Lafresnaye, 1837) |  | 6 | 51 | 9 | 10 |  | **76** |  | 2, 31, 32, 33, 120, 264, 374, 375, 378, 382, 391, MCP, MHNCI, WA |
| *Pyrrhocoma ruficeps* (Strickland, 1844) |  | 29 | 56 | 1 | 126 |  | **212** |  | 17, 19, 21, 94, 150, 190, 194, 279, 294, 306, 309, 313, 357, 363, 364, 375, 378, 379, 382, 385, 387, 391, 392, 393, 414, MHNCI, WA |
| *Cypsnagra hirundinacea* (Lesson, 1831) |  |  |  |  |  | 7 | **7** |  | 325, 389, MHNCI |
| [*Tachyphonus rufus*] (Boddaert, 1783) |  |  | 1 |  |  |  | **1** |  | 313 |
| *Tachyphonus coronatus* (Vieillot, 1822) |  | 208 | 153 | 214 | 279 | 5 | **859** |  | 5, 10, 13, 14, 17, 19, 21, 22, 29, 47, 54, 59, 81, 83, 85, 86, 87, 94, 119, 134, 137, 150, 155, 156, 173, 181, 190, 194, 196, 198, 208, 219, 230, 264, 279, 294, 305, 306, 309, 313, 316, 322, 349, 357, 360, 363, 364, 369, 375, 378, 379, 382, 383, 385, 387, 389, 391, 392, 393, 405, 414, FMNH, MHNCI, WA |
| *Ramphocelus bresilius* (Linnaeus, 1766) |  | 2 |  | 368 |  |  | **370** |  | 81, 98, 134, 137, 173, 198, 207, 219, 230, 246, 250, 349, 382, 391, MHNCI, WA |
| *Ramphocelus carbo* (Pallas, 1764) |  |  | 48 |  |  |  | **48** |  | 21, 23, 59, 120, 136, 264, 294, 369, 375, 391, 392, MHNCI, WA |
| *Lanio cristatus* (Linnaeus, 1766) |  |  |  | 139 |  |  | **139** |  | 47, 81, 85, 86, 98, 134, 137, 230, 360, 363, 382, 391, MHNCI, WA |
| *Lanio cucullatus* (Statius Muller, 1776) |  | 71 | 190 | 9 | 141 | 9 | **420** |  | 5, 21, 23, 132, 134, 137, 150, 155, 181, 264, 294, 305, 306, 309, 313, 316, 369, 375, 378, 379, 383, 385, 387, 389, 391, 393, FMNH, MHNCI, WA |
| *Lanio penicillatus* (Spix, 1825) |  |  | 3 |  |  |  | **3** |  | 313, MHNCI |
| *Lanio melanops* (Vieillot, 1818) |  | 113 | 164 | 136 | 159 | 2 | **574** |  | 5, 13, 14, 15, 17, 19, 21, 22, 29, 47, 59, 81, 85, 86, 119, 134, 137, 150, 155, 181, 194, 196, 201, 230, 264, 279, 305, 306, 309, 313, 316, 357, 360, 363, 364, 378, 379, 382, 383, 385, 387, 389, 391, 392, 393, 414, FMNH, MHNCI, MZUSP, WA |
| *Tangara seledon* (Statius Muller, 1776) |  | 1 | 44 | 343 | 3 |  | **391** |  | 5, 21, 47, 72, 81, 85, 98, 134, 137, 230, 264, 306, 349, 360, 363, 369, 379, 382, 387, 391, 392, MHNCI, WA |
| *Tangara cyanocephala* (Statius Muller, 1776) |  |  |  | 263 |  |  | **263** |  | 5, 47, 72, 81, 85, 98, 134, 137, 207, 208, 349, 360, 363, 382, 391, MHNCI, WA |
| *Tangara desmaresti* (Vieillot, 1819) |  | 38 |  | 33 | 14 |  | **85** |  | 19, 21, 22, 149, 150, 196, 250, 289, 363, 378, 382, 391, MHNCI, WA |
| *Tangara sayaca* (Linnaeus, 1766) |  | 557 | 322 | 111 | 547 | 8 | **1545** |  | 5, 10, 13, 14, 17, 19, 20, 21, 22, 23, 25, 46, 47, 54, 59, 81, 86, 98, 112, 116, 119, 120, 126, 134, 135, 137, 139, 147, 150, 155, 156, 161, 170, 181, 189, 194, 198, 202, 203, 207, 219, 230, 246, 250, 253, 264, 283, 294, 305, 306, 309, 313, 316, 322, 357, 360, 363, 364, 375, 378, 379, 382, 383, 385, 387, 389, 391, 414, 415, FMNH, MHNCI, MZUEL, WA |
| *Tangara cyanoptera* (Vieillot, 1817) |  | 33 |  | 161 | 20 |  | **214** |  | 5, 47, 81, 86, 94, 134, 137, 196, 219, 230, 360, 363, 364, 378, 382, 391, MHNCI, WA |
| *Tangara palmarum* (Wied, 1823) |  | 10 | 30 | 124 | 3 | 1 | **168** |  | 21, 81, 120, 134, 137, 264, 313, 360, 363, 375, 378, 382, 389, 391, MHNCI, WA |
| *Tangara ornata* (Sparrman, 1789) |  | 19 |  | 88 | 3 |  | **110** |  | 5, 81, 86, 360, 363, 378, 382, 391, MHNCI, WA |
| *Tangara peruviana* (Desmarest, 1806) |  |  |  | 162 |  |  | **162** |  | 81, 85, 137, 198, 207, 219, 220, 230, 349, 382, 391, MHNCI, WA |
| *Tangara preciosa* (Cabanis, 1850) |  | 279 | 14 | 28 | 181 |  | **502** |  | 10, 14, 17, 19, 21, 22, 46, 47, 54, 81, 94, 98, 118, 134, 141, 150, 160, 161, 175, 194, 250, 289, 305, 306, 309, 316, 357, 360, 363, 364, 378, 379, 382, 391, 393, 414, MHNCI, WA |
| *Tangara cayana* (Linnaeus, 1766) |  | 15 | 21 |  | 2 | 9 | **47** |  | 13, 21, 87, 120, 264, 294, 309, 369, 375, 385, 389, 391, FMNH, MHNCI, WA |
| *Stephanophorus diadematus* (Temminck, 1823) |  | 114 |  | 25 | 181 | 1 | **321** |  | 5, 14, 17, 19, 21, 22, 46, 94, 150, 194, 232, 250, 267, 309, 316, 322, 357, 360, 363, 364, 378, 379, 382, 391, FMNH, MHNCI, WA |
| *Neothraupis fasciata* (Lichtenstein, 1823) |  |  |  |  |  | 11 | **11** |  | 21, 325, 389, MHNCI |
| *Cissopis leverianus* (Gmelin, 1788) |  | 10 | 181 | 14 | 69 | 1 | **275** |  | 13, 14, 19, 21, 59, 73, 134, 181, 264, 294, 306, 313, 357, 363, 369, 379, 383, 385, 387, 391, 392, FMNH, MHNCI, WA |
| *Schistochlamys melanopis* (Latham, 1790) |  |  | 4 |  |  |  | **4** | **N, C** | WA |
| *Schistochlamys ruficapillus* (Vieillot, 1817) |  | 52 |  | 3 | 20 | 12 | **87** |  | 5, 21, 250, 309, 316, 325, 389, 391, MHNCI, WA |
| *Paroaria coronata* (Miller, 1776) |  | 108 | 19 |  | 32 |  | **159** |  | 21, 324, 378, 391, WA |
| *Paroaria dominicana* (Linnaeus, 1758) |  |  | 1 |  |  |  | **1** | **N, C** | WA |
| *Paroaria capitata* (d'Orbigny & Lafresnaye, 1837) |  | 3 | 38 |  | 6 |  | **47** |  | 120, 324, 374, 375, 378, 391, WA |
| *Pipraeidea melanonota* (Vieillot, 1819) |  | 186 | 73 | 40 | 267 | 2 | **568** |  | 5, 10, 14, 17, 19, 20, 21, 22, 46, 47, 54, 59, 81, 94, 134, 137, 147, 150, 152, 155, 161, 170, 181, 219, 250, 294, 305, 306, 309, 313, 316, 357, 360, 363, 364, 375, 378, 379, 382, 385, 387, 389, 391, 392, 393, 395, MHNCI, WA |
| *Pipraeidea bonariensis* (Gmelin, 1789) |  | 368 | 112 | 4 | 312 |  | **796** |  | 5, 10, 14, 17, 19, 20, 21, 29, 54, 87, 135, 155, 161, 170, 194, 232, 309, 322, 357, 378, 379, 391, MHNCI, WA |
| *Tersina viridis* (Illiger, 1811) |  | 238 | 179 | 69 | 317 | 7 | **810** |  | 5, 14, 17, 19, 21, 22, 47, 81, 86, 134, 135, 147, 150, 155, 161, 173, 181, 198, 207, 219, 230, 264, 305, 306, 309, 313, 316, 349, 357, 360, 363, 378, 379, 382, 385, 387, 389, 391, 393, 414, FMNH, MHNCI, WA |
| *Dacnis nigripes* Pelzeln, 1856 |  |  |  | 22 |  |  | **22** |  | 324, 382, MHNCI, WA |
| *Dacnis cayana* (Linnaeus, 1766) |  | 109 | 187 | 193 | 67 | 3 | **559** |  | 5, 21, 47, 59, 81, 87, 98, 123, 134, 135, 136, 137, 150, 155, 156, 181, 219, 230, 267, 294, 306, 309, 313, 316, 349, 360, 363, 364, 375, 378, 379, 382, 383, 385, 387, 389, 391, 392, MHNCI, WA |
| *Chlorophanes spiza* (Linnaeus, 1758) |  |  |  | 103 |  |  | **103** |  | 47, 81, 98, 363, 382, 391, MHNCI, MZUSP, WA |
| *Hemithraupis guira* (Linnaeus, 1766) |  | 28 | 163 | 7 | 131 | 2 | **331** |  | 13, 14, 21, 59, 81, 85, 86, 87, 118, 123, 137, 155, 156, 181, 195, 294, 305, 306, 309, 313, 316, 357, 364, 375, 379, 382, 383, 385, 387, 389, 391, 392, 393, 414, MCP, MHNCI, WA |
| *Hemithraupis ruficapilla* (Vieillot, 1818) |  | 7 | 1 | 97 | 2 |  | **107** |  | 14, 17, 19, 21, 47, 81, 134, 137, 230, 309, 360, 363, 382, 391, FMNH, MHNCI, WA |
| *Conirostrum speciosum* (Temminck, 1824) |  | 26 | 161 | 5 | 78 | 1 | **271** |  | 13, 14, 21, 23, 59, 81, 87, 119, 120, 134, 135, 155, 156, 181, 194, 306, 309, 313, 316, 364, 375, 378, 379, 382, 383, 385, 387, 391, 392, 414, MHNCI, WA |
| *Conirostrum bicolor* (Vieillot, 1809) |  |  |  | 42 |  |  | **42** |  | 81, 198, 207, 311, 324, WA |
| *Haplospiza unicolor* Cabanis, 1851 |  | 66 | 22 | 39 | 86 |  | **213** |  | 5, 14, 17, 19, 21, 43, 47, 81, 134, 150, 173, 190, 195, 196, 230, 279, 305, 306, 309, 313, 316, 363, 364, 378, 379, 382, 387, 391, 392, 393, MHNCI, WA |
| *Donacospiza albifrons* (Vieillot, 1817) |  | 60 |  | 1 | 72 |  | **133** |  | 19, 21, 250, 309, 316, 363, 378, 379, 391, 405, MHNCI, WA |
| *Poospiza thoracica* (Nordmann, 1835) |  | 3 |  | 6 | 30 |  | **39** |  | 5, 190, 363, 378, 379, 391, 393, MHNCI, WA |
| *Poospiza nigrorufa* (d'Orbigny & Lafresnaye, 1837) |  | 96 |  |  | 100 |  | **196** |  | 19, 21, 150, 177, 309, 316, 322, 378, 379, 391, 405, MHNCI, WA |
| *Poospiza cabanisi* Bonaparte, 1850 |  | 257 |  | 7 | 316 | 3 | **583** |  | 5, 10, 14, 17, 19, 20, 21, 46, 54, 94, 95, 118, 134, 150, 161, 190, 194, 250, 279, 284, 305, 309, 316, 322, 357, 360, 363, 364, 378, 379, 382, 389, 391, 393, 414, FMNH, MHNCI, WA |
| *Sicalis citrina* Pelzeln, 1870 |  | 44 |  |  | 6 | 9 | **59** |  | 19, 21, 250, 309, 316, 325, 378, 379, 389, 391, MHNCI, WA |
| *Sicalis flaveola* (Linnaeus, 1766) |  | 621 | 278 | 177 | 609 | 3 | **1688** |  | 5, 10, 17, 19, 20, 21, 23, 37, 42, 46, 81, 94, 120, 134, 137, 150, 152, 161, 170, 173, 194, 198, 219, 250, 305, 306, 309, 313, 316, 322, 349, 357, 360, 363, 364, 375, 378, 379, 382, 383, 385, 387, 389, 391, 392, 405, 414, FMNH, MHNCI, WA |
| *Sicalis luteola* (Sparrman, 1789) |  | 84 | 81 | 1 | 71 | 19 | **256** |  | 19, 21, 305, 309, 316, 363, 378, 379, 391, 393, MHNCI, WA |
| *Emberizoides herbicola* (Vieillot, 1817) |  | 72 | 28 | 3 | 49 | 7 | **159** |  | 5, 19, 21, 150, 305, 309, 316, 363, 378, 379, 389, 391, 393, MHNCI, WA |
| *Emberizoides ypiranganus* Ihering & Ihering, 1907 |  | 39 | 1 |  | 18 | 1 | **59** |  | 19, 21, 287, 309, 316, 391, MHNCI, WA |
| *Embernagra platensis* (Gmelin, 1789) |  | 180 | 24 | 2 | 177 | 3 | **386** |  | 5, 19, 21, 81, 150, 250, 267, 276, 305, 309, 316, 322, 363, 364, 375, 378, 379, 385, 387, 389, 391, 393, 405, MHNCI, WA |
| *Volatinia jacarina* (Linnaeus, 1766) |  | 221 | 186 | 40 | 294 | 6 | **747** |  | 5, 10, 17, 19, 20, 21, 23, 29, 59, 81, 112, 119, 120, 132, 134, 150, 155, 161, 181, 194, 198, 276, 305, 306, 309, 313, 316, 322, 357, 363, 364, 375, 378, 379, 382, 383, 385, 387, 389, 391, 405, FMNH, MHNCI, WA |
| *Sporophila frontalis* (Verreaux, 1869) |  | 10 |  | 47 | 6 |  | **63** |  | 5, 47, 81, 88, 218, 363, 382, 391, MHNCI, WA |
| *Sporophila falcirostris* (Temminck, 1820) |  | 2 | 1 | 23 | 4 |  | **30** |  | 21, 43, 49, 81, 88, 324, 379, 382, 391, MHNCI, WA |
| *Sporophila plumbea* (Wied, 1830) |  | 18 |  |  |  | 1 | **19** |  | 21, 87, 250, 309, 316, 378, AMNH, MCP, MHNCI, WA |
| *Sporophila beltoni* Repenning & Fontana, 2013 |  | 34 |  |  |  |  | **34** | **N** | 90, WA |
| *Sporophila collaris* (Boddaert, 1783) |  | 1 | 41 | 5 | 4 |  | **51** |  | 21, 23, 374, 375, 378, 391, MCP, MHNCI, WA |
| *Sporophila lineola* (Linnaeus, 1758) |  | 12 | 67 | 40 | 35 | 1 | **155** |  | 21, 23, 81, 87, 119, 120, 374, 375, 382, 391, MHNCI, WA |
| *Sporophila nigricollis* (Vieillot, 1823) |  |  | 4 | 1 | 1 |  | **6** |  | 324, 391, WA |
| *Sporophila caerulescens* (Vieillot, 1823) |  | 316 | 273 | 93 | 389 | 8 | **1079** |  | 5, 10, 17, 19, 20, 21, 23, 43, 46, 59, 81, 112, 119, 120, 132, 134, 137, 150, 155, 161, 170, 181, 194, 198, 219, 246, 250, 294, 305, 306, 309, 313, 316, 322, 357, 360, 363, 364, 369, 375, 378, 379, 382, 383, 385, 387, 389, 391, 392, 393, FMNH, MCP, MHNCI, WA |
| *Sporophila leucoptera* (Vieillot, 1817) |  | 2 | 22 | 1 | 2 |  | **27** |  | 310, 391, WA |
| *Sporophila pileata* (Sclater, 1864) |  | 46 | 1 |  | 2 |  | **49** |  | 19, 21, 87, 90, 305, 309, 316, 378, MCP, WA |
| *Sporophila hypoxantha* Cabanis, 1851 |  | 77 | 4 |  | 35 |  | **116** |  | 19, 21, 44, 87, 90, 250, 305, 309, 316, 375, 378, 379, 391, MCP, MHNCI, WA |
| *Sporophila ruficollis* Cabanis, 1851 |  |  | 1 |  |  |  | **1** | **N** | WA |
| *Sporophila palustris* (Barrows, 1883) |  | 1 |  |  |  |  | **1** |  | 90 |
| *Sporophila cinnamomea* (Lafresnaye, 1839) |  | 5 | 2 |  |  |  | **7** |  | 87, 90, MHNCI, WA |
| *Sporophila melanogaster* (Pelzeln, 1870) |  | 12 |  | 2 | 2 |  | **16** |  | 19, 21, 87, 90, 309, WA |
| *Sporophila angolensis* (Linnaeus, 1766) |  | 13 | 12 | 39 | 21 | 1 | **86** |  | 19, 21, 23, 81, 87, 134, 316, 363, 378, 379, 382, 391, 393, 405, MHNCI, WA |
| *Tiaris fuliginosus* (Wied, 1830) |  | 6 | 18 | 29 | 2 |  | **55** |  | 14, 17, 19, 21, 47, 59, 62, 81, 88, 279, 379, 382, 383, 391, MHNCI, WA |
| *Coryphaspiza melanotis* (Temminck, 1822) |  | 4 |  |  |  |  | **4** |  | MHNCI, WA |
| Cardinalidae Ridgway, 1901 |  |  |  |  |  |  |  |  |  |
| *Piranga flava* (Vieillot, 1822) |  | 19 |  |  | 29 | 1 | **49** |  | 21, 22, 94, 101, 157, 250, 309, 378, 379, 391, FMNH, MHNCI, WA |
| *Habia rubica* (Vieillot, 1817) |  | 14 | 94 | 112 | 17 |  | **237** |  | 13, 14, 17, 19, 21, 22, 29, 47, 59, 81, 85, 137, 155, 173, 196, 264, 279, 306, 309, 313, 360, 363, 364, 379, 382, 383, 385, 387, 391, 392, 393, MHNCI, MZUSP, WA |
| *Amaurospiza moesta* (Hartlaub, 1853) |  | 26 | 12 | 7 | 63 | 1 | **109** |  | 19, 21, 27, 41, 134, 157, 190, 196, 306, 363, 379, 382, 385, 387, 389, 391, 393, FMNH, MHNCI, WA |
| *Cyanoloxia glaucocaerulea* (d'Orbigny & Lafresnaye, 1837) |  | 65 | 22 | 5 | 52 |  | **144** |  | 5, 19, 21, 45, 46, 87, 157, 193, 194, 305, 306, 309, 316, 360, 363, 375, 378, 379, 391, 392, 393, MHNCI, WA |
| *Cyanoloxia brissonii* (Lichtenstein, 1823) |  | 21 | 21 | 32 | 94 |  | **168** |  | 19, 21, 29, 87, 118, 157, 181, 194, 195, 309, 313, 316, 322, 357, 363, 364, 378, 379, 382, 383, 385, 391, 393, 414, FMNH, MHNCI, WA |
| Fringillidae Leach, 1820 |  |  |  |  |  |  |  |  |  |
| *Sporagra magellanica* (Vieillot, 1805) |  | 346 | 52 | 28 | 408 | 5 | **839** |  | 5, 10, 17, 19, 20, 21, 46, 81, 150, 161, 181, 194, 250, 276, 305, 306, 309, 313, 316, 322, 357, 360, 363, 364, 378, 379, 383, 385, 389, 391, FMNH, MHNCI, WA |
| *Euphonia chlorotica* (Linnaeus, 1766) |  | 30 | 249 | 5 | 56 | 2 | **342** |  | 10, 13, 14, 21, 22, 25, 47, 59, 87, 118, 119, 120, 129, 137, 155, 170, 181, 294, 305, 306, 309, 313, 316, 360, 375, 378, 379, 383, 385, 387, 389, 391, MHNCI, WA |
| *Euphonia violacea* (Linnaeus, 1758) |  | 36 | 80 | 259 | 59 |  | **434** |  | 5, 13, 14, 21, 29, 47, 81, 85, 98, 120, 123, 134, 137, 150, 155, 198, 219, 230, 264, 294, 306, 309, 313, 316, 360, 363, 364, 378, 379, 382, 383, 385, 387, 391, 414, MHNCI, WA |
| *Euphonia chalybea* (Mikan, 1825) |  | 120 | 16 | 38 | 93 |  | **267** |  | 17, 19, 21, 22, 81, 134, 150, 289, 316, 360, 363, 364, 378, 379, 382, 391, 393, MHNCI, WA |
| *Euphonia cyanocephala* (Vieillot, 1818) |  | 29 | 56 | 19 | 18 | 1 | **123** |  | 13, 14, 17, 19, 21, 22, 81, 155, 181, 230, 309, 324, 378, 379, 382, 391, 414, MHNCI, WA |
| *Euphonia pectoralis* (Latham, 1801) |  | 30 | 44 | 186 | 5 |  | **265** |  | 5, 17, 19, 21, 22, 47, 48, 81, 85, 98, 118, 134, 136, 137, 155, 173, 196, 230, 264, 306, 360, 363, 364, 378, 379, 382, 385, 387, 391, 392, 393, MHNCI, WA |
| *Chlorophonia cyanea* (Thunberg, 1822) |  | 45 | 31 | 2 | 49 |  | **127** |  | 21, 264, 309, 316, 357, 378, 379, 382, 391, 393, MHNCI, WA |
| Estrildidae Bonaparte, 1850 |  |  |  |  |  |  |  |  |  |
| *Estrilda astrild* (Linnaeus, 1758) |  | 205 | 38 | 48 | 210 |  | **501** | **I** | 10, 20, 81, 137, 150, 155, 161, 181, 246, 309, 316, 322, 378, 391, 405, MHNCI, WA |
| Passeridae Rafinesque, 1815 |  |  |  |  |  |  |  |  |  |
| *Passer domesticus* (Linnaeus, 1758) |  | 384 | 186 | 58 | 341 | 3 | **972** | **I** | 5, 10, 19, 20, 23, 42, 54, 59, 81, 116, 119, 137, 150, 155, 161, 170, 181, 189, 198, 207, 219, 246, 309, 316, 322, 363, 364, 378, 379, 382, 385, 391, 397, 405, 415, MHNCI, WA |
