# Supplementary material for: Traditional scientific data vs. uncoordinated citizen science effort: A review of the current status and comparison of data on avifauna in Southern Brazil
Source: PLoS One. 2017 Dec 11;12(12):e0188819. doi: 10.1371/journal.pone.0188819 (PMC5724844; doi:10.1371/journal.pone.0188819)
Supplement: S4 Table — Vegetation types of records (when available): EGL–Grassland; FES–Semideciduous Tropical Forest; FOD–Tropical Rainforest; FOM–Araucaria Moist Forest; SA–Savanna. Comments: “probably misidentification” and “probably scaped” are based on Klemann-Junior personal observations. Nomenclature and taxonomic order follow CBRO [35]. * Escaped from captivity, established population confirmation is required. (DOCX) [file pone.0188819.s004.docx]

**S4 Table.** Terciary list of bird species in the state of Paraná. Vegetation types of records (when available): **EGL** – Grassland; **FES** – Semideciduous Tropical Forest; **FOD** – Tropical Rainforest; **FOM** – Araucaria Moist Forest; **SA** – Savanna. Comments: “probably misidentification” and “probably scaped” are based on Klemann-Junior personal observations. Nomenclature and taxonomic order follow CBRO [35]. * Escaped from captivity, established population confirmation is required.

| **Taxon name** | **Veg. type** | **Source (see S2 Table)** | **Comments** |
| --- | --- | --- | --- |
| Tinamiformes Huxley, 1872 |  |  |  |
| Tinamidae Gray, 1840 |  |  |  |
| *Crypturellus soui* (Hermann, 1783) |  | 295 | Probably misidentification. Southern distribution limits in Brazil: Rio de Janeiro state [61]. |
| *Crypturellus variegatus* (Gmelin, 1789) |  | 295 | Probably misidentification. Southern distribution limits in Brazil: Rio de Janeiro state [61]. |
| Anseriformes Linnaeus, 1758 |  |  |  |
| Anatidae Leach, 1820 |  |  |  |
| Anserinae Vigors, 1825 |  |  |  |
| *Cygnus melancoryphus* (Molina, 1782) |  | 324 | Without specific information. Occurrence in Paraná is based on overall species distribution. |
| Anatinae Leach, 1820 |  |  |  |
| *Neochen jubata* (Spix, 1825)* | FOM | WA | Escaped from captivity, established population confirmation is required. |
| *Anas platyrhynchos* Linnaeus, 1758* | FOM | 405 | Escaped from captivity, established population confirmation is required. |
| Galliformes Linnaeus, 1758 |  |  |  |
| Cracidae Rafinesque, 1815 |  |  |  |
| *Aburria cumanensis* (Jacquin, 1784) | FES | 298, 375 | Without specific information. Occurrence in Paraná is based on overall species distribution. |
| Phoenicopteriformes Fürbringer, 1888 |  |  |  |
| Phoenicopteridae Bonaparte, 1831 |  |  |  |
| *Phoenicopterus ruber* Linnaeus, 1758 | FOM | 324 | Probably escaped from captivity. |
| Procellariiformes Fürbringer, 1888 |  |  |  |
| Diomedeidae Gray, 1840 |  |  |  |
| *Phoebetria fusca* (Hilsenberg, 1822) |  | 281, 295 | Without specific information. Occurrence in Paraná is based on overall species distribution. |
| *Phoebetria palpebrata* (Forster, 1785) |  | 281, 295 | Without specific information. Occurrence in Paraná is based on overall species distribution. |
| *Diomedea epomophora* Lesson, 1825 |  | 324 | Without specific information. Occurrence in Paraná is based on overall species distribution. |
| Procellariidae Leach, 1820 |  |  |  |
| *Lugensa brevirostris* (Lesson, 1831) |  | 324 | Without specific information. Occurrence in Paraná is based on overall species distribution. |
| *Pachyptila desolata* (Gmelin, 1789) |  | 292 | Without specific information. Occurrence in Paraná is based on overall species distribution. |
| Hydrobatidae Mathews, 1912 |  |  |  |
| Oceanitinae Forbes, 1882 |  |  |  |
| *Fregetta grallaria* (Vieillot, 1818) |  | 324 | Without specific information. Occurrence in Paraná is based on overall species distribution. |
| *Fregetta tropica* (Gould, 1844) |  | 324 | Without specific information. Occurrence in Paraná is based on overall species distribution. |
| Hydrobatinae Mathews, 1912 |  |  |  |
| *Oceanodroma castro (Harcourt, 1851)* |  | 292 | Without specific information. Occurrence in Paraná is based on overall species distribution. |
| Pelecaniformes Sharpe, 1891 |  |  |  |
| Ardeidae Leach, 1820 |  |  |  |
| *Ardea herodias* Linnaeus, 1758 | EGL | 393 | Probably misidentification. Southern distribution limits in South America: Ecuador [61]. |
| Cathartiformes Seebohm, 1890 |  |  |  |
| Cathartidae Lafresnaye, 1839 |  |  |  |
| *Vultur gryphus* Linnaeus, 1758 | FES | 376 | Probably misidentification. Distribution: Andes, descending to sea-level in Peru and Chile [61]. |
| Charadriiformes Huxley, 1867 |  |  |  |
| Scolopaci Steijneger, 1885 |  |  |  |
| Scolopacidae Rafinesque, 1815 |  |  |  |
| *Phalaropus lobatus* (Linnaeus, 1758) |  | 324 | Without specific information. Occurrence in Paraná is based on overall species distribution. |
| Lari Sharpe, 1891 |  |  |  |
| Sternidae Vigors, 1825 |  |  |  |
| *Gelochelidon nilotica* (Gmelin, 1789) |  | 292 | Without specific information. Occurrence in Paraná is based on overall species distribution. |
| Columbiformes Latham, 1790 |  |  |  |
| Columbidae Leach, 1820 |  |  |  |
| *Patagioenas maculosa* (Temminck, 1813) | EGL, FES | 13, 17, 19, 21, 22 | Probably misidentification. Northern distribution limits in Brazil: south of Rio Grande do Sul [61]. |
| Nyctibiiformes Yuri, Kimball, Harshman, Bowie, Braun, Chojnowski, Han, Hackett, Huddleston, Moore, Reddy, Sheldon, Steadman, Witt & Braun, 2013 |  |  |  |
| Nyctibiidae Chenu & Des Murs, 1851 |  |  |  |
| *Nyctibius grandis* (Gmelin, 1789) | FES | 355 | Probably misidentification. Southern distribution limits in Brazil: Rio de Janeiro state [61]. |
| Caprimulgiformes Ridgway, 1881 |  |  |  |
| Caprimulgidae Vigors, 1825 |  |  |  |
| *Hydropsalis maculicauda* (Lawrence, 1862) | FES | 355 | Probably misidentification. Southern distribution limits in Brazil: center of São Paulo state [61]. |
| Apodiformes Peters, 1940 |  |  |  |
| Apodidae Olphe-Galliard, 1887 |  |  |  |
| *Tachornis squamata* (Cassin, 1853) | FOD | 324 | Probably misidentification. Southern distribution limits in Brazil: São Paulo state, associated to palm forests (*Mauritia spp*.) [61]. |
| Trochilidae Vigors, 1825 |  |  |  |
| Phaethornithinae Jardine, 1833 |  |  |  |
| *Phaethornis ruber* (Linnaeus, 1758) |  | 282, 324 | Without specific information. Occurrence in Paraná is based on overall species distribution. |
| Trochilinae Vigors, 1825 |  |  |  |
| *Colibri coruscans* (Gould, 1846) | FES | 355 | Probably misidentification. Distribution in Brazil: restricted to northern region (Amazonas and Roraima) [61]. |
| *Heliactin bilophus* (Temminck, 1820) |  | 282 | Probably misidentification. Southern distribution limits in Brazil: north of São Paulo state [61]. |
| Trogoniformes A. O. U., 1886 |  |  |  |
| Trogonidae Lesson, 1828 |  |  |  |
| *Trogon curucui* Linnaeus, 1766 |  | 295 | Probably misidentification. Southern distribution limits in Brazil: Mato Grosso do Sul state 61]. |
| Galbuliformes Fürbringer, 1888 |  |  |  |
| Bucconidae Horsfield, 1821 |  |  |  |
| *Monasa nigrifrons* (Spix, 1824) |  | 292 | Probably misidentification. Southern distribution limits in Brazil: midwest of São Paulo state [61]. |
| Piciformes Meyer & Wolf, 1810 |  |  |  |
| Picidae Leach, 1820 |  |  |  |
| *Campephilus leucopogon* (Valenciennes, 1826) |  | 332 | Probably misidentification. Distribution in Brazil: Rio Grande do Sul and west of Mato Grosso do Sul [61]. |
| Cariamiformes Furbringer, 1888 |  |  |  |
| Cariamidae Bonaparte, 1850 |  |  |  |
| *Chunga burmeisteri* (Hartlaub, 1860) |  | 337 | Probably misidentification. Distribution: Bolivia, Paraguay and Argentina [61]. |
| Falconiformes Bonaparte, 1831 |  |  |  |
| Falconidae Leach, 1820 |  |  |  |
| *Micrastur gilvicollis* (Vieillot, 1817) | FOM | 393 | Probably misidentification. Southern distribution limits in Brazil: Rondônia state [61]. |
| *Falco deiroleucus* Temminck, 1825 |  | 326 | Without specific information. Occurrence in Paraná is based on overall species distribution. |
| Psittaciformes Wagler, 1830 |  |  |  |
| Psittacidae Rafinesque, 1815 |  |  |  |
| *Anodorhynchus hyacinthinus* (Latham, 1790) |  | 298 | Without specific information. Occurrence in Paraná is based on overall species distribution. |
| *Anodorhynchus glaucus* (Vieillot, 1816) | FES | 357 | Without specific information. Occurrence in Paraná is based on overall species distribution. |
| *Amazona pretrei* (Temminck, 1830) |  | 324 | Without specific information. Occurrence in Paraná is based on overall species distribution. |
| Passeriformes Linnaeus, 1758 |  |  |  |
| Tyranni Wetmore & Miller, 1926 |  |  |  |
| Thamnophilida Patterson, 1987 |  |  |  |
| Thamnophilidae Swainson, 1824 |  |  |  |
| Thamnophilinae Swainson, 1824 |  |  |  |
| *Willisornis poecilinotus* (Cabanis, 1847) | EGL | 279 | Probably misidentification. Distribution in Brazil: restricted to Amazon Forest [61]. |
| Furnariida Sibley, Ahlquist & Monroe, 1988 |  |  |  |
| Furnarioidea Gray, 1840 |  |  |  |
| Furnariidae Gray, 1840 |  |  |  |
| Philydorinae Sclater & Salvin, 1873 |  |  |  |
| *Automolus rufipileatus* (Pelzeln, 1859) | EGL | 279 | Probably misidentification. Distribution in Brazil: restricted to Amazon Forest [61]. |
| Synallaxiinae De Selys-Longchamps, 1839 (1836) |  |  |  |
| *Schoeniophylax phryganophilus* (Vieillot, 1817) |  | 136 | Without specific information. Occurrence in Paraná is based on overall species distribution. |
| Tyrannida Wetmore & Miller, 1926 |  |  |  |
| Pipridae Rafinesque, 1815 |  |  |  |
| Neopelminae Tello, Moyle, Marchese & Cracraft, 2009 |  |  |  |
| *Neopelma pallescens* (Lafresnaye, 1853) | EGL, FES, FOD | 21, 22, 306, 324 | Probably misidentification. Southern distribution limits in Brazil: south of São Paulo state [61]. |
| Cotingoidea Bonaparte, 1849 |  |  |  |
| Tityridae Gray, 1840 |  |  |  |
| Tityrinae Gray, 1840 |  |  |  |
| *Pachyramphus rufus* (Boddaert, 1783) |  | 292 | Probably misidentification. Distribution in Brazil: restricted to Amazon Forest [61]. |
| Cotingidae Bonaparte, 1849 |  |  |  |
| Cotinginae Bonaparte, 1849 |  |  |  |
| *Tijuca atra* Ferrusac, 1829 |  | 292 | Probably misidentification. Southern distribution limits in Brazil: north of São Paulo state [61]. |
| Tyrannoidea Vigors, 1825 |  |  |  |
| Rhynchocyclidae Berlepsch, 1907 |  |  |  |
| Rhynchocyclinae Berlepsch, 1907 |  |  |  |
| *Rhynchocyclus olivaceus* (Temminck, 1820) |  | 298 | Probably misidentification. Southern distribution limits in Brazil: Rio de Janeiro state [61]. |
| Tyrannidae Vigors, 1825 |  |  |  |
| Tyranninae Vigors, 1825 |  |  |  |
| *Myiarchus tuberculifer* (d'Orbigny & Lafresnaye, 1837) | EGL | 309 | Probably misidentification. Southern distribution limits in Brazil: Rio de Janeiro state [61]. |
| Fluvicolinae Swainson, 1832 |  |  |  |
| *Sublegatus modestus* (Wied, 1831) | FES | 306, 324 | Probably misidentification. Distribution limits in South Brazil: Rio Grande do Sul state [61]. |
| *Alectrurus risora* (Vieillot, 1824) |  | 292 | Without specific information. Occurrence in Paraná is based on overall species distribution. |
| Passeri Linnaeus, 1758 |  |  |  |
| Corvida Wagler 1830 |  |  |  |
| Corvidae Leach, 1820 |  |  |  |
| *Cyanocorax cyanopogon* (Wied, 1821) |  | 295 | Probably misidentification. Southern distribution limits in Brazil: north of São Paulo state [61]. |
| Passerida Linnaeus, 1758 |  |  |  |
| Polioptilidae Baird, 1858 |  |  |  |
| *Polioptila dumicola* (Vieillot, 1817) |  | 292 | Without specific information. Occurrence in Paraná is based on overall species distribution. |
| Passerellidae Cabanis & Heine, 1850 |  |  |  |
| *Arremon taciturnus* (Hermann, 1783) | FOM | 292 | Probably misidentification. Southern distribution limits in Brazil: Espírito Santo state [61]. |
| Thraupidae Cabanis, 1847 |  |  |  |
| *Saltator maximus* (Statius Muller, 1776) | FES | 355 | Probably misidentification. Southern distribution limits in Brazil: Rio de Janeiro state [61]. |
| *Cyanerpes cyaneus* (Linnaeus, 1766) | EGL, FES, FOM | 21, 292 | Probably misidentification. Southern distribution limits in Brazil: south of São Paulo state [61]. |
