# Supplementary material for: Traditional scientific data vs. uncoordinated citizen science effort: A review of the current status and comparison of data on avifauna in Southern Brazil
Source: PLoS One. 2017 Dec 11;12(12):e0188819. doi: 10.1371/journal.pone.0188819 (PMC5724844; doi:10.1371/journal.pone.0188819)
Supplement: S5 Table — Vegetation types: EGL–Grassland; FES–Semideciduous Tropical Forest; FOD–Tropical Rainforest; FOM–Araucaria Moist Forest; SA–Savanna. Actual taxon: misidentifications were corrected following author’s knowledge and expertise in Paraná avifauna, and/or when misidentifications were obvious given the habitat preferences among congeners. Comments: distributions based on [27], [61]. (DOCX) [file pone.0188819.s005.docx]

**S5 Table.** Individual occurrences that need further documentation in the vegetation types listed and were thus excluded from our database due to being displaced from their known geographic distribution within the Paraná state. Vegetation types: **EGL** – Grassland; **FES** – Semideciduous Tropical Forest; **FOD** – Tropical Rainforest; **FOM** – Araucaria Moist Forest; **SA** –Savanna. **Actual taxon:** misidentifications were corrected following author’s knowledge and expertise in Paraná avifauna, and/or when misidentifications were obvious given the habitat preferences among congeners. **Comments**: distributions based on [27], [61].

| **Species** | **Actual taxon** | **Site** | **Municipality** | **Veg. type** | **Source (see S1/S2 Table)** | **Comments** |
| --- | --- | --- | --- | --- | --- | --- |
| *Agelasticus cyanopus* |  | Varanal | Telêmaco Borba | EGL | 21 | Known in south Brazil only in FES and FOD. Probably misidentification of other Icteriidae (e.g., *Molothrus bonariensis*). |
| *Agelasticus thilius* |  |  | Sertaneja | FES | 21 | Known in Paraná only in coastal marshes (FOD). Probably misidentification of other Icteriidae (e.g., *Icterus pyrrhopterus*). |
|  |  | Varanal | Telêmaco Borba | EGL | 21 |  |
| *Anabacerthia amaurotis* |  | Fazenda Santa Rita | Palmeira | EGL | 21 | Known in south Brazil only in montane or high-montane FOD. Probably misidentification of other Furnariidae (e.g., *Syndactyla rufosuperciliata*, *Heliobletus contaminates, Cichlocolaptes leucophrus*). |
|  |  | Fazenda Santa Rita | Palmeira | EGL | 19 |  |
|  |  | Reserva Natural Taruma | Palmeira/Campo Largo | EGL | 14 |  |
|  |  | Fazenda Santa Rita/PE de Vila Velha | Palmeira/Ponta Grossa | EGL | 17 |  |
|  |  | Parque Ecológico Klabin | Telêmaco Borba | EGL | 111 |  |
|  |  | Parque Ecológico Klabin | Telêmaco Borba | EGL | 14 |  |
|  |  | Imbauzinho | Telêmaco Borba | EGL | 21 |  |
|  |  | Varanal | Telêmaco Borba | EGL | 21 |  |
|  |  | Tibagi | Tibagi | EGL | 21 |  |
|  |  | Parque Estadual Mata dos Godoy | Londrina | FES | 13 |  |
|  |  | Serra do Cadeado |  | FOM | 21 |  |
|  |  | Floresta Nacional de Irati | Fernandes Pinheiro/Teixeira Soares | FOM | 111 |  |
|  |  | Floresta Nacional de Irati | Fernandes Pinheiro/Teixeira Soares | FOM | 414 |  |
|  |  | Fazendas da empresa Emilio B. Gomes & Filhos S/A, Guamirim | Irati | FOM | 194 |  |
|  |  | Fazenda Arapongas | Lapa | FOM | 205 |  |
|  |  | Reserva Natural Taruma | Palmeira/Campo Largo | FOM | 111 |  |
|  |  | Floresta Estadual do Palmito | Paranaguá | FOD | 81 |  |
| *Anabazenops fuscus* |  | Angaí | Fernandes Pinheiro | FOM | 21 | Known in south Brazil only in FOD. Probably misidentification of other Furnariidae (e.g., *Clibanornis dendrocolaptoides*). |
|  |  | Fazenda Santa Rita | Palmeira | EGL | 21 |  |
| *Anthus correndera* |  | Rio Cunhaporanga |  | EGL | 21 | Known in Santa Catarina, Paraná and São Paulo states only in coastal region (FOD). Probably misidentification of other Motacilidae (e.g., *Anthus lutescens*, *Anthus nattereri* or *Anthus hellmayri*). |
|  |  | Fazenda Santa Rita | Palmeira | EGL | 21 |  |
| *Attila rufus* |  | Capão da Imbuia | Curitiba | EGL | 10 | Known in south Brazil only in FOD. Probably misidentification of *Attila phoenicurus*. |
|  |  | Parque Estadual Mata dos Godoy | Londrina | FES | 21 |  |
|  |  | Varanal | Telêmaco Borba | EGL | 21 |  |
| *Brotogeris chiriri* | *Brotogeris tirica* | Parque Estadual do Guartelá | Tibagi | EGL | 309 | Misidentification. personal coments of authors’ records. |
| *Campylorhamphus trochilirostris* | *Campylorhamphus falcularius* | Parque Ecológico Klabin | Telêmaco Borba | EGL | 21 | Known in south Brazil only in northwest of Paraná (FES). Misidentification of *Campylorhamphus falcularius*. |
|  |  | Varanal | Telêmaco Borba | EGL | 21 |  |
|  |  |  | Tibagi | EGL | 21 |  |
| *Cantorchilus longirostris* | *Cantorchilus leucotis* | Parque Nacional de Sete Quedas | Guaíra | FES | 294 | Misidentification. personal coments of authors’ records. |
| *Chamaeza ruficauda* |  | Parque Estadual Mata dos Godoy | Londrina | FES | 21 | Known in Paraná only in East and Southeast regions (montane FOD and FOM). Probably misidentification of *Chamaeza campanisona*. |
| *Chloroceryle aenea* |  | Varanal | Telêmaco Borba | EGL | 21 | Known in Paraná only in FOD. Probably misidentification of other *Chloroceryle* spp. (e.g., *Chloroceryle americana*). |
|  |  | Varanal | Telêmaco Borba | EGL | 22 |  |
| *Cistothorus platensis* |  | Sertaneja | Sertaneja | FES | 21 | Known in Paraná only in EGL+FOM+SA. Probably misidentification of other Troglodytidae (e.g., *Troglodytes musculus*). |
| *Clibanornis dendrocolaptoides* |  | Parque Estadual Mata dos Godoy | Londrina | FES | 13 | Known in Praná only in FOM+EGL. Probably misidentification of other Furnariidae (e.g., *Syndactyla rufosuperciliata*). |
|  |  | Parque Estadual Mata dos Godoy | Londrina | FES | 14 |  |
| *Clibanornis rectirostris* |  |  | São Mateus do Sul | FOM | 366 | Known in south Brazil only in northwest of Paraná (FES). Probably misidentification of other Furnariidae (e.g., *Clibanornis dendrocolaptoides*). |
| *Conopias trivirgatus* |  | Parque Ecológico Klabin | Telêmaco Borba | EGL | 21 | Known in Paraná only in extreme East and West regions (FOD and FES). Probably misidentification of other Tyrannidae (e.g., *Myiozetetes similis*) |
| *Cypsnagra hirundinacea* |  |  | Londrina | FES | 21 | Known in south Brazil only in the savannas of Paraná state (SA). Probably misidentification. |
|  |  | Santa Rita | Palmeira | EGL | 22 |  |
|  |  |  | Sertaneja | FES | 21 |  |
| *Dacnis nigripes* |  | Imbauzinho |  | *EGL* | 21 | Known in south Brazil only in FOD. Probably misidentification of *Dacnis cayana*. |
|  |  | Estação Ecológica do Caiuá | Diamante do Norte | *FES* | 310 |  |
|  |  | Estação Ecológica do Caiuá | Diamante do Norte | *FES* | 313 |  |
| *Dysithamnus stictothorax* |  | Parque Estadual Mata dos Godoy | Londrina | FES | 21 | Known in south Brazil only in FOD. Probably misidentification of *Dysithamnus mentalis*. |
| *Dysithamnus xanthopterus* |  | Varanal | Telêmaco Borba | EGL | 21 | Known in south Brazil only in high-montane FOD. Probably misidentification of *Dysithamnus mentalis*. |
| *Formicivora rufa* |  | Guaricana | São José dos Pinhais | FOD | 356 | Known in south Brazil only in northwest of Paraná state (FES). Probably misidentification of other Thamnophilidae (e.g., *Myrmoderus squamosus*). |
| *Hylopezus nattereri* |  | Parque Estadual Mata dos Godoy | Londrina | FES | 285 | Known in Paraná only in FOM and FOD. Probably misidentification of other Grallariidae (e.g., *Chamaeza campanisona*). |
|  |  |  | Londrina | FES | 183 |  |
| *Jacamaralcyon tridactyla* |  | Represa de Guaricana | São José dos Pinhais/ Morretes/Guaratuba | FOD | 360 | Known in Paraná only in FES. Probably misidentification. |
| *Knipolegus nigerrimus* |  |  | Icaraíma | FES | 23 | Known in Paraná only in EGL+FOM and FOD. Probably misidentification of *Knipolegus cyanirostris*. |
| *Lipaugus lanioides* |  | Parque Estadual Mata dos Godoy | Londrina | FES | 21 | Known in south Brazil only in FOD. Probably misidentification. |
|  |  | Parque Estadual Mata dos Godoy | Londrina | FES | 22 |  |
|  |  | Fazenda Santa Rita | Palmeira | EGL | 21 |  |
| *Myiobius barbatus* |  | Parque Estadual Mata dos Godoy | Londrina | FES | 21 | Known in south Brazil only in FOD. Probably misidentification. |
| *Myiothlypis leucoblephara* |  | Ilha do Mel | Paranaguá | FOD | 219 | Known in Paraná only from Serra do Mar to west. Probably misidentification of *Myiothlypis rivularis*. |
| *Neothraupis fasciata* |  | Tibagi | Tibagi | EGL | 21 | Known in south Brazil only in the savannas of Paraná state (SA). Probably misidentification. |
| *Orchesticus abeillei* |  | Parque Estadual Mata dos Godoy | Londrina | FES | 13 | Known in Paraná only in EGL+FOM and FOD. Probably misidentification. |
| *Orthogonys chloricterus* |  | Varanal | Telêmaco Borba | EGL | 21 | Known in Paraná only in extreme East and West regions (FOD and FES). Probably misidentification. |
|  |  | Varanal | Telêmaco Borba | EGL | 22 |  |
| *Oxyruncus cristatus* |  | Varanal | Telêmaco Borba | EGL | 21 | Known in Paraná FOD and FES. Probably misidentification. |
| *Patagioenas speciosa* |  | Barra Branca | Morretes | FOD | 134 | Known in south Brazil only in northwest of Paraná state (FES). Probably misidentification of other Columbidae (e.g., *Patagioenas picazuro*). |
|  |  | Imbauzinho | Telêmaco Borba | EGL | 21 |  |
|  |  | Fazenda Santa Rita | Palmeira | EGL | 21 |  |
|  |  | Fazenda Santa Rita | Palmeira | EGL | 17 |  |
|  |  | Fazenda Santa Rita | Palmeira | EGL | 19 |  |
| *Phaethornis pretrei* |  | RPPN Rio Cachoeira | Antonina | FOD | 47 | Known in Paraná only in EGL+FOM and FES. Probably misidentification of other *Phaethornis* spp. (e.g., *Phaethornis eurynome*, *Phaethornis squalidus*). |
| *Pipra fasciicauda* |  | Joao Surrá | Adrianópolis | FOD | MHNCI | Known in south Brazil only in FES. Probably misidentification. |
|  |  | Pontal do Sul | Pontal do Paraná | FOD | MHNCI |  |
| *Piranga flava* |  | Parque Estadual Mata dos Godoy | Londrina | FES | 21 | Known in Paraná only in EGL+FOM and SA. Probably misidentification. |
| *Procacicus solitarius* |  | Ilha Rasa | Guaraqueçaba | FOD | 230 | Known in Paraná only in FES. Probably misidentification of *Cacicus haemorrhous*. |
| *Pteroglossus aracari* |  |  | Morretes | FOD | MHNCI | Known in Paraná only in FES. Captivity specimen. |
| *Pulsatrix perspicillata* |  | RPPN Rio Cachoeira | Antonina | FOD | 47 | Known in Paraná only in FES. Probably misidentification of *Pulsatrix koeniswaldiana*. |
| *Ramphastos toco* |  | Estação Ferroviária Marumbi, Rio Taquaral | Morretes | FOD | 363 | Known in Paraná only in FES. Misidentification or captivity specimen. |
|  |  | Parque Estadual do Guartelá | Tibagi | EGL | 309 |  |
| *Saltatricula atricollis* |  | Parque Estadual Mata dos Godoy | Londrina | FES | 21 | Known in Paraná only in EGL and SA. Probably misidentification. |
|  |  |  | Sertaneja | FES | 21 |  |
| *Schistochlamys ruficapillus* |  | Ilha das Bananas | Paranaguá | FOD | 230 | Known in Paraná only in EGL+FOM, FES, SA and high-montane FOD. Probably misidentification. |
| *Scytalopus pachecoi* | *Scytalopus speluncae* | Parque Estadual do Guartelá | Tibagi | EGL | 309 | Misidentification. personal coments of authors’ records. |
| *Syndactyla dimidiata* |  | Fazenda Santa Rita | Palmeira | EGL | 21 | Known in Paraná only in FES. Probably misidentification of *Pachyramphus* spp. (e.g., *Pachyramphus* *castaneu or Pachyramphus validus*). |
| *Tachyphonus rufus* |  | Região central do estado |  | FOM | 267 | Known in Paraná only in FES. Probably misidentification of *Tachyphonus coronatus*. |
| *Triclaria malachitacea* |  | Parque Estadual Mata dos Godoy | Londrina | FES | 13 | Known in south Brazil only in FOD and FOM. Probably misidentification of other Psittacidae. |
|  |  | Parque Estadual Mata dos Godoy | Londrina | FES | 14 |  |
|  |  | Parque Estadual Mata dos Godoy | Londrina | FES | 21 |  |
|  |  | Parque Estadual Mata dos Godoy | Londrina | FES | 334 |  |
| *Trogon viridis* |  | Parque Estadual Mata dos Godoy | Londrina | FES | 13 | Known in South Braazil only in FOD. Probably misidentification of *Trogon rufus*. |
|  |  | Parque Estadual Mata dos Godoy | Londrina | FES | 14 |  |
|  |  | Parque Estadual Mata dos Godoy | Londrina | FES | 21 |  |
