# Supplementary material for: Traditional scientific data vs. uncoordinated citizen science effort: A review of the current status and comparison of data on avifauna in Southern Brazil
Source: PLoS One. 2017 Dec 11;12(12):e0188819. doi: 10.1371/journal.pone.0188819 (PMC5724844; doi:10.1371/journal.pone.0188819)
Supplement: S6 Table — Vegetation types: EGL–Grassland; FES–Semideciduous Tropical Forest; FOD–Tropical Rainforest; FOM–Araucaria Moist Forest; SA–Savanna. (DOCX) [file pone.0188819.s006.docx]

**S6 Table.** Years with bird records in each vegetation type in Paraná state considering only data from traditional scientific references (BM), and including CS data (BM+CS). Vegetation types: **EGL** – Grassland; **FES** – Semideciduous Tropical Forest; **FOD** – Tropical Rainforest; **FOM** – Araucaria Moist Forest; **SA** –Savanna.

| **Vegetation Types** | | | | | | | | | |
| --- | --- | --- | --- | --- | --- | --- | --- | --- | --- |
| **EGL** | | **FES** | | **FOD** | | **FOM** | | **SA** | |
| **BM** | **BM+CS** | **BM** | **BM+CS** | **BM** | **BM+CS** | **BM** | **BM+CS** | **BM** | **BM+CS** |
| 1820 | 1820 | 1849 | 1849 | 1820 | 1820 | 1820 | 1820 | 1990 | 1990 |
| 1821 | 1821 | 1865 | 1865 | 1821 | 1821 | 1821 | 1821 | 1991 | 1991 |
| 1898 | 1898 | 1874 | 1874 | 1907 | 1907 | 1901 | 1901 | 1994 | 1994 |
| 1907 | 1907 | 1876 | 1876 | 1923 | 1923 | 1903 | 1903 | 1997 | 1997 |
| 1914 | 1914 | 1900 | 1900 | 1928 | 1928 | 1907 | 1907 | 1998 | 1998 |
| 1915 | 1915 | 1901 | 1901 | 1929 | 1929 | 1910 | 1910 | 1999 | 1999 |
| 1920 | 1920 | 1903 | 1903 | 1930 | 1930 | 1911 | 1911 | 2000 | 2000 |
| 1923 | 1923 | 1907 | 1907 | 1942 | 1942 | 1914 | 1914 | 2001 | 2001 |
| 1924 | 1924 | 1920 | 1920 | 1944 | 1944 | 1921 | 1921 | 2005 | 2005 |
| 1929 | 1929 | 1921 | 1921 | 1945 | 1945 | 1922 | 1922 | 2007 | 2007 |
| 1930 | 1930 | 1922 | 1922 | 1946 | 1946 | 1923 | 1923 | 2011 | 2008 |
| 1936 | 1936 | 1923 | 1923 | 1947 | 1947 | 1925 | 1925 | 2012 | 2010 |
| 1937 | 1937 | 1929 | 1929 | 1948 | 1948 | 1927 | 1927 |  | 2011 |
| 1938 | 1938 | 1930 | 1930 | 1949 | 1949 | 1928 | 1928 |  | 2012 |
| 1939 | 1939 | 1940 | 1940 | 1950 | 1950 | 1929 | 1929 |  | 2013 |
| 1940 | 1940 | 1942 | 1942 | 1951 | 1951 | 1930 | 1930 |  | 2014 |
| 1941 | 1941 | 1943 | 1943 | 1952 | 1952 | 1938 | 1938 |  | 2015 |
| 1942 | 1942 | 1944 | 1944 | 1953 | 1953 | 1939 | 1939 |  |  |
| 1943 | 1943 | 1945 | 1945 | 1954 | 1954 | 1940 | 1940 |  |  |
| 1944 | 1944 | 1946 | 1946 | 1955 | 1955 | 1942 | 1942 |  |  |
| 1945 | 1945 | 1948 | 1948 | 1957 | 1957 | 1943 | 1943 |  |  |
| 1946 | 1946 | 1950 | 1950 | 1958 | 1958 | 1944 | 1944 |  |  |
| 1947 | 1947 | 1951 | 1951 | 1960 | 1960 | 1945 | 1945 |  |  |
| 1948 | 1948 | 1954 | 1954 | 1961 | 1961 | 1946 | 1946 |  |  |
| 1949 | 1949 | 1955 | 1955 | 1965 | 1965 | 1947 | 1947 |  |  |
| 1950 | 1950 | 1956 | 1956 | 1970 | 1970 | 1948 | 1948 |  |  |
| 1951 | 1951 | 1957 | 1957 | 1972 | 1972 | 1949 | 1949 |  |  |
| 1952 | 1952 | 1958 | 1958 | 1976 | 1976 | 1950 | 1950 |  |  |
| 1953 | 1953 | 1959 | 1959 | 1977 | 1977 | 1951 | 1951 |  |  |
| 1954 | 1954 | 1960 | 1960 | 1980 | 1980 | 1952 | 1952 |  |  |
| 1955 | 1955 | 1961 | 1961 | 1981 | 1981 | 1953 | 1953 |  |  |
| 1956 | 1956 | 1962 | 1962 | 1982 | 1982 | 1954 | 1954 |  |  |
| 1958 | 1958 | 1964 | 1964 | 1983 | 1983 | 1955 | 1955 |  |  |
| 1959 | 1959 | 1965 | 1965 | 1984 | 1984 | 1956 | 1956 |  |  |
| 1960 | 1960 | 1967 | 1967 | 1985 | 1985 | 1957 | 1957 |  |  |
| 1961 | 1961 | 1968 | 1968 | 1986 | 1986 | 1958 | 1958 |  |  |
| 1964 | 1964 | 1970 | 1970 | 1987 | 1987 | 1959 | 1959 |  |  |
| 1965 | 1965 | 1972 | 1972 | 1988 | 1988 | 1960 | 1960 |  |  |
| 1966 | 1966 | 1974 | 1974 | 1989 | 1989 | 1961 | 1961 |  |  |
| 1968 | 1968 | 1975 | 1975 | 1990 | 1990 | 1962 | 1962 |  |  |
| 1969 | 1969 | 1976 | 1976 | 1991 | 1991 | 1964 | 1964 |  |  |
| 1970 | 1970 | 1980 | 1980 | 1992 | 1992 | 1965 | 1965 |  |  |
| 1971 | 1971 | 1982 | 1982 | 1993 | 1993 | 1966 | 1966 |  |  |
| 1972 | 1972 | 1983 | 1983 | 1994 | 1994 | 1967 | 1967 |  |  |
| 1973 | 1973 | 1984 | 1984 | 1995 | 1995 | 1968 | 1968 |  |  |
| 1974 | 1974 | 1986 | 1986 | 1996 | 1996 | 1969 | 1969 |  |  |
| 1975 | 1975 | 1987 | 1987 | 1997 | 1997 | 1970 | 1970 |  |  |
| 1976 | 1976 | 1988 | 1988 | 1998 | 1998 | 1971 | 1971 |  |  |
| 1978 | 1978 | 1989 | 1989 | 1999 | 1999 | 1972 | 1972 |  |  |
| 1979 | 1979 | 1990 | 1990 | 2000 | 2000 | 1973 | 1973 |  |  |
| 1980 | 1980 | 1991 | 1991 | 2001 | 2001 | 1975 | 1975 |  |  |
| 1981 | 1981 | 1992 | 1992 | 2002 | 2002 | 1980 | 1980 |  |  |
| 1982 | 1982 | 1993 | 1993 | 2003 | 2003 | 1981 | 1981 |  |  |
| 1983 | 1983 | 1994 | 1994 | 2004 | 2004 | 1982 | 1982 |  |  |
| 1984 | 1984 | 1995 | 1995 | 2005 | 2005 | 1983 | 1983 |  |  |
| 1985 | 1985 | 1996 | 1996 | 2006 | 2006 | 1984 | 1984 |  |  |
| 1986 | 1986 | 1997 | 1997 | 2007 | 2007 | 1985 | 1985 |  |  |
| 1987 | 1987 | 1998 | 1998 | 2008 | 2008 | 1986 | 1986 |  |  |
| 1988 | 1988 | 1999 | 1999 | 2009 | 2009 | 1987 | 1987 |  |  |
| 1989 | 1989 | 2000 | 2000 | 2010 | 2010 | 1988 | 1988 |  |  |
| 1990 | 1990 | 2001 | 2001 | 2011 | 2011 | 1989 | 1989 |  |  |
| 1991 | 1991 | 2002 | 2002 | 2012 | 2012 | 1990 | 1990 |  |  |
| 1992 | 1992 | 2003 | 2003 |  | 2013 | 1991 | 1991 |  |  |
| 1993 | 1993 | 2004 | 2004 |  | 2014 | 1992 | 1992 |  |  |
| 1994 | 1994 | 2005 | 2005 |  | 2015 | 1993 | 1993 |  |  |
| 1995 | 1995 | 2006 | 2006 |  |  | 1994 | 1994 |  |  |
| 1996 | 1996 | 2007 | 2007 |  |  | 1995 | 1995 |  |  |
| 1997 | 1997 | 2008 | 2008 |  |  | 1996 | 1996 |  |  |
| 1998 | 1998 | 2009 | 2009 |  |  | 1997 | 1997 |  |  |
| 1999 | 1999 | 2010 | 2010 |  |  | 1998 | 1998 |  |  |
| 2000 | 2000 | 2011 | 2011 |  |  | 1999 | 1999 |  |  |
| 2001 | 2001 | 2012 | 2012 |  |  | 2000 | 2000 |  |  |
| 2002 | 2002 | 2013 | 2013 |  |  | 2001 | 2001 |  |  |
| 2003 | 2003 | 2014 | 2014 |  |  | 2002 | 2002 |  |  |
| 2004 | 2004 |  | 2015 |  |  | 2003 | 2003 |  |  |
| 2005 | 2005 |  |  |  |  | 2004 | 2004 |  |  |
| 2006 | 2006 |  |  |  |  | 2005 | 2005 |  |  |
| 2007 | 2007 |  |  |  |  | 2006 | 2006 |  |  |
| 2008 | 2008 |  |  |  |  | 2007 | 2007 |  |  |
| 2009 | 2009 |  |  |  |  | 2008 | 2008 |  |  |
| 2010 | 2010 |  |  |  |  | 2009 | 2009 |  |  |
| 2011 | 2011 |  |  |  |  | 2010 | 2010 |  |  |
| 2012 | 2012 |  |  |  |  | 2011 | 2011 |  |  |
| 2013 | 2013 |  |  |  |  | 2012 | 2012 |  |  |
| 2014 | 2014 |  |  |  |  | 2013 | 2013 |  |  |
|  | 2015 |  |  |  |  | 2014 | 2014 |  |  |
|  |  |  |  |  |  |  | 2015 |  |  |
