# Supplementary material for: Traditional scientific data vs. uncoordinated citizen science effort: A review of the current status and comparison of data on avifauna in Southern Brazil
Source: PLoS One. 2017 Dec 11;12(12):e0188819. doi: 10.1371/journal.pone.0188819 (PMC5724844; doi:10.1371/journal.pone.0188819)
Supplement: S7 Table — Vegetation type: EGL–Grassland; FES–Semideciduous Tropical Forest; FOD–Tropical Rainforest; FOM–Araucaria Moist Forest; SA–Savanna. The total number of species in each vegetation type is presented within brackets. (DOCX) [file pone.0188819.s007.docx]

**S7 Table.** Indicator species and their Indicator Values (IndVal) in each vegetation type in Paraná state followed by specificity, fidelity and *p*-values considering only data from traditional scientific references (BM), and including CS data (BM+CS). Vegetation type: **EGL** – Grassland; **FES** – Semideciduous Tropical Forest; **FOD** – Tropical Rainforest; **FOM** – Araucaria Moist Forest; **SA** – Savanna. The total number of species in each vegetation type is presented within brackets.

| **BM** | | | | | **BM+CS** | | | | |
| --- | --- | --- | --- | --- | --- | --- | --- | --- | --- |
| **Vegetation type and Taxon** | **Specificity** | **Fidelity** | **IndVal** | ***p*-value** | **Vegetation type/Taxa** | **Specificity** | **Fidelity** | **IndVal** | ***p*-value** |
| EGL (4) |  |  |  |  | EGL (3) |  |  |  |  |
| *Sporophila hypoxantha* | 0.701 | 0.165 | 0.340 | 0.025 | *Sporophila pileata* | 0.936 | 0.163 | 0.390 | 0.003 |
| *Knipolegus nigerrimus* | 0.708 | 0.141 | 0.316 | 0.034 | *Urubitinga coronata* | 0.711 | 0.151 | 0.328 | 0.011 |
| *Sporophila pileata* | 0.868 | 0.106 | 0.303 | 0.029 | *Sporophila beltoni* | 1.000 | 0.070 | 0.264 | 0.030 |
| *Gallinago undulata* | 0.835 | 0.059 | 0.222 | 0.023 |  |  |  |  |  |
|  |  |  |  |  | FES (80) |  |  |  |  |
| FES (45) |  |  |  |  | *Thamnophilus doliatus* | 0.952 | 0.373 | 0.596 | 0.001 |
| *Ara chloropterus* | 0.970 | 0.324 | 0.561 | 0.002 | *Ara chloropterus* | 0.974 | 0.360 | 0.592 | 0.001 |
| *Penelope superciliaris* | 0.727 | 0.311 | 0.475 | 0.005 | *Psittacara leucophthalmus* | 0.765 | 0.387 | 0.544 | 0.001 |
| *Psittacara leucophthalmus* | 0.729 | 0.297 | 0.466 | 0.006 | *Pipra fasciicauda* | 1.000 | 0.293 | 0.542 | 0.001 |
| *Baryphthengus ruficapillus* | 0.616 | 0.351 | 0.465 | 0.006 | *Ramphastos toco* | 1.000 | 0.293 | 0.542 | 0.001 |
| *Arremon flavirostris* | 1.000 | 0.203 | 0.450 | 0.004 | *Penelope superciliaris* | 0.800 | 0.360 | 0.537 | 0.001 |
| *Pipra fasciicauda* | 1.000 | 0.203 | 0.450 | 0.001 | *Donacobius atricapilla* | 0.895 | 0.320 | 0.535 | 0.001 |
| *Capsiempis flaveola* | 0.756 | 0.257 | 0.441 | 0.006 | *Hylocharis chrysura* | 0.851 | 0.333 | 0.533 | 0.002 |
| *Myiopagis viridicata* | 0.782 | 0.243 | 0.436 | 0.007 | *Baryphthengus ruficapillus* | 0.655 | 0.413 | 0.520 | 0.001 |
| *Ramphocelus carbo* | 1.000 | 0.189 | 0.435 | 0.004 | *Arremon flavirostris* | 1.000 | 0.267 | 0.516 | 0.002 |
| *Corythopis delalandi* | 0.821 | 0.230 | 0.434 | 0.004 | *Ramphocelus carbo* | 1.000 | 0.253 | 0.503 | 0.001 |
| *Picumnus albosquamatus* | 1.000 | 0.176 | 0.419 | 0.004 | *Ictinia plumbea* | 0.675 | 0.373 | 0.502 | 0.001 |
| *Ramphastos toco* | 1.000 | 0.162 | 0.403 | 0.004 | *Corythopis delalandi* | 0.792 | 0.307 | 0.493 | 0.002 |
| *Nemosia pileata* | 1.000 | 0.149 | 0.386 | 0.008 | *Pteroglossus castanotis* | 0.865 | 0.280 | 0.492 | 0.001 |
| *Icterus pyrrhopterus* | 0.699 | 0.203 | 0.376 | 0.022 | *Euphonia chlorotica* | 0.737 | 0.320 | 0.486 | 0.004 |
| *Pteroglossus aracari* | 1.000 | 0.135 | 0.368 | 0.008 | *Columbina picui* | 0.725 | 0.320 | 0.482 | 0.001 |
| *Crotophaga major* | 0.699 | 0.189 | 0.364 | 0.011 | *Picumnus albosquamatus* | 1.000 | 0.227 | 0.476 | 0.001 |
| *Ara ararauna* | 1.000 | 0.122 | 0.349 | 0.014 | *Capsiempis flaveola* | 0.734 | 0.307 | 0.474 | 0.002 |
| *Aramus guarauna* | 0.686 | 0.162 | 0.333 | 0.026 | *Myiopagis viridicata* | 0.761 | 0.293 | 0.472 | 0.001 |
| *Galbula ruficauda* | 1.000 | 0.108 | 0.329 | 0.009 | *Todirostrum cinereum* | 0.701 | 0.293 | 0.453 | 0.002 |
| *Veniliornis passerinus* | 1.000 | 0.108 | 0.329 | 0.009 | *Crotophaga major* | 0.796 | 0.253 | 0.449 | 0.001 |
| *Rostrhamus sociabilis* | 0.705 | 0.149 | 0.324 | 0.018 | *Phaethornis pretrei* | 0.756 | 0.267 | 0.449 | 0.004 |
| *Brotogeris chiriri* | 1.000 | 0.095 | 0.308 | 0.021 | *Crypturellus tataupa* | 0.652 | 0.307 | 0.447 | 0.003 |
| *Campylorhamphus trochilirostris* | 1.000 | 0.095 | 0.308 | 0.025 | *Rostrhamus sociabilis* | 0.828 | 0.240 | 0.446 | 0.002 |
| *Crax fasciolata* | 1.000 | 0.095 | 0.308 | 0.029 | *Nemosia pileata* | 0.987 | 0.200 | 0.444 | 0.002 |
| *Taraba major* | 1.000 | 0.095 | 0.308 | 0.027 | *Aramus guarauna* | 0.793 | 0.240 | 0.436 | 0.004 |
| *Tigrisoma lineatum* | 0.630 | 0.149 | 0.306 | 0.035 | *Brotogeris chiriri* | 0.975 | 0.173 | 0.411 | 0.002 |
| *Gubernetes yetapa* | 0.697 | 0.122 | 0.291 | 0.035 | *Veniliornis passerinus* | 1.000 | 0.160 | 0.400 | 0.002 |
| *Cantorchilus leucotis* | 1.000 | 0.081 | 0.285 | 0.012 | *Pteroglossus aracari* | 1.000 | 0.147 | 0.383 | 0.004 |
| *Cathartes burrovianus* | 1.000 | 0.081 | 0.285 | 0.022 | *Gampsonyx swainsonii* | 0.980 | 0.147 | 0.379 | 0.004 |
| *Cranioleuca vulpina* | 1.000 | 0.081 | 0.285 | 0.011 | *Anhinga anhinga* | 0.594 | 0.240 | 0.378 | 0.018 |
| *Herpsilochmus longirostris* | 1.000 | 0.081 | 0.285 | 0.015 | *Gubernetes yetapa* | 0.744 | 0.187 | 0.373 | 0.010 |
| *Procacicus solitarius* | 1.000 | 0.081 | 0.285 | 0.024 | *Ara ararauna* | 0.915 | 0.147 | 0.366 | 0.004 |
| *Phaetusa simplex* | 0.735 | 0.108 | 0.282 | 0.048 | *Campylorhamphus trochilirostris* | 1.000 | 0.133 | 0.365 | 0.005 |
| *Vanellus cayanus* | 0.839 | 0.081 | 0.261 | 0.038 | *Cathartes burrovianus* | 1.000 | 0.133 | 0.365 | 0.003 |
| *Clibanornis rectirostris* | 1.000 | 0.068 | 0.260 | 0.047 | *Herpsilochmus longirostris* | 1.000 | 0.133 | 0.365 | 0.003 |
| *Columbina minuta* | 1.000 | 0.068 | 0.260 | 0.020 | *Taraba major* | 1.000 | 0.133 | 0.365 | 0.003 |
| *Fluvicola albiventer* | 1.000 | 0.068 | 0.260 | 0.049 | *Phaetusa simplex* | 0.805 | 0.160 | 0.359 | 0.005 |
| *Heliomaster squamosus* | 1.000 | 0.068 | 0.260 | 0.012 | *Sporophila collaris* | 0.803 | 0.160 | 0.358 | 0.005 |
| *Jabiru mycteria* | 1.000 | 0.068 | 0.260 | 0.019 | *Hemitriccus margaritaceiventer* | 0.914 | 0.133 | 0.349 | 0.007 |
| *Jacamaralcyon tridactyla* | 1.000 | 0.068 | 0.260 | 0.044 | *Cantorchilus leucotis* | 1.000 | 0.120 | 0.346 | 0.006 |
| *Hemitriccus margaritaceiventer* | 0.744 | 0.081 | 0.246 | 0.039 | *Cranioleuca vulpina* | 1.000 | 0.120 | 0.346 | 0.005 |
| *Ciconia maguari* | 0.875 | 0.068 | 0.243 | 0.036 | *Cyanocorax cyanomelas* | 1.000 | 0.120 | 0.346 | 0.004 |
| *Hylocharis sapphirina* | 0.852 | 0.068 | 0.240 | 0.022 | *Fluvicola albiventer* | 1.000 | 0.120 | 0.346 | 0.005 |
| *Crypturellus undulatus* | 1.000 | 0.054 | 0.232 | 0.047 | *Galbula ruficauda* | 1.000 | 0.120 | 0.346 | 0.005 |
| *Eupsittula aurea* | 1.000 | 0.054 | 0.232 | 0.040 | *Eupsittula aurea* | 0.963 | 0.120 | 0.340 | 0.010 |
|  |  |  |  |  | *Anhima cornuta* | 1.000 | 0.107 | 0.327 | 0.005 |
| FOD (81) |  |  |  |  | *Myiothlypis flaveola* | 1.000 | 0.107 | 0.327 | 0.004 |
| *Tangara cyanocephala* | 1.000 | 0.403 | 0.635 | 0.001 | *Procacicus solitarius* | 1.000 | 0.107 | 0.327 | 0.005 |
| *Ramphocelus bresilius* | 1.000 | 0.355 | 0.596 | 0.001 | *Ciconia maguari* | 0.868 | 0.120 | 0.323 | 0.013 |
| *Ramphodon naevius* | 0.984 | 0.339 | 0.577 | 0.001 | *Paroaria capitata* | 0.830 | 0.120 | 0.316 | 0.015 |
| *Myrmoderus squamosus* | 0.932 | 0.355 | 0.575 | 0.001 | *Elaenia spectabilis* | 0.789 | 0.120 | 0.308 | 0.023 |
| *Attila rufus* | 1.000 | 0.323 | 0.568 | 0.001 | *Clibanornis rectirostris* | 1.000 | 0.093 | 0.306 | 0.011 |
| *Manacus manacus* | 0.831 | 0.387 | 0.567 | 0.001 | *Crax fasciolata* | 1.000 | 0.093 | 0.306 | 0.009 |
| *Drymophila squamata* | 0.977 | 0.290 | 0.533 | 0.001 | *Phacellodomus ruber* | 1.000 | 0.093 | 0.306 | 0.006 |
| *Myrmotherula unicolor* | 1.000 | 0.274 | 0.524 | 0.001 | *Busarellus nigricollis* | 0.920 | 0.093 | 0.293 | 0.010 |
| *Philydor atricapillus* | 0.777 | 0.339 | 0.513 | 0.001 | *Chordeiles minor* | 0.890 | 0.093 | 0.288 | 0.003 |
| *Tangara cyanoptera* | 0.739 | 0.355 | 0.512 | 0.005 | *Heliomaster squamosus* | 0.890 | 0.093 | 0.288 | 0.010 |
| *Conopophaga melanops* | 1.000 | 0.258 | 0.508 | 0.001 | *Griseotyrannus aurantioatrocristatus* | 1.000 | 0.080 | 0.283 | 0.012 |
| *Trogon viridis* | 1.000 | 0.258 | 0.508 | 0.002 | *Progne subis* | 1.000 | 0.080 | 0.283 | 0.012 |
| *Lanio cristatus* | 1.000 | 0.242 | 0.492 | 0.002 | *Thamnophilus pelzelni* | 1.000 | 0.080 | 0.283 | 0.016 |
| *Orthogonys chloricterus* | 0.969 | 0.242 | 0.484 | 0.002 | *Amblyramphus holosericeus* | 0.954 | 0.080 | 0.276 | 0.027 |
| *Tangara seledon* | 0.761 | 0.306 | 0.483 | 0.005 | *Vanellus cayanus* | 0.839 | 0.080 | 0.259 | 0.017 |
| *Amazona brasiliensis* | 1.000 | 0.226 | 0.475 | 0.004 | *Campylorhynchus turdinus* | 1.000 | 0.067 | 0.258 | 0.017 |
| *Ramphastos vitellinus* | 1.000 | 0.226 | 0.475 | 0.001 | *Columbina minuta* | 1.000 | 0.067 | 0.258 | 0.018 |
| *Aphantochroa cirrochloris* | 0.972 | 0.226 | 0.468 | 0.001 | *Jacamaralcyon tridactyla* | 1.000 | 0.067 | 0.258 | 0.020 |
| *Drymophila ferruginea* | 1.000 | 0.210 | 0.458 | 0.002 | *Rhea americana* | 1.000 | 0.067 | 0.258 | 0.022 |
| *Hemithraupis ruficapilla* | 0.850 | 0.242 | 0.454 | 0.007 | *Sporophila leucoptera* | 0.826 | 0.080 | 0.257 | 0.042 |
| *Cantorchilus longirostris* | 0.974 | 0.210 | 0.452 | 0.005 | *Hylocharis sapphirina* | 0.851 | 0.067 | 0.238 | 0.030 |
| *Sporophila frontalis* | 0.970 | 0.210 | 0.451 | 0.001 | *Antilophia galeata* | 1.000 | 0.053 | 0.231 | 0.018 |
| *Carpornis cucullata* | 0.819 | 0.242 | 0.445 | 0.004 | *Casiornis rufus* | 1.000 | 0.053 | 0.231 | 0.018 |
| *Dendrocincla turdina* | 0.644 | 0.306 | 0.444 | 0.005 | *Crypturellus undulatus* | 1.000 | 0.053 | 0.231 | 0.019 |
| *Formicarius colma* | 1.000 | 0.194 | 0.440 | 0.002 | *Formicivora rufa* | 1.000 | 0.053 | 0.231 | 0.015 |
| *Tangara peruviana* | 1.000 | 0.194 | 0.440 | 0.003 | *Icterus croconotus* | 1.000 | 0.053 | 0.231 | 0.021 |
| *Piculus flavigula* | 0.947 | 0.194 | 0.428 | 0.007 | *Momotus momota* | 1.000 | 0.053 | 0.231 | 0.017 |
| *Todirostrum poliocephalum* | 0.849 | 0.210 | 0.422 | 0.008 | *Nyctibius aethereus* | 1.000 | 0.053 | 0.231 | 0.021 |
| *Nyctanassa violacea* | 1.000 | 0.177 | 0.421 | 0.004 | *Pulsatrix perspicillata* | 1.000 | 0.053 | 0.231 | 0.021 |
| *Platyrinchus leucoryphus* | 1.000 | 0.177 | 0.421 | 0.004 | *Tyrannus albogularis* | 0.933 | 0.053 | 0.223 | 0.023 |
| *Egretta caerulea* | 0.841 | 0.210 | 0.420 | 0.003 | *Heliornis fulica* | 0.813 | 0.053 | 0.208 | 0.050 |
| *Charadrius collaris* | 0.701 | 0.242 | 0.412 | 0.008 | *Chrysolampis mosquitus* | 0.776 | 0.053 | 0.203 | 0.043 |
| *Chlorophanes spiza* | 1.000 | 0.161 | 0.402 | 0.007 |  |  |  |  |  |
| *Conirostrum bicolor* | 1.000 | 0.161 | 0.402 | 0.005 | FOD (103) |  |  |  |  |
| *Dysithamnus stictothorax* | 1.000 | 0.161 | 0.402 | 0.004 | *Tangara cyanocephala* | 1.000 | 0.462 | 0.679 | 0.001 |
| *Ramphocaenus melanurus* | 1.000 | 0.161 | 0.402 | 0.005 | *Ramphocelus bresilius* | 0.996 | 0.462 | 0.678 | 0.001 |
| *Stymphalornis acutirostris* | 1.000 | 0.161 | 0.402 | 0.005 | *Attila rufus* | 1.000 | 0.400 | 0.632 | 0.001 |
| *Terenura maculata* | 0.905 | 0.177 | 0.401 | 0.004 | *Ramphodon naevius* | 0.996 | 0.400 | 0.631 | 0.001 |
| *Triclaria malachitacea* | 0.824 | 0.194 | 0.399 | 0.004 | *Manacus manacus* | 0.875 | 0.446 | 0.625 | 0.001 |
| *Patagioenas plumbea* | 0.545 | 0.290 | 0.398 | 0.028 | *Tangara cyanoptera* | 0.801 | 0.446 | 0.598 | 0.001 |
| *Charadrius semipalmatus* | 0.887 | 0.177 | 0.397 | 0.009 | *Myrmoderus squamosus* | 0.882 | 0.400 | 0.594 | 0.001 |
| *Ilicura militaris* | 0.956 | 0.161 | 0.393 | 0.002 | *Philydor atricapillus* | 0.897 | 0.385 | 0.587 | 0.001 |
| *Phylloscartes oustaleti* | 0.784 | 0.194 | 0.390 | 0.012 | *Tangara seledon* | 0.893 | 0.385 | 0.586 | 0.001 |
| *Eudocimus ruber* | 1.000 | 0.145 | 0.381 | 0.002 | *Lanio cristatus* | 1.000 | 0.338 | 0.582 | 0.001 |
| *Ortalis squamata* | 1.000 | 0.145 | 0.381 | 0.004 | *Trogon viridis* | 1.000 | 0.338 | 0.582 | 0.001 |
| *Phylloscartes kronei* | 1.000 | 0.145 | 0.381 | 0.003 | *Drymophila squamata* | 0.993 | 0.338 | 0.580 | 0.001 |
| *Tangara ornata* | 0.868 | 0.161 | 0.374 | 0.007 | *Aphantochroa cirrochloris* | 0.988 | 0.338 | 0.578 | 0.001 |
| *Clytolaema rubricauda* | 0.713 | 0.194 | 0.372 | 0.010 | *Amazona brasiliensis* | 1.000 | 0.323 | 0.568 | 0.001 |
| *Conopias trivirgatus* | 0.761 | 0.177 | 0.367 | 0.013 | *Myrmotherula unicolor* | 1.000 | 0.323 | 0.568 | 0.001 |
| *Rhopias gularis* | 0.757 | 0.177 | 0.367 | 0.016 | *Orthogonys chloricterus* | 0.991 | 0.323 | 0.566 | 0.001 |
| *Chloroceryle aenea* | 1.000 | 0.129 | 0.359 | 0.009 | *Euphonia pectoralis* | 0.742 | 0.431 | 0.566 | 0.003 |
| *Myiobius barbatus* | 1.000 | 0.129 | 0.359 | 0.010 | *Hemithraupis ruficapilla* | 0.927 | 0.338 | 0.560 | 0.001 |
| *Hemitriccus orbitatus* | 0.795 | 0.161 | 0.358 | 0.013 | *Conopophaga melanops* | 1.000 | 0.308 | 0.555 | 0.001 |
| *Malacoptila striata* | 0.561 | 0.226 | 0.356 | 0.022 | *Tangara peruviana* | 1.000 | 0.308 | 0.555 | 0.001 |
| *Cichlocolaptes leucophrus* | 0.691 | 0.177 | 0.350 | 0.021 | *Myiothlypis rivularis* | 0.885 | 0.338 | 0.547 | 0.001 |
| *Xenops minutus* | 0.619 | 0.194 | 0.346 | 0.028 | *Ramphastos vitellinus* | 1.000 | 0.292 | 0.541 | 0.001 |
| *Onychorhynchus swainsoni* | 0.823 | 0.145 | 0.346 | 0.008 | *Egretta caerulea* | 0.944 | 0.308 | 0.539 | 0.001 |
| *Drymophila ochropyga* | 0.739 | 0.161 | 0.345 | 0.009 | *Euphonia violacea* | 0.648 | 0.446 | 0.538 | 0.004 |
| *Oxyruncus cristatus* | 0.807 | 0.145 | 0.342 | 0.011 | *Dendrocincla turdina* | 0.777 | 0.369 | 0.536 | 0.001 |
| *Anabacerthia amaurotis* | 1.000 | 0.113 | 0.336 | 0.012 | *Drymophila ferruginea* | 1.000 | 0.277 | 0.526 | 0.001 |
| *Anabazenops fuscus* | 1.000 | 0.113 | 0.336 | 0.007 | *Cantorchilus longirostris* | 0.994 | 0.277 | 0.525 | 0.001 |
| *Dysithamnus xanthopterus* | 1.000 | 0.113 | 0.336 | 0.014 | *Chlorophanes spiza* | 1.000 | 0.262 | 0.511 | 0.001 |
| *Amadonastur lacernulatus* | 0.933 | 0.113 | 0.325 | 0.014 | *Nyctanassa violacea* | 1.000 | 0.262 | 0.511 | 0.001 |
| *Phaethornis squalidus* | 0.588 | 0.177 | 0.323 | 0.041 | *Charadrius collaris* | 0.816 | 0.308 | 0.501 | 0.002 |
| *Phyllomyias griseocapilla* | 0.906 | 0.113 | 0.320 | 0.029 | *Ortalis squamata* | 1.000 | 0.246 | 0.496 | 0.001 |
| *Myiobius atricaudus* | 0.775 | 0.129 | 0.316 | 0.015 | *Phylloscartes kronei* | 1.000 | 0.246 | 0.496 | 0.001 |
| *Crypturellus noctivagus* | 1.000 | 0.097 | 0.311 | 0.022 | *Ilicura militaris* | 0.988 | 0.246 | 0.493 | 0.001 |
| *Lipaugus lanioides* | 1.000 | 0.097 | 0.311 | 0.017 | *Formicarius colma* | 0.986 | 0.246 | 0.493 | 0.001 |
| *Lophornis chalybeus* | 1.000 | 0.097 | 0.311 | 0.015 | *Charadrius semipalmatus* | 0.972 | 0.246 | 0.489 | 0.001 |
| *Merulaxis ater* | 1.000 | 0.097 | 0.311 | 0.021 | *Todirostrum poliocephalum* | 0.859 | 0.277 | 0.488 | 0.001 |
| *Chondrohierax uncinatus* | 0.837 | 0.097 | 0.285 | 0.033 | *Piculus flavigula* | 0.963 | 0.246 | 0.487 | 0.001 |
| *Agelasticus thilius* | 1.000 | 0.081 | 0.284 | 0.016 | *Conopias trivirgatus* | 0.902 | 0.246 | 0.471 | 0.002 |
| *Buteogallus aequinoctialis* | 1.000 | 0.081 | 0.284 | 0.008 | *Sporophila frontalis* | 0.796 | 0.277 | 0.470 | 0.002 |
| *Carpornis melanocephala* | 1.000 | 0.081 | 0.284 | 0.014 | *Dysithamnus stictothorax* | 1.000 | 0.215 | 0.464 | 0.001 |
| *Chloroceryle inda* | 1.000 | 0.081 | 0.284 | 0.012 | *Eudocimus ruber* | 1.000 | 0.215 | 0.464 | 0.001 |
| *Glaucidium minutissimum* | 1.000 | 0.081 | 0.284 | 0.007 | *Ramphocaenus melanurus* | 1.000 | 0.215 | 0.464 | 0.002 |
| *Phylloscartes difficilis* | 0.787 | 0.097 | 0.276 | 0.018 | *Stymphalornis acutirostris* | 1.000 | 0.215 | 0.464 | 0.002 |
| *Ramphotrigon megacephalum* | 0.676 | 0.097 | 0.256 | 0.045 | *Malacoptila striata* | 0.731 | 0.292 | 0.462 | 0.004 |
| *Chamaeza meruloides* | 1.000 | 0.065 | 0.254 | 0.041 | *Amazilia fimbriata* | 0.925 | 0.231 | 0.462 | 0.002 |
| *Claravis geoffroyi* | 1.000 | 0.065 | 0.254 | 0.033 | *Cichlocolaptes leucophrus* | 0.865 | 0.246 | 0.462 | 0.002 |
| *Panyptila cayennensis* | 0.874 | 0.065 | 0.237 | 0.044 | *Phylloscartes oustaleti* | 0.914 | 0.231 | 0.459 | 0.001 |
|  |  |  |  |  | *Terenura maculata* | 0.914 | 0.231 | 0.459 | 0.001 |
| FOM (3) |  |  |  |  | *Triclaria malachitacea* | 0.842 | 0.246 | 0.455 | 0.002 |
| *Saltator maxillosus* | 0.666 | 0.233 | 0.393 | 0.018 | *Hemitriccus orbitatus* | 0.944 | 0.215 | 0.451 | 0.002 |
| *Netta peposaca* | 0.753 | 0.163 | 0.350 | 0.018 | *Carpornis cucullata* | 0.625 | 0.323 | 0.449 | 0.005 |
| *Gallinula melanops* | 0.822 | 0.128 | 0.324 | 0.016 | *Conirostrum bicolor* | 1.000 | 0.200 | 0.447 | 0.001 |
|  |  |  |  |  | *Tangara palmarum* | 0.758 | 0.262 | 0.445 | 0.004 |
| AS (25) |  |  |  |  | *Xenops minutus* | 0.803 | 0.246 | 0.445 | 0.003 |
| *Schistochlamys ruficapillus* | 0.897 | 0.500 | 0.670 | 0.001 | *Lophornis chalybeus* | 0.987 | 0.200 | 0.444 | 0.001 |
| *Cyanocorax cristatellus* | 0.973 | 0.417 | 0.637 | 0.001 | *Tangara ornata* | 0.841 | 0.231 | 0.441 | 0.002 |
| *Cariama cristata* | 0.805 | 0.500 | 0.634 | 0.001 | *Oxyruncus cristatus* | 0.858 | 0.215 | 0.430 | 0.003 |
| *Lepidocolaptes angustirostris* | 0.835 | 0.417 | 0.590 | 0.001 | *Myiobius barbatus* | 1.000 | 0.185 | 0.430 | 0.001 |
| *Cypsnagra hirundinacea* | 1.000 | 0.333 | 0.577 | 0.001 | *Platyrinchus leucoryphus* | 1.000 | 0.185 | 0.430 | 0.001 |
| *Neothraupis fasciata* | 1.000 | 0.333 | 0.577 | 0.001 | *Onychorhynchus swainsoni* | 0.840 | 0.215 | 0.425 | 0.003 |
| *Sicalis citrina* | 0.762 | 0.417 | 0.563 | 0.001 | *Phaethornis squalidus* | 0.772 | 0.231 | 0.422 | 0.007 |
| *Synallaxis albescens* | 0.941 | 0.333 | 0.560 | 0.001 | *Amadonastur lacernulatus* | 0.964 | 0.185 | 0.422 | 0.001 |
| *Knipolegus lophotes* | 0.737 | 0.417 | 0.554 | 0.001 | *Crypturellus noctivagus* | 1.000 | 0.169 | 0.411 | 0.001 |
| *Myiarchus tyrannulus* | 0.869 | 0.333 | 0.538 | 0.001 | *Rhopias gularis* | 0.729 | 0.231 | 0.410 | 0.004 |
| *Xolmis velatus* | 0.771 | 0.333 | 0.507 | 0.001 | *Agelasticus thilius* | 1.000 | 0.154 | 0.392 | 0.002 |
| *Elaenia chiriquensis* | 0.613 | 0.417 | 0.505 | 0.002 | *Anabacerthia amaurotis* | 1.000 | 0.154 | 0.392 | 0.002 |
| *Hirundinea ferruginea* | 0.596 | 0.417 | 0.498 | 0.001 | *Rallus longirostris* | 1.000 | 0.154 | 0.392 | 0.001 |
| *Tangara cayana* | 0.744 | 0.333 | 0.498 | 0.001 | *Phyllomyias griseocapilla* | 0.831 | 0.185 | 0.392 | 0.009 |
| *Hydropsalis torquata* | 0.659 | 0.333 | 0.469 | 0.003 | *Phylloscartes difficilis* | 0.904 | 0.154 | 0.373 | 0.006 |
| *Euscarthmus meloryphus* | 0.530 | 0.333 | 0.420 | 0.006 | *Buteogallus aequinoctialis* | 1.000 | 0.138 | 0.372 | 0.005 |
| *Elaenia cristata* | 1.000 | 0.167 | 0.408 | 0.003 | *Carpornis melanocephala* | 1.000 | 0.138 | 0.372 | 0.005 |
| *Eupetomena macroura* | 0.395 | 0.417 | 0.406 | 0.033 | *Chloroceryle aenea* | 1.000 | 0.138 | 0.372 | 0.004 |
| *Suiriri suiriri* | 0.895 | 0.167 | 0.386 | 0.003 | *Dysithamnus xanthopterus* | 1.000 | 0.138 | 0.372 | 0.003 |
| *Asio flammeus* | 0.554 | 0.250 | 0.372 | 0.010 | *Merulaxis ater* | 1.000 | 0.138 | 0.372 | 0.004 |
| *Hylophilus amaurocephalus* | 0.819 | 0.167 | 0.369 | 0.007 | *Tachuris rubrigastra* | 1.000 | 0.138 | 0.372 | 0.009 |
| *Phaeomyias murina* | 0.767 | 0.167 | 0.358 | 0.006 | *Actitis macularius* | 0.710 | 0.185 | 0.362 | 0.013 |
| *Cypseloides senex* | 0.477 | 0.250 | 0.345 | 0.028 | *Phylloscartes paulista* | 0.924 | 0.138 | 0.358 | 0.012 |
| *Cistothorus platensis* | 0.640 | 0.167 | 0.327 | 0.018 | *Orchesticus abeillei* | 0.825 | 0.154 | 0.356 | 0.013 |
| *Geranoaetus melanoleucus* | 0.520 | 0.167 | 0.294 | 0.032 | *Anabazenops fuscus* | 1.000 | 0.123 | 0.351 | 0.006 |
|  |  |  |  |  | *Dacnis nigripes* | 1.000 | 0.123 | 0.351 | 0.003 |
| EGL+FOM (48) |  |  |  |  | *Lipaugus lanioides* | 1.000 | 0.123 | 0.351 | 0.010 |
| *Cranioleuca obsoleta* | 0.950 | 0.444 | 0.650 | 0.001 | *Phleocryptes melanops* | 1.000 | 0.123 | 0.351 | 0.010 |
| *Leptasthenura setaria* | 0.982 | 0.415 | 0.638 | 0.001 | *Hemitriccus nidipendulus* | 0.666 | 0.185 | 0.351 | 0.015 |
| *Leucochloris albicollis* | 0.916 | 0.439 | 0.634 | 0.001 | *Sternula superciliaris* | 0.822 | 0.138 | 0.337 | 0.007 |
| *Sicalis flaveola* | 0.859 | 0.444 | 0.618 | 0.002 | *Chloroceryle inda* | 1.000 | 0.108 | 0.328 | 0.006 |
| *Estrilda astrild* | 0.962 | 0.392 | 0.614 | 0.001 | *Glaucidium minutissimum* | 1.000 | 0.108 | 0.328 | 0.006 |
| *Pipraeidea bonariensis* | 0.974 | 0.368 | 0.599 | 0.001 | *Heliothryx auritus* | 1.000 | 0.108 | 0.328 | 0.008 |
| *Passer domesticus* | 0.870 | 0.409 | 0.597 | 0.001 | *Hemitriccus kaempferi* | 1.000 | 0.108 | 0.328 | 0.005 |
| *Molothrus bonariensis* | 0.833 | 0.427 | 0.596 | 0.003 | *Panyptila cayennensis* | 0.969 | 0.108 | 0.323 | 0.011 |
| *Aramides saracura* | 0.832 | 0.427 | 0.596 | 0.002 | *Sporophila falcirostris* | 0.811 | 0.123 | 0.316 | 0.012 |
| *Syrigma sibilatrix* | 0.861 | 0.386 | 0.576 | 0.003 | *Accipiter bicolor* | 0.711 | 0.138 | 0.314 | 0.023 |
| *Columba livia* | 0.985 | 0.327 | 0.568 | 0.001 | *Ramphotrigon megacephalum* | 0.768 | 0.123 | 0.307 | 0.019 |
| *Gallinula galeata* | 0.850 | 0.374 | 0.564 | 0.002 | *Amaurolimnas concolor* | 0.860 | 0.108 | 0.304 | 0.015 |
| *Nycticorax nycticorax* | 0.872 | 0.363 | 0.562 | 0.003 | *Myiobius atricaudus* | 0.727 | 0.123 | 0.299 | 0.026 |
| *Satrapa icterophrys* | 0.864 | 0.357 | 0.555 | 0.001 | *Chondrohierax uncinatus* | 0.784 | 0.108 | 0.291 | 0.009 |
| *Asio clamator* | 0.945 | 0.322 | 0.551 | 0.002 | *Mimus triurus* | 0.682 | 0.123 | 0.290 | 0.048 |
| *Syndactyla rufosuperciliata* | 0.880 | 0.345 | 0.551 | 0.001 | *Pachyramphus marginatus* | 1.000 | 0.077 | 0.277 | 0.026 |
| *Phylloscartes ventralis* | 0.822 | 0.357 | 0.541 | 0.004 | *Touit melanonotus* | 1.000 | 0.077 | 0.277 | 0.032 |
| *Cranioleuca pallida* | 0.962 | 0.304 | 0.541 | 0.003 | *Claravis geoffroyi* | 1.000 | 0.062 | 0.248 | 0.013 |
| *Tangara preciosa* | 0.886 | 0.327 | 0.539 | 0.002 | *Laterallus exilis* | 1.000 | 0.062 | 0.248 | 0.013 |
| *Heliobletus contaminatus* | 0.865 | 0.333 | 0.537 | 0.002 | *Calidris subruficollis* | 0.800 | 0.077 | 0.248 | 0.036 |
| *Pachyramphus polychopterus* | 0.784 | 0.363 | 0.533 | 0.004 | *Ixobrychus exilis* | 0.930 | 0.062 | 0.239 | 0.036 |
| *Tringa solitaria* | 0.873 | 0.322 | 0.530 | 0.003 | *Pseudocolopteryx flaviventris* | 1.000 | 0.046 | 0.215 | 0.015 |
| *Elanus leucurus* | 0.900 | 0.310 | 0.528 | 0.003 |  |  |  |  |  |
| *Synallaxis cinerascens* | 0.862 | 0.322 | 0.527 | 0.003 | FOM (4) |  |  |  |  |
| *Poospiza nigrorufa* | 1.000 | 0.275 | 0.524 | 0.004 | *Hydropsalis forcipata* | 0.632 | 0.322 | 0.451 | 0.006 |
| *Piculus aurulentus* | 0.837 | 0.322 | 0.519 | 0.003 | *Dryocopus galeatus* | 0.717 | 0.161 | 0.340 | 0.014 |
| *Clibanornis dendrocolaptoides* | 1.000 | 0.263 | 0.513 | 0.005 | *Poospiza thoracica* | 0.731 | 0.149 | 0.330 | 0.018 |
| *Calliphlox amethystina* | 0.885 | 0.287 | 0.503 | 0.005 | *Polioptila lactea* | 0.763 | 0.126 | 0.311 | 0.021 |
| *Picumnus nebulosus* | 0.952 | 0.257 | 0.495 | 0.006 |  |  |  |  |  |
| *Tringa melanoleuca* | 0.923 | 0.263 | 0.493 | 0.006 | AS (14) |  |  |  |  |
| *Chaetura meridionalis* | 0.779 | 0.310 | 0.491 | 0.021 | *Cypsnagra hirundinacea* | 1.000 | 0.235 | 0.485 | 0.001 |
| *Stephanoxis lalandi* | 0.809 | 0.275 | 0.472 | 0.013 | *Neothraupis fasciata* | 1.000 | 0.235 | 0.485 | 0.001 |
| *Megascops sanctaecatarinae* | 0.963 | 0.222 | 0.463 | 0.004 | *Cyanocorax cristatellus* | 0.754 | 0.294 | 0.471 | 0.001 |
| *Accipiter striatus* | 0.865 | 0.240 | 0.455 | 0.011 | *Lepidocolaptes angustirostris* | 0.610 | 0.294 | 0.424 | 0.004 |
| *Knipolegus cyanirostris* | 0.860 | 0.234 | 0.449 | 0.017 | *Myiarchus tyrannulus* | 0.566 | 0.294 | 0.408 | 0.002 |
| *Sicalis luteola* | 0.908 | 0.216 | 0.443 | 0.013 | *Synallaxis albescens* | 0.674 | 0.235 | 0.398 | 0.002 |
| *Podilymbus podiceps* | 0.900 | 0.193 | 0.417 | 0.016 | *Schistochlamys ruficapillus* | 0.448 | 0.353 | 0.398 | 0.002 |
| *Donacospiza albifrons* | 0.978 | 0.175 | 0.414 | 0.012 | *Cariama cristata* | 0.430 | 0.353 | 0.390 | 0.010 |
| *Chordeiles nacunda* | 0.804 | 0.205 | 0.406 | 0.019 | *Elaenia chiriquensis* | 0.389 | 0.294 | 0.338 | 0.029 |
| *Coccyzus americanus* | 0.966 | 0.152 | 0.383 | 0.021 | *Elaenia cristata* | 0.898 | 0.118 | 0.325 | 0.009 |
| *Pardirallus sanguinolentus* | 0.887 | 0.164 | 0.381 | 0.025 | *Suiriri suiriri* | 0.859 | 0.118 | 0.318 | 0.003 |
| *Anthus hellmayri* | 0.960 | 0.129 | 0.351 | 0.023 | *Hymenops perspicillatus* | 0.708 | 0.118 | 0.289 | 0.020 |
| *Scytalopus iraiensis* | 1.000 | 0.123 | 0.350 | 0.025 | *Hylophilus amaurocephalus* | 0.657 | 0.118 | 0.278 | 0.020 |
| *Calidris melanotos* | 0.845 | 0.135 | 0.337 | 0.050 | *Polystictus pectoralis* | 0.939 | 0.059 | 0.235 | 0.047 |
| *Alopochelidon fucata* | 0.883 | 0.129 | 0.337 | 0.040 |  |  |  |  |  |
| *Anas versicolor* | 1.000 | 0.111 | 0.333 | 0.031 | EGL+FOD (2) |  |  |  |  |
| *Plegadis chihi* | 0.915 | 0.117 | 0.327 | 0.036 | *Clytolaema rubricauda* | 0.908 | 0.166 | 0.388 | 0.011 |
| *Leptasthenura striolata* | 1.000 | 0.094 | 0.306 | 0.036 | *Knipolegus nigerrimus* | 0.902 | 0.166 | 0.386 | 0.013 |
|  |  |  |  |  |  |  |  |  |  |
| EGL+AS (5) |  |  |  |  | EGL+FOM (41) |  |  |  |  |
| *Sporophila plumbea* | 1.000 | 0.134 | 0.366 | 0.008 | *Poospiza cabanisi* | 0.959 | 0.486 | 0.682 | 0.001 |
| *Hydropsalis anomala* | 0.809 | 0.165 | 0.365 | 0.030 | *Leptasthenura setaria* | 0.980 | 0.439 | 0.656 | 0.001 |
| *Alectrurus tricolor* | 0.940 | 0.103 | 0.311 | 0.041 | *Leucochloris albicollis* | 0.903 | 0.468 | 0.650 | 0.001 |
| *Culicivora caudacuta* | 0.945 | 0.072 | 0.261 | 0.049 | *Cranioleuca obsoleta* | 0.913 | 0.462 | 0.650 | 0.001 |
| *Emberizoides ypiranganus* | 0.939 | 0.072 | 0.260 | 0.042 | *Lepidocolaptes falcinellus* | 0.913 | 0.445 | 0.638 | 0.001 |
|  |  |  |  |  | *Sporagra magellanica* | 0.860 | 0.439 | 0.615 | 0.001 |
| FES+FOD (10) |  |  |  |  | *Heliobletus contaminatus* | 0.898 | 0.382 | 0.585 | 0.001 |
| *Selenidera maculirostris* | 0.940 | 0.257 | 0.492 | 0.004 | *Columba livia* | 0.897 | 0.382 | 0.585 | 0.001 |
| *Habia rubica* | 0.804 | 0.294 | 0.486 | 0.005 | *Tangara preciosa* | 0.896 | 0.376 | 0.580 | 0.003 |
| *Hypoedaleus guttatus* | 0.939 | 0.213 | 0.448 | 0.003 | *Embernagra platensis* | 0.887 | 0.376 | 0.577 | 0.002 |
| *Notharchus swainsoni* | 0.955 | 0.184 | 0.419 | 0.003 | *Phylloscartes ventralis* | 0.841 | 0.393 | 0.575 | 0.001 |
| *Herpsilochmus rufimarginatus* | 1.000 | 0.169 | 0.411 | 0.008 | *Estrilda astrild* | 0.794 | 0.410 | 0.571 | 0.001 |
| *Tinamus solitarius* | 0.891 | 0.184 | 0.405 | 0.011 | *Pipraeidea bonariensis* | 0.835 | 0.387 | 0.569 | 0.002 |
| *Anabacerthia lichtensteini* | 0.862 | 0.169 | 0.382 | 0.014 | *Piculus aurulentus* | 0.900 | 0.358 | 0.568 | 0.002 |
| *Piprites chloris* | 0.910 | 0.103 | 0.306 | 0.041 | *Syndactyla rufosuperciliata* | 0.815 | 0.393 | 0.566 | 0.001 |
| *Sporophila lineola* | 0.956 | 0.081 | 0.278 | 0.040 | *Cacicus chrysopterus* | 0.857 | 0.370 | 0.563 | 0.001 |
| *Sternula superciliaris* | 1.000 | 0.051 | 0.227 | 0.040 | *Cranioleuca pallida* | 0.939 | 0.335 | 0.561 | 0.001 |
|  |  |  |  |  | *Clibanornis dendrocolaptoides* | 1.000 | 0.306 | 0.553 | 0.001 |
| FES+FOM (2) |  |  |  |  | *Serpophaga nigricans* | 0.818 | 0.364 | 0.546 | 0.001 |
| *Aratinga auricapillus* | 0.966 | 0.125 | 0.348 | 0.025 | *Calliphlox amethystina* | 0.896 | 0.329 | 0.543 | 0.001 |
| *Anhinga anhinga* | 0.883 | 0.125 | 0.332 | 0.025 | *Poospiza nigrorufa* | 1.000 | 0.289 | 0.538 | 0.002 |
|  |  |  |  |  | *Picumnus nebulosus* | 0.957 | 0.289 | 0.526 | 0.002 |
| FES+AS (12) |  |  |  |  | *Stephanoxis lalandi* | 0.853 | 0.312 | 0.516 | 0.003 |
| *Thamnophilus doliatus* | 1.000 | 0.279 | 0.528 | 0.001 | *Stephanophorus diadematus* | 0.885 | 0.301 | 0.516 | 0.002 |
| *Turdus leucomelas* | 0.767 | 0.349 | 0.517 | 0.003 | *Megascops sanctaecatarinae* | 0.936 | 0.277 | 0.510 | 0.002 |
| *Hylocharis chrysura* | 0.961 | 0.233 | 0.473 | 0.002 | *Asio clamator* | 0.773 | 0.335 | 0.509 | 0.003 |
| *Donacobius atricapilla* | 0.973 | 0.198 | 0.439 | 0.002 | *Knipolegus cyanirostris* | 0.874 | 0.283 | 0.498 | 0.002 |
| *Synallaxis frontalis* | 0.790 | 0.233 | 0.429 | 0.006 | *Donacospiza albifrons* | 0.990 | 0.231 | 0.478 | 0.003 |
| *Todirostrum cinereum* | 0.853 | 0.198 | 0.411 | 0.006 | *Podilymbus podiceps* | 0.841 | 0.254 | 0.463 | 0.004 |
| *Ictinia plumbea* | 0.653 | 0.256 | 0.409 | 0.012 | *Saltator maxillosus* | 0.913 | 0.214 | 0.442 | 0.002 |
| *Columbina picui* | 0.754 | 0.221 | 0.408 | 0.020 | *Anthus hellmayri* | 0.986 | 0.168 | 0.407 | 0.003 |
| *Phaethornis pretrei* | 0.800 | 0.174 | 0.373 | 0.011 | *Alopochelidon fucata* | 0.892 | 0.179 | 0.400 | 0.004 |
| *Pteroglossus castanotis* | 0.898 | 0.151 | 0.368 | 0.009 | *Coccyzus americanus* | 0.909 | 0.173 | 0.397 | 0.004 |
| *Amazilia lactea* | 0.844 | 0.128 | 0.329 | 0.020 | *Scytalopus iraiensis* | 1.000 | 0.150 | 0.388 | 0.006 |
| *Picumnus cirratus* | 0.917 | 0.116 | 0.327 | 0.027 | *Sporophila hypoxantha* | 0.961 | 0.156 | 0.387 | 0.006 |
|  |  |  |  |  | *Anas versicolor* | 0.984 | 0.139 | 0.370 | 0.014 |
| FOD+FOM (5) |  |  |  |  | *Netta peposaca* | 0.936 | 0.139 | 0.360 | 0.013 |
| *Hydropsalis forcipata* | 0.838 | 0.216 | 0.426 | 0.010 | *Leptasthenura striolata* | 1.000 | 0.116 | 0.340 | 0.016 |
| *Pseudastur polionotus* | 0.825 | 0.155 | 0.358 | 0.030 | *Gallinula melanops* | 0.964 | 0.092 | 0.299 | 0.035 |
| *Scytalopus speluncae* | 0.841 | 0.122 | 0.320 | 0.046 | *Phacellodomus striaticollis* | 1.000 | 0.081 | 0.284 | 0.027 |
| *Poospiza thoracica* | 0.962 | 0.095 | 0.302 | 0.042 | *Laterallus leucopyrrhus* | 0.943 | 0.081 | 0.276 | 0.047 |
| *Accipiter superciliosus* | 1.000 | 0.068 | 0.260 | 0.039 |  |  |  |  |  |
|  |  |  |  |  | EGL+AS (8) |  |  |  |  |
| FOD+AS (3) |  |  |  |  | *Sicalis citrina* | 0.938 | 0.214 | 0.448 | 0.003 |
| *Myiothlypis rivularis* | 0.721 | 0.230 | 0.407 | 0.016 | *Knipolegus lophotes* | 0.807 | 0.223 | 0.424 | 0.005 |
| *Tangara palmarum* | 0.693 | 0.149 | 0.321 | 0.038 | *Hydropsalis anomala* | 0.843 | 0.184 | 0.394 | 0.007 |
| *Chordeiles acutipennis* | 0.948 | 0.095 | 0.299 | 0.030 | *Sporophila plumbea* | 1.000 | 0.136 | 0.369 | 0.003 |
|  |  |  |  |  | *Geranoaetus melanoleucus* | 0.826 | 0.146 | 0.347 | 0.011 |
| FOM+AS (1) |  |  |  |  | *Cistothorus platensis* | 0.921 | 0.126 | 0.341 | 0.019 |
| *Rhynchotus rufescens* | 0.725 | 0.296 | 0.463 | 0.007 | *Culicivora caudacuta* | 0.848 | 0.126 | 0.327 | 0.010 |
|  |  |  |  |  | *Alectrurus tricolor* | 0.933 | 0.097 | 0.301 | 0.015 |
| EGL+FES+FOD (2) |  |  |  |  |  |  |  |  |  |
| *Euphonia pectoralis* | 0.964 | 0.240 | 0.481 | 0.007 | FES+FOD (23) |  |  |  |  |
| *Aramides cajaneus* | 0.967 | 0.149 | 0.380 | 0.020 | *Celeus flavescens* | 0.896 | 0.386 | 0.588 | 0.001 |
|  |  |  |  |  | *Selenidera maculirostris* | 0.974 | 0.350 | 0.584 | 0.001 |
| EGL+FES+FOM (3) |  |  |  |  | *Habia rubica* | 0.893 | 0.357 | 0.565 | 0.001 |
| *Machetornis rixosa* | 0.927 | 0.339 | 0.560 | 0.005 | *Automolus leucophthalmus* | 0.914 | 0.321 | 0.542 | 0.001 |
| *Pyrrhocoma ruficeps* | 0.984 | 0.184 | 0.425 | 0.016 | *Hypoedaleus guttatus* | 0.956 | 0.286 | 0.523 | 0.001 |
| *Arundinicola leucocephala* | 1.000 | 0.139 | 0.373 | 0.032 | *Myiornis auricularis* | 0.826 | 0.307 | 0.504 | 0.005 |
|  |  |  |  |  | *Forpus xanthopterygius* | 0.861 | 0.279 | 0.490 | 0.004 |
| EGL+FOD+FOM (15) |  |  |  |  | *Herpsilochmus rufimarginatus* | 0.976 | 0.243 | 0.487 | 0.002 |
| *Turdus flavipes* | 0.993 | 0.348 | 0.588 | 0.002 | *Pyriglena leucoptera* | 0.754 | 0.314 | 0.487 | 0.049 |
| *Turdus albicollis* | 0.875 | 0.391 | 0.585 | 0.011 | *Amazilia versicolor* | 0.812 | 0.286 | 0.482 | 0.003 |
| *Brotogeris tirica* | 0.981 | 0.343 | 0.580 | 0.002 | *Anabacerthia lichtensteini* | 0.913 | 0.243 | 0.471 | 0.001 |
| *Cyanocorax caeruleus* | 0.988 | 0.339 | 0.579 | 0.001 | *Aramides cajaneus* | 0.883 | 0.243 | 0.463 | 0.006 |
| *Ardea alba* | 0.908 | 0.318 | 0.537 | 0.028 | *Tinamus solitarius* | 0.892 | 0.236 | 0.459 | 0.004 |
| *Chaetura cinereiventris* | 0.926 | 0.279 | 0.508 | 0.003 | *Notharchus swainsoni* | 0.900 | 0.214 | 0.439 | 0.003 |
| *Procnias nudicollis* | 0.939 | 0.249 | 0.483 | 0.007 | *Aburria jacutinga* | 0.868 | 0.200 | 0.417 | 0.012 |
| *Haplospiza unicolor* | 0.922 | 0.223 | 0.454 | 0.017 | *Tigrisoma lineatum* | 0.849 | 0.186 | 0.397 | 0.006 |
| *Attila phoenicurus* | 0.968 | 0.206 | 0.447 | 0.016 | *Rynchops niger* | 0.827 | 0.157 | 0.360 | 0.018 |
| *Sclerurus scansor* | 0.927 | 0.210 | 0.442 | 0.014 | *Psilorhamphus guttatus* | 0.799 | 0.150 | 0.346 | 0.030 |
| *Strix hylophila* | 0.908 | 0.193 | 0.419 | 0.033 | *Piprites chloris* | 0.936 | 0.114 | 0.327 | 0.017 |
| *Batara cinerea* | 0.942 | 0.176 | 0.407 | 0.028 | *Tiaris fuliginosus* | 0.881 | 0.114 | 0.317 | 0.046 |
| *Phyllomyias fasciatus* | 0.947 | 0.163 | 0.393 | 0.030 | *Agelasticus cyanopus* | 1.000 | 0.086 | 0.293 | 0.028 |
| *Euphonia chalybea* | 0.966 | 0.159 | 0.392 | 0.026 | *Tigrisoma fasciatum* | 1.000 | 0.071 | 0.267 | 0.038 |
| *Spizaetus tyrannus* | 0.924 | 0.155 | 0.378 | 0.041 | *Riparia riparia* | 1.000 | 0.057 | 0.239 | 0.050 |
|  |  |  |  |  |  |  |  |  |  |
| EGL+FOM+AS (46) |  |  |  |  | FES+FOM (12) |  |  |  |  |
| *Poospiza cabanisi* | 0.986 | 0.437 | 0.657 | 0.001 | *Hemithraupis guira* | 0.872 | 0.284 | 0.498 | 0.003 |
| *Colaptes campestris* | 0.899 | 0.475 | 0.654 | 0.001 | *Tachycineta albiventer* | 0.854 | 0.278 | 0.487 | 0.004 |
| *Zonotrichia capensis* | 0.866 | 0.470 | 0.638 | 0.001 | *Conirostrum speciosum* | 0.874 | 0.259 | 0.476 | 0.002 |
| *Sporagra magellanica* | 0.957 | 0.421 | 0.635 | 0.001 | *Cissopis leverianus* | 0.891 | 0.253 | 0.475 | 0.006 |
| *Pipraeidea melanonota* | 0.898 | 0.426 | 0.619 | 0.001 | *Icterus pyrrhopterus* | 0.948 | 0.222 | 0.459 | 0.004 |
| *Veniliornis spilogaster* | 0.861 | 0.443 | 0.617 | 0.002 | *Glaucidium brasilianum* | 0.853 | 0.216 | 0.429 | 0.006 |
| *Athene cunicularia* | 0.857 | 0.443 | 0.616 | 0.001 | *Aratinga auricapillus* | 0.990 | 0.185 | 0.428 | 0.005 |
| *Falco sparverius* | 0.874 | 0.432 | 0.614 | 0.002 | *Primolius maracana* | 0.948 | 0.117 | 0.333 | 0.017 |
| *Turdus amaurochalinus* | 0.801 | 0.465 | 0.610 | 0.008 | *Falco rufigularis* | 0.977 | 0.105 | 0.320 | 0.019 |
| *Cyclarhis gujanensis* | 0.802 | 0.459 | 0.607 | 0.022 | *Claravis pretiosa* | 0.912 | 0.099 | 0.300 | 0.028 |
| *Lepidocolaptes falcinellus* | 0.942 | 0.388 | 0.604 | 0.001 | *Heliomaster furcifer* | 1.000 | 0.074 | 0.272 | 0.029 |
| *Tyrannus savana* | 0.857 | 0.421 | 0.601 | 0.001 | *Nyctiphrynus ocellatus* | 1.000 | 0.074 | 0.272 | 0.036 |
| *Myiothlypis leucoblephara* | 0.894 | 0.399 | 0.597 | 0.001 |  |  |  |  |  |
| *Chlorostilbon lucidus* | 0.867 | 0.404 | 0.592 | 0.001 | FES+AS (6) |  |  |  |  |
| *Serpophaga subcristata* | 0.916 | 0.383 | 0.592 | 0.001 | *Synallaxis frontalis* | 0.847 | 0.283 | 0.489 | 0.001 |
| *Thamnophilus ruficapillus* | 0.937 | 0.372 | 0.590 | 0.001 | *Tangara cayana* | 0.804 | 0.196 | 0.397 | 0.004 |
| *Vanellus chilensis* | 0.865 | 0.399 | 0.587 | 0.009 | *Amazilia lactea* | 0.737 | 0.207 | 0.390 | 0.011 |
| *Caracara plancus* | 0.839 | 0.404 | 0.583 | 0.002 | *Picumnus cirratus* | 0.909 | 0.163 | 0.385 | 0.002 |
| *Pygochelidon cyanoleuca* | 0.837 | 0.399 | 0.578 | 0.002 | *Jabiru mycteria* | 1.000 | 0.109 | 0.330 | 0.010 |
| *Myiophobus fasciatus* | 0.869 | 0.383 | 0.576 | 0.004 | *Dendrocygna autumnalis* | 0.835 | 0.098 | 0.286 | 0.034 |
| *Embernagra platensis* | 0.958 | 0.344 | 0.574 | 0.001 |  |  |  |  |  |
| *Elaenia parvirostris* | 0.957 | 0.333 | 0.565 | 0.002 | FOD+FOM (2) |  |  |  |  |
| *Elaenia mesoleuca* | 0.930 | 0.339 | 0.561 | 0.003 | *Pseudastur polionotus* | 0.849 | 0.217 | 0.429 | 0.005 |
| *Tachycineta leucorrhoa* | 0.912 | 0.333 | 0.551 | 0.002 | *Accipiter superciliosus* | 1.000 | 0.072 | 0.269 | 0.042 |
| *Serpophaga nigricans* | 0.926 | 0.328 | 0.551 | 0.002 |  |  |  |  |  |
| *Pardirallus nigricans* | 0.868 | 0.344 | 0.547 | 0.001 | FOD+AS (2) |  |  |  |  |
| *Tyto furcata* | 0.867 | 0.344 | 0.546 | 0.003 | *Pluvialis dominica* | 0.720 | 0.159 | 0.338 | 0.026 |
| *Theristicus caudatus* | 0.967 | 0.301 | 0.539 | 0.003 | *Chordeiles acutipennis* | 0.746 | 0.098 | 0.270 | 0.043 |
| *Colibri serrirostris* | 0.962 | 0.301 | 0.538 | 0.001 |  |  |  |  |  |
| *Turdus subalaris* | 0.872 | 0.328 | 0.535 | 0.003 | EGL+FES+FOM (27) |  |  |  |  |
| *Anumbius annumbi* | 0.966 | 0.284 | 0.524 | 0.003 | *Cyanocorax chrysops* | 0.973 | 0.411 | 0.632 | 0.001 |
| *Pseudoleistes guirahuro* | 0.957 | 0.284 | 0.521 | 0.002 | *Leptotila verreauxi* | 0.896 | 0.432 | 0.622 | 0.002 |
| *Progne chalybea* | 0.858 | 0.317 | 0.521 | 0.007 | *Colaptes melanochloros* | 0.925 | 0.411 | 0.617 | 0.001 |
| *Cacicus chrysopterus* | 0.886 | 0.306 | 0.521 | 0.004 | *Guira guira* | 0.913 | 0.407 | 0.610 | 0.009 |
| *Tringa flavipes* | 0.862 | 0.301 | 0.509 | 0.007 | *Pipraeidea melanonota* | 0.894 | 0.395 | 0.594 | 0.006 |
| *Gallinago paraguaiae* | 0.902 | 0.268 | 0.491 | 0.007 | *Myiothlypis leucoblephara* | 0.941 | 0.375 | 0.594 | 0.001 |
| *Emberizoides herbicola* | 0.964 | 0.235 | 0.476 | 0.005 | *Furnarius rufus* | 0.919 | 0.383 | 0.593 | 0.008 |
| *Stephanophorus diadematus* | 0.861 | 0.257 | 0.470 | 0.012 | *Passer domesticus* | 0.910 | 0.363 | 0.575 | 0.002 |
| *Chrysomus ruficapillus* | 0.901 | 0.235 | 0.460 | 0.011 | *Poecilotriccus plumbeiceps* | 0.910 | 0.355 | 0.568 | 0.001 |
| *Himantopus melanurus* | 0.941 | 0.219 | 0.454 | 0.006 | *Elanus leucurus* | 0.970 | 0.327 | 0.563 | 0.001 |
| *Xolmis cinereus* | 0.948 | 0.202 | 0.438 | 0.011 | *Synallaxis cinerascens* | 0.977 | 0.323 | 0.561 | 0.001 |
| *Heterospizias meridionalis* | 0.907 | 0.202 | 0.428 | 0.010 | *Pachyramphus polychopterus* | 0.899 | 0.347 | 0.558 | 0.009 |
| *Buteo brachyurus* | 0.866 | 0.186 | 0.401 | 0.029 | *Tyto furcata* | 0.931 | 0.331 | 0.555 | 0.001 |
| *Amazona vinacea* | 0.852 | 0.175 | 0.386 | 0.034 | *Pardirallus nigricans* | 0.906 | 0.339 | 0.554 | 0.016 |
| *Geranoaetus albicaudatus* | 0.842 | 0.153 | 0.359 | 0.037 | *Trogon surrucura* | 0.920 | 0.331 | 0.552 | 0.028 |
| *Xolmis dominicanus* | 0.905 | 0.142 | 0.359 | 0.035 | *Tringa solitaria* | 0.956 | 0.290 | 0.527 | 0.001 |
|  |  |  |  |  | *Pseudoleistes guirahuro* | 0.968 | 0.258 | 0.500 | 0.003 |
| FES+FOD+FOM (7) |  |  |  |  | *Pyrrhocoma ruficeps* | 0.994 | 0.234 | 0.482 | 0.003 |
| *Euphonia violacea* | 0.924 | 0.243 | 0.474 | 0.021 | *Coccyzus melacoryphus* | 0.889 | 0.254 | 0.475 | 0.013 |
| *Melanerpes flavifrons* | 0.881 | 0.248 | 0.467 | 0.031 | *Accipiter striatus* | 0.945 | 0.222 | 0.458 | 0.009 |
| *Myiozetetes similis* | 0.886 | 0.234 | 0.456 | 0.031 | *Cyanoloxia glaucocaerulea* | 0.955 | 0.190 | 0.426 | 0.017 |
| *Mackenziaena severa* | 0.914 | 0.180 | 0.406 | 0.024 | *Sarcoramphus papa* | 0.947 | 0.169 | 0.400 | 0.023 |
| *Eleoscytalopus indigoticus* | 0.937 | 0.171 | 0.400 | 0.024 | *Geranospiza caerulescens* | 0.907 | 0.141 | 0.358 | 0.047 |
| *Tityra inquisitor* | 0.940 | 0.153 | 0.379 | 0.028 | *Chlorophonia cyanea* | 0.980 | 0.129 | 0.356 | 0.031 |
| *Aburria jacutinga* | 1.000 | 0.113 | 0.336 | 0.032 | *Phylloscartes eximius* | 1.000 | 0.117 | 0.342 | 0.020 |
|  |  |  |  |  | *Paroaria coronata* | 1.000 | 0.109 | 0.330 | 0.042 |
| FES+FOD+AS (4) |  |  |  |  | *Phibalura flavirostris* | 1.000 | 0.101 | 0.318 | 0.043 |
| *Celeus flavescens* | 0.878 | 0.277 | 0.493 | 0.006 |  |  |  |  |  |
| *Automolus leucophthalmus* | 0.912 | 0.243 | 0.471 | 0.004 | EGL+FES+AS (1) |  |  |  |  |
| *Pyriglena leucoptera* | 0.800 | 0.243 | 0.441 | 0.044 | *Asio flammeus* | 0.863 | 0.140 | 0.348 | 0.031 |
| *Forpus xanthopterygius* | 0.869 | 0.196 | 0.413 | 0.025 |  |  |  |  |  |
|  |  |  |  |  | EGL+FOD+FOM (24) |  |  |  |  |
| FES+FOM+AS (3) |  |  |  |  | *Cyanocorax caeruleus* | 0.995 | 0.399 | 0.630 | 0.001 |
| *Hemithraupis guira* | 0.856 | 0.221 | 0.435 | 0.012 | *Turdus flavipes* | 0.991 | 0.391 | 0.622 | 0.001 |
| *Crypturellus parvirostris* | 0.933 | 0.192 | 0.423 | 0.005 | *Brotogeris tirica* | 0.979 | 0.382 | 0.612 | 0.001 |
| *Tachycineta albiventer* | 0.889 | 0.198 | 0.419 | 0.008 | *Streptoprocne zonaris* | 0.887 | 0.324 | 0.536 | 0.031 |
|  |  |  |  |  | *Procnias nudicollis* | 0.969 | 0.294 | 0.534 | 0.003 |
| EGL+FES+FOD+FOM (4) |  |  |  |  | *Mionectes rufiventris* | 0.922 | 0.307 | 0.532 | 0.005 |
| *Picumnus temminckii* | 0.979 | 0.371 | 0.603 | 0.033 | *Chaetura cinereiventris* | 0.905 | 0.294 | 0.516 | 0.004 |
| *Phalacrocorax brasilianus* | 1.000 | 0.319 | 0.565 | 0.013 | *Attila phoenicurus* | 0.967 | 0.265 | 0.506 | 0.003 |
| *Dysithamnus mentalis* | 0.974 | 0.319 | 0.557 | 0.044 | *Haplospiza unicolor* | 0.889 | 0.277 | 0.497 | 0.013 |
| *Butorides striata* | 1.000 | 0.283 | 0.532 | 0.026 | *Hylophilus poicilotis* | 0.905 | 0.269 | 0.493 | 0.013 |
|  |  |  |  |  | *Sclerurus scansor* | 0.942 | 0.256 | 0.491 | 0.003 |
| EGL+FES+FOM+AS (31) |  |  |  |  | *Batara cinerea* | 0.971 | 0.223 | 0.465 | 0.004 |
| *Troglodytes musculus* | 0.934 | 0.405 | 0.615 | 0.012 | *Pyroderus scutatus* | 0.895 | 0.240 | 0.463 | 0.011 |
| *Rupornis magnirostris* | 0.924 | 0.401 | 0.609 | 0.017 | *Euphonia chalybea* | 0.935 | 0.219 | 0.452 | 0.007 |
| *Columbina talpacoti* | 0.950 | 0.385 | 0.605 | 0.003 | *Patagioenas plumbea* | 0.957 | 0.206 | 0.444 | 0.006 |
| *Setophaga pitiayumi* | 0.924 | 0.377 | 0.590 | 0.014 | *Strix hylophila* | 0.916 | 0.214 | 0.443 | 0.009 |
| *Guira guira* | 0.961 | 0.362 | 0.590 | 0.002 | *Phyllomyias fasciatus* | 0.932 | 0.193 | 0.424 | 0.014 |
| *Cyanocorax chrysops* | 1.000 | 0.346 | 0.588 | 0.004 | *Spizaetus tyrannus* | 0.963 | 0.181 | 0.417 | 0.008 |
| *Piaya cayana* | 0.932 | 0.370 | 0.587 | 0.007 | *Muscipipra vetula* | 0.965 | 0.168 | 0.403 | 0.019 |
| *Zenaida auriculata* | 0.993 | 0.342 | 0.583 | 0.001 | *Tangara desmaresti* | 1.000 | 0.147 | 0.383 | 0.011 |
| *Sporophila caerulescens* | 0.921 | 0.366 | 0.580 | 0.029 | *Asio stygius* | 0.984 | 0.147 | 0.380 | 0.010 |
| *Camptostoma obsoletum* | 0.932 | 0.358 | 0.578 | 0.002 | *Scytalopus speluncae* | 1.000 | 0.135 | 0.367 | 0.018 |
| *Colaptes melanochloros* | 0.948 | 0.350 | 0.576 | 0.001 | *Hylopezus nattereri* | 1.000 | 0.126 | 0.355 | 0.028 |
| *Crotophaga ani* | 0.924 | 0.358 | 0.575 | 0.012 | *Streptoprocne biscutata* | 0.954 | 0.122 | 0.341 | 0.047 |
| *Volatinia jacarina* | 0.981 | 0.331 | 0.570 | 0.002 |  |  |  |  |  |
| *Furnarius rufus* | 0.956 | 0.339 | 0.569 | 0.002 | EGL+FOM+AS (13) |  |  |  |  |
| *Patagioenas picazuro* | 0.973 | 0.319 | 0.557 | 0.002 | *Thamnophilus ruficapillus* | 0.883 | 0.395 | 0.590 | 0.001 |
| *Amazonetta brasiliensis* | 0.950 | 0.304 | 0.537 | 0.004 | *Elaenia mesoleuca* | 0.918 | 0.353 | 0.569 | 0.001 |
| *Elaenia flavogaster* | 0.920 | 0.307 | 0.532 | 0.037 | *Anumbius annumbi* | 0.979 | 0.326 | 0.565 | 0.001 |
| *Poecilotriccus plumbeiceps* | 0.932 | 0.304 | 0.532 | 0.004 | *Colibri serrirostris* | 0.938 | 0.326 | 0.553 | 0.001 |
| *Mimus saturninus* | 0.989 | 0.269 | 0.515 | 0.005 | *Elaenia parvirostris* | 0.816 | 0.347 | 0.532 | 0.003 |
| *Myiarchus swainsoni* | 0.923 | 0.280 | 0.508 | 0.046 | *Theristicus caudatus* | 0.857 | 0.321 | 0.524 | 0.002 |
| *Ammodramus humeralis* | 1.000 | 0.253 | 0.503 | 0.002 | *Xolmis cinereus* | 0.946 | 0.253 | 0.489 | 0.001 |
| *Melanerpes candidus* | 0.959 | 0.261 | 0.500 | 0.005 | *Amazona vinacea* | 0.860 | 0.216 | 0.431 | 0.008 |
| *Amazona aestiva* | 1.000 | 0.249 | 0.499 | 0.006 | *Pardirallus sanguinolentus* | 0.873 | 0.200 | 0.418 | 0.007 |
| *Gnorimopsar chopi* | 0.962 | 0.249 | 0.489 | 0.006 | *Xolmis dominicanus* | 0.927 | 0.174 | 0.401 | 0.007 |
| *Lanio cucullatus* | 0.969 | 0.230 | 0.472 | 0.009 | *Calidris melanotos* | 0.900 | 0.163 | 0.383 | 0.017 |
| *Nothura maculosa* | 1.000 | 0.222 | 0.471 | 0.006 | *Piranga flava* | 1.000 | 0.126 | 0.355 | 0.011 |
| *Dendrocygna viduata* | 0.979 | 0.210 | 0.454 | 0.009 | *Emberizoides ypiranganus* | 0.982 | 0.095 | 0.305 | 0.034 |
| *Coccyzus melacoryphus* | 0.959 | 0.206 | 0.445 | 0.013 |  |  |  |  |  |
| *Columbina squammata* | 0.982 | 0.160 | 0.396 | 0.027 | FES+FOD+FOM (12) |  |  |  |  |
| *Nystalus chacuru* | 1.000 | 0.148 | 0.385 | 0.024 | *Melanerpes flavifrons* | 0.938 | 0.322 | 0.549 | 0.001 |
| *Falco femoralis* | 0.966 | 0.148 | 0.378 | 0.048 | *Myiozetetes similis* | 0.919 | 0.295 | 0.521 | 0.002 |
|  |  |  |  |  | *Campephilus robustus* | 0.887 | 0.260 | 0.480 | 0.007 |
| EGL+FOD+FOM+AS (10) |  |  |  |  | *Cnemotriccus fuscatus* | 0.901 | 0.251 | 0.476 | 0.039 |
| *Turdus rufiventris* | 0.942 | 0.433 | 0.638 | 0.002 | *Mackenziaena severa* | 0.959 | 0.234 | 0.473 | 0.003 |
| *Chiroxiphia caudata* | 0.917 | 0.392 | 0.600 | 0.009 | *Eleoscytalopus indigoticus* | 0.957 | 0.225 | 0.464 | 0.007 |
| *Vireo chivi* | 0.916 | 0.392 | 0.599 | 0.001 | *Tityra inquisitor* | 0.937 | 0.216 | 0.450 | 0.004 |
| *Synallaxis spixi* | 0.965 | 0.343 | 0.575 | 0.001 | *Pteroglossus bailloni* | 0.935 | 0.198 | 0.430 | 0.012 |
| *Geothlypis aequinoctialis* | 0.919 | 0.335 | 0.555 | 0.004 | *Hirundo rustica* | 0.970 | 0.154 | 0.387 | 0.019 |
| *Penelope obscura* | 0.942 | 0.318 | 0.548 | 0.004 | *Hemitriccus diops* | 0.944 | 0.150 | 0.376 | 0.026 |
| *Tersina viridis* | 0.925 | 0.302 | 0.529 | 0.008 | *Nonnula rubecula* | 0.944 | 0.137 | 0.359 | 0.030 |
| *Streptoprocne zonaris* | 0.954 | 0.269 | 0.507 | 0.008 | *Drymophila ochropyga* | 1.000 | 0.115 | 0.338 | 0.021 |
| *Lochmias nematura* | 0.936 | 0.253 | 0.487 | 0.011 |  |  |  |  |  |
| *Drymophila malura* | 0.917 | 0.204 | 0.433 | 0.043 | FES+FOM+AS (3) |  |  |  |  |
|  |  |  |  |  | *Turdus leucomelas* | 0.840 | 0.318 | 0.517 | 0.005 |
|  |  |  |  |  | *Crypturellus parvirostris* | 0.921 | 0.229 | 0.459 | 0.003 |
|  |  |  |  |  | *Euscarthmus meloryphus* | 0.887 | 0.162 | 0.379 | 0.011 |
|  |  |  |  |  |  |  |  |  |  |
|  |  |  |  |  | EGL+FES+FOD+FOM (44) |  |  |  |  |
|  |  |  |  |  | *Picumnus temminckii* | 0.990 | 0.415 | 0.641 | 0.002 |
|  |  |  |  |  | *Sicalis flaveola* | 0.992 | 0.393 | 0.624 | 0.009 |
|  |  |  |  |  | *Molothrus bonariensis* | 0.977 | 0.390 | 0.617 | 0.010 |
|  |  |  |  |  | *Sittasomus griseicapillus* | 0.982 | 0.387 | 0.616 | 0.009 |
|  |  |  |  |  | *Turdus albicollis* | 0.989 | 0.383 | 0.616 | 0.006 |
|  |  |  |  |  | *Phalacrocorax brasilianus* | 1.000 | 0.377 | 0.614 | 0.004 |
|  |  |  |  |  | *Aramides saracura* | 0.980 | 0.374 | 0.605 | 0.022 |
|  |  |  |  |  | *Dysithamnus mentalis* | 0.989 | 0.364 | 0.600 | 0.004 |
|  |  |  |  |  | *Chloroceryle americana* | 0.989 | 0.358 | 0.595 | 0.008 |
|  |  |  |  |  | *Pyrrhura frontalis* | 0.970 | 0.361 | 0.592 | 0.022 |
|  |  |  |  |  | *Synallaxis ruficapilla* | 0.982 | 0.355 | 0.590 | 0.016 |
|  |  |  |  |  | *Conopophaga lineata* | 0.973 | 0.355 | 0.587 | 0.016 |
|  |  |  |  |  | *Machetornis rixosa* | 0.994 | 0.345 | 0.586 | 0.008 |
|  |  |  |  |  | *Ardea alba* | 0.978 | 0.345 | 0.581 | 0.020 |
|  |  |  |  |  | *Butorides striata* | 0.984 | 0.339 | 0.577 | 0.021 |
|  |  |  |  |  | *Ramphastos dicolorus* | 0.981 | 0.339 | 0.576 | 0.037 |
|  |  |  |  |  | *Platyrinchus mystaceus* | 0.970 | 0.342 | 0.576 | 0.029 |
|  |  |  |  |  | *Nycticorax nycticorax* | 1.000 | 0.329 | 0.574 | 0.005 |
|  |  |  |  |  | *Chloroceryle amazona* | 0.990 | 0.332 | 0.574 | 0.013 |
|  |  |  |  |  | *Dendrocolaptes platyrostris* | 0.978 | 0.332 | 0.570 | 0.024 |
|  |  |  |  |  | *Megascops choliba* | 0.977 | 0.326 | 0.564 | 0.027 |
|  |  |  |  |  | *Pionus maximiliani* | 0.991 | 0.316 | 0.560 | 0.016 |
|  |  |  |  |  | *Megarynchus pitangua* | 0.980 | 0.320 | 0.560 | 0.039 |
|  |  |  |  |  | *Thalurania glaucopis* | 0.984 | 0.316 | 0.558 | 0.032 |
|  |  |  |  |  | *Egretta thula* | 0.984 | 0.316 | 0.558 | 0.028 |
|  |  |  |  |  | *Progne chalybea* | 0.987 | 0.313 | 0.556 | 0.031 |
|  |  |  |  |  | *Satrapa icterophrys* | 1.000 | 0.304 | 0.551 | 0.012 |
|  |  |  |  |  | *Nyctibius griseus* | 0.988 | 0.307 | 0.551 | 0.026 |
|  |  |  |  |  | *Xiphorhynchus fuscus* | 0.985 | 0.304 | 0.547 | 0.017 |
|  |  |  |  |  | *Lanio melanops* | 0.984 | 0.304 | 0.547 | 0.048 |
|  |  |  |  |  | *Empidonomus varius* | 1.000 | 0.294 | 0.542 | 0.013 |
|  |  |  |  |  | *Pyrocephalus rubinus* | 0.992 | 0.294 | 0.540 | 0.042 |
|  |  |  |  |  | *Florisuga fusca* | 1.000 | 0.291 | 0.539 | 0.011 |
|  |  |  |  |  | *Leptotila rufaxilla* | 1.000 | 0.281 | 0.530 | 0.007 |
|  |  |  |  |  | *Chaetura meridionalis* | 0.985 | 0.272 | 0.517 | 0.033 |
|  |  |  |  |  | *Anthracothorax nigricollis* | 0.988 | 0.268 | 0.515 | 0.038 |
|  |  |  |  |  | *Xiphocolaptes albicollis* | 1.000 | 0.265 | 0.515 | 0.006 |
|  |  |  |  |  | *Bubulcus ibis* | 0.988 | 0.268 | 0.515 | 0.045 |
|  |  |  |  |  | *Tityra cayana* | 0.982 | 0.268 | 0.513 | 0.039 |
|  |  |  |  |  | *Philydor rufum* | 1.000 | 0.262 | 0.512 | 0.017 |
|  |  |  |  |  | *Colonia colonus* | 0.988 | 0.256 | 0.502 | 0.049 |
|  |  |  |  |  | *Lurocalis semitorquatus* | 1.000 | 0.249 | 0.499 | 0.023 |
|  |  |  |  |  | *Geotrygon montana* | 1.000 | 0.243 | 0.493 | 0.019 |
|  |  |  |  |  | *Legatus leucophaius* | 1.000 | 0.243 | 0.493 | 0.032 |
|  |  |  |  |  |  |  |  |  |  |
|  |  |  |  |  | EGL+FES+FOM+AS (38) |  |  |  |  |
|  |  |  |  |  | *Colaptes campestris* | 0.947 | 0.445 | 0.649 | 0.001 |
|  |  |  |  |  | *Falco sparverius* | 0.986 | 0.411 | 0.637 | 0.001 |
|  |  |  |  |  | *Cyclarhis gujanensis* | 0.943 | 0.419 | 0.628 | 0.015 |
|  |  |  |  |  | *Chlorostilbon lucidus* | 0.974 | 0.400 | 0.624 | 0.001 |
|  |  |  |  |  | *Piaya cayana* | 0.938 | 0.415 | 0.624 | 0.013 |
|  |  |  |  |  | *Tyrannus savana* | 0.952 | 0.404 | 0.620 | 0.001 |
|  |  |  |  |  | *Zenaida auriculata* | 0.988 | 0.377 | 0.611 | 0.001 |
|  |  |  |  |  | *Camptostoma obsoletum* | 0.910 | 0.400 | 0.603 | 0.016 |
|  |  |  |  |  | *Serpophaga subcristata* | 0.979 | 0.359 | 0.592 | 0.001 |
|  |  |  |  |  | *Volatinia jacarina* | 0.935 | 0.374 | 0.591 | 0.001 |
|  |  |  |  |  | *Patagioenas picazuro* | 0.964 | 0.359 | 0.588 | 0.001 |
|  |  |  |  |  | *Amazonetta brasiliensis* | 0.960 | 0.343 | 0.574 | 0.006 |
|  |  |  |  |  | *Ammodramus humeralis* | 1.000 | 0.317 | 0.563 | 0.001 |
|  |  |  |  |  | *Gallinula galeata* | 0.937 | 0.336 | 0.561 | 0.014 |
|  |  |  |  |  | *Tachycineta leucorrhoa* | 0.953 | 0.313 | 0.546 | 0.009 |
|  |  |  |  |  | *Turdus subalaris* | 0.983 | 0.302 | 0.545 | 0.001 |
|  |  |  |  |  | *Mimus saturninus* | 0.967 | 0.302 | 0.540 | 0.003 |
|  |  |  |  |  | *Gnorimopsar chopi* | 0.979 | 0.298 | 0.540 | 0.001 |
|  |  |  |  |  | *Melanerpes candidus* | 0.971 | 0.294 | 0.535 | 0.002 |
|  |  |  |  |  | *Myiarchus swainsoni* | 0.920 | 0.309 | 0.534 | 0.019 |
|  |  |  |  |  | *Lanio cucullatus* | 0.976 | 0.279 | 0.522 | 0.003 |
|  |  |  |  |  | *Nothura maculosa* | 1.000 | 0.268 | 0.518 | 0.001 |
|  |  |  |  |  | *Amazona aestiva* | 0.990 | 0.268 | 0.515 | 0.002 |
|  |  |  |  |  | *Tringa flavipes* | 0.929 | 0.283 | 0.513 | 0.024 |
|  |  |  |  |  | *Dendrocygna viduata* | 0.982 | 0.257 | 0.502 | 0.002 |
|  |  |  |  |  | *Chrysomus ruficapillus* | 0.989 | 0.249 | 0.496 | 0.003 |
|  |  |  |  |  | *Rhynchotus rufescens* | 1.000 | 0.245 | 0.495 | 0.003 |
|  |  |  |  |  | *Nystalus chacuru* | 1.000 | 0.223 | 0.472 | 0.004 |
|  |  |  |  |  | *Sicalis luteola* | 0.996 | 0.219 | 0.467 | 0.007 |
|  |  |  |  |  | *Columbina squammata* | 0.995 | 0.219 | 0.467 | 0.006 |
|  |  |  |  |  | *Heterospizias meridionalis* | 0.936 | 0.223 | 0.457 | 0.017 |
|  |  |  |  |  | *Emberizoides herbicola* | 0.979 | 0.208 | 0.451 | 0.004 |
|  |  |  |  |  | *Falco femoralis* | 0.956 | 0.208 | 0.445 | 0.023 |
|  |  |  |  |  | *Geranoaetus albicaudatus* | 0.979 | 0.193 | 0.434 | 0.015 |
|  |  |  |  |  | *Tachybaptus dominicus* | 0.970 | 0.189 | 0.428 | 0.015 |
|  |  |  |  |  | *Cypseloides senex* | 1.000 | 0.143 | 0.379 | 0.030 |
|  |  |  |  |  | *Falco peregrinus* | 0.990 | 0.136 | 0.367 | 0.048 |
|  |  |  |  |  | *Xolmis velatus* | 1.000 | 0.117 | 0.342 | 0.041 |
|  |  |  |  |  |  |  |  |  |  |
|  |  |  |  |  | EGL+FOD+FOM+AS (4) |  |  |  |  |
|  |  |  |  |  | *Synallaxis spixi* | 0.915 | 0.369 | 0.581 | 0.008 |
|  |  |  |  |  | *Penelope obscura* | 0.918 | 0.345 | 0.563 | 0.027 |
|  |  |  |  |  | *Gallinago paraguaiae* | 0.953 | 0.255 | 0.493 | 0.003 |
|  |  |  |  |  | *Tringa melanoleuca* | 0.946 | 0.212 | 0.448 | 0.007 |
|  |  |  |  |  |  |  |  |  |  |
|  |  |  |  |  | FES+FOD+FOM+AS (1) |  |  |  |  |
|  |  |  |  |  | *Sirystes sibilator* | 0.933 | 0.246 | 0.479 | 0.009 |
