# Supplementary material for: Traditional scientific data vs. uncoordinated citizen science effort: A review of the current status and comparison of data on avifauna in Southern Brazil
Source: PLoS One. 2017 Dec 11;12(12):e0188819. doi: 10.1371/journal.pone.0188819 (PMC5724844; doi:10.1371/journal.pone.0188819)
Supplement: S8 Table — *Species extinct in the state of Paraná. Excluded species: 1 Species of marginal occurrence in shown vegetation type. 2 Migrants, partial migrants and species that perform seasonal dispersal, nomadic or other with poorly known mobility [61]. 3 Seabirds. Vegetation type: EGL–Grassland; FES–Semideciduous Tropical Forest; FOD–Tropical Rainforest; FOM–Araucaria Moist Forest. Species are organized in alphabetical order. Global and of Paraná state (PR) threat categories of the species, according to IUCN [96] and Straube et al. [94]: LC–Least Concern; NT–Near Threatened; VU–Vulnerable; EN–Endangered; CR–Critically Endangered; RE–Regionally Extinct; DD–Data Deficient; - –Not Evaluated. (DOCX) [file pone.0188819.s008.docx]

**S8 Table.** Extinct species in each vegetation type in Paraná state considering only data from traditional scientific references (BM) and including CS data (BM+CS). *Species extinct in the state of Paraná. Excluded species: ^1^ Species of marginal occurrence in shown vegetation type. ^2^ Migrants, partial migrants and species that perform seasonal dispersal, nomadic or other with poorly known mobility [61]. ^3^ Seabirds. Vegetation type: **EGL** – Grassland; **FES** – Semideciduous Tropical Forest; **FOD** – Tropical Rainforest; **FOM** – Araucaria Moist Forest. Species are organized in alphabetical order. Global and of Paraná state (PR) threat categories of the species, according to IUCN [96] and Straube et al. [94]: **LC** – Least Concern; **NT** – Near Threatened; **VU** – Vulnerable; **EN** – Endangered; **CR** – Critically Endangered; **RE** – Regionally Extinct; **DD** – Data Deficient; **-** – Not Evaluated.

| **Extinct** | | | | | **Excluded** | | | | |
| --- | --- | --- | --- | --- | --- | --- | --- | --- | --- |
| **Taxon** | **Vegetation type** | | **Threat categories** | | **Taxon** | **Vegetation type** | | **Threat categories** | |
|  | **BM** | **BM+CS** | **Global** | **PR** |  | **BM** | **BM+CS** | **Global** | **PR** |
| **After 1959** |  |  |  |  | **After 1959** |  |  |  |  |
| *Antilophia galeata* | FES* |  | LC | CR | *Antrostomus sericocaudatus^1^* | EGL | EGL | LC | EN |
| *Carpornis cucullata* | FES | FES | NT | - | *Ara chloropterus^1^* | EGL | EGL | LC | CR |
| *Cochlearius cochlearius* | FES, FOD* | FES | LC | DD | *Aratinga auricapillus^1^* | EGL | EGL | LC | - |
| *Geotrygon violacea* | FES | FES | LC | DD | *Buteo swainsoni^2^* | FES |  | LC | DD |
| *Harpia harpyja* | FES | FES | NT | CR | *Cantorchilus longirostris^1^* | EGL | EGL | LC | - |
| *Hemitriccus orbitatus* | FES | FES | NT | - | *Charadrius semipalmatus^2^* | FES | FES | LC | - |
| *Herpsilochmus atricapillus* | FES* | FES* | LC | RE | *Cyanocorax caeruleus^1^* | FES | FES | NT | - |
| *Ibycter americanus* | FES* | FES* | LC | RE | *Dryocopus galeatus^1^* | EGL | EGL | VU | CR |
| *Ilicura militaris* | FES | FES | LC | - | *Elaenia chiriquensis^2^* | FES, FOD, FOM | FOD, FOM | LC | - |
| *Malacoptila striata* | FES |  | NT | - | *Emberizoides ypiranganus^1^* | FOM |  | LC | - |
| *Mergus octosetaceus* | FES | FES | CR | CR | *Gallinago undulata^1^* | FOM |  | LC | DD |
| *Myrmoderus squamosus* | FOM |  | LC | - | *Lophornis magnificus^2^* | EGL | EGL | LC | DD |
| *Onychorhynchus swainsoni* | FES | FES | VU | DD | *Myiarchus tyrannulus^2^* | FOM |  | LC | - |
| *Orthogonys chloricterus* | FES | FES | LC | - | *Nomonyx dominica^2^* | FES |  | LC | DD |
| *Phylloscartes difficilis* | FOM | FOM | NT | VU | *Notharchus swainsoni^2^* | FOM |  | LC | - |
| *Phylloscartes paulista* | FES | FES | NT | NT | *Phaetusa simplex^2^* | FOD | FOD | LC | - |
| *Piculus flavigula* | FES | FES | LC | NT | *Primolius maracana^1^* | EGL | EGL | NT | EN |
| *Picumnus cirratus* | EGL |  | LC | - | *Sporophila nigricollis^2^* | FOD | FOD | LC | - |
| *Psarocolius decumanus* | FES | FES | LC | CR | *Turdus flavipes^1^* | FES |  | LC | - |
| *Ramphodon naevius* | FOM | FOM | NT | - | *Xolmis cinereus^2^* | FOD | FOD | LC | - |
| *Ramphotrigon megacephalum* | FES |  | LC | DD | **After 1989** |  |  |  |  |
| *Rhopias gularis* | EGL, FES | FES | LC | - | *Alectrurus tricolor^2^* | FES | FES | VU | EN |
| *Syndactyla dimidiata* | FES* | FES* | LC | CR | *Anas bahamensis^2^* | FES |  | LC | - |
| *Taoniscus nanus* | EGL* | EGL* | VU | RE | *Anas georgica^2^* | FOD | FOD | LC | - |
| **After 1989** |  |  |  |  | *Anumbius annumbi^1^* | FOD | FOD | LC | - |
| *Agelaioides badius* | FOD |  | LC | - | *Aramides cajaneus^1^* | FOM | FOM | LC | - |
| *Anhinga anhinga* | EGL, FOD |  | LC | - | *Botaurus pinnatus^4^* | FES | FES | LC | DD |
| *Aramides ypecaha* | FOD | FOD | LC | DD | *Buteo swainsoni^2^* | FOD | FOD | LC | DD |
| *Asio clamator* | FES |  | LC | - | *Calonectris edwardsii^2,3^* | * |  | NT | - |
| *Atticora tibialis* | FES* | FES* | LC | - | *Cariama cristata^1^* | FOM |  | LC | NT |
| *Chauna torquata* | FOD* | FOD* | LC | - | *Casiornis rufus^2^* | FES* |  | LC | - |
| *Chondrohierax uncinatus* | FES |  | LC | VU | *Charadrius modestus^2^* | FES, FOD* | FES | LC | - |
| *Falco femoralis* | FOD |  | LC | - | *Charadrius semipalmatus^2^* | EGL | EGL | LC | - |
| *Hylophilus amaurocephalus* | FES |  | LC | DD | *Chionis albus^2,3^* | * | * | LC | - |
| *Jacamaralcyon tridactyla* | FES* | FES* | VU | DD | *Chordeiles nacunda^2^* | FOD |  | LC | - |
| *Lepidocolaptes falcinellus* | FES |  | LC | - | *Chrysolampis mosquitus^2^* | FOD | FOD | LC | - |
| *Mergus octosetaceus* | FOM* | FOM* | CR | CR | *Culicivora caudacuta^1^* | FOM |  | VU | VU |
| *Mimus gilvus* | FOD* | FOD* | LC | - | *Dendrocincla turdina^1^* | FOM | FOM | LC | - |
| *Morphnus guianensis* | FES* | FES* | NT | RE | *Diomedea exulans^2,3^* | * | * | VU | VU |
| *Pardirallus sanguinolentus* | FOD |  | LC | - | *Dolichonyx oryzivorus^2^* | FES | FES | LC | - |
| *Pilherodius pileatus* | FOD |  | LC | NT | *Donacospiza albifrons^1^* | FOD | FOD | LC | - |
| *Plegadis chihi* | FOD |  | LC | NT | *Drymophila squamata^1^* | FOM | FOM | LC | - |
| *Podilymbus podiceps* | FOD |  | LC | - | *Falco peregrinus^2^* | FOD | FOD | LC | - |
| *Pseudastur polionotus* | FES |  | NT | NT | *Geranoaetus melanoleucus^1^* | FES | FES | LC | - |
| *Rostrhamus sociabilis* | FOD |  | LC | - | *Heliomaster furcifer^2^* | FES* |  | LC | - |
| *Sarcoramphus papa* | FOD |  | LC | - | *Heliomaster longirostris^2^* | FES* | FES* | LC | - |
| *Serpophaga nigricans* | FOD |  | LC | - | *Hylocharis chrysura^2^* | EGL |  | LC | - |
| *Spizaetus melanoleucus* | FOD |  | LC | EN | *Hymenops perspicillatus^2^* | EGL, FOM | EGL, FOM | LC | NT |
| *Spizaetus ornatus* | FOD |  | NT | EN | *Ictinia plumbea^2^* | FOD |  | LC | - |
| *Sporophila angolensis* | FES |  | LC | VU | *Laniisoma elegans^2^* | EGL | EGL | LC | DD |
|  |  |  |  |  | *Lophornis magnificus^2^* | FOD* | FOD* | LC | DD |
|  |  |  |  |  | *Myiothlypis rivularis^1^* | EGL |  | LC | - |
|  |  |  |  |  | *Nomonyx dominica^2^* | FOD |  | LC | - |
|  |  |  |  |  | *Oceanites oceanicus^2,3^* | * | * | LC | - |
|  |  |  |  |  | *Pachyptila belcheri^2,3^* | * | * | LC | - |
|  |  |  |  |  | *Pardirallus maculatus^1^* | EGL | EGL | LC | DD |
|  |  |  |  |  | *Phalaropus tricolor^2^* | FOD |  | LC | - |
|  |  |  |  |  | *Picumnus nebulosus^1^* | FOD |  | LC | - |
|  |  |  |  |  | *Podicephorus major^2^* | FOD* |  | LC | - |
|  |  |  |  |  | *Pyrrhocoma ruficeps^1^* | FOD | FOD | LC | - |
|  |  |  |  |  | *Riparia riparia^2^* | FES, FOD* | FOD | LC | - |
|  |  |  |  |  | *Rollandia rolland^2^* | FOM | FOM | LC | - |
|  |  |  |  |  | *Sicalis luteola^1^* | FOD | FOD | LC | - |
|  |  |  |  |  | *Stercorarius antarcticus^2,3^* | * |  | LC | - |
|  |  |  |  |  | *Stercorarius maccormicki^2,3^* | * |  | LC | - |
|  |  |  |  |  | *Tachybaptus dominicus^1^* | FOD | FOD | LC | - |
|  |  |  |  |  | *Thalassarche chrysostoma^2,3^* | * | * | EN | - |
|  |  |  |  |  | *Vanellus cayanus^2^* | FES | FES | LC | DD |
|  |  |  |  |  | *Xolmis irupero^2^* | FOM* |  | LC | EN |
