# Supplementary material for: Traditional scientific data vs. uncoordinated citizen science effort: A review of the current status and comparison of data on avifauna in Southern Brazil
Source: PLoS One. 2017 Dec 11;12(12):e0188819. doi: 10.1371/journal.pone.0188819 (PMC5724844; doi:10.1371/journal.pone.0188819)
Supplement: S9 Table — Vegetation type (Veg. type): EGL–Grassland; FES–Semideciduous Tropical Forest; FOD–Tropical Rainforest; FOM–Araucaria Moist Forest. (DOCX) [file pone.0188819.s009.docx]

**S9 Table.** Species that show local population increases and native invasive species (regional population and geographic distribution increases) in each vegetation type in Paraná state considering only data from traditional scientific references (BM), and including CS data (BM+CS). Vegetation type (Veg. type): **EGL** – Grassland; **FES** – Semideciduous Tropical Forest; **FOD** – Tropical Rainforest; **FOM** – Araucaria Moist Forest.

| **Native Invasive Species** | | | **Local Population Increase** | | |
| --- | --- | --- | --- | --- | --- |
| **Taxon** | **Veg. type** | | **Taxon** | **Veg. type** | |
|  | **BM** | **BM+CS** |  | **BM** | **BM+CS** |
| *Aramides saracura* | FES, FOM | EGL, FES | *Aburria jacutinga* |  | FOD |
| *Ardea alba* | EGL, FES, FOD, FOM | EGL, FES, FOD, FOM | *Agelasticus thilius* | FOD | FOD |
| *Ardea cocoi* | FOM | FOD, FOM | *Amazilia fimbriata* |  | FOD |
| *Athene cunicularia* | FES, FOM | FES, FOD, FOM | *Amazilia lactea* |  | FES |
| *Bubulcus ibis* | EGL, FES, FOM | EGL, FES, FOM | *Amazona aestiva* | FOM | FOM |
| *Camptostoma obsoletum* | FES, FOD, FOM |  | *Amazonetta brasiliensis* | FOD | EGL |
| *Caracara plancus* | FES, FOM | EGL, FES | *Ammodramus humeralis* | FES | FES |
| *Cathartes aura* | EGL, FOD | EGL, FOD | *Anabacerthia lichtensteini* | FOD | FOD |
| *Chaetura cinereiventris* | EGL, FES, FOD |  | *Anthracothorax nigricollis* |  | FES |
| *Cnemotriccus fuscatus* | FES, FOD |  | *Arremon semitorquatus* | FOD | FOD |
| *Colaptes campestris* | FES, FOM | FES, FOM | *Asio stygius* | FOD |  |
| *Columbina talpacoti* | EGL, FES, FOD, FOM | EGL, FES | *Brotogeris chiriri* |  | FES |
| *Coragyps atratus* | EGL, FES, FOD, FOM | EGL, FES, FOD, FOM | *Brotogeris tirica* | EGL | EGL |
| *Crypturellus obsoletus* | FES, FOD |  | *Buteo brachyurus* | FES | FES |
| *Crypturellus tataupa* | FES, FOD |  | *Butorides striata* |  | EGL |
| *Cyclarhis gujanensis* | FES, FOD |  | *Cairina moschata* | FOD |  |
| *Dendrocygna viduata* |  | FES, FOM | *Campephilus robustus* |  | FOD |
| *Dromococcyx pavoninus* | FES, FOD |  | *Carpornis melanocephala* | FOD | FOD |
| *Egretta thula* |  | EGL, FES, FOD | *Certhiaxis cinnamomeus* | FOD | FOD |
| *Elaenia flavogaster* | EGL, FES, FOD, FOM | FES, FOM | *Chaetura meridionalis* | EGL | EGL |
| *Elanoides forficatus* | FES, FOM | FES, FOM | *Charadrius semipalmatus* |  | FOD |
| *Eleoscytalopus indigoticus* | FES, FOD |  | *Chloroceryle aenea* | FOD |  |
| *Emberizoides herbicola* |  | EGL, FOM | *Chloroceryle inda* | FOD |  |
| *Eupetomena macroura* | FES, FOD | FES, FOD | *Chlorophanes spiza* | FOD | FOD |
| *Furnarius rufus* | EGL, FES, FOM | EGL, FES, FOM | *Chlorostilbon lucidus* | FOM | FOM |
| *Gallinula galeata* | EGL, FOD | EGL, FES, FOD | *Chondrohierax uncinatus* | FOD |  |
| *Guira guira* | EGL, FES, FOM |  | *Chrysomus ruficapillus* | EGL | EGL |
| *Hydropsalis albicollis* | EGL, FOD |  | *Cnemotriccus fuscatus* |  | FOD |
| *Machetornis rixosa* | EGL, FES, FOD, FOM | FES, FOD | *Coereba flaveola* | FES | FES |
| *Megarynchus pitangua* |  | FES, FOM | *Colaptes melanochloros* | FOD | FOD |
| *Melanerpes candidus* | FES, FOD, FOM | FES, FOM | *Colonia colonus* | FES | FES |
| *Molothrus bonariensis* | EGL, FOM | EGL, FOM | *Columbina picui* | FES |  |
| *Nyctibius griseus* |  | FES, FOM | *Columbina squammata* |  | FOM |
| *Nycticorax nycticorax* | EGL, FOM | EGL, FOD | *Conirostrum bicolor* | FOD |  |
| *Pachyramphus validus* |  | FES, FOM | *Conirostrum speciosum* | FES | FES |
| *Patagioenas picazuro* | EGL, FES, FOM | EGL, FES, FOM | *Conopias trivirgatus* | FOD | FOD |
| *Phalacrocorax brasilianus* | EGL, FOM | EGL, FOM | *Cranioleuca obsoleta* | FOM |  |
| *Piaya cayana* | FES, FOM | FES, FOM | *Crotophaga ani* | FES | FES |
| *Pipraeidea bonariensis* |  | EGL, FOM | *Crypturellus noctivagus* | FOD | FOD |
| *Pitangus sulphuratus* | FES, FOD, FOM | FES, FOM | *Cyanoloxia brissonii* | FES |  |
| *Podilymbus podiceps* |  | EGL, FOM | *Cyanoloxia glaucocaerulea* |  | FOM |
| *Poecilotriccus plumbeiceps* | FES, FOM |  | *Cyclarhis gujanensis* |  | FES |
| *Progne chalybea* | EGL, FOD |  | *Dendrocincla turdina* | FES |  |
| *Progne tapera* |  | EGL, FES, FOD | *Dendrocygna viduata* | FOM |  |
| *Pygochelidon cyanoleuca* | EGL, FES, FOM | EGL, FES | *Dromococcyx pavoninus* |  | FES |
| *Pyrocephalus rubinus* |  | FES, FOD, FOM | *Drymophila ochropyga* | FOD | FOD |
| *Rupornis magnirostris* | EGL, FES | EGL, FES | *Dysithamnus stictothorax* | FOD | FOD |
| *Saltator similis* | FES, FOD, FOM | FES, FOM | *Egretta thula* | FOD |  |
| *Setophaga pitiayumi* | FES, FOD, FOM | FES, FOD, FOM | *Elaenia parvirostris* |  | FES |
| *Sicalis flaveola* | FES, FOD, FOM | FES, FOD, FOM | *Elaenia spectabilis* | FES | FES |
| *Sporagra magellanica* | EGL, FES, FOM | EGL, FES, FOM | *Elanus leucurus* |  | FES |
| *Sturnella superciliaris* |  | EGL, FES, FOD | *Eleoscytalopus indigoticus* |  | FES |
| *Synallaxis ruficapilla* | EGL, FES |  | *Emberizoides herbicola* | FOM |  |
| *Synallaxis spixi* | FES, FOD |  | *Empidonomus varius* | FES | FES |
| *Tachyphonus coronatus* |  | EGL, FES | *Euphonia chalybea* |  | FOD |
| *Tangara sayaca* | EGL, FES | EGL, FES | *Euphonia chlorotica* | FES | FES |
| *Tersina viridis* | FES, FOD |  | *Falco peregrinus* |  | FES |
| *Theristicus caudatus* |  | FES, FOM | *Florisuga fusca* | FOM | FOM |
| *Tringa melanoleuca* | EGL, FOM |  | *Fluvicola nengeta* |  | FES |
| *Troglodytes musculus* | EGL, FES, FOD, FOM | EGL, FES, FOM | *Forpus xanthopterygius* | FOD | FOD |
| *Turdus albicollis* | FES, FOM |  | *Geranoaetus albicaudatus* | FES | FES |
| *Turdus amaurochalinus* | FES, FOD | FES, FOD | *Grallaria varia* | FES |  |
| *Turdus rufiventris* | EGL, FES, FOM | EGL, FES, FOM | *Guira guira* |  | FES |
| *Tyrannus melancholicus* | EGL, FES, FOD, FOM | EGL, FES, FOM | *Haplospiza unicolor* | FOD |  |
| *Tyrannus savana* | EGL, FES, FOM | EGL, FES, FOM | *Hemitriccus orbitatus* |  | FOD |
| *Vanellus chilensis* | EGL, FES, FOD, FOM | EGL, FES | *Herpetotheres cachinnans* |  | FOD |
| *Volatinia jacarina* | EGL, FES | FES, FOD | *Herpsilochmus rufimarginatus* | FOD | FOD |
| *Xenops rutilans* | FES, FOD | FES, FOD | *Himantopus melanurus* |  | FES |
| *Zenaida auriculata* | EGL, FOM | EGL, FES, FOM | *Hydropsalis albicollis* |  | EGL |
| *Zonotrichia capensis* | FES, FOD, FOM | FES, FOM | *Hylocharis chrysura* |  | FES |
|  |  |  | *Hypoedaleus guttatus* | FOD |  |
|  |  |  | *Ilicura militaris* |  | FOD |
|  |  |  | *Jacana jacana* | EGL | EGL |
|  |  |  | *Knipolegus nigerrimus* |  | FOD |
|  |  |  | *Laterallus melanophaius* |  | FOD |
|  |  |  | *Lathrotriccus euleri* | FES |  |
|  |  |  | *Leptotila rufaxilla* | EGL | EGL |
|  |  |  | *Leucochloris albicollis* | FOM |  |
|  |  |  | *Lophornis chalybeus* |  | FOD |
|  |  |  | *Mackenziaena severa* | FOD |  |
|  |  |  | *Megarynchus pitangua* | FES |  |
|  |  |  | *Melanerpes flavifrons* |  | FES |
|  |  |  | *Milvago chimachima* | FES | FES |
|  |  |  | *Mimus saturninus* | FES | FES |
|  |  |  | *Muscipipra vetula* | FOD |  |
|  |  |  | *Mycteria americana* | FES | FES |
|  |  |  | *Myiarchus tyrannulus* |  | FES |
|  |  |  | *Myiopagis caniceps* | FES | FES |
|  |  |  | *Myiornis auricularis* | FES | FOD |
|  |  |  | *Myiothlypis rivularis* |  | FOD |
|  |  |  | *Myiozetetes similis* | FES |  |
|  |  |  | *Nonnula rubecula* |  | FES |
|  |  |  | *Nyctibius griseus* | FES |  |
|  |  |  | *Pachyramphus castaneus* |  | FOM |
|  |  |  | *Pachyramphus validus* | FES |  |
|  |  |  | *Pachyramphus viridis* | FES |  |
|  |  |  | *Phaethornis eurynome* | FES | FES |
|  |  |  | *Phaethornis squalidus* | FOD | FOD |
|  |  |  | *Phylloscartes paulista* |  | FOD |
|  |  |  | *Pionus maximiliani* | EGL | EGL |
|  |  |  | *Pipraeidea bonariensis* | FOM |  |
|  |  |  | *Pipraeidea melanonota* | FOD | FOD |
|  |  |  | *Podilymbus podiceps* | FOM |  |
|  |  |  | *Poecilotriccus plumbeiceps* |  | FES |
|  |  |  | *Progne chalybea* |  | EGL |
|  |  |  | *Progne tapera* | FES |  |
|  |  |  | *Pseudoleistes guirahuro* |  | FES |
|  |  |  | *Pteroglossus bailloni* | FOD |  |
|  |  |  | *Pulsatrix koeniswaldiana* | FOD |  |
|  |  |  | *Ramphastos vitellinus* | FOD | FOD |
|  |  |  | *Ramphocaenus melanurus* | FOD | FOD |
|  |  |  | *Serpophaga subcristata* | FES | FES |
|  |  |  | *Sirystes sibilator* |  | FOD |
|  |  |  | *Spizaetus tyrannus* |  | FOM |
|  |  |  | *Sporophila caerulescens* | FES | FES |
|  |  |  | *Sporophila frontalis* | FOD | FOD |
|  |  |  | *Sporophila hypoxantha* |  | EGL |
|  |  |  | *Sporophila lineola* | FES | FES |
|  |  |  | *Stelgidopteryx ruficollis* | EGL |  |
|  |  |  | *Sturnella superciliaris* | FES |  |
|  |  |  | *Synallaxis frontalis* | FES | FES |
|  |  |  | *Synallaxis ruficapilla* |  | FES |
|  |  |  | *Synallaxis spixi* |  | FES |
|  |  |  | *Syndactyla rufosuperciliata* | FES | FES |
|  |  |  | *Syrigma sibilatrix* | FES | FES |
|  |  |  | *Tachybaptus dominicus* |  | FES |
|  |  |  | *Tachycineta albiventer* |  | FES |
|  |  |  | *Tachyphonus coronatus* | FES |  |
|  |  |  | *Tangara palmarum* |  | FOD |
|  |  |  | *Tersina viridis* |  | FES |
|  |  |  | *Thalasseus acuflavidus* | FOD | FOD |
|  |  |  | *Thalurania glaucopis* |  | FOM |
|  |  |  | *Thamnophilus ruficapillus* | FES | FES |
|  |  |  | *Theristicus caudatus* | FOM |  |
|  |  |  | *Tityra cayana* | FES |  |
|  |  |  | *Todirostrum cinereum* | FES | FES |
|  |  |  | *Todirostrum poliocephalum* |  | FOD |
|  |  |  | *Tringa flavipes* |  | EGL |
|  |  |  | *Tringa melanoleuca* |  | FOM |
|  |  |  | *Trogon rufus* | FES | FES |
|  |  |  | *Trogon surrucura* | FES | FES |
|  |  |  | *Turdus albicollis* |  | FES |
|  |  |  | *Turdus leucomelas* | FOM | FOM |
|  |  |  | *Turdus subalaris* | FES | FES |
|  |  |  | *Vireo chivi* | FES |  |
|  |  |  | *Xiphorhynchus fuscus* | EGL | EGL |
