# Supplementary material for: Traditional scientific data vs. uncoordinated citizen science effort: A review of the current status and comparison of data on avifauna in Southern Brazil
Source: PLoS One. 2017 Dec 11;12(12):e0188819. doi: 10.1371/journal.pone.0188819 (PMC5724844; doi:10.1371/journal.pone.0188819)
Supplement: S10 Table — Number of records, species, sources of information and sites per microregions in Paraná considering only data from traditional scientific references (BM), and including CS data (BM+CS). Microregions are presented in alphabetical order. (DOCX) [file pone.0188819.s010.docx]

**S10 Table.** Level of avifaunal knowledge in Paraná state. Number of records, species, sources of information and sites per microregions in Paraná considering only data from traditional scientific references (BM), and including CS data (BM+CS). Microregions are presented in alphabetical order.

| **Microregions** | **Number of records** | | **Number of species** | | **Sources of information** | | **Number of sites** | |
| --- | --- | --- | --- | --- | --- | --- | --- | --- |
|  | **BM** | **BM+CS** | **BM** | **BM+CS** | **BM** | **BM+CS** | **BM** | **BM+CS** |
| Apucarana | 181 | 5259 | 144 | 339 | 6 | 63 | 6 | 15 |
| Assaí | 20 | 125 | 17 | 73 | 4 | 20 | 4 | 10 |
| Astorga | 103 | 480 | 76 | 177 | 5 | 43 | 7 | 26 |
| Campo Mourão | 1150 | 3168 | 308 | 397 | 14 | 69 | 30 | 52 |
| Capanema | 3 | 632 | 2 | 222 | 2 | 22 | 2 | 8 |
| Cascavel | 1010 | 3463 | 241 | 320 | 8 | 80 | 26 | 40 |
| Cerro Azul | 215 | 402 | 117 | 194 | 13 | 37 | 15 | 20 |
| Cianorte | 3 | 380 | 3 | 153 | 1 | 21 | 1 | 9 |
| Cornelio Procópio | 457 | 901 | 204 | 262 | 11 | 49 | 17 | 28 |
| Curitiba | 38893 | 53066 | 469 | 500 | 109 | 700 | 340 | 574 |
| Faxinal | 17 | 323 | 16 | 155 | 3 | 24 | 4 | 11 |
| Floraí | 156 | 271 | 81 | 129 | 3 | 15 | 7 | 13 |
| Foz do Iguaçu | 978 | 3830 | 290 | 364 | 21 | 390 | 34 | 49 |
| Francisco Beltrão | 48 | 397 | 44 | 157 | 4 | 34 | 7 | 17 |
| Goioêre | 4 | 188 | 4 | 114 | 1 | 18 | 1 | 8 |
| Guarapuava | 1668 | 3999 | 349 | 381 | 22 | 111 | 71 | 90 |
| Ibaiti | 20 | 343 | 15 | 194 | 4 | 20 | 7 | 15 |
| Irati | 390 | 5063 | 204 | 339 | 9 | 68 | 9 | 15 |
| Ivaiporã | 944 | 2611 | 275 | 391 | 11 | 29 | 17 | 32 |
| Jacarezinho | 261 | 800 | 177 | 287 | 11 | 44 | 13 | 18 |
| Jaguariaíva | 2094 | 3067 | 358 | 385 | 25 | 73 | 51 | 63 |
| Lapa | 195 | 2538 | 147 | 303 | 6 | 49 | 10 | 14 |
| Londrina | 1874 | 8149 | 369 | 415 | 46 | 178 | 30 | 39 |
| Maringá | 432 | 2045 | 165 | 239 | 11 | 98 | 13 | 18 |
| Palmas | 644 | 1846 | 250 | 311 | 12 | 46 | 26 | 37 |
| Paranaguá | 7661 | 22465 | 545 | 582 | 154 | 626 | 340 | 512 |
| Paranavaí | 1443 | 1948 | 338 | 359 | 25 | 72 | 56 | 83 |
| Pato Branco | 511 | 1212 | 265 | 281 | 4 | 27 | 8 | 18 |
| Pitanga | 52 | 394 | 52 | 179 | 2 | 20 | 3 | 9 |
| Ponta Grossa | 2960 | 5944 | 385 | 407 | 54 | 200 | 62 | 87 |
| Porecatu | 168 | 430 | 142 | 187 | 8 | 41 | 7 | 14 |
| Prudentópolis | 361 | 1120 | 187 | 264 | 12 | 74 | 19 | 26 |
| Rio Negro | 685 | 1503 | 247 | 282 | 11 | 67 | 21 | 29 |
| São Mateus do Sul | 126 | 390 | 68 | 177 | 11 | 40 | 14 | 20 |
| Telêmaco Borba | 1605 | 4025 | 392 | 421 | 22 | 156 | 30 | 42 |
| Toledo | 537 | 2875 | 251 | 364 | 27 | 97 | 35 | 62 |
| Umuarama | 1282 | 2211 | 321 | 353 | 20 | 57 | 36 | 64 |
| União da Vitória | 1181 | 1658 | 270 | 286 | 21 | 54 | 36 | 45 |
| Wenceslau Braz | 14 | 293 | 10 | 139 | 4 | 22 | 6 | 15 |
| **Total** | **70346** | **149815** | **747** | **766** | **417** | **1882** | **1421** | **2247** |
